# Supplementary material for: Real-space recipes for general topological crystalline states
Source: Nat Commun. 2020 Aug 21;11:4197. doi: 10.1038/s41467-020-17685-5 (PMC7442842; doi:10.1038/s41467-020-17685-5)
Supplement: Supplementary file 1 — Supplementary Information [file 41467_2020_17685_MOESM1_ESM.pdf]

Supplemental Information: Real-space recipes for general topological crystalline states

Zhida Song,[1,2,*] Chen Fang,[1,3,4,5,†] and Yang Qi[6,7,8,‡]

[1] *Beijing National Research Center for Condensed Matter Physics, and Institute of Physics, Chinese Academy of Sciences, Beijing 100190, China*
[2] *University of Chinese Academy of Sciences, Beijing 100049, China*
[3] *Kavli Institute for Theoretical Sciences, Chinese Academy of Sciences, Beijing 100190, China*
[4] *CAS Center for Excellence in Topological Quantum Computation, Beijing, China*
[5] *Songshan Lake Laboratory For Materials Science, Guangdong 523808, China*
[6] *State Key Laboratory of Surface Physics, Fudan University, Shanghai 200433, China*
[7] *Center for Field Theory and Particle Physics, Department of Physics, Fudan University, Shanghai 200433, China*
[8] *Collaborative Innovation Center of Advanced Microstructures, Nanjing 210093, China*

(Dated: July 4, 2020)

CONTENTS

List of Tables 1

I. Review of onsite-symmetry SPT classification 1
 A. Abstract description 1
 B. Bosonic SPT and group cohomology 2
 C. Interacting-fermion SPTs 4

II. Symmetry actions on cochains and cocycles 5

III. Transfer maps 6

IV. Spectral sequence and equivariant cohomology 7

V. Proof of trivial d_2 maps and trivial group extensions when $G = SG \times G_0$. 7

VI. 2-cells and 1-cells in space group $P\bar{4}n2$ 8

VII. Full classification for bosonic TCS 9

References 44

LIST OF TABLES

I Classifications of 2D SPT phases. 10
II Classifications of 3D SPT phases. 13

References to the sections, equations and figures in the main text is denoted by the prefix M-.

* Current address: Department of Physics, Princeton University, Princeton, New Jersey 08544, USA.
† cfang@iphy.ac.cn
‡ qiyang@fudan.edu.cn

I. REVIEW OF ONSITE-SYMMETRY SPT CLASSIFICATION

In this section, we briefly review the classification of SPT states protected solely by onsite symmetries. We first give some general and abstract description of such classification in various systems, including bosonic systems (which are necessarily interacting), free-fermion systems and interacting-fermion systems. We then focus on the classification of bosonic SPTs, which can be described by the group-cohomology theory (plus some exceptions of beyond-group-cohomology states). This will be the main focus of this appendix. At the end of this appendix, we will briefly review the structure of fermionic SPT classification.

A. Abstract description

We first introduce some abstract notations that apply to both bosonic, free-fermion and interacting fermion systems, and list some common properties shared by all these systems.

We use the notation $\Phi^d(G)$ to denote the set of d-dimensional SPT phases protected by the onsite symmetry group G. Since SPT phases are equivalence classes of states, we use $[\alpha]$ to denote them, and use $\sim$ to denote two identical phases. The phases in $\Phi^d(G)$ actually form an Abelian group: First, two phases $[\alpha]$ and $[\beta]$ can be added together. Denoted by $[\alpha]+[\beta]$, the sum represents the resulting phase obtained by stacking the two phases. The addition is commutative:

$$[\alpha] + [\beta] \sim [\beta] + [\alpha]. \tag{1}$$

Second, there is a trivial phase, which we denote by 0. Third, each phase $[\psi] \in \Phi^d(G)$ has an inverse, denoted by $-[\psi]$, such that stacking them together gives the trivial phase. In other words, phases in $\Phi^d(G)$ are "invertible" and have no topological ground-state degeneracy. This Abelian group can also be viewed as a $\mathbb{Z}$-module, or a linear space with integral coefficients.

In general, the classification $\Phi^d(G)$ at different dimensions can be computed using a generalized cohomology

theory. Intuitively, we may understand this mathematical structure using fixed-point wave functions (or partition functions) of the SPT phases. Here, fixed-point means that the wave functions or partition functions have zero correlation lengths, which make them suitable to describe gapped phases. In particular, we need to consider fixed-point wave functions in the geometry where a d-dimensional bulk terminates at a $(d-1)$-dimensional boundary. We need to consider not only the bulk wave functions, but also wave functions on the boundary of another bulk wave function. All such wave functions form a set, which we denote by $\Psi^d(G)$. Similar to the notation $\Phi^d(G)$, the superscript d denotes the dimensionality of the wave function and G denotes the onsite symmetry group.

The set $\Psi^d(G)$ has a similar structure as $\Phi^d(G)$, with a crucial difference: For fermionic systems, the stacking operation is not commutative, due to the statistical phases generated by exchanging fermionic operators. For this reason, we denote this stacking operation by $\boxplus$ instead.

Another important structure of $\Psi^d(G)$ is the coboundary operator d, which represents the relation between two wave functions in the bulk-boundary geometry. Given a d-dimensional bulk wave function $\alpha \in \Psi^d(G)$, not all $(d-1)$-dimensional wave functions can be realized on the boundary. If a wave function β can be put on the boundary of α, we denote this relation by

$$d\beta = \alpha. \tag{2}$$

Here, $d^{d-1} : \Psi^{d-1}(G) \to \Psi^d(G)$ is the coboundary map. The superscript $d-1$ indicates the domain of the map, and it is often omitted when it can be inferred from the context.

The two operations $\boxplus$ and d should satisfy some consistency conditions. First, we require that the coboundary operation commutes with stacking: $d\psi_1 \boxplus d\psi_2 = d(\psi_1 \boxplus \psi_2)$. In other words, $d : \Psi^p(G_\sigma) \to \Psi^{p+1}(G_\sigma)$ is a group homomorphism. This is because we can stack the bulk and boundary separately, and the resulting states still satisfies the bulk-boundary correspondence. Second, we require that the commutator between two states, $[\psi_1, \psi_2] = \psi_1 \boxplus \psi_2 - \psi_2 \boxplus \psi_1$, is a coboundary of another state in $(p-1)$ dimension. This is because although $\psi_1 \boxplus \psi_2$ and $\psi_2 \boxplus \psi_1$ may be different states, they should still belong to the same topological phase.

With these conditions satisfied, the SPT classification $\Phi^d(G)$ can be computed from $\Psi^d(G)$ using the coboundary maps. First, a standalone d-dimensional wave function must satisfy the cocycle equation $d\alpha = 0$, because it can be viewed as the boundary wave function of a $(d+1)$-dimensional vacuum. Second, if a $(d-1)$-dimensional wave function β can be put on the boundary of a d-dimensional wave function α: $\alpha = d\beta$, then α belongs to the trivial phase. This is because as a fixed-point wave function, β represents a gapped symmetric boundary state, and the existence of such a gapped symmetric boundary indicates that the bulk α belongs to the trivial SPT phase. Furthermore, two wave functions differ only by a coboundary $d\beta$ belong to the same SPT phase. One important property of d is that it satisfies the relation $d^2 = 0$, meaning that if $\alpha = d\beta$ is a bulk wave function, α is naturally a standalone d-dimensional wave function and satisfies $d\alpha = 0$. Using these results, we can compute the SPT classification using the cohomology group of the d map,

$$\Phi^d(G) = \frac{\ker d^d}{\operatorname{img} d^{d-1}}. \tag{3}$$

Here, the $\ker d$ in the numerator means that a wave function representing an SPT phase must satisfy the cocycle equation $d\alpha = 0$; the $\operatorname{img} d$ in the denominator means that two wave functions differ by a coboundary $d\beta$ represents the same phase.

B. Bosonic SPT and group cohomology

We first briefly review the definition of group cohomology for a discrete group G. The group cohomology can be computed using the homogeneous cochains, which are functions that map $(p+1)$ numbers of group elements to the coefficient ring $\mathrm{U}(1)_X$ (here, X denotes a generic group action, which will be explained below),

$$\alpha(g_0, \ldots, g_p) \in \mathrm{U}(1). \tag{4}$$

The word "homogeneous" means α satisfies the homogeneous condition,

$$\alpha(gg_0, \ldots, gg_p) = \rho_X(g)\alpha(g_0, \ldots, g_p), \tag{5}$$

where ρ_X denotes an arbitrary group action. In this work, we consider the following symmetry actions given by three $\mathbb{Z}_2$ gradings of the symmetry group G: First, we use $\rho_T(g) = \pm 1$ to denote whether $g \in G$ is a time-reversal operation: $\rho_T(g) = \pm 1$ if g is time-reversal even (odd), respectively. Second, we use $\rho_P(g) = \pm 1$ to denote whether g reverses the spatial orientation: a proper transformation, including a translation, a rotation and a skew rotation, has $\rho_P(g) = +1$; an improper transformation, including a mirror-reflection, a 3D inversion and a glide reflection, has $\rho_P(g) = -1$. Finally, we use ρ_{PT} to denote $\rho_{PT}(g) = \rho_P(g)\rho_T(g)$. Furthermore, we use $\mathrm{U}(1)_T$, $\mathrm{U}(1)_P$ and $\mathrm{U}(1)_{PT}$ to denote U(1) coefficient modules with the corresponding symmetry actions: $g \in G$ acts as a unitary (antiunitary) operator on coefficients in $\mathrm{U}(1)_X$ if $\rho_X(g) = \pm 1$, respectively.

An α satisfying Eqs. (4) and (5) is called a p-cochain. Notice that, in this paper, we treat U(1)coefficients as phase angles modulo 2π, instead of a phase factor. In this notation, the cochains form an additive group instead of a multiplicative group. All p cochains form an Abelian group (actually a $\mathbb{Z}$-linear space), denoted by $C^p[G, \mathrm{U}(1)_X]$.

We now define a coboundary map $d^p : C^p[G, \mathrm{U}(1)_X] \to C^{p+1}[G, \mathrm{U}(1)_X]$, with the following explicit formula,

$$d^p\alpha(g_0, \ldots, g_{p+1}) = \sum_{k=0}^{p+1}(-1)^k\alpha(g_0, \ldots, \hat{g}_k, \ldots, g_{p+1}), \tag{6}$$

where $\hat{g}_k$ means the element g_k is skipped. The superscript p in d^p denotes the cochain space it acts upon, and as in the main text, we often omit it when it can be determined from the context. The cochain map defined in Eq. (6) satisfies the condition

$$d^p d^{p-1} = 0. \tag{7}$$

Hence, linked by d^p, the cochain spaces $C^p[G, \mathrm{U}(1)_X]$ form a cochain complex,

$$\cdots \to C^{p-1}[G, \mathrm{U}(1)_X] \xrightarrow{d^{p-1}} C^p[G, \mathrm{U}(1)_X] \xrightarrow{d^p} C^{p+1}[G, \mathrm{U}(1)_X] \to \cdots . \tag{8}$$

The group cohomology of G is defined as the cohomology group of this cochain complex,

$$H^p[G, \mathrm{U}(1)_X] = \frac{\ker d^p}{\mathrm{img}\, d^{p-1}}. \tag{9}$$

The numerator $\ker d^p$ contains cochains satisfying the cocycle condition $d\omega = 0$, which are then called p-cocycles. The denominator implies that two cocycles differ by a coboundary $d\mu$ are considered as the same cohomology class. The cohomology group is then the quotient group of the cocycles over the coboundary equivalence.

For an onsite symmetry group G_0, the cohomology classes in $H^{d+1}[G_0, \mathrm{U}(1)_T]$ are in one-to-one correspondence to d-dimensional SPT states (there are a few beyond-group-cohomology SPT states that are not described by any cohomology classes.) This correspondence can be explicitly demonstrated using the group-cohomology models, which we briefly review below. These onsite SPT states are used in the main text as the building block of the topological crystals.

A group-cohomology model lives on a d-dimensional lattice, with a triangularization and a branching structure. The vertices of the lattice is organized into d-dimensional simplexes (triangles in 2D and tetrahedra in 3D). The branching structure is a set of orientations on all links between vertices, satisfying the condition that the links do not form any oriented loop. We also require that the orientations are invariant under the action of SG. It can be shown that one can always choose such a branching structure [1]. The Hilbert space of the model consists of a local Hilbert space on each vertex, spanned by basis vectors $|g_i\rangle$ corresponding to group elements $g_i \in G$. Using a cocycle $\alpha \in H^{d+1}[G, \mathrm{U}(1)_{PT}]$, one can construct the following fixed-point wave function on such a lattice,

$$|\Psi[\alpha]\rangle = \sum_{\{g_i\}} \prod_{\Delta i_1 \cdots i_d} \exp\{is(i_1, \ldots, i_d)\alpha(g_0, g_{i_1}, \ldots g_{i_d})\}\, |g_1 g_2 \cdots g_N\rangle, \tag{10}$$

where the product runs over all d-dimensional simplexes in the lattice, and $s(i_1, \ldots i_{d+1}) = \pm 1$ denotes whether the orientation of the simplex determined from the branching structure is the same or opposite to an overall orientation of the manifold. Recall that, in this paper, we treat U(1)coefficients as phase angles modulo 2π, instead of a phase factor. Hence, the cocycle α appears in the exponent in the above equation. In Eq. (10), g_0 is an arbitrary but fixed group element in G. One can shown that the wave function is independent of the choice of g_0, and it is invariant under the action of G on a d-dimensional manifold without a boundary, if we assume G acts in the following way: $g|g_i\rangle = |gg_{i'}\rangle$, where i' is the image of i under the action of g.

We now consider a more complicated geometry, where we simutaneously decorate cochains both inside a d-dimensional bulk and on its $(d-1)$-dimensional boundary. We start with the simplest case, where the bulk is one d-simplex, and the boundary has $(d+1)$ pieces of $(d-1)$-simplices. We decorate the bulk and the surface with wave functions constructed from a $(d+1)$-cochain α and a d-cochain β, respectively. Choosing the orientation of the bulk to be $+1$, the phase factor contributed from the bulk wave function is $\exp\{i\alpha(g_0, g_1, \ldots g_{d+1})\}$. It is straigntforward to check that the phase factors from all surface d-simplices is given by $d\beta$, as $\exp\{-id\beta(g_0, g_1, \ldots g_{d+1}) - i\beta(g_1, \ldots g_{d+1})\}$. Hence, if the two cochains satisfy

$$\alpha = d\beta, \tag{11}$$

the total phase factor is then $\exp\{-i\beta(g_1, \ldots g_{d+1})\}$, which is invariant under G_0-action, because β satisfies the homogeneous condition (5).

This can be generalized to an arbitrary d-dimensional bulk, which are divided into many d-simplices. For each simplex, the phase factors attached to the bulk and its surface is G_0-invariant. When multiplying the phase factors from different simplices together, the phase factors on the interior $(d-1)$-simplices cancel with each other, and we obtain a wave function that has phase factors from each d-simplex in the bulk, and each $(d-1)$-simplex on the boundary. Therefore, the wave function on the entire bulk and surface is also G_0-invariant, if the condition (11) is satisfied.

This setup can be used to demonstrate the bulk-boundary correspondence of SPT states. To realize gapped and symmetric states on both the bulk and the boundary, the cochains decorated to the bulk and the boundary must satisfy the condition in Eq. (11). If the bulk is empty, i. e. $\alpha = 0$, the decoration on the boundary must be a cocycle, since Eq. (11) then becomes the cocycle condition $d\beta = 0$. This is consistent with our understanding that a cocycle in $H^d[G, \mathrm{U}(1)_T]$ represents a $(d-1)$-dimensional symmetric gapped SPT state. If the bulk is not empty: $\alpha \neq 0$, the cochain β is then not a cocycle, as $d\beta = \alpha \neq 0$. This further implies that α is a trivial cocycle in $H^{d+1}[G, \mathrm{U}(1)_T]$, since $d\beta$ is a coboundary. On the other hand, if α is a nontrivial cocycle, then there is no solution of β that would satisfy Eq. (11). This means that the boundary of a nontrivial SPT state cannot be both gapped and symmetric. In this case, the nontrivial SPT state in the bulk induces a symmetry anomaly on the boundary, which obstructs the construction of a gapped symmetric wave function.

In summary, the group-cohomology models demonstrate that the cochains satisfied the general property we need in Sec. I A, and can be used to represent fixed-point wave functions of onsite-symmetry SPT states. In particular, we can take $\Psi^d(G) = C^{d+1}[G, \mathrm{U}(1)_T]$. Correspondingly, the SPT classification is given by $\Phi^d(G) = H^{d+1}[G, \mathrm{U}(1)_T]$.

In our paper, we need a more general type of cochain functions, which can be used to derive the same group-cohomology classes $H^p[G, \mathrm{U}(1)_X]$. Here, we consider two groups, H and G, where H is a subgroup of G: $H \subset G$. To compute the group cohomology of H, we consider cochains similar to Eq. (4), but the variables g_i take values in G instead of H. Different from regular cochains, we only require the G-valued cochains to be invariant under the action of H,

$$\alpha(hg_0, \dots, hg_p) = \rho_X(h)\alpha(g_0, \dots, g_p), \quad \forall h \in H. \tag{12}$$

Hence, we will refer to these cochains as the G-valued-H-invariant cochains. We denote the set of these cochains by $C^p_H[G, \mathrm{U}(1)_X]$, which is also a $\mathbb{Z}$-module. In general, α is not invariant under the action of $g \in G$, and the G-action on the G-valued-H-invariant cochains will induce a useful symmetry action that we will discuss in more details in Appendix II.

The coboundary maps d^p can be defined in a similary way as in Eq. (6). With these coboundary maps, the G-valued-H-invariant cochains also form a cochain complex. It can be shown using the fundamental lemma of homological algebra (see Lemma 7.4 of Ref. [2]] that the cohomology group of the G-valued cochain complex gives the same group cohomology of H.

In summary, one can choose to compute $H^p[H, \mathrm{U}(1)_X]$ using G-valued-H-invariant cochains where $H \subset G$. In practise, this seems to be a redundant way to compute $H^p[H, \mathrm{U}(1)_X]$, but as we see in the main text, this is a useful tool in the spectral-sequence computation, because it allows us to compute the symmetry actions on the cochains and the transfer map, which we will introduce in Appendices II and III, respectively. In the main text, we will use G-valued-G_σ-invariant cochains to represent states decorated on a cell σ, where G_σ is the local symmetry group of σ.

We can also use G-valued-H-invariant cocycles to construct group-cohomology models, which will be useful for Appendices II and III. Since the cochains are valued in G, we construct local Hilbert space as $|g\rangle$, where $g \in G$. We can then use a G-valued-H-invariant cocycle α to construct the wave function in Eq. (10). Since α is only invariant under H, the constructed wave function is also only invariant under H but not G. Hence, although we chose an enlarged local Hilbert space, the constructed wave functions still represent H-SPT states.

C. Interacting-fermion SPTs

In this section, we briefly review the classification of SPT phases in interacting-fermion systems, using the notations outlined in Sec. I A

First, the space of fixed-point wave functions $\Psi^d(G)$, as a set, is constructed by combining the following cochain spaces:

$$\Psi^d(G) = C^0(G_b, \Omega^{d+1}) \times C^1(G_b, \Omega^d) \times \cdots \times C^{d+1}(G_b, \Omega^0). \tag{13}$$

Here, G_b is the bosonic subgroup of the total symmetry group G: G always contains the fermion-parity symmetry group $\mathbb{Z}_2^f$ as a normal subgroup, and $G_b = G/\mathbb{Z}_2^f$ is the quotient group. Ω^d denotes the Abelian group of $(d+1)$-dimensional invertible topological phases. For fermionic systems, nontrivial entries of Ω^d are $\Omega^0 = \mathrm{U}(1)_T$, $\Omega^1 = \mathbb{Z}_2$, $\Omega^2 = \mathbb{Z}_2$ and $\Omega^3 = \mathbb{Z}_T$. Therefore, an element $\psi \in \Psi^d(G)$ is expressed as a tuple $(\psi^0, \psi^1, \dots, \psi^{d+1})$, where $\psi^p \in C^p(G, \Omega^{d-p+1})$.

The stacking operation, however, is not a simple componentwise addition of two tuples: $\psi_1 \boxplus \psi_2 \neq (\psi_1^{d+1} + \psi_2^{d+1}, \dots, \psi_1^0 + \psi_2^0)$. Instead, the result on the pth layer will be twisted by layers with smaller p. Such twisting has the following general form,

$$\begin{aligned}(\psi_1 \boxplus \psi_2)^p = {} & \psi_1^p + \psi_2^p \\ & + \mathcal{A}^{p,d-p+1}(\psi_1^0, \psi_2^0, \dots, \psi_1^{p-1}, \psi_2^{p-1}),\end{aligned} \tag{14}$$

where $\mathcal{A}^{p,d-p+1}$ is a generic function describing the twisting.

Next, we consider the coboundary map $d:\Psi^d(G)\to\Psi^{d+1}(G)$. Because the coboundary operation commutes with the stacking, it is suffice to consider the coboundary of a state that has only a single nonvanishing layer $\psi=(0,\dots,0,\psi^p,0,\dots,0)$. Generally, the coboundary of such a single-layer state has the following form,

$$(d\psi)^{p'}=\mathcal{D}^{p,d-p+1}_{p'-p}(\psi^p). \tag{15}$$

Here, $\mathcal{D}^{p,q}_r$ is a function from $C^p(G_b,\Omega^q)$ to $C^{p+r}(G_b,\Omega^{q-r+1})$, and it encodes the form of coboundaries. It is only nonvanishing for $r>0$, and for $r=1$, it is simply $\mathcal{D}^{p,q}_0(\psi^p)=d\psi^p$.

As an example, we list explicit forms of $\mathcal{A}^{pq}$ and $\mathcal{D}^{pq}_r$ for the case of $p+q=3$ (corresponding to $d=2$), and $G=G_b\times\mathbb{Z}_2^f$. Derivation of these results can be found in Refs. [3–5]. First, we list the nontrivial entries of $\mathcal{D}^{pq}_r$.

$$\mathcal{D}^{21}_2=\frac{1}{2}\psi^2\cup\psi^2+\frac{1}{2}\psi^2\cup_1 d\psi^2+\mathcal{O}'_4(d\psi^2); \tag{16}$$

$$\mathcal{D}^{12}_2=s\cup\psi^1\cup\psi^1, \tag{17}$$

where $O'_4(\psi^2)$ is a 4-cochain with the following form:

$$O'_4(\psi^2)(01234)=\frac{1}{2}d\psi^2(0124)d\psi^2(0234)\\ -\frac{1}{4}\{d\psi^2(0123)[1-d\psi^2(0124)]\mod 2\}. \tag{18}$$

Here, we follow Ref. [4] and use the simplified notation $\alpha(i_1\cdots i_m)=\alpha(g_{i_1},\dots,g_{i_m})$. s denotes the one-cocycle $s(g)=\pm1$ indicating whether g is a unitary or an antiunitary operation.

Next, we list the nontrivial entries of $\mathcal{A}^{pq}$. For simplicity, the parameters of the function are omitted, as they can be inferred from Eq. (14).

$$\mathcal{A}^{21}=\psi^1_1\cup\psi^1_2; \tag{19}$$

$$\mathcal{A}^{30}=\frac{1}{2}\psi^2_1\cup_1\psi^2_2+\frac{1}{2}(\psi^1_1\cup\psi^1_2)\cup_1(\psi^2_1+\psi^2_2)\\ +\frac{1}{2}\psi^1_1\cup(\psi^1_1\cup_1\psi^1_2)\cup\psi^1_2. \tag{20}$$

The above results of A^{pq} only apply to the limited cases when all symmetry operations in G_b are unitary.

II. SYMMETRY ACTIONS ON COCHAINS AND COCYCLES

In this appendix, we derive the mathematical form of symmetry actions relating cochains representing fixed-point wave functions of bosonic SPT states decorated on symmetry-related cells.

We consider two p-cells σ and σ', which are related by a symmetry operation $g_{\sigma\sigma'}\in G$ as $\sigma'=g_{\sigma\sigma'}\sigma$. The SPT states decorated on σ are classified by the cohomology group $H^{p+1}[G_\sigma,\mathrm{U}(1)_T]$. To study the G action on these cohomology classes, we represent these states using the group-cohomology models with G-valued-G_σ-invariant cocycles $C^{p+1}_{G_\sigma}[G,\mathrm{U}(1)_T]$ introduced in Appendix I. We consider a triangularization of σ with vertices $i_1,i_2,\dots,i_N$. On σ', we construct a symmetry-related triangularization, where each vertex i'_a is given by the image of i_a: $i'_a=gi_a$. On each vertex, the local Hilbert space is spanned by basis states $|g\rangle$, where $g\in G$. The symmetry group G acts on this Hilbert space in the following way: A local-symmetry operation $h\in G_\sigma$ acts locally on the Hilbert space on each vertex i on σ: $h|g_i\rangle_i=|hg_i\rangle_i$. A symmetry operation g in the left coset $g_{\sigma\sigma'}G_0$ maps the local Hilbert space on i to i', in addition to multiplying the onsite group element: $g|g_i\rangle_i=|gg_i\rangle_{i'}$. Symmetry elements not in the two cosets G_0 and $g_{\sigma\sigma'}G_0$ maps σ to other cells, and therefore are not considered here. As discussed before in Appendix I, the symmetries in G_σ requires that the cocycles decorated on σ must be invariant under G_σ, which then implies that they are classified by $H^{p+1}[G_\sigma,\mathrm{U}(1)_T]$.

The symmetries in $g_{\sigma\sigma'}G_0$, on the other hand, gives constraints between the decorations on σ and σ', which we now discuss. Assume that σ is decorated with the wave function $|\Psi[\alpha]\rangle$ as in Eq. (10), constructed with a G-valued-G_σ-invariant cochain α. The action $g_{\sigma\sigma'}$ maps the state $|g_{i_1}\rangle_{i_1}\otimes\cdots\otimes|g_{i_N}\rangle_{i_N}$ on σ to the state $|g_{\sigma\sigma'}g_{i_1}\rangle_{i'_1}\otimes\cdots\otimes|g_{\sigma\sigma'}g_{i_N}\rangle_{i'_N}$ on σ'. Therefore, it maps $|\Psi[\alpha]\rangle$ to the following wave function on σ',

$$|\Psi'\rangle=\sum_{\{g_{i_a}\}}\prod_{\Delta i_1\cdots i_p}\exp\left\{i\rho_T(g_{\sigma\sigma'})s(i_1,\dots,i_p)\alpha(g_0,g_{i_1},\dots,g_{i_p})\right\}|g_{\sigma\sigma'}g_{i_1}\rangle_{i'_1}\otimes\cdots\otimes|g_{\sigma\sigma'}g_{i_N}\rangle_{i'_N}\\ =\sum_{\{g_{i'_a}\}}\prod_{\Delta i'_1\cdots i'_p}\exp\left\{i\rho_T(g_{\sigma\sigma'})s(i'_1,\dots,i'_p)\alpha(g^{-1}_{\sigma\sigma'}g_0,g^{-1}_{\sigma\sigma'}g_{i'_1},\dots,g^{-1}_{\sigma\sigma'}g_{i'_p})\right\}|g_{i'_1}\rangle_{i'_1}\otimes\cdots\otimes|g_{i'_N}\rangle_{i'_N}. \tag{21}$$

This can be viewed as a wave function constructed from the following cochain α',

$$\alpha'(g_0,\dots,g_{p+1})=\rho_T(g_{\sigma\sigma'})\alpha(g^{-1}_{\sigma\sigma'}g_0,\dots g^{-1}_{\sigma\sigma'}g_N). \tag{22}$$

In other words, in order to get a G-symmetric decoration, if σ is decorated with a cochain α, σ' must be decorated with the cochain α' defined in Eq. (22).

This result motivates us to define a symmetry action on the G-valued cochains: for any $g \in G$ and a G-valued p-cochain α, we define $g \cdot \alpha$ as the following p-cochain:

$$(g \cdot \alpha)(g_0, \ldots, g_p) = \rho_T(g)\alpha(g^{-1}g_0, \ldots, g^{-1}g_p). \quad (23)$$

Using this notation, the constraint in Eq. (22) can be written in a concise way, as $\alpha' = g_{\sigma\sigma'} \cdot \alpha$. This group action is used in Secs. M-II A and M-IV C 1 in the main text, and the explicit form in Eq. (23) is used in Sec. M-IV C.

We end this appendix by listing some obvious but interesting properties of this symmetry action:

1. A cochain is H-invariant if for all $h \in H$, $h \cdot \alpha = \alpha$. This can be checked directly by comparing Eqs. (5) and (23).

2. If a cochain α is H-invariant, then the cochain $g \cdot \alpha$ is gHg^{-1}-invariant.

3. The group action commutes with the coboundary operator. Therefore, it maps cocycles to cocycles, and coboundaries to coboundaries.

4. Combining properties 2 and 3, we see that the group action $g \cdot \alpha$ induces an isomorphism between $H^p[H, \mathrm{U}(1)_T]$ and $H^p[gHg^{-1}, \mathrm{U}(1)_T]$. This isomorphism is used in Sec. M-II A. We notice that, in this way, this isomorphism is expressed using Eq. (23) in terms of G-valued cochains. Alternatively, it can also be expressed using H-valued and gHg^{-1}-valued cochains. Using this notation, we get the following isomorphism between $\alpha \in H^p[H, \mathrm{U}(1)_T]$ and $\alpha' \in H^p[gHg^{-1}, \mathrm{U}(1)_T]$:

$$\alpha(g_0, \ldots, g_p) = \alpha'(gg_0g^{-1}, \ldots, gg_pg^{-1}). \quad (24)$$

This looks different from the g-action in Eq. (23), but they actually gives the same isomorphism between the two cohomology groups, which can be calculated either using G-valued or H-valued cochains. The derivation of Eq. (24) can be found in Ref. [2].

Now let us explain why Eq. (24) gives same isomorphism between the two cohomology groups with Eq. (23). First, we show that the H-valued cochains and G-valued cochains form the same cohomology group. On one hand, by limiting the group elements of a G-valued cochain in H, we can map each G-valued cochain to a H-valued cochain. Apparently, (i) the resulted H-valued cohain is homogeneous under H and (ii) this map commutes with the boundary map d. On the other hand, we can map each H-valued cochain back to a G-valued cochain. We choose a representative for each right coset $H\backslash G$. For a given $g_i \in G$, we denote the representative of the belonging coset as $\bar{g}_i$. Then we define the map $\rho: G \to H$ as $\rho(g_i) = g_i\bar{g}_i^{-1}$. ρ introduces a map from H-valued cohains to G-valued cochains

$$(\rho\alpha)(g_0, \cdots, g_p) = \alpha(\rho(g_0), \cdots, \rho(g_p)). \quad (25)$$

It is direct to verify that (i) $\rho\alpha$ is homogeneous under H, and (ii) the boundary map d commutes with ρ. Therefore the cohomology groups formed by $\rho\alpha$ and α are isomorphic. We also introduce the map $\rho' : G \to gHg^{-1}$ as $\rho'(g_i) = g_i(g\bar{g}_i)^{-1}$, by choosing the representative of the right coset $gHg^{-1}\backslash G$ g_i belonging to as $g\bar{g}_i$. Similarly, ρ' introduces a map from gHg^{-1}-valued cochains, α', to G-valued cochains, $\rho'\alpha'$, and, the cohomology groups formed by α' and $\rho'\alpha'$ are isomorphic. Then it is direct to show that Eq. (24) implies

$$(\rho'\alpha')(g_0, \cdots, g_p) = (\rho\alpha)(g^{-1}g_0, \cdots, g^{-1}g_p), \quad (26)$$

which is equivalent with Eq. (23).

III. TRANSFER MAPS

In this appendix, we introduce the transfer map between the cohomology groups of two groups H and G, where H is a subgroup of G. This transfer map can be used to compute the d_1 map in Sec. M-II D. A detailed introduction to the transfer map can be found in Sec. 3.9 of Ref. [2].

To describe the transfer map, it is convenient to use the G-valued cochains. Consider a p-cochain α valued in G but invariant in H. Since it is not invariant under G, acting with an element $g \in G$ will generate a different cochain $g \cdot \alpha$, as defined in Appendix II. Hence, we use the following summation to obtain a G-invariant cochain, which we denote by $\overline{\mathrm{tr}}$,

$$\overline{\mathrm{tr}}_H^G \alpha = \sum_{g \in G/H} g \cdot \alpha. \quad (27)$$

It is easy to check that the $\overline{\mathrm{tr}}$ map commutes with the coboundary operator d. Therefore, it induces a homomorphism from $H^p[H, \mathrm{U}(1)_T]$ to $H^p[G, \mathrm{U}(1)_T]$, which is known as the transfer map and is denote by tr_H^G. The explicit form of tr_H^G is the following,

$$\mathrm{tr}_H^G[\alpha] = \sum_{g \in G/H} g \cdot [\alpha]. \quad (28)$$

Using the transfer map in (28), one can rewrite the d_1 map in Eq. (M-6) in the following compact form,

$$(d_1[\psi])|_\tau = \sum_{\sigma \in Y_p/G} \sum_{g \in G_\tau \backslash G/G_\sigma} \langle \partial\tau | g\sigma \rangle \, \mathrm{tr}_{gG_\sigma g^{-1}}^{G_\tau} \left(g \cdot [\psi|_\sigma] \right). \quad (29)$$

Similarly, the $\overline{tr}$ map in (27) can be used to express Eq. (M-16) in the following form,

$$(\partial\psi)|_\tau = \sum_{\sigma \in Y_p/G} \sum_{g \in G_\tau \backslash G/G_\sigma} \langle \partial\tau | g\sigma \rangle \, \overline{\mathrm{tr}}_{gG_\sigma g^{-1}}^{G_\tau} \left(g \cdot \psi|_\sigma \right). \quad (30)$$

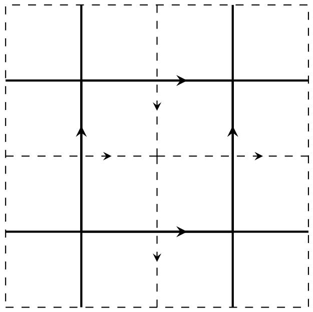

FIG. 1. The duality between the two G-complexes X and Y. The 1-cells in Y and X are represented by solid and dashed lines, respectively. The orientations on the 1-cells in X and Y are related by the right-hand rule.

IV. SPECTRAL SEQUENCE AND EQUIVARIANT COHOMOLOGY

In this appendix, we prove that for bosonic SPTs, the TCS classification we compute using the algorithm presented in this work, especially in Sec. M-IV C, exactly reproduces the result of Ref. [1]. We first briefly review the results of Ref. [1], which state that the classification is given by an equivariant cohomology. Following Chapter 7 of Ref. [2], we point out that the equivariant cohomology can be expressed as the cohomology group of a double cochain complex, which allows it to be computed by constructing a spectral sequence. We then construct the equivariant cohomology using the dual complex of Y as the topological space, and prove that the spectral sequence obtained in this way exactly agrees with the TCS computation presented in Sec. M-IV C. Mathematical details of our proof can be found in Ref. [2], especially Chapter 7.

Thorngren and Else [1] had shown that the classification of d-dimensional TCS protected by symmetry group G (with the exception of so-called "beyond-group-cohomology" states) can be computed using the following group-cohomology formula,

$$H^{d+1}[G, \mathrm{U}(1)_{PT}], \tag{31}$$

where $\mathrm{U}(1)_{PT}$ denotes the nontrivial action G has on the U(1) coefficients. Furthermore, this group cohomology can be computed as an equivariant cohomology, using a G-complex X [1],

$$H^{d+1}[G, \mathrm{U}(1)_{PT}] \simeq H_G^{d+1}[X, \mathrm{U}(1)_{PT}], \tag{32}$$

provided that X is topologically trivial, $X \simeq \mathrm{pt}$. A formal prove of this can be found in Proposition (7.3) of Ref. [2].

Here, we take X to be the dual complex of the G-complex Y we constructed in Sec. M-II A. As illustrated in Fig. 1, the p-cells of Y is in one-to-one correspondence to the $(d-p)$-cells of X, $Y_p \simeq X_{d-p}$, where d denotes the dimensionality of Y. We denote the cells in Y using greek letters without bars. In particular, for a p-cell $\bar{\sigma} \in Y_p$, we denote its dual $(d-p)$-cell as $\sigma \in X_{d-p}$.

As shown in Fig. 1, the orientation of cells on Y naturally induces an orientation of cells on X. For example, in 2D, the orientation on 1-cells in Y and dual 1-cells in X are related by the right-hand-rule. In particular, the relation between the orientations on X and Y depends on the orientation of the space $\mathbb{R}^n$. Hence, this relation is reversed under improper symmetry operations in SG, such as mirror reflections and glide symmetries. Recall that the orientation on Y is invariant under G_σ because the actions are pointwise. This implies that its dual $\bar{\sigma}$ transforms as the following under G_σ:

$$g\sigma = \rho_P(g)\sigma, \forall g \in G_\sigma. \tag{33}$$

Since X is the same as $\mathbb{R}^d$, which is contractible, Eq. (32) implies that the SPT classification can be computed using the equivariant cohomology $H_G^{d+1}[X, \mathrm{U}(1)_{PT}]$. Following Sec. 7.7 of Ref. [2], this equivariant cohomology can be expressed as the cohomology group of a double cochain complex, which can then be computed using a spectral sequence. As shown in Eq. (7.10) in Ref. [2], the E_1 page of this spectral sequence is given by

$$E_1^{pq} = \bigoplus_{\bar{\sigma} \in X_p/G} H^q[G_\sigma, \mathrm{U}(1)_T]. \tag{34}$$

Here, the coefficient module in the r.h.s becomes $\mathrm{U}(1)_T$ instead of $\mathrm{U}(1)_{PT}$ in Eq. (32), because the symmetry action in Eq. (33) cancels the P-action in $\mathrm{U}(1)_{PT}$. Using the duality $X_p \simeq Y_{d-p}$ and the fact that $H^q[G_\sigma, \mathrm{U}(1)_T]$ classifies the SPT phases $\Phi^{q-1}(G_\sigma)$, we see that E_1^{pq} is the same as the first-page module $E_{d-p,1}^{q-1}$ in Eq. (M-5). Moreover, the two coboundary operations in the double cochain complex is given by $d_1 = d$, the coboundary operation of the cochains, and the dual of the ∂ map defined in Eq. (M-16). Hence, more generally, the $E_{p,r}^q$ modules computed in Sec. M-IV C are the same as the r-th page modules $E_r^{d-p,q+1}$ in the spectral sequence (34). Therefore, using the result of Eq. (7.10) in Ref. [2], we can prove that the TCS classification computed in the main text exactly reproduces the group-cohomology classification in Ref. [1],

$$E_{p,r}^q \Rightarrow H^{q-p+d+1}[G, \mathrm{U}(1)_{PT}]. \tag{35}$$

V. PROOF OF TRIVIAL d_2 MAPS AND TRIVIAL GROUP EXTENSIONS WHEN $G = SG \times G_0$.

In this appendix, we prove that for 2D and 3D bosonic TCSs, the d_2 maps and group-extension problems are all trivial. Consequently, the simplified formula (M-11) in Sec. M-II produces the correct results for these cases.

We begin by pointing out some obvious cases where the two problems are trivial. First, recall that the d_2

map reduces the spatial dimension by 2: it maps a TCS pattern on p-cells to an anomaly pattern on $(p-2)$-cells. Therefore, the d_2 map can only be nontrivial if $p \geq 2$. Second, recall that the group-extension problem arises when n copies of a $d_b = p$ TCS pattern becomes trivial on p-cells but nontrivial on lower-dimensional cells. Since we don't view $d_b = 0$ TCSs as nontrivial states in (M-11), the group-extension problem may only exist for $p \geq 2$ as well.

Last but not least, we argue that these two problems do not arise for the top building-block dimension $d_b = d$. This is because by construction, the d-cells are the AUs of the space group (or the wallpaper group in 2D), where the local symmetry group is just $G_\sigma = G_0$. Therefore, the local SPT states we can decorate on the d-cells are simply d-dimensional SPT states protected by G_0 alone, which are described by $\Phi^d(G_0)$. If every AU is filled with the same element $[\alpha] \in \Phi^d(G_0)$, the entire system is nothing but the 3D strong SPT phase $[\alpha]$. When SG has no improper symmetries (like mirror reflections and glide planes), it is well known that α is always compatible with SG. When SG has improper symmetries, α is compatible with SG if and only if $[\alpha] \sim -[\alpha]$. In either cases, whether the assembly $[\alpha]$ satisfies the no-open-edge conditions can be figured out from d_1 map, and the higher-page maps and group-extensions are all trivial.

In summary, ruling out all these obvious cases, we see that the d_2 maps and the group-extension problems can only be nontrivial for the case of $p = 2$ in 3D.

We now focus on this case, and consider an assembly $[\psi]$ in $E^2_{2,\infty}$, representing 3D TCS states constructed by decorating 2D SPT states on 2-cells. The building blocks ψ_2 contains 3-cocycles $\psi_2|_\sigma$ attached to 2-cells. In order to compute the subleading terms, we need to express these 3-cocycles using G-valued cochains. The main result of this section is to show that, when G is a direct product of a 3D space group SG and an onsite symmetry group G_0, there is a canonical way to lift these 3-cocycles of G_σ to G-valued cochains, which happen to be not only invariant in G_σ, but also invariant in G. As a result, one can choose a vanishing subleading term $\psi_1 = 0$. This implies that the map d_2 vanishes. It also implies that the group extension of combining $E^2_{2,\infty}$ and $E^2_{2,\infty}$ is trivial.

Consider a 3-cocycle $\psi_2|\sigma$ decorated on a 2-cell σ. As discussed in Appendix I, it can be lifted to a G-valued cochain $\phi^*_\sigma\psi|_\sigma$, using a map $\phi_\sigma : G \to G_\sigma$,

$$\phi^*_\sigma\psi|_\sigma(g_0, g_1, g_2, g_3) = \psi_\sigma(\phi_\sigma(g_0), \phi_\sigma(g_1), \phi_\sigma(g_2), \phi_\sigma(g_3)). \tag{36}$$

In particular, we construct the following group homomorphism $\phi_\sigma : G \to G_\sigma$. Since $G = SG \times G_0$, we can also decompose G_σ as $G_\sigma = SG_\sigma \times G_0$, where $SG_\sigma = G_\sigma \cap SG$ denotes the subgroup of SG that keeps σ invariant. Notice that for two-cells in 3D, there are only two possibilities of SG_σ: $SG_\sigma = \mathbb{Z}_2$ if σ is a mirror plane, and $SG_\sigma = \mathbb{Z}_1$ (the trivial group) if it is not. An element $g \in G$ can be expressed as $g = g_1 g_2$, where $g_1 \in SG$ and $g_2 \in G_0$, respectively. For a mirror plane, we construct the following map,

$$\phi_\sigma(g) = \phi_\sigma(g_1 g_2) = \begin{cases} g_2, & \rho_M(g_1) = 1; \\ m_\sigma g_2, & \rho_M(g_1) = -1. \end{cases} \tag{37}$$

Here, m_σ denotes the nontrivial element of $SG_\sigma = \mathbb{Z}_2$. For a non-mirror plane, we simply define $\phi_\sigma = g_2$.

It is straightforward to check that the map ϕ_σ defined above is a group homomorphism, $\phi_\sigma(g_1 g_2) = \phi_\sigma(g_1)\phi_\sigma(g_2)$. Furthermore, it satisfies the condition that its restriction to G_σ is identity. Using this properties, we can show that $\phi^*_\sigma\psi|_\sigma$ is actually invariant under G actions:

$$\begin{aligned} \phi^*_\sigma\psi|_\sigma(gg_0, \ldots, gg_3) &= \psi|_\sigma(\phi_\sigma(g)\phi_\sigma(g_0), \ldots, \phi_\sigma(g)\phi_\sigma(g_3)) \\ &= \rho_T(\phi_\sigma(g))\psi|_\sigma(\phi_\sigma(g_0), \ldots, \phi_\sigma(g_3)). \end{aligned}$$

Therefore, this map defines a canonical way to embed $H^3[G_\sigma, \mathrm{U}(1)_T]$ into $H^3[G, \mathrm{U}(1)_T]$ as a subset. This means that when calculating the ∂ map in Eq. (M-16), one can ignore the g-action and just simply adds the cochains up. One can then explicitly check that, in such simple cases, if $[\partial\psi_2]$, there is a choice of $\partial\psi_2$ such that $\partial_1 = 0$. As a result, the subtleties of d_2 map and group-extension won't arise.

VI. 2-CELLS AND 1-CELLS IN SPACE GROUP $P\bar{4}n2$

Here we give the definitions of the 2-cells and 1-cells shown in Fig. M-5. For simplicity, we assume the lattice constant as 1. The 2-cells and 1-cells in one unit cell are defined by their vertices as

1. σ_1: $(00\frac{1}{4})$-$(00\frac{3}{4})$-$(\frac{1}{2}0\frac{3}{4})$-$(\frac{1}{2}0\frac{1}{4})$.
2. σ_2: $(0\frac{1}{2}\frac{1}{4})$-$(0\frac{1}{2}\frac{3}{4})$-$(\frac{1}{2}\frac{1}{2}\frac{3}{4})$-$(\frac{1}{2}\frac{1}{2}\frac{1}{4})$.
3. σ_3: $(00\frac{1}{4})$-$(0\frac{1}{2}\frac{1}{4})$-$(\frac{1}{2}\frac{1}{2}\frac{1}{4})$-$(\frac{1}{2}0\frac{1}{4})$.
4. σ_4: $(00\frac{3}{4})$-$(0\frac{1}{2}\frac{3}{4})$-$(\frac{1}{2}\frac{1}{2}\frac{3}{4})$-$(\frac{1}{2}0\frac{3}{4})$.
5. τ_1: $(0\frac{1}{2}\frac{1}{4})$-$(0\frac{1}{2}\frac{3}{4})$.
6. τ_2: $(00\frac{3}{4})$-$(\frac{1}{2}0\frac{3}{4})$.
7. τ_3: $(00\frac{1}{4})$-$(00\frac{3}{4})$.
8. τ_4: $(00\frac{3}{4})$-$(0\frac{1}{2}\frac{3}{4})$.
9. τ_5: $(\frac{1}{2}\frac{1}{2}\frac{1}{4})$-$(\frac{1}{2}\frac{1}{2}\frac{3}{4})$.

The equivalent cells can be generated by applying the space group operations on these cells. The generators of $P\bar{4}n2$ are

1. Translation t_x: $(x, y, z) \to (x+1, y, z)$.
2. Translation t_y: $(x, y, z) \to (x, y+1, z)$.
3. Translation t_z: $(x, y, z) \to (x, y, z+1)$.

4. Roto-reflection S_4: $(x, y, z) \to (-y, x, -z)$.

5. Glide mirror n: $(x, y, z) \to (-x + \frac{1}{2}, y + \frac{1}{2}, z + \frac{1}{2})$.

Since the building block used in Section M-IV F is $\mathbb{Z}_2$ 2D SPT state, we only need to match the boundary anomalies up to mod 2. Thus, in Eq. (M-52) to (M-55), we have omitted the boundary terms with even multiplicities.

VII. FULL CLASSIFICATION FOR BOSONIC TCS

TABLE I: Classifications of two-dimensional SPT phases.

G_0	$E_{2,\infty}^2$	$E_{1,\infty}^1$	$E_{0,\infty}^0$
Wallpaper group #1 $p1$			
$\varnothing$			
Z_2^T		$\mathbb{Z}_2^2$	
Z_2	$\mathbb{Z}_2$		$\mathbb{Z}_2$
$Z_2 \times Z_2^T$	$\mathbb{Z}_2^2$	$\mathbb{Z}_2^4$	$\mathbb{Z}_2$
Z_4	$\mathbb{Z}_4$		$\mathbb{Z}_4$
Z_6	$\mathbb{Z}_6$		$\mathbb{Z}_6$
$Z_2 \times Z_2$	$\mathbb{Z}_2^3$	$\mathbb{Z}_2^2$	$\mathbb{Z}_2^2$
$Z_4 \times Z_4$	$\mathbb{Z}_4^3$	$\mathbb{Z}_4^2$	$\mathbb{Z}_4^2$
$Z_6 \times Z_6$	$\mathbb{Z}_6^3$	$\mathbb{Z}_6^2$	$\mathbb{Z}_6^2$
$U(1)$	$\mathbb{Z}$		$\mathbb{Z}$
$SU(2)$	$\mathbb{Z}$		
$SO(3)$	$\mathbb{Z}$	$\mathbb{Z}_2^2$	
Wallpaper group #2 $p2$			
$\varnothing$			$\mathbb{Z}_2^4$
Z_2^T		$\mathbb{Z}_2^3$	$\mathbb{Z}_2^4$
Z_2	$\mathbb{Z}_2$		$\mathbb{Z}_2^8$
$Z_2 \times Z_2^T$	$\mathbb{Z}_2^2$	$\mathbb{Z}_2^6$	$\mathbb{Z}_2^8$
Z_4	$\mathbb{Z}_4$		$\mathbb{Z}_2^7 \times \mathbb{Z}_4$
Z_6	$\mathbb{Z}_6$		$\mathbb{Z}_2^7 \times \mathbb{Z}_6$
$Z_2 \times Z_2$	$\mathbb{Z}_2^3$	$\mathbb{Z}_2^3$	$\mathbb{Z}_2^{12}$
$Z_4 \times Z_4$	$\mathbb{Z}_4^3$	$\mathbb{Z}_2^3$	$\mathbb{Z}_2^{10} \times \mathbb{Z}_4^2$
$Z_6 \times Z_6$	$\mathbb{Z}_6^3$	$\mathbb{Z}_2^3$	$\mathbb{Z}_2^{10} \times \mathbb{Z}_6^2$
$U(1)$	$\mathbb{Z}$		$\mathbb{Z} \times \mathbb{Z}_2^7$
$SU(2)$	$\mathbb{Z}$		$\mathbb{Z}_2^4$
$SO(3)$	$\mathbb{Z}$	$\mathbb{Z}_2^3$	$\mathbb{Z}_2^4$
Wallpaper group #3 pm			
$\varnothing$			$\mathbb{Z}_2^2$
Z_2^T		$\mathbb{Z}_2^5$	$\mathbb{Z}_2^2$
Z_2	$\mathbb{Z}_2$	$\mathbb{Z}_2^2$	$\mathbb{Z}_2^4$
$Z_2 \times Z_2^T$	$\mathbb{Z}_2^2$	$\mathbb{Z}_2^{10}$	$\mathbb{Z}_2^4$
Z_4	$\mathbb{Z}_2$	$\mathbb{Z}_2^2$	$\mathbb{Z}_2^3 \times \mathbb{Z}_4$
Z_6	$\mathbb{Z}_2$	$\mathbb{Z}_2^2$	$\mathbb{Z}_2^3 \times \mathbb{Z}_6$
$Z_2 \times Z_2$	$\mathbb{Z}_2^3$	$\mathbb{Z}_2^7$	$\mathbb{Z}_2^6$
$Z_4 \times Z_4$	$\mathbb{Z}_2^3$	$\mathbb{Z}_2^6 \times \mathbb{Z}_4$	$\mathbb{Z}_2^4 \times \mathbb{Z}_4^2$
$Z_6 \times Z_6$	$\mathbb{Z}_2^3$	$\mathbb{Z}_2^6 \times \mathbb{Z}_6$	$\mathbb{Z}_2^4 \times \mathbb{Z}_6^2$
$U(1)$			$\mathbb{Z} \times \mathbb{Z}_2^3$
$SU(2)$			$\mathbb{Z}_2^2$
$SO(3)$		$\mathbb{Z}_2^3$	$\mathbb{Z}_2^2$
Wallpaper group #4 pg			
$\varnothing$			
Z_2^T		$\mathbb{Z}_2^2$	
Z_2	$\mathbb{Z}_2$		$\mathbb{Z}_2$
$Z_2 \times Z_2^T$	$\mathbb{Z}_2^2$	$\mathbb{Z}_2^4$	$\mathbb{Z}_2$
Z_4	$\mathbb{Z}_2$		$\mathbb{Z}_4$
Z_6	$\mathbb{Z}_2$		$\mathbb{Z}_6$
$Z_2 \times Z_2$	$\mathbb{Z}_2^3$	$\mathbb{Z}_2^2$	$\mathbb{Z}_2^2$
$Z_4 \times Z_4$	$\mathbb{Z}_2^3$	$\mathbb{Z}_2 \times \mathbb{Z}_4$	$\mathbb{Z}_4^2$
$Z_6 \times Z_6$	$\mathbb{Z}_2^3$	$\mathbb{Z}_2 \times \mathbb{Z}_6$	$\mathbb{Z}_6^2$

TABLE I (Continued.)

G_0	$E_{2,\infty}^2$	$E_{1,\infty}^1$	$E_{0,\infty}^0$
$U(1)$			$\mathbb{Z}$
$SU(2)$			
$SO(3)$		$\mathbb{Z}_2^2$	
Wallpaper group #5 cm			
$\varnothing$			$\mathbb{Z}_2$
Z_2^T		$\mathbb{Z}_2^3$	$\mathbb{Z}_2$
Z_2	$\mathbb{Z}_2$	$\mathbb{Z}_2$	$\mathbb{Z}_2^2$
$Z_2 \times Z_2^T$	$\mathbb{Z}_2^2$	$\mathbb{Z}_2^6$	$\mathbb{Z}_2^2$
Z_4	$\mathbb{Z}_2$	$\mathbb{Z}_2$	$\mathbb{Z}_2 \times \mathbb{Z}_4$
Z_6	$\mathbb{Z}_2$	$\mathbb{Z}_2$	$\mathbb{Z}_2 \times \mathbb{Z}_6$
$Z_2 \times Z_2$	$\mathbb{Z}_2^3$	$\mathbb{Z}_2^4$	$\mathbb{Z}_2^3$
$Z_4 \times Z_4$	$\mathbb{Z}_2^3$	$\mathbb{Z}_2^3 \times \mathbb{Z}_4$	$\mathbb{Z}_2 \times \mathbb{Z}_4^2$
$Z_6 \times Z_6$	$\mathbb{Z}_2^3$	$\mathbb{Z}_2^3 \times \mathbb{Z}_6$	$\mathbb{Z}_2 \times \mathbb{Z}_6^2$
$U(1)$			$\mathbb{Z} \times \mathbb{Z}_2$
$SU(2)$			$\mathbb{Z}_2$
$SO(3)$		$\mathbb{Z}_2^2$	$\mathbb{Z}_2$
Wallpaper group #6 $p2mm$			
$\varnothing$			$\mathbb{Z}_2^8$
Z_2^T		$\mathbb{Z}_2^8$	$\mathbb{Z}_2^8$
Z_2	$\mathbb{Z}_2$	$\mathbb{Z}_2^4$	$\mathbb{Z}_2^{12}$
$Z_2 \times Z_2^T$	$\mathbb{Z}_2^2$	$\mathbb{Z}_2^{16}$	$\mathbb{Z}_2^{12}$
Z_4	$\mathbb{Z}_2$	$\mathbb{Z}_2^4$	$\mathbb{Z}_2^{11} \times \mathbb{Z}_4$
Z_6	$\mathbb{Z}_2$	$\mathbb{Z}_2^4$	$\mathbb{Z}_2^{11} \times \mathbb{Z}_6$
$Z_2 \times Z_2$	$\mathbb{Z}_2^3$	$\mathbb{Z}_2^{12}$	$\mathbb{Z}_2^{16}$
$Z_4 \times Z_4$	$\mathbb{Z}_2^3$	$\mathbb{Z}_2^{12}$	$\mathbb{Z}_2^{14} \times \mathbb{Z}_4^2$
$Z_6 \times Z_6$	$\mathbb{Z}_2^3$	$\mathbb{Z}_2^{12}$	$\mathbb{Z}_2^{14} \times \mathbb{Z}_6^2$
$U(1)$			$\mathbb{Z} \times \mathbb{Z}_2^{11}$
$SU(2)$			$\mathbb{Z}_2^8$
$SO(3)$		$\mathbb{Z}_2^4$	$\mathbb{Z}_2^8$
Wallpaper group #7 $p2mg$			
$\varnothing$			$\mathbb{Z}_2^3$
Z_2^T		$\mathbb{Z}_2^4$	$\mathbb{Z}_2^3$
Z_2	$\mathbb{Z}_2$	$\mathbb{Z}_2$	$\mathbb{Z}_2^6$
$Z_2 \times Z_2^T$	$\mathbb{Z}_2^2$	$\mathbb{Z}_2^8$	$\mathbb{Z}_2^6$
Z_4	$\mathbb{Z}_2$	$\mathbb{Z}_2$	$\mathbb{Z}_2^5 \times \mathbb{Z}_4$
Z_6	$\mathbb{Z}_2$	$\mathbb{Z}_2$	$\mathbb{Z}_2^5 \times \mathbb{Z}_6$
$Z_2 \times Z_2$	$\mathbb{Z}_2^3$	$\mathbb{Z}_2^5$	$\mathbb{Z}_2^9$
$Z_4 \times Z_4$	$\mathbb{Z}_2^3$	$\mathbb{Z}_2^5$	$\mathbb{Z}_2^7 \times \mathbb{Z}_4^2$
$Z_6 \times Z_6$	$\mathbb{Z}_2^3$	$\mathbb{Z}_2^5$	$\mathbb{Z}_2^7 \times \mathbb{Z}_6^2$
$U(1)$			$\mathbb{Z} \times \mathbb{Z}_2^5$
$SU(2)$			$\mathbb{Z}_2^3$
$SO(3)$		$\mathbb{Z}_2^3$	$\mathbb{Z}_2^3$
Wallpaper group #8 $p2gg$			
$\varnothing$			$\mathbb{Z}_2^2$
Z_2^T		$\mathbb{Z}_2^2$	$\mathbb{Z}_2^2$
Z_2	$\mathbb{Z}_2$		$\mathbb{Z}_2^4$
$Z_2 \times Z_2^T$	$\mathbb{Z}_2^2$	$\mathbb{Z}_2^4$	$\mathbb{Z}_2^4$
Z_4	$\mathbb{Z}_2$		$\mathbb{Z}_2^3 \times \mathbb{Z}_4$
Z_6	$\mathbb{Z}_2$		$\mathbb{Z}_2^3 \times \mathbb{Z}_6$

TABLE I (Continued.)

G_0	$E_{2,\infty}^2$	$E_{1,\infty}^1$	$E_{0,\infty}^0$
$Z_2 \times Z_2$	$\mathbb{Z}_2^3$	$\mathbb{Z}_2^2$	$\mathbb{Z}_2^6$
$Z_4 \times Z_4$	$\mathbb{Z}_2^3$	$\mathbb{Z}_2^2$	$\mathbb{Z}_2^4 \times \mathbb{Z}_4^2$
$Z_6 \times Z_6$	$\mathbb{Z}_2^3$	$\mathbb{Z}_2^2$	$\mathbb{Z}_2^4 \times \mathbb{Z}_6^2$
$U(1)$			$\mathbb{Z} \times \mathbb{Z}_2^3$
$SU(2)$			$\mathbb{Z}_2^2$
$SO(3)$		$\mathbb{Z}_2^2$	$\mathbb{Z}_2^2$
Wallpaper group #9 $c2mm$			
$\varnothing$			$\mathbb{Z}_2^5$
Z_2^T		$\mathbb{Z}_2^5$	$\mathbb{Z}_2^5$
Z_2	$\mathbb{Z}_2$	$\mathbb{Z}_2^2$	$\mathbb{Z}_2^8$
$Z_2 \times Z_2^T$	$\mathbb{Z}_2^2$	$\mathbb{Z}_2^{10}$	$\mathbb{Z}_2^8$
Z_4	$\mathbb{Z}_2$	$\mathbb{Z}_2^2$	$\mathbb{Z}_2^7 \times \mathbb{Z}_4$
Z_6	$\mathbb{Z}_2$	$\mathbb{Z}_2^2$	$\mathbb{Z}_2^7 \times \mathbb{Z}_6$
$Z_2 \times Z_2$	$\mathbb{Z}_2^3$	$\mathbb{Z}_2^7$	$\mathbb{Z}_2^{11}$
$Z_4 \times Z_4$	$\mathbb{Z}_2^3$	$\mathbb{Z}_2^7$	$\mathbb{Z}_2^9 \times \mathbb{Z}_4^2$
$Z_6 \times Z_6$	$\mathbb{Z}_2^3$	$\mathbb{Z}_2^7$	$\mathbb{Z}_2^9 \times \mathbb{Z}_6^2$
$U(1)$			$\mathbb{Z} \times \mathbb{Z}_2^7$
$SU(2)$			$\mathbb{Z}_2^5$
$SO(3)$		$\mathbb{Z}_2^3$	$\mathbb{Z}_2^5$
Wallpaper group #10 $p4$			
$\varnothing$			$\mathbb{Z}_2 \times \mathbb{Z}_4^2$
Z_2^T		$\mathbb{Z}_2^2$	$\mathbb{Z}_2^3$
Z_2	$\mathbb{Z}_2$		$\mathbb{Z}_2^4 \times \mathbb{Z}_4^2$
$Z_2 \times Z_2^T$	$\mathbb{Z}_2^2$	$\mathbb{Z}_2^4$	$\mathbb{Z}_2^6$
Z_4	$\mathbb{Z}_4$		$\mathbb{Z}_2^2 \times \mathbb{Z}_4^4$
Z_6	$\mathbb{Z}_6$		$\mathbb{Z}_2^3 \times \mathbb{Z}_4^2 \times \mathbb{Z}_6$
$Z_2 \times Z_2$	$\mathbb{Z}_2^3$	$\mathbb{Z}_2^2$	$\mathbb{Z}_2^7 \times \mathbb{Z}_4^2$
$Z_4 \times Z_4$	$\mathbb{Z}_4^3$	$\mathbb{Z}_2 \times \mathbb{Z}_4$	$\mathbb{Z}_2^3 \times \mathbb{Z}_4^6$
$Z_6 \times Z_6$	$\mathbb{Z}_6^3$	$\mathbb{Z}_2^2$	$\mathbb{Z}_2^5 \times \mathbb{Z}_4^2 \times \mathbb{Z}_6^2$
$U(1)$	$\mathbb{Z}$		$\mathbb{Z} \times \mathbb{Z}_2^2 \times \mathbb{Z}_4^3$
$SU(2)$	$\mathbb{Z}$		$\mathbb{Z}_2 \times \mathbb{Z}_4^2$
$SO(3)$	$\mathbb{Z}$	$\mathbb{Z}_2^2$	$\mathbb{Z}_2 \times \mathbb{Z}_4^2$
Wallpaper group #11 $p4mm$			
$\varnothing$			$\mathbb{Z}_2^6$
Z_2^T		$\mathbb{Z}_2^6$	$\mathbb{Z}_2^6$
Z_2	$\mathbb{Z}_2$	$\mathbb{Z}_2^3$	$\mathbb{Z}_2^9$
$Z_2 \times Z_2^T$	$\mathbb{Z}_2^2$	$\mathbb{Z}_2^{12}$	$\mathbb{Z}_2^9$
Z_4	$\mathbb{Z}_2$	$\mathbb{Z}_2^3$	$\mathbb{Z}_2^7 \times \mathbb{Z}_4^2$
Z_6	$\mathbb{Z}_2$	$\mathbb{Z}_2^3$	$\mathbb{Z}_2^8 \times \mathbb{Z}_6$
$Z_2 \times Z_2$	$\mathbb{Z}_2^3$	$\mathbb{Z}_2^9$	$\mathbb{Z}_2^{12}$
$Z_4 \times Z_4$	$\mathbb{Z}_2^3$	$\mathbb{Z}_2^8 \times \mathbb{Z}_4$	$\mathbb{Z}_2^8 \times \mathbb{Z}_4^4$
$Z_6 \times Z_6$	$\mathbb{Z}_2^3$	$\mathbb{Z}_2^9$	$\mathbb{Z}_2^{10} \times \mathbb{Z}_6^2$
$U(1)$			$\mathbb{Z} \times \mathbb{Z}_2^7 \times \mathbb{Z}_4$
$SU(2)$			$\mathbb{Z}_2^6$
$SO(3)$		$\mathbb{Z}_2^3$	$\mathbb{Z}_2^6$
Wallpaper group #12 $p4gm$			
$\varnothing$			$\mathbb{Z}_2^2 \times \mathbb{Z}_4$
Z_2^T		$\mathbb{Z}_2^3$	$\mathbb{Z}_2^3$
Z_2	$\mathbb{Z}_2$	$\mathbb{Z}_2$	$\mathbb{Z}_2^4 \times \mathbb{Z}_4$

TABLE I (Continued.)

G_0	$E_{2,\infty}^2$	$E_{1,\infty}^1$	$E_{0,\infty}^0$
$Z_2 \times Z_2^T$	$\mathbb{Z}_2^2$	$\mathbb{Z}_2^6$	$\mathbb{Z}_2^5$
Z_4	$\mathbb{Z}_2$	$\mathbb{Z}_2$	$\mathbb{Z}_2^2 \times \mathbb{Z}_4^3$
Z_6	$\mathbb{Z}_2$	$\mathbb{Z}_2$	$\mathbb{Z}_2^3 \times \mathbb{Z}_4 \times \mathbb{Z}_6$
$Z_2 \times Z_2$	$\mathbb{Z}_2^3$	$\mathbb{Z}_2^4$	$\mathbb{Z}_2^6 \times \mathbb{Z}_4$
$Z_4 \times Z_4$	$\mathbb{Z}_2^3$	$\mathbb{Z}_2^3 \times \mathbb{Z}_4$	$\mathbb{Z}_2^2 \times \mathbb{Z}_4^5$
$Z_6 \times Z_6$	$\mathbb{Z}_2^3$	$\mathbb{Z}_2^4$	$\mathbb{Z}_2^4 \times \mathbb{Z}_4 \times \mathbb{Z}_6^2$
$U(1)$			$\mathbb{Z} \times \mathbb{Z}_2^2 \times \mathbb{Z}_4^2$
$SU(2)$			$\mathbb{Z}_2^2 \times \mathbb{Z}_4$
$SO(3)$		$\mathbb{Z}_2^2$	$\mathbb{Z}_2^2 \times \mathbb{Z}_4$
Wallpaper group #13 $p3$			
$\varnothing$			$\mathbb{Z}_3^3$
Z_2^T			
Z_2	$\mathbb{Z}_2$		$\mathbb{Z}_2 \times \mathbb{Z}_3^3$
$Z_2 \times Z_2^T$	$\mathbb{Z}_2^2$		$\mathbb{Z}_2$
Z_4	$\mathbb{Z}_4$		$\mathbb{Z}_3^3 \times \mathbb{Z}_4$
Z_6	$\mathbb{Z}_6$		$\mathbb{Z}_3^5 \times \mathbb{Z}_6$
$Z_2 \times Z_2$	$\mathbb{Z}_2^3$		$\mathbb{Z}_2^2 \times \mathbb{Z}_3^3$
$Z_4 \times Z_4$	$\mathbb{Z}_4^3$		$\mathbb{Z}_3^3 \times \mathbb{Z}_4^2$
$Z_6 \times Z_6$	$\mathbb{Z}_6^3$	$\mathbb{Z}_3^2$	$\mathbb{Z}_3^7 \times \mathbb{Z}_6^2$
$U(1)$	$\mathbb{Z}$		$\mathbb{Z} \times \mathbb{Z}_3^5$
$SU(2)$	$\mathbb{Z}$		$\mathbb{Z}_3^3$
$SO(3)$	$\mathbb{Z}$		$\mathbb{Z}_3^3$
Wallpaper group #14 $p3m1$			
$\varnothing$			$\mathbb{Z}_2$
Z_2^T		$\mathbb{Z}_2^2$	$\mathbb{Z}_2$
Z_2	$\mathbb{Z}_2$	$\mathbb{Z}_2$	$\mathbb{Z}_2^2$
$Z_2 \times Z_2^T$	$\mathbb{Z}_2^2$	$\mathbb{Z}_2^4$	$\mathbb{Z}_2^2$
Z_4	$\mathbb{Z}_2$	$\mathbb{Z}_2$	$\mathbb{Z}_2 \times \mathbb{Z}_4$
Z_6	$\mathbb{Z}_2$	$\mathbb{Z}_2$	$\mathbb{Z}_2 \times \mathbb{Z}_3^2 \times \mathbb{Z}_6$
$Z_2 \times Z_2$	$\mathbb{Z}_2^3$	$\mathbb{Z}_2^3$	$\mathbb{Z}_2^3$
$Z_4 \times Z_4$	$\mathbb{Z}_2^3$	$\mathbb{Z}_2^3$	$\mathbb{Z}_2 \times \mathbb{Z}_4^2$
$Z_6 \times Z_6$	$\mathbb{Z}_2^3$	$\mathbb{Z}_2^2 \times \mathbb{Z}_3 \times \mathbb{Z}_6$	$\mathbb{Z}_2 \times \mathbb{Z}_3^4 \times \mathbb{Z}_6^2$
$U(1)$			$\mathbb{Z} \times \mathbb{Z}_2 \times \mathbb{Z}_3^2$
$SU(2)$			$\mathbb{Z}_2$
$SO(3)$		$\mathbb{Z}_2$	$\mathbb{Z}_2$
Wallpaper group #15 $p31m$			
$\varnothing$			$\mathbb{Z}_2 \times \mathbb{Z}_3$
Z_2^T		$\mathbb{Z}_2^2$	$\mathbb{Z}_2$
Z_2	$\mathbb{Z}_2$	$\mathbb{Z}_2$	$\mathbb{Z}_2^2 \times \mathbb{Z}_3$
$Z_2 \times Z_2^T$	$\mathbb{Z}_2^2$	$\mathbb{Z}_2^4$	$\mathbb{Z}_2^2$
Z_4	$\mathbb{Z}_2$	$\mathbb{Z}_2$	$\mathbb{Z}_2 \times \mathbb{Z}_3 \times \mathbb{Z}_4$
Z_6	$\mathbb{Z}_2$	$\mathbb{Z}_2$	$\mathbb{Z}_2 \times \mathbb{Z}_3^2 \times \mathbb{Z}_6$
$Z_2 \times Z_2$	$\mathbb{Z}_2^3$	$\mathbb{Z}_2^3$	$\mathbb{Z}_2^3 \times \mathbb{Z}_3$
$Z_4 \times Z_4$	$\mathbb{Z}_2^3$	$\mathbb{Z}_2^3$	$\mathbb{Z}_2 \times \mathbb{Z}_3 \times \mathbb{Z}_4^2$
$Z_6 \times Z_6$	$\mathbb{Z}_2^3$	$\mathbb{Z}_2^2 \times \mathbb{Z}_6$	$\mathbb{Z}_2 \times \mathbb{Z}_3^3 \times \mathbb{Z}_6^2$
$U(1)$			$\mathbb{Z} \times \mathbb{Z}_2 \times \mathbb{Z}_3^2$
$SU(2)$			$\mathbb{Z}_2 \times \mathbb{Z}_3$
$SO(3)$		$\mathbb{Z}_2$	$\mathbb{Z}_2 \times \mathbb{Z}_3$
Wallpaper group #16 $p6$			

TABLE I (Continued.)

G_0	$E_{2,\infty}^2$	$E_{1,\infty}^1$	$E_{0,\infty}^0$
$\varnothing$			$\mathbb{Z}_2 \times \mathbb{Z}_3 \times \mathbb{Z}_6$
Z_2^T		$\mathbb{Z}_2$	$\mathbb{Z}_2^2$
Z_2	$\mathbb{Z}_2$		$\mathbb{Z}_2^3 \times \mathbb{Z}_3 \times \mathbb{Z}_6$
$Z_2 \times Z_2^T$	$\mathbb{Z}_2^2$	$\mathbb{Z}_2^2$	$\mathbb{Z}_2^4$
Z_4	$\mathbb{Z}_4$		$\mathbb{Z}_2^2 \times \mathbb{Z}_3 \times \mathbb{Z}_4 \times \mathbb{Z}_6$
Z_6	$\mathbb{Z}_6$		$\mathbb{Z}_2 \times \mathbb{Z}_3 \times \mathbb{Z}_6^3$
$Z_2 \times Z_2$	$\mathbb{Z}_2^3$	$\mathbb{Z}_2$	$\mathbb{Z}_2^5 \times \mathbb{Z}_3 \times \mathbb{Z}_6$
$Z_4 \times Z_4$	$\mathbb{Z}_4^3$	$\mathbb{Z}_2$	$\mathbb{Z}_2^3 \times \mathbb{Z}_3 \times \mathbb{Z}_4^2 \times \mathbb{Z}_6$
$Z_6 \times Z_6$	$\mathbb{Z}_6^3$	$\mathbb{Z}_6$	$\mathbb{Z}_2 \times \mathbb{Z}_3 \times \mathbb{Z}_6^5$
$U(1)$	$\mathbb{Z}$		$\mathbb{Z} \times \mathbb{Z}_2 \times \mathbb{Z}_3 \times \mathbb{Z}_6^2$
$SU(2)$	$\mathbb{Z}$		$\mathbb{Z}_2 \times \mathbb{Z}_3 \times \mathbb{Z}_6$
$SO(3)$	$\mathbb{Z}$	$\mathbb{Z}_2$	$\mathbb{Z}_2 \times \mathbb{Z}_3 \times \mathbb{Z}_6$
Wallpaper group #17 *p6mm*			
$\varnothing$			$\mathbb{Z}_2^4$
Z_2^T		$\mathbb{Z}_2^4$	$\mathbb{Z}_2^4$
Z_2	$\mathbb{Z}_2$	$\mathbb{Z}_2^2$	$\mathbb{Z}_2^6$
$Z_2 \times Z_2^T$	$\mathbb{Z}_2^2$	$\mathbb{Z}_2^8$	$\mathbb{Z}_2^6$
Z_4	$\mathbb{Z}_2$	$\mathbb{Z}_2^2$	$\mathbb{Z}_2^5 \times \mathbb{Z}_4$
Z_6	$\mathbb{Z}_2$	$\mathbb{Z}_2^2$	$\mathbb{Z}_2^4 \times \mathbb{Z}_6^2$
$Z_2 \times Z_2$	$\mathbb{Z}_2^3$	$\mathbb{Z}_2^6$	$\mathbb{Z}_2^8$
$Z_4 \times Z_4$	$\mathbb{Z}_2^3$	$\mathbb{Z}_2^6$	$\mathbb{Z}_2^6 \times \mathbb{Z}_4^2$
$Z_6 \times Z_6$	$\mathbb{Z}_2^3$	$\mathbb{Z}_2^5 \times \mathbb{Z}_6$	$\mathbb{Z}_2^4 \times \mathbb{Z}_6^4$
$U(1)$			$\mathbb{Z} \times \mathbb{Z}_2^4 \times \mathbb{Z}_6$
$SU(2)$			$\mathbb{Z}_2^4$
$SO(3)$		$\mathbb{Z}_2^2$	$\mathbb{Z}_2^4$

TABLE II: Classifications of three-dimensional SPT phases.

G_0	$E^3_{3,\infty}$	$E^2_{2,\infty}$	$E^1_{1,\infty}$	$E^0_{0,\infty}$
Space group #1 $P1$				
$\varnothing$				
Z_2^T	$\mathbb{Z}_2$		$\mathbb{Z}_2^3$	
Z_2		$\mathbb{Z}_2^3$		$\mathbb{Z}_2$
$Z_2 \times Z_2^T$	$\mathbb{Z}_2^3$	$\mathbb{Z}_2^6$	$\mathbb{Z}_2^6$	$\mathbb{Z}_2$
Z_4		$\mathbb{Z}_4^3$		$\mathbb{Z}_4$
Z_6		$\mathbb{Z}_6^3$		$\mathbb{Z}_6$
$Z_2 \times Z_2$	$\mathbb{Z}_2^2$	$\mathbb{Z}_2^9$	$\mathbb{Z}_2^3$	$\mathbb{Z}_2^2$
$Z_4 \times Z_4$	$\mathbb{Z}_4^2$	$\mathbb{Z}_4^9$	$\mathbb{Z}_4^3$	$\mathbb{Z}_4^2$
$Z_6 \times Z_6$	$\mathbb{Z}_6^2$	$\mathbb{Z}_6^9$	$\mathbb{Z}_6^3$	$\mathbb{Z}_6^2$
$U(1)$		$\mathbb{Z}^3$		$\mathbb{Z}$
$SU(2)$		$\mathbb{Z}^3$		
$SO(3)$		$\mathbb{Z}^3$	$\mathbb{Z}_2^3$	
Space group #2 $P\bar{1}$				
$\varnothing$				$\mathbb{Z}_2^8$
Z_2^T	$\mathbb{Z}_2$		$\mathbb{Z}_2^7$	$\mathbb{Z}_2^8$
Z_2		$\mathbb{Z}_2^4$		$\mathbb{Z}_2^{16}$
$Z_2 \times Z_2^T$	$\mathbb{Z}_2^3$	$\mathbb{Z}_2^8$	$\mathbb{Z}_2^{14}$	$\mathbb{Z}_2^{16}$
Z_4		$\mathbb{Z}_2 \times \mathbb{Z}_4^3$		$\mathbb{Z}_2^{15} \times \mathbb{Z}_4$
Z_6		$\mathbb{Z}_2 \times \mathbb{Z}_6^3$		$\mathbb{Z}_2^{15} \times \mathbb{Z}_6$
$Z_2 \times Z_2$	$\mathbb{Z}_2^2$	$\mathbb{Z}_2^{12}$	$\mathbb{Z}_2^7$	$\mathbb{Z}_2^{24}$
$Z_4 \times Z_4$	$\mathbb{Z}_2^2$	$\mathbb{Z}_2^3 \times \mathbb{Z}_4^9$	$\mathbb{Z}_2^7$	$\mathbb{Z}_2^{22} \times \mathbb{Z}_4^2$
$Z_6 \times Z_6$	$\mathbb{Z}_2^2$	$\mathbb{Z}_2^3 \times \mathbb{Z}_6^9$	$\mathbb{Z}_2^7$	$\mathbb{Z}_2^{22} \times \mathbb{Z}_6^2$
$U(1)$		$\mathbb{Z}^3 \times \mathbb{Z}_2$		$\mathbb{Z} \times \mathbb{Z}_2^{15}$
$SU(2)$		$\mathbb{Z}^3 \times \mathbb{Z}_2$		$\mathbb{Z}_2^8$
$SO(3)$		$\mathbb{Z}^3 \times \mathbb{Z}_2$	$\mathbb{Z}_2^7$	$\mathbb{Z}_2^8$
Space group #3 $P2$				
$\varnothing$				$\mathbb{Z}_2^4$
Z_2^T	$\mathbb{Z}_2$		$\mathbb{Z}_2^{11}$	$\mathbb{Z}_2^4$
Z_2		$\mathbb{Z}_2^4$	$\mathbb{Z}_2^4$	$\mathbb{Z}_2^8$
$Z_2 \times Z_2^T$	$\mathbb{Z}_2^3$	$\mathbb{Z}_2^8$	$\mathbb{Z}_2^{22}$	$\mathbb{Z}_2^8$
Z_4		$\mathbb{Z}_2^3 \times \mathbb{Z}_4$	$\mathbb{Z}_2^4$	$\mathbb{Z}_2^7 \times \mathbb{Z}_4$
Z_6		$\mathbb{Z}_2^3 \times \mathbb{Z}_6$	$\mathbb{Z}_2^4$	$\mathbb{Z}_2^7 \times \mathbb{Z}_6$
$Z_2 \times Z_2$	$\mathbb{Z}_2^2$	$\mathbb{Z}_2^{12}$	$\mathbb{Z}_2^{15}$	$\mathbb{Z}_2^{12}$
$Z_4 \times Z_4$	$\mathbb{Z}_4^2$	$\mathbb{Z}_2^9 \times \mathbb{Z}_4^3$	$\mathbb{Z}_2^{14} \times \mathbb{Z}_4$	$\mathbb{Z}_2^{10} \times \mathbb{Z}_4^2$
$Z_6 \times Z_6$	$\mathbb{Z}_6^2$	$\mathbb{Z}_2^9 \times \mathbb{Z}_6^3$	$\mathbb{Z}_2^{14} \times \mathbb{Z}_6$	$\mathbb{Z}_2^{10} \times \mathbb{Z}_6^2$
$U(1)$		$\mathbb{Z}$		$\mathbb{Z} \times \mathbb{Z}_2^7$
$SU(2)$		$\mathbb{Z}$		$\mathbb{Z}_2^4$
$SO(3)$		$\mathbb{Z}$	$\mathbb{Z}_2^7$	$\mathbb{Z}_2^4$
Space group #4 $P2_1$				
$\varnothing$				
Z_2^T	$\mathbb{Z}_2$		$\mathbb{Z}_2^3$	
Z_2		$\mathbb{Z}_2^3$		$\mathbb{Z}_2$
$Z_2 \times Z_2^T$	$\mathbb{Z}_2^3$	$\mathbb{Z}_2^6$	$\mathbb{Z}_2^6$	$\mathbb{Z}_2$
Z_4		$\mathbb{Z}_2^2 \times \mathbb{Z}_4$		$\mathbb{Z}_4$
Z_6		$\mathbb{Z}_2^2 \times \mathbb{Z}_6$		$\mathbb{Z}_6$
$Z_2 \times Z_2$	$\mathbb{Z}_2^2$	$\mathbb{Z}_2^9$	$\mathbb{Z}_2^3$	$\mathbb{Z}_2^2$
$Z_4 \times Z_4$	$\mathbb{Z}_4^2$	$\mathbb{Z}_2^6 \times \mathbb{Z}_4^3$	$\mathbb{Z}_2^2 \times \mathbb{Z}_4$	$\mathbb{Z}_4^2$
$Z_6 \times Z_6$	$\mathbb{Z}_6^2$	$\mathbb{Z}_2^6 \times \mathbb{Z}_6^3$	$\mathbb{Z}_2^2 \times \mathbb{Z}_6$	$\mathbb{Z}_6^2$

TABLE II (Continued.)

G_0	$E^3_{3,\infty}$	$E^2_{2,\infty}$	$E^1_{1,\infty}$	$E^0_{0,\infty}$
$U(1)$		$\mathbb{Z}$		$\mathbb{Z}$
$SU(2)$		$\mathbb{Z}$		
$SO(3)$		$\mathbb{Z}$	$\mathbb{Z}_2^3$	
Space group #5 $C2$				
$\varnothing$				$\mathbb{Z}_2^2$
Z_2^T	$\mathbb{Z}_2$		$\mathbb{Z}_2^6$	$\mathbb{Z}_2^2$
Z_2		$\mathbb{Z}_2^3$	$\mathbb{Z}_2^2$	$\mathbb{Z}_2^4$
$Z_2 \times Z_2^T$	$\mathbb{Z}_2^3$	$\mathbb{Z}_2^6$	$\mathbb{Z}_2^{12}$	$\mathbb{Z}_2^4$
Z_4		$\mathbb{Z}_2^2 \times \mathbb{Z}_4$	$\mathbb{Z}_2^2$	$\mathbb{Z}_2^3 \times \mathbb{Z}_4$
Z_6		$\mathbb{Z}_2^2 \times \mathbb{Z}_6$	$\mathbb{Z}_2^2$	$\mathbb{Z}_2^3 \times \mathbb{Z}_6$
$Z_2 \times Z_2$	$\mathbb{Z}_2^2$	$\mathbb{Z}_2^9$	$\mathbb{Z}_2^8$	$\mathbb{Z}_2^6$
$Z_4 \times Z_4$	$\mathbb{Z}_4^2$	$\mathbb{Z}_2^6 \times \mathbb{Z}_4^3$	$\mathbb{Z}_2^7 \times \mathbb{Z}_4$	$\mathbb{Z}_2^4 \times \mathbb{Z}_4^2$
$Z_6 \times Z_6$	$\mathbb{Z}_6^2$	$\mathbb{Z}_2^6 \times \mathbb{Z}_6^3$	$\mathbb{Z}_2^7 \times \mathbb{Z}_6$	$\mathbb{Z}_2^4 \times \mathbb{Z}_6^2$
$U(1)$		$\mathbb{Z}$		$\mathbb{Z} \times \mathbb{Z}_2^3$
$SU(2)$		$\mathbb{Z}$		$\mathbb{Z}_2^2$
$SO(3)$		$\mathbb{Z}$	$\mathbb{Z}_2^4$	$\mathbb{Z}_2^2$
Space group #6 Pm				
$\varnothing$		$\mathbb{Z}_2^2$		$\mathbb{Z}_2^2$
Z_2^T	$\mathbb{Z}_2$	$\mathbb{Z}_2^4$	$\mathbb{Z}_2^9$	$\mathbb{Z}_2^2$
Z_2		$\mathbb{Z}_2^8$	$\mathbb{Z}_2^4$	$\mathbb{Z}_2^4$
$Z_2 \times Z_2^T$	$\mathbb{Z}_2^3$	$\mathbb{Z}_2^{16}$	$\mathbb{Z}_2^{18}$	$\mathbb{Z}_2^4$
Z_4		$\mathbb{Z}_2^7 \times \mathbb{Z}_4$	$\mathbb{Z}_2^4$	$\mathbb{Z}_2^3 \times \mathbb{Z}_4$
Z_6		$\mathbb{Z}_2^7 \times \mathbb{Z}_6$	$\mathbb{Z}_2^4$	$\mathbb{Z}_2^3 \times \mathbb{Z}_6$
$Z_2 \times Z_2$	$\mathbb{Z}_2^2$	$\mathbb{Z}_2^{20}$	$\mathbb{Z}_2^{13}$	$\mathbb{Z}_2^6$
$Z_4 \times Z_4$	$\mathbb{Z}_2^2$	$\mathbb{Z}_2^{17} \times \mathbb{Z}_4^3$	$\mathbb{Z}_2^{11} \times \mathbb{Z}_4^2$	$\mathbb{Z}_2^4 \times \mathbb{Z}_4^2$
$Z_6 \times Z_6$	$\mathbb{Z}_2^2$	$\mathbb{Z}_2^{17} \times \mathbb{Z}_6^3$	$\mathbb{Z}_2^{11} \times \mathbb{Z}_6^2$	$\mathbb{Z}_2^4 \times \mathbb{Z}_6^2$
$U(1)$		$\mathbb{Z} \times \mathbb{Z}_2^5$		$\mathbb{Z} \times \mathbb{Z}_2^3$
$SU(2)$		$\mathbb{Z} \times \mathbb{Z}_2^3$		$\mathbb{Z}_2^2$
$SO(3)$		$\mathbb{Z} \times \mathbb{Z}_2^5$	$\mathbb{Z}_2^5$	$\mathbb{Z}_2^2$
Space group #7 Pc				
$\varnothing$				
Z_2^T	$\mathbb{Z}_2$		$\mathbb{Z}_2^3$	
Z_2		$\mathbb{Z}_2^3$		$\mathbb{Z}_2$
$Z_2 \times Z_2^T$	$\mathbb{Z}_2^3$	$\mathbb{Z}_2^6$	$\mathbb{Z}_2^6$	$\mathbb{Z}_2$
Z_4		$\mathbb{Z}_2^2 \times \mathbb{Z}_4$		$\mathbb{Z}_4$
Z_6		$\mathbb{Z}_2^2 \times \mathbb{Z}_6$		$\mathbb{Z}_6$
$Z_2 \times Z_2$	$\mathbb{Z}_2^2$	$\mathbb{Z}_2^9$	$\mathbb{Z}_2^3$	$\mathbb{Z}_2^2$
$Z_4 \times Z_4$	$\mathbb{Z}_2^2$	$\mathbb{Z}_2^6 \times \mathbb{Z}_4^3$	$\mathbb{Z}_2 \times \mathbb{Z}_4^2$	$\mathbb{Z}_4^2$
$Z_6 \times Z_6$	$\mathbb{Z}_2^2$	$\mathbb{Z}_2^6 \times \mathbb{Z}_6^3$	$\mathbb{Z}_2 \times \mathbb{Z}_6^2$	$\mathbb{Z}_6^2$
$U(1)$		$\mathbb{Z} \times \mathbb{Z}_2$		$\mathbb{Z}$
$SU(2)$		$\mathbb{Z} \times \mathbb{Z}_2$		
$SO(3)$		$\mathbb{Z} \times \mathbb{Z}_2$	$\mathbb{Z}_2^3$	
Space group #8 Cm				
$\varnothing$		$\mathbb{Z}_2$		$\mathbb{Z}_2$
Z_2^T	$\mathbb{Z}_2$	$\mathbb{Z}_2^2$	$\mathbb{Z}_2^5$	$\mathbb{Z}_2$
Z_2		$\mathbb{Z}_2^5$	$\mathbb{Z}_2^2$	$\mathbb{Z}_2^2$
$Z_2 \times Z_2^T$	$\mathbb{Z}_2^3$	$\mathbb{Z}_2^{10}$	$\mathbb{Z}_2^{10}$	$\mathbb{Z}_2^2$
Z_4		$\mathbb{Z}_2^4 \times \mathbb{Z}_4$	$\mathbb{Z}_2^2$	$\mathbb{Z}_2 \times \mathbb{Z}_4$
Z_6		$\mathbb{Z}_2^4 \times \mathbb{Z}_6$	$\mathbb{Z}_2^2$	$\mathbb{Z}_2 \times \mathbb{Z}_6$

TABLE II (Continued.)

G_0	$E_{3,\infty}^3$	$E_{2,\infty}^2$	$E_{1,\infty}^1$	$E_{0,\infty}^0$
$Z_2 \times Z_2$	$\mathbb{Z}_2^2$	$\mathbb{Z}_2^{13}$	$\mathbb{Z}_2^7$	$\mathbb{Z}_2^3$
$Z_4 \times Z_4$	$\mathbb{Z}_2^2$	$\mathbb{Z}_2^{10} \times \mathbb{Z}_4^3$	$\mathbb{Z}_2^5 \times \mathbb{Z}_4^2$	$\mathbb{Z}_2 \times \mathbb{Z}_4^2$
$Z_6 \times Z_6$	$\mathbb{Z}_2^2$	$\mathbb{Z}_2^{10} \times \mathbb{Z}_6^3$	$\mathbb{Z}_2^5 \times \mathbb{Z}_6^2$	$\mathbb{Z}_2 \times \mathbb{Z}_6^2$
$U(1)$		$\mathbb{Z} \times \mathbb{Z}_2^3$		$\mathbb{Z} \times \mathbb{Z}_2$
$SU(2)$		$\mathbb{Z} \times \mathbb{Z}_2^2$		$\mathbb{Z}_2$
$SO(3)$		$\mathbb{Z} \times \mathbb{Z}_2^3$	$\mathbb{Z}_2^3$	$\mathbb{Z}_2$
Space group #9 Cc				
$\varnothing$				
Z_2^T	$\mathbb{Z}_2$		$\mathbb{Z}_2^2$	
Z_2		$\mathbb{Z}_2^2$		$\mathbb{Z}_2$
$Z_2 \times Z_2^T$	$\mathbb{Z}_2^3$	$\mathbb{Z}_2^4$	$\mathbb{Z}_2^4$	$\mathbb{Z}_2$
Z_4		$\mathbb{Z}_2 \times \mathbb{Z}_4$		$\mathbb{Z}_4$
Z_6		$\mathbb{Z}_2 \times \mathbb{Z}_6$		$\mathbb{Z}_6$
$Z_2 \times Z_2$	$\mathbb{Z}_2^2$	$\mathbb{Z}_2^6$	$\mathbb{Z}_2^2$	$\mathbb{Z}_2^2$
$Z_4 \times Z_4$	$\mathbb{Z}_2^2$	$\mathbb{Z}_2^3 \times \mathbb{Z}_4^3$	$\mathbb{Z}_4^2$	$\mathbb{Z}_4^2$
$Z_6 \times Z_6$	$\mathbb{Z}_2^2$	$\mathbb{Z}_2^3 \times \mathbb{Z}_6^3$	$\mathbb{Z}_6^2$	$\mathbb{Z}_6^2$
$U(1)$		$\mathbb{Z} \times \mathbb{Z}_2$		$\mathbb{Z}$
$SU(2)$		$\mathbb{Z} \times \mathbb{Z}_2$		
$SO(3)$		$\mathbb{Z} \times \mathbb{Z}_2$	$\mathbb{Z}_2^2$	
Space group #10 $P2/m$				
$\varnothing$		$\mathbb{Z}_2^2$		$\mathbb{Z}_2^{16}$
Z_2^T	$\mathbb{Z}_2$	$\mathbb{Z}_2^4$	$\mathbb{Z}_2^{20}$	$\mathbb{Z}_2^{16}$
Z_2		$\mathbb{Z}_2^9$	$\mathbb{Z}_2^{10}$	$\mathbb{Z}_2^{24}$
$Z_2 \times Z_2^T$	$\mathbb{Z}_2^3$	$\mathbb{Z}_2^{18}$	$\mathbb{Z}_2^{40}$	$\mathbb{Z}_2^{24}$
Z_4		$\mathbb{Z}_2^8 \times \mathbb{Z}_4$	$\mathbb{Z}_2^{10}$	$\mathbb{Z}_2^{23} \times \mathbb{Z}_4$
Z_6		$\mathbb{Z}_2^8 \times \mathbb{Z}_6$	$\mathbb{Z}_2^{10}$	$\mathbb{Z}_2^{23} \times \mathbb{Z}_6$
$Z_2 \times Z_2$	$\mathbb{Z}_2^2$	$\mathbb{Z}_2^{23}$	$\mathbb{Z}_2^{30}$	$\mathbb{Z}_2^{32}$
$Z_4 \times Z_4$	$\mathbb{Z}_2^2$	$\mathbb{Z}_2^{20} \times \mathbb{Z}_4^3$	$\mathbb{Z}_2^{30}$	$\mathbb{Z}_2^{30} \times \mathbb{Z}_4^2$
$Z_6 \times Z_6$	$\mathbb{Z}_2^2$	$\mathbb{Z}_2^{20} \times \mathbb{Z}_6^3$	$\mathbb{Z}_2^{30}$	$\mathbb{Z}_2^{30} \times \mathbb{Z}_6^2$
$U(1)$		$\mathbb{Z} \times \mathbb{Z}_2^5$		$\mathbb{Z} \times \mathbb{Z}_2^{23}$
$SU(2)$		$\mathbb{Z} \times \mathbb{Z}_2^3$		$\mathbb{Z}_2^{16}$
$SO(3)$		$\mathbb{Z} \times \mathbb{Z}_2^5$	$\mathbb{Z}_2^{10}$	$\mathbb{Z}_2^{16}$
Space group #11 $P2_1/m$				
$\varnothing$		$\mathbb{Z}_2$		$\mathbb{Z}_2^5$
Z_2^T	$\mathbb{Z}_2$	$\mathbb{Z}_2^2$	$\mathbb{Z}_2^8$	$\mathbb{Z}_2^5$
Z_2		$\mathbb{Z}_2^6$	$\mathbb{Z}_2^2$	$\mathbb{Z}_2^{10}$
$Z_2 \times Z_2^T$	$\mathbb{Z}_2^3$	$\mathbb{Z}_2^{12}$	$\mathbb{Z}_2^{16}$	$\mathbb{Z}_2^{10}$
Z_4		$\mathbb{Z}_2^5 \times \mathbb{Z}_4$	$\mathbb{Z}_2^2$	$\mathbb{Z}_2^9 \times \mathbb{Z}_4$
Z_6		$\mathbb{Z}_2^5 \times \mathbb{Z}_6$	$\mathbb{Z}_2^2$	$\mathbb{Z}_2^9 \times \mathbb{Z}_6$
$Z_2 \times Z_2$	$\mathbb{Z}_2^2$	$\mathbb{Z}_2^{16}$	$\mathbb{Z}_2^{10}$	$\mathbb{Z}_2^{15}$
$Z_4 \times Z_4$	$\mathbb{Z}_2^2$	$\mathbb{Z}_2^{13} \times \mathbb{Z}_4^3$	$\mathbb{Z}_2^{10}$	$\mathbb{Z}_2^{13} \times \mathbb{Z}_4^2$
$Z_6 \times Z_6$	$\mathbb{Z}_2^2$	$\mathbb{Z}_2^{13} \times \mathbb{Z}_6^3$	$\mathbb{Z}_2^{10}$	$\mathbb{Z}_2^{13} \times \mathbb{Z}_6^2$
$U(1)$		$\mathbb{Z} \times \mathbb{Z}_2^3$		$\mathbb{Z} \times \mathbb{Z}_2^9$
$SU(2)$		$\mathbb{Z} \times \mathbb{Z}_2^2$		$\mathbb{Z}_2^5$
$SO(3)$		$\mathbb{Z} \times \mathbb{Z}_2^3$	$\mathbb{Z}_2^6$	$\mathbb{Z}_2^5$
Space group #12 $C2/m$				
$\varnothing$		$\mathbb{Z}_2$		$\mathbb{Z}_2^{10}$
Z_2^T	$\mathbb{Z}_2$	$\mathbb{Z}_2^2$	$\mathbb{Z}_2^{12}$	$\mathbb{Z}_2^{10}$
Z_2		$\mathbb{Z}_2^6$	$\mathbb{Z}_2^5$	$\mathbb{Z}_2^{16}$

TABLE II (Continued.)

G_0	$E_{3,\infty}^3$	$E_{2,\infty}^2$	$E_{1,\infty}^1$	$E_{0,\infty}^0$
$Z_2 \times Z_2^T$	$\mathbb{Z}_2^3$	$\mathbb{Z}_2^{12}$	$\mathbb{Z}_2^{24}$	$\mathbb{Z}_2^{16}$
Z_4		$\mathbb{Z}_2^5 \times \mathbb{Z}_4$	$\mathbb{Z}_2^5$	$\mathbb{Z}_2^{15} \times \mathbb{Z}_4$
Z_6		$\mathbb{Z}_2^5 \times \mathbb{Z}_6$	$\mathbb{Z}_2^5$	$\mathbb{Z}_2^{15} \times \mathbb{Z}_6$
$Z_2 \times Z_2$	$\mathbb{Z}_2^2$	$\mathbb{Z}_2^{16}$	$\mathbb{Z}_2^{17}$	$\mathbb{Z}_2^{22}$
$Z_4 \times Z_4$	$\mathbb{Z}_2^2$	$\mathbb{Z}_2^{13} \times \mathbb{Z}_4^3$	$\mathbb{Z}_2^{17}$	$\mathbb{Z}_2^{20} \times \mathbb{Z}_4^2$
$Z_6 \times Z_6$	$\mathbb{Z}_2^2$	$\mathbb{Z}_2^{13} \times \mathbb{Z}_6^3$	$\mathbb{Z}_2^{17}$	$\mathbb{Z}_2^{20} \times \mathbb{Z}_6^2$
$U(1)$		$\mathbb{Z} \times \mathbb{Z}_2^3$		$\mathbb{Z} \times \mathbb{Z}_2^{15}$
$SU(2)$		$\mathbb{Z} \times \mathbb{Z}_2^2$		$\mathbb{Z}_2^{10}$
$SO(3)$		$\mathbb{Z} \times \mathbb{Z}_2^3$	$\mathbb{Z}_2^7$	$\mathbb{Z}_2^{10}$
Space group #13 $P2/c$				
$\varnothing$				$\mathbb{Z}_2^6$
Z_2^T	$\mathbb{Z}_2$		$\mathbb{Z}_2^9$	$\mathbb{Z}_2^6$
Z_2		$\mathbb{Z}_2^4$	$\mathbb{Z}_2^2$	$\mathbb{Z}_2^{12}$
$Z_2 \times Z_2^T$	$\mathbb{Z}_2^3$	$\mathbb{Z}_2^8$	$\mathbb{Z}_2^{18}$	$\mathbb{Z}_2^{12}$
Z_4		$\mathbb{Z}_2^3 \times \mathbb{Z}_4$	$\mathbb{Z}_2^2$	$\mathbb{Z}_2^{11} \times \mathbb{Z}_4$
Z_6		$\mathbb{Z}_2^3 \times \mathbb{Z}_6$	$\mathbb{Z}_2^2$	$\mathbb{Z}_2^{11} \times \mathbb{Z}_6$
$Z_2 \times Z_2$	$\mathbb{Z}_2^2$	$\mathbb{Z}_2^{12}$	$\mathbb{Z}_2^{11}$	$\mathbb{Z}_2^{18}$
$Z_4 \times Z_4$	$\mathbb{Z}_2^2$	$\mathbb{Z}_2^9 \times \mathbb{Z}_4^3$	$\mathbb{Z}_2^{11}$	$\mathbb{Z}_2^{16} \times \mathbb{Z}_4^2$
$Z_6 \times Z_6$	$\mathbb{Z}_2^2$	$\mathbb{Z}_2^9 \times \mathbb{Z}_6^3$	$\mathbb{Z}_2^{11}$	$\mathbb{Z}_2^{16} \times \mathbb{Z}_6^2$
$U(1)$		$\mathbb{Z} \times \mathbb{Z}_2$		$\mathbb{Z} \times \mathbb{Z}_2^{11}$
$SU(2)$		$\mathbb{Z} \times \mathbb{Z}_2$		$\mathbb{Z}_2^6$
$SO(3)$		$\mathbb{Z} \times \mathbb{Z}_2$	$\mathbb{Z}_2^7$	$\mathbb{Z}_2^6$
Space group #14 $P2_1/c$				
$\varnothing$				$\mathbb{Z}_2^4$
Z_2^T	$\mathbb{Z}_2$		$\mathbb{Z}_2^4$	$\mathbb{Z}_2^4$
Z_2		$\mathbb{Z}_2^3$		$\mathbb{Z}_2^8$
$Z_2 \times Z_2^T$	$\mathbb{Z}_2^3$	$\mathbb{Z}_2^6$	$\mathbb{Z}_2^8$	$\mathbb{Z}_2^8$
Z_4		$\mathbb{Z}_2^2 \times \mathbb{Z}_4$		$\mathbb{Z}_2^7 \times \mathbb{Z}_4$
Z_6		$\mathbb{Z}_2^2 \times \mathbb{Z}_6$		$\mathbb{Z}_2^7 \times \mathbb{Z}_6$
$Z_2 \times Z_2$	$\mathbb{Z}_2^2$	$\mathbb{Z}_2^9$	$\mathbb{Z}_2^4$	$\mathbb{Z}_2^{12}$
$Z_4 \times Z_4$	$\mathbb{Z}_2^2$	$\mathbb{Z}_2^6 \times \mathbb{Z}_4^3$	$\mathbb{Z}_2^4$	$\mathbb{Z}_2^{10} \times \mathbb{Z}_4^2$
$Z_6 \times Z_6$	$\mathbb{Z}_2^2$	$\mathbb{Z}_2^6 \times \mathbb{Z}_6^3$	$\mathbb{Z}_2^4$	$\mathbb{Z}_2^{10} \times \mathbb{Z}_6^2$
$U(1)$		$\mathbb{Z} \times \mathbb{Z}_2$		$\mathbb{Z} \times \mathbb{Z}_2^7$
$SU(2)$		$\mathbb{Z} \times \mathbb{Z}_2$		$\mathbb{Z}_2^4$
$SO(3)$		$\mathbb{Z} \times \mathbb{Z}_2$	$\mathbb{Z}_2^4$	$\mathbb{Z}_2^4$
Space group #15 $C2/c$				
$\varnothing$				$\mathbb{Z}_2^5$
Z_2^T	$\mathbb{Z}_2$		$\mathbb{Z}_2^6$	$\mathbb{Z}_2^5$
Z_2		$\mathbb{Z}_2^3$	$\mathbb{Z}_2$	$\mathbb{Z}_2^{10}$
$Z_2 \times Z_2^T$	$\mathbb{Z}_2^3$	$\mathbb{Z}_2^6$	$\mathbb{Z}_2^{12}$	$\mathbb{Z}_2^{10}$
Z_4		$\mathbb{Z}_2^2 \times \mathbb{Z}_4$	$\mathbb{Z}_2$	$\mathbb{Z}_2^9 \times \mathbb{Z}_4$
Z_6		$\mathbb{Z}_2^2 \times \mathbb{Z}_6$	$\mathbb{Z}_2$	$\mathbb{Z}_2^9 \times \mathbb{Z}_6$
$Z_2 \times Z_2$	$\mathbb{Z}_2^2$	$\mathbb{Z}_2^9$	$\mathbb{Z}_2^7$	$\mathbb{Z}_2^{15}$
$Z_4 \times Z_4$	$\mathbb{Z}_2^2$	$\mathbb{Z}_2^6 \times \mathbb{Z}_4^3$	$\mathbb{Z}_2^7$	$\mathbb{Z}_2^{13} \times \mathbb{Z}_4^2$
$Z_6 \times Z_6$	$\mathbb{Z}_2^2$	$\mathbb{Z}_2^6 \times \mathbb{Z}_6^3$	$\mathbb{Z}_2^7$	$\mathbb{Z}_2^{13} \times \mathbb{Z}_6^2$
$U(1)$		$\mathbb{Z} \times \mathbb{Z}_2$		$\mathbb{Z} \times \mathbb{Z}_2^9$
$SU(2)$		$\mathbb{Z} \times \mathbb{Z}_2$		$\mathbb{Z}_2^5$
$SO(3)$		$\mathbb{Z} \times \mathbb{Z}_2$	$\mathbb{Z}_2^5$	$\mathbb{Z}_2^5$
Space group #16 $P222$				

TABLE II (Continued.)

G_0	$E_{3,\infty}^3$	$E_{2,\infty}^2$	$E_{1,\infty}^1$	$E_{0,\infty}^0$
$\varnothing$				$\mathbb{Z}_2^{16}$
Z_2^T	$\mathbb{Z}_2$		$\mathbb{Z}_2^{24}$	$\mathbb{Z}_2^{16}$
Z_2		$\mathbb{Z}_2^5$	$\mathbb{Z}_2^{12}$	$\mathbb{Z}_2^{24}$
$Z_2 \times Z_2^T$	$\mathbb{Z}_2^3$	$\mathbb{Z}_2^{10}$	$\mathbb{Z}_2^{48}$	$\mathbb{Z}_2^{24}$
Z_4		$\mathbb{Z}_2^5$	$\mathbb{Z}_2^{12}$	$\mathbb{Z}_2^{23} \times \mathbb{Z}_4$
Z_6		$\mathbb{Z}_2^5$	$\mathbb{Z}_2^{12}$	$\mathbb{Z}_2^{23} \times \mathbb{Z}_6$
$Z_2 \times Z_2$	$\mathbb{Z}_2^2$	$\mathbb{Z}_2^{15}$	$\mathbb{Z}_2^{36}$	$\mathbb{Z}_2^{32}$
$Z_4 \times Z_4$	$\mathbb{Z}_4^2$	$\mathbb{Z}_2^{15}$	$\mathbb{Z}_2^{36}$	$\mathbb{Z}_2^{30} \times \mathbb{Z}_4^2$
$Z_6 \times Z_6$	$\mathbb{Z}_6^2$	$\mathbb{Z}_2^{15}$	$\mathbb{Z}_2^{36}$	$\mathbb{Z}_2^{30} \times \mathbb{Z}_6^2$
$U(1)$				$\mathbb{Z} \times \mathbb{Z}_2^{23}$
$SU(2)$				$\mathbb{Z}_2^{16}$
$SO(3)$			$\mathbb{Z}_2^{12}$	$\mathbb{Z}_2^{16}$
Space group #17 $P222_1$				
$\varnothing$				$\mathbb{Z}_2^4$
Z_2^T	$\mathbb{Z}_2$		$\mathbb{Z}_2^{11}$	$\mathbb{Z}_2^4$
Z_2		$\mathbb{Z}_2^4$	$\mathbb{Z}_2^4$	$\mathbb{Z}_2^8$
$Z_2 \times Z_2^T$	$\mathbb{Z}_2^3$	$\mathbb{Z}_2^8$	$\mathbb{Z}_2^{22}$	$\mathbb{Z}_2^8$
Z_4		$\mathbb{Z}_2^4$	$\mathbb{Z}_2^4$	$\mathbb{Z}_2^7 \times \mathbb{Z}_4$
Z_6		$\mathbb{Z}_2^4$	$\mathbb{Z}_2^4$	$\mathbb{Z}_2^7 \times \mathbb{Z}_6$
$Z_2 \times Z_2$	$\mathbb{Z}_2^2$	$\mathbb{Z}_2^{12}$	$\mathbb{Z}_2^{15}$	$\mathbb{Z}_2^{12}$
$Z_4 \times Z_4$	$\mathbb{Z}_4^2$	$\mathbb{Z}_2^{12}$	$\mathbb{Z}_2^{15}$	$\mathbb{Z}_2^{10} \times \mathbb{Z}_4^2$
$Z_6 \times Z_6$	$\mathbb{Z}_6^2$	$\mathbb{Z}_2^{12}$	$\mathbb{Z}_2^{15}$	$\mathbb{Z}_2^{10} \times \mathbb{Z}_6^2$
$U(1)$				$\mathbb{Z} \times \mathbb{Z}_2^7$
$SU(2)$				$\mathbb{Z}_2^4$
$SO(3)$			$\mathbb{Z}_2^7$	$\mathbb{Z}_2^4$
Space group #18 $P2_12_12$				
$\varnothing$				$\mathbb{Z}_2^2$
Z_2^T	$\mathbb{Z}_2$		$\mathbb{Z}_2^6$	$\mathbb{Z}_2^2$
Z_2		$\mathbb{Z}_2^3$	$\mathbb{Z}_2^2$	$\mathbb{Z}_2^4$
$Z_2 \times Z_2^T$	$\mathbb{Z}_2^3$	$\mathbb{Z}_2^6$	$\mathbb{Z}_2^{12}$	$\mathbb{Z}_2^4$
Z_4		$\mathbb{Z}_2^2 \times \mathbb{Z}_4$	$\mathbb{Z}_2^2$	$\mathbb{Z}_2^3 \times \mathbb{Z}_4$
Z_6		$\mathbb{Z}_2^3$	$\mathbb{Z}_2^2$	$\mathbb{Z}_2^3 \times \mathbb{Z}_6$
$Z_2 \times Z_2$	$\mathbb{Z}_2^2$	$\mathbb{Z}_2^9$	$\mathbb{Z}_2^8$	$\mathbb{Z}_2^6$
$Z_4 \times Z_4$	$\mathbb{Z}_4^2$	$\mathbb{Z}_2^6 \times \mathbb{Z}_4^3$	$\mathbb{Z}_2^7 \times \mathbb{Z}_4$	$\mathbb{Z}_2^4 \times \mathbb{Z}_4^2$
$Z_6 \times Z_6$	$\mathbb{Z}_6^2$	$\mathbb{Z}_2^9$	$\mathbb{Z}_2^8$	$\mathbb{Z}_2^4 \times \mathbb{Z}_6^2$
$U(1)$				$\mathbb{Z} \times \mathbb{Z}_2^3$
$SU(2)$				$\mathbb{Z}_2^2$
$SO(3)$			$\mathbb{Z}_2^4$	$\mathbb{Z}_2^2$
Space group #19 $P2_12_12_1$				
$\varnothing$				
Z_2^T	$\mathbb{Z}_2$		$\mathbb{Z}_2^2$	
Z_2		$\mathbb{Z}_2^2$		$\mathbb{Z}_2$
$Z_2 \times Z_2^T$	$\mathbb{Z}_2^3$	$\mathbb{Z}_2^4$	$\mathbb{Z}_2^4$	$\mathbb{Z}_2$
Z_4		$\mathbb{Z}_4^2$		$\mathbb{Z}_4$
Z_6		$\mathbb{Z}_2^2$		$\mathbb{Z}_6$
$Z_2 \times Z_2$	$\mathbb{Z}_2^2$	$\mathbb{Z}_2^6$	$\mathbb{Z}_2^2$	$\mathbb{Z}_2^2$
$Z_4 \times Z_4$	$\mathbb{Z}_4^2$	$\mathbb{Z}_4^6$	$\mathbb{Z}_4^2$	$\mathbb{Z}_4^2$
$Z_6 \times Z_6$	$\mathbb{Z}_6^2$	$\mathbb{Z}_2^6$	$\mathbb{Z}_2^2$	$\mathbb{Z}_6^2$
$U(1)$				$\mathbb{Z}$

TABLE II (Continued.)

G_0	$E_{3,\infty}^3$	$E_{2,\infty}^2$	$E_{1,\infty}^1$	$E_{0,\infty}^0$
$SU(2)$				
$SO(3)$			$\mathbb{Z}_2^2$	
Space group #20 $C222_1$				
$\varnothing$				$\mathbb{Z}_2^2$
Z_2^T	$\mathbb{Z}_2$		$\mathbb{Z}_2^6$	$\mathbb{Z}_2^2$
Z_2		$\mathbb{Z}_2^3$	$\mathbb{Z}_2^2$	$\mathbb{Z}_2^4$
$Z_2 \times Z_2^T$	$\mathbb{Z}_2^3$	$\mathbb{Z}_2^6$	$\mathbb{Z}_2^{12}$	$\mathbb{Z}_2^4$
Z_4		$\mathbb{Z}_2^3$	$\mathbb{Z}_2^2$	$\mathbb{Z}_2^3 \times \mathbb{Z}_4$
Z_6		$\mathbb{Z}_2^3$	$\mathbb{Z}_2^2$	$\mathbb{Z}_2^3 \times \mathbb{Z}_6$
$Z_2 \times Z_2$	$\mathbb{Z}_2^2$	$\mathbb{Z}_2^9$	$\mathbb{Z}_2^8$	$\mathbb{Z}_2^6$
$Z_4 \times Z_4$	$\mathbb{Z}_4^2$	$\mathbb{Z}_2^9$	$\mathbb{Z}_2^8$	$\mathbb{Z}_2^4 \times \mathbb{Z}_4^2$
$Z_6 \times Z_6$	$\mathbb{Z}_6^2$	$\mathbb{Z}_2^9$	$\mathbb{Z}_2^8$	$\mathbb{Z}_2^4 \times \mathbb{Z}_6^2$
$U(1)$				$\mathbb{Z} \times \mathbb{Z}_2^3$
$SU(2)$				$\mathbb{Z}_2^2$
$SO(3)$			$\mathbb{Z}_2^4$	$\mathbb{Z}_2^2$
Space group #21 $C222$				
$\varnothing$				$\mathbb{Z}_2^9$
Z_2^T	$\mathbb{Z}_2$		$\mathbb{Z}_2^{15}$	$\mathbb{Z}_2^9$
Z_2		$\mathbb{Z}_2^4$	$\mathbb{Z}_2^7$	$\mathbb{Z}_2^{14}$
$Z_2 \times Z_2^T$	$\mathbb{Z}_2^3$	$\mathbb{Z}_2^8$	$\mathbb{Z}_2^{30}$	$\mathbb{Z}_2^{14}$
Z_4		$\mathbb{Z}_2^4$	$\mathbb{Z}_2^7$	$\mathbb{Z}_2^{13} \times \mathbb{Z}_4$
Z_6		$\mathbb{Z}_2^4$	$\mathbb{Z}_2^7$	$\mathbb{Z}_2^{13} \times \mathbb{Z}_6$
$Z_2 \times Z_2$	$\mathbb{Z}_2^2$	$\mathbb{Z}_2^{12}$	$\mathbb{Z}_2^{22}$	$\mathbb{Z}_2^{19}$
$Z_4 \times Z_4$	$\mathbb{Z}_4^2$	$\mathbb{Z}_2^{12}$	$\mathbb{Z}_2^{22}$	$\mathbb{Z}_2^{17} \times \mathbb{Z}_4^2$
$Z_6 \times Z_6$	$\mathbb{Z}_6^2$	$\mathbb{Z}_2^{12}$	$\mathbb{Z}_2^{22}$	$\mathbb{Z}_2^{17} \times \mathbb{Z}_6^2$
$U(1)$				$\mathbb{Z} \times \mathbb{Z}_2^{13}$
$SU(2)$				$\mathbb{Z}_2^9$
$SO(3)$			$\mathbb{Z}_2^8$	$\mathbb{Z}_2^9$
Space group #22 $F222$				
$\varnothing$				$\mathbb{Z}_2^8$
Z_2^T	$\mathbb{Z}_2$		$\mathbb{Z}_2^{13}$	$\mathbb{Z}_2^8$
Z_2		$\mathbb{Z}_2^4$	$\mathbb{Z}_2^6$	$\mathbb{Z}_2^{12}$
$Z_2 \times Z_2^T$	$\mathbb{Z}_2^3$	$\mathbb{Z}_2^8$	$\mathbb{Z}_2^{26}$	$\mathbb{Z}_2^{12}$
Z_4		$\mathbb{Z}_2^4$	$\mathbb{Z}_2^6$	$\mathbb{Z}_2^{10} \times \mathbb{Z}_4^2$
Z_6		$\mathbb{Z}_2^4$	$\mathbb{Z}_2^6$	$\mathbb{Z}_2^{11} \times \mathbb{Z}_6$
$Z_2 \times Z_2$	$\mathbb{Z}_2^2$	$\mathbb{Z}_2^{12}$	$\mathbb{Z}_2^{19}$	$\mathbb{Z}_2^{16}$
$Z_4 \times Z_4$	$\mathbb{Z}_4^2$	$\mathbb{Z}_2^{12}$	$\mathbb{Z}_2^{18} \times \mathbb{Z}_4$	$\mathbb{Z}_2^{12} \times \mathbb{Z}_4^4$
$Z_6 \times Z_6$	$\mathbb{Z}_6^2$	$\mathbb{Z}_2^{12}$	$\mathbb{Z}_2^{19}$	$\mathbb{Z}_2^{14} \times \mathbb{Z}_6^2$
$U(1)$				$\mathbb{Z} \times \mathbb{Z}_2^{10} \times \mathbb{Z}_4$
$SU(2)$				$\mathbb{Z}_2^8$
$SO(3)$			$\mathbb{Z}_2^7$	$\mathbb{Z}_2^8$
Space group #23 $I222$				
$\varnothing$				$\mathbb{Z}_2^8$
Z_2^T	$\mathbb{Z}_2$		$\mathbb{Z}_2^{12}$	$\mathbb{Z}_2^8$
Z_2		$\mathbb{Z}_2^3$	$\mathbb{Z}_2^6$	$\mathbb{Z}_2^{12}$
$Z_2 \times Z_2^T$	$\mathbb{Z}_2^3$	$\mathbb{Z}_2^6$	$\mathbb{Z}_2^{24}$	$\mathbb{Z}_2^{12}$
Z_4		$\mathbb{Z}_2^2 \times \mathbb{Z}_4$	$\mathbb{Z}_2^6$	$\mathbb{Z}_2^{11} \times \mathbb{Z}_4$
Z_6		$\mathbb{Z}_2^3$	$\mathbb{Z}_2^6$	$\mathbb{Z}_2^{11} \times \mathbb{Z}_6$
$Z_2 \times Z_2$	$\mathbb{Z}_2^2$	$\mathbb{Z}_2^9$	$\mathbb{Z}_2^{18}$	$\mathbb{Z}_2^{16}$

TABLE II (Continued.)

G_0	$E_{3,\infty}^3$	$E_{2,\infty}^2$	$E_{1,\infty}^1$	$E_{0,\infty}^0$
$Z_4 \times Z_4$	$\mathbb{Z}_4^2$	$\mathbb{Z}_2^6 \times \mathbb{Z}_4^3$	$\mathbb{Z}_2^{17} \times \mathbb{Z}_4$	$\mathbb{Z}_2^{14} \times \mathbb{Z}_4^2$
$Z_6 \times Z_6$	$\mathbb{Z}_6^2$	$\mathbb{Z}_2^9$	$\mathbb{Z}_2^{18}$	$\mathbb{Z}_2^{14} \times \mathbb{Z}_6^2$
$U(1)$				$\mathbb{Z} \times \mathbb{Z}_2^{11}$
$SU(2)$				$\mathbb{Z}_2^8$
$SO(3)$			$\mathbb{Z}_2^6$	$\mathbb{Z}_2^8$
Space group #24 $I2_12_12_1$				
$\varnothing$				$\mathbb{Z}_2^3$
Z_2^T	$\mathbb{Z}_2$		$\mathbb{Z}_2^8$	$\mathbb{Z}_2^3$
Z_2		$\mathbb{Z}_2^3$	$\mathbb{Z}_2^3$	$\mathbb{Z}_2^6$
$Z_2 \times Z_2^T$	$\mathbb{Z}_2^3$	$\mathbb{Z}_2^6$	$\mathbb{Z}_2^{16}$	$\mathbb{Z}_2^6$
Z_4		$\mathbb{Z}_2^3$	$\mathbb{Z}_2^3$	$\mathbb{Z}_2^5 \times \mathbb{Z}_4$
Z_6		$\mathbb{Z}_2^3$	$\mathbb{Z}_2^3$	$\mathbb{Z}_2^5 \times \mathbb{Z}_6$
$Z_2 \times Z_2$	$\mathbb{Z}_2^2$	$\mathbb{Z}_2^9$	$\mathbb{Z}_2^{11}$	$\mathbb{Z}_2^9$
$Z_4 \times Z_4$	$\mathbb{Z}_4^2$	$\mathbb{Z}_2^9$	$\mathbb{Z}_2^{11}$	$\mathbb{Z}_2^7 \times \mathbb{Z}_4^2$
$Z_6 \times Z_6$	$\mathbb{Z}_6^2$	$\mathbb{Z}_2^9$	$\mathbb{Z}_2^{11}$	$\mathbb{Z}_2^7 \times \mathbb{Z}_6^2$
$U(1)$				$\mathbb{Z} \times \mathbb{Z}_2^5$
$SU(2)$				$\mathbb{Z}_2^3$
$SO(3)$			$\mathbb{Z}_2^5$	$\mathbb{Z}_2^3$
Space group #25 $Pmm2$				
$\varnothing$		$\mathbb{Z}_2^4$	$\mathbb{Z}_2^4$	$\mathbb{Z}_2^8$
Z_2^T	$\mathbb{Z}_2$	$\mathbb{Z}_2^8$	$\mathbb{Z}_2^{24}$	$\mathbb{Z}_2^8$
Z_2		$\mathbb{Z}_2^{13}$	$\mathbb{Z}_2^{16}$	$\mathbb{Z}_2^{12}$
$Z_2 \times Z_2^T$	$\mathbb{Z}_2^3$	$\mathbb{Z}_2^{26}$	$\mathbb{Z}_2^{44}$	$\mathbb{Z}_2^{12}$
Z_4		$\mathbb{Z}_2^{13}$	$\mathbb{Z}_2^{16}$	$\mathbb{Z}_2^{11} \times \mathbb{Z}_4$
Z_6		$\mathbb{Z}_2^{13}$	$\mathbb{Z}_2^{16}$	$\mathbb{Z}_2^{11} \times \mathbb{Z}_6$
$Z_2 \times Z_2$	$\mathbb{Z}_2^2$	$\mathbb{Z}_2^{31}$	$\mathbb{Z}_2^{36}$	$\mathbb{Z}_2^{16}$
$Z_4 \times Z_4$	$\mathbb{Z}_2^2$	$\mathbb{Z}_2^{31}$	$\mathbb{Z}_2^{35} \times \mathbb{Z}_4$	$\mathbb{Z}_2^{14} \times \mathbb{Z}_4^2$
$Z_6 \times Z_6$	$\mathbb{Z}_2^2$	$\mathbb{Z}_2^{31}$	$\mathbb{Z}_2^{35} \times \mathbb{Z}_6$	$\mathbb{Z}_2^{14} \times \mathbb{Z}_6^2$
$U(1)$		$\mathbb{Z}_2^9$	$\mathbb{Z}_2^4$	$\mathbb{Z} \times \mathbb{Z}_2^{11}$
$SU(2)$		$\mathbb{Z}_2^5$	$\mathbb{Z}_2^4$	$\mathbb{Z}_2^8$
$SO(3)$		$\mathbb{Z}_2^9$	$\mathbb{Z}_2^{12}$	$\mathbb{Z}_2^8$
Space group #26 $Pmc2_1$				
$\varnothing$		$\mathbb{Z}_2^2$		$\mathbb{Z}_2^2$
Z_2^T	$\mathbb{Z}_2$	$\mathbb{Z}_2^4$	$\mathbb{Z}_2^9$	$\mathbb{Z}_2^2$
Z_2		$\mathbb{Z}_2^8$	$\mathbb{Z}_2^4$	$\mathbb{Z}_2^4$
$Z_2 \times Z_2^T$	$\mathbb{Z}_2^3$	$\mathbb{Z}_2^{16}$	$\mathbb{Z}_2^{18}$	$\mathbb{Z}_2^4$
Z_4		$\mathbb{Z}_2^8$	$\mathbb{Z}_2^4$	$\mathbb{Z}_2^3 \times \mathbb{Z}_4$
Z_6		$\mathbb{Z}_2^8$	$\mathbb{Z}_2^4$	$\mathbb{Z}_2^3 \times \mathbb{Z}_6$
$Z_2 \times Z_2$	$\mathbb{Z}_2^2$	$\mathbb{Z}_2^{20}$	$\mathbb{Z}_2^{13}$	$\mathbb{Z}_2^6$
$Z_4 \times Z_4$	$\mathbb{Z}_2^2$	$\mathbb{Z}_2^{20}$	$\mathbb{Z}_2^{12} \times \mathbb{Z}_4$	$\mathbb{Z}_2^4 \times \mathbb{Z}_4^2$
$Z_6 \times Z_6$	$\mathbb{Z}_2^2$	$\mathbb{Z}_2^{20}$	$\mathbb{Z}_2^{12} \times \mathbb{Z}_6$	$\mathbb{Z}_2^4 \times \mathbb{Z}_6^2$
$U(1)$		$\mathbb{Z}_2^5$		$\mathbb{Z} \times \mathbb{Z}_2^3$
$SU(2)$		$\mathbb{Z}_2^3$		$\mathbb{Z}_2^2$
$SO(3)$		$\mathbb{Z}_2^5$	$\mathbb{Z}_2^5$	$\mathbb{Z}_2^2$
Space group #27 $Pcc2$				
$\varnothing$				$\mathbb{Z}_2^4$
Z_2^T	$\mathbb{Z}_2$		$\mathbb{Z}_2^{11}$	$\mathbb{Z}_2^4$
Z_2		$\mathbb{Z}_2^4$	$\mathbb{Z}_2^4$	$\mathbb{Z}_2^8$
$Z_2 \times Z_2^T$	$\mathbb{Z}_2^3$	$\mathbb{Z}_2^8$	$\mathbb{Z}_2^{22}$	$\mathbb{Z}_2^8$

TABLE II (Continued.)

G_0	$E_{3,\infty}^3$	$E_{2,\infty}^2$	$E_{1,\infty}^1$	$E_{0,\infty}^0$
Z_4		$\mathbb{Z}_2^4$	$\mathbb{Z}_2^4$	$\mathbb{Z}_2^7 \times \mathbb{Z}_4$
Z_6		$\mathbb{Z}_2^4$	$\mathbb{Z}_2^4$	$\mathbb{Z}_2^7 \times \mathbb{Z}_6$
$Z_2 \times Z_2$	$\mathbb{Z}_2^2$	$\mathbb{Z}_2^{12}$	$\mathbb{Z}_2^{15}$	$\mathbb{Z}_2^{12}$
$Z_4 \times Z_4$	$\mathbb{Z}_2^2$	$\mathbb{Z}_2^{12}$	$\mathbb{Z}_2^{14} \times \mathbb{Z}_4$	$\mathbb{Z}_2^{10} \times \mathbb{Z}_4^2$
$Z_6 \times Z_6$	$\mathbb{Z}_2^2$	$\mathbb{Z}_2^{12}$	$\mathbb{Z}_2^{14} \times \mathbb{Z}_6$	$\mathbb{Z}_2^{10} \times \mathbb{Z}_6^2$
$U(1)$		$\mathbb{Z}_2$		$\mathbb{Z} \times \mathbb{Z}_2^7$
$SU(2)$		$\mathbb{Z}_2$		$\mathbb{Z}_2^4$
$SO(3)$		$\mathbb{Z}_2$	$\mathbb{Z}_2^7$	$\mathbb{Z}_2^4$
Space group #28 $Pma2$				
$\varnothing$		$\mathbb{Z}_2$		$\mathbb{Z}_2^3$
Z_2^T	$\mathbb{Z}_2$	$\mathbb{Z}_2^2$	$\mathbb{Z}_2^{10}$	$\mathbb{Z}_2^3$
Z_2		$\mathbb{Z}_2^6$	$\mathbb{Z}_2^4$	$\mathbb{Z}_2^6$
$Z_2 \times Z_2^T$	$\mathbb{Z}_2^3$	$\mathbb{Z}_2^{12}$	$\mathbb{Z}_2^{20}$	$\mathbb{Z}_2^6$
Z_4		$\mathbb{Z}_2^6$	$\mathbb{Z}_2^4$	$\mathbb{Z}_2^5 \times \mathbb{Z}_4$
Z_6		$\mathbb{Z}_2^6$	$\mathbb{Z}_2^4$	$\mathbb{Z}_2^5 \times \mathbb{Z}_6$
$Z_2 \times Z_2$	$\mathbb{Z}_2^2$	$\mathbb{Z}_2^{16}$	$\mathbb{Z}_2^{14}$	$\mathbb{Z}_2^9$
$Z_4 \times Z_4$	$\mathbb{Z}_2^2$	$\mathbb{Z}_2^{16}$	$\mathbb{Z}_2^{13} \times \mathbb{Z}_4$	$\mathbb{Z}_2^7 \times \mathbb{Z}_4^2$
$Z_6 \times Z_6$	$\mathbb{Z}_2^2$	$\mathbb{Z}_2^{16}$	$\mathbb{Z}_2^{13} \times \mathbb{Z}_6$	$\mathbb{Z}_2^7 \times \mathbb{Z}_6^2$
$U(1)$		$\mathbb{Z}_2^3$		$\mathbb{Z} \times \mathbb{Z}_2^5$
$SU(2)$		$\mathbb{Z}_2^2$		$\mathbb{Z}_2^3$
$SO(3)$		$\mathbb{Z}_2^3$	$\mathbb{Z}_2^6$	$\mathbb{Z}_2^3$
Space group #29 $Pca2_1$				
$\varnothing$				
Z_2^T	$\mathbb{Z}_2$		$\mathbb{Z}_2^3$	
Z_2		$\mathbb{Z}_2^3$		$\mathbb{Z}_2$
$Z_2 \times Z_2^T$	$\mathbb{Z}_2^3$	$\mathbb{Z}_2^6$	$\mathbb{Z}_2^6$	$\mathbb{Z}_2$
Z_4		$\mathbb{Z}_2^3$		$\mathbb{Z}_4$
Z_6		$\mathbb{Z}_2^3$		$\mathbb{Z}_6$
$Z_2 \times Z_2$	$\mathbb{Z}_2^2$	$\mathbb{Z}_2^9$	$\mathbb{Z}_2^3$	$\mathbb{Z}_2^2$
$Z_4 \times Z_4$	$\mathbb{Z}_2^2$	$\mathbb{Z}_2^9$	$\mathbb{Z}_2^2 \times \mathbb{Z}_4$	$\mathbb{Z}_4^2$
$Z_6 \times Z_6$	$\mathbb{Z}_2^2$	$\mathbb{Z}_2^9$	$\mathbb{Z}_2^2 \times \mathbb{Z}_6$	$\mathbb{Z}_6^2$
$U(1)$		$\mathbb{Z}_2$		$\mathbb{Z}$
$SU(2)$		$\mathbb{Z}_2$		
$SO(3)$		$\mathbb{Z}_2$	$\mathbb{Z}_2^3$	
Space group #30 $Pnc2$				
$\varnothing$				$\mathbb{Z}_2^2$
Z_2^T	$\mathbb{Z}_2$		$\mathbb{Z}_2^6$	$\mathbb{Z}_2^2$
Z_2		$\mathbb{Z}_2^3$	$\mathbb{Z}_2^2$	$\mathbb{Z}_2^4$
$Z_2 \times Z_2^T$	$\mathbb{Z}_2^3$	$\mathbb{Z}_2^6$	$\mathbb{Z}_2^{12}$	$\mathbb{Z}_2^4$
Z_4		$\mathbb{Z}_2^3$	$\mathbb{Z}_2^2$	$\mathbb{Z}_2^3 \times \mathbb{Z}_4$
Z_6		$\mathbb{Z}_2^3$	$\mathbb{Z}_2^2$	$\mathbb{Z}_2^3 \times \mathbb{Z}_6$
$Z_2 \times Z_2$	$\mathbb{Z}_2^2$	$\mathbb{Z}_2^9$	$\mathbb{Z}_2^8$	$\mathbb{Z}_2^6$
$Z_4 \times Z_4$	$\mathbb{Z}_2^2$	$\mathbb{Z}_2^9$	$\mathbb{Z}_2^7 \times \mathbb{Z}_4$	$\mathbb{Z}_2^4 \times \mathbb{Z}_4^2$
$Z_6 \times Z_6$	$\mathbb{Z}_2^2$	$\mathbb{Z}_2^9$	$\mathbb{Z}_2^7 \times \mathbb{Z}_6$	$\mathbb{Z}_2^4 \times \mathbb{Z}_6^2$
$U(1)$		$\mathbb{Z}_2$		$\mathbb{Z} \times \mathbb{Z}_2^3$
$SU(2)$		$\mathbb{Z}_2$		$\mathbb{Z}_2^2$
$SO(3)$		$\mathbb{Z}_2$	$\mathbb{Z}_2^4$	$\mathbb{Z}_2^2$
Space group #31 $Pmn2_1$				
$\varnothing$		$\mathbb{Z}_2$		$\mathbb{Z}_2$

TABLE II (Continued.)

G_0	$E^3_{3,\infty}$	$E^2_{2,\infty}$	$E^1_{1,\infty}$	$E^0_{0,\infty}$
Z_2^T	$\mathbb{Z}_2$	$\mathbb{Z}_2^2$	$\mathbb{Z}_2^5$	$\mathbb{Z}_2$
Z_2		$\mathbb{Z}_2^5$	$\mathbb{Z}_2^2$	$\mathbb{Z}_2^2$
$Z_2 \times Z_2^T$	$\mathbb{Z}_2^3$	$\mathbb{Z}_2^{10}$	$\mathbb{Z}_2^{10}$	$\mathbb{Z}_2^2$
Z_4		$\mathbb{Z}_2^4 \times \mathbb{Z}_4$	$\mathbb{Z}_2^2$	$\mathbb{Z}_2 \times \mathbb{Z}_4$
Z_6		$\mathbb{Z}_2^5$	$\mathbb{Z}_2^2$	$\mathbb{Z}_2 \times \mathbb{Z}_6$
$Z_2 \times Z_2$	$\mathbb{Z}_2^2$	$\mathbb{Z}_2^{13}$	$\mathbb{Z}_2^7$	$\mathbb{Z}_2^3$
$Z_4 \times Z_4$	$\mathbb{Z}_2^2$	$\mathbb{Z}_2^{10} \times \mathbb{Z}_4^3$	$\mathbb{Z}_2^5 \times \mathbb{Z}_4^2$	$\mathbb{Z}_2 \times \mathbb{Z}_4^2$
$Z_6 \times Z_6$	$\mathbb{Z}_2^2$	$\mathbb{Z}_2^{13}$	$\mathbb{Z}_2^6 \times \mathbb{Z}_6$	$\mathbb{Z}_2 \times \mathbb{Z}_6^2$
$U(1)$		$\mathbb{Z}_2^3$		$\mathbb{Z} \times \mathbb{Z}_2$
$SU(2)$		$\mathbb{Z}_2^2$		$\mathbb{Z}_2$
$SO(3)$		$\mathbb{Z}_2^3$	$\mathbb{Z}_2^3$	$\mathbb{Z}_2$
Space group #32 $Pba2$				
$\varnothing$				$\mathbb{Z}_2^2$
Z_2^T	$\mathbb{Z}_2$		$\mathbb{Z}_2^6$	$\mathbb{Z}_2^2$
Z_2		$\mathbb{Z}_2^3$	$\mathbb{Z}_2^2$	$\mathbb{Z}_2^4$
$Z_2 \times Z_2^T$	$\mathbb{Z}_2^3$	$\mathbb{Z}_2^6$	$\mathbb{Z}_2^{12}$	$\mathbb{Z}_2^4$
Z_4		$\mathbb{Z}_2^3$	$\mathbb{Z}_2^2$	$\mathbb{Z}_2^3 \times \mathbb{Z}_4$
Z_6		$\mathbb{Z}_2^3$	$\mathbb{Z}_2^2$	$\mathbb{Z}_2^3 \times \mathbb{Z}_6$
$Z_2 \times Z_2$	$\mathbb{Z}_2^2$	$\mathbb{Z}_2^9$	$\mathbb{Z}_2^8$	$\mathbb{Z}_2^6$
$Z_4 \times Z_4$	$\mathbb{Z}_2^2$	$\mathbb{Z}_2^9$	$\mathbb{Z}_2^7 \times \mathbb{Z}_4$	$\mathbb{Z}_2^4 \times \mathbb{Z}_4^2$
$Z_6 \times Z_6$	$\mathbb{Z}_2^2$	$\mathbb{Z}_2^9$	$\mathbb{Z}_2^7 \times \mathbb{Z}_6$	$\mathbb{Z}_2^4 \times \mathbb{Z}_6^2$
$U(1)$		$\mathbb{Z}_2$		$\mathbb{Z} \times \mathbb{Z}_2^3$
$SU(2)$		$\mathbb{Z}_2$		$\mathbb{Z}_2^2$
$SO(3)$		$\mathbb{Z}_2$	$\mathbb{Z}_2^4$	$\mathbb{Z}_2^2$
Space group #33 $Pna2_1$				
$\varnothing$				
Z_2^T	$\mathbb{Z}_2$		$\mathbb{Z}_2^2$	
Z_2		$\mathbb{Z}_2^2$		$\mathbb{Z}_2$
$Z_2 \times Z_2^T$	$\mathbb{Z}_2^3$	$\mathbb{Z}_2^4$	$\mathbb{Z}_2^4$	$\mathbb{Z}_2$
Z_4		$\mathbb{Z}_2 \times \mathbb{Z}_4$		$\mathbb{Z}_4$
Z_6		$\mathbb{Z}_2^2$		$\mathbb{Z}_6$
$Z_2 \times Z_2$	$\mathbb{Z}_2^2$	$\mathbb{Z}_2^6$	$\mathbb{Z}_2^2$	$\mathbb{Z}_2^2$
$Z_4 \times Z_4$	$\mathbb{Z}_2^2$	$\mathbb{Z}_2^3 \times \mathbb{Z}_4^3$	$\mathbb{Z}_4^2$	$\mathbb{Z}_4^2$
$Z_6 \times Z_6$	$\mathbb{Z}_2^2$	$\mathbb{Z}_2^6$	$\mathbb{Z}_2 \times \mathbb{Z}_6$	$\mathbb{Z}_6^2$
$U(1)$		$\mathbb{Z}_2$		$\mathbb{Z}$
$SU(2)$		$\mathbb{Z}_2$		
$SO(3)$		$\mathbb{Z}_2$	$\mathbb{Z}_2^2$	
Space group #34 $Pnn2$				
$\varnothing$				$\mathbb{Z}_2^2$
Z_2^T	$\mathbb{Z}_2$		$\mathbb{Z}_2^6$	$\mathbb{Z}_2^2$
Z_2		$\mathbb{Z}_2^3$	$\mathbb{Z}_2^2$	$\mathbb{Z}_2^4$
$Z_2 \times Z_2^T$	$\mathbb{Z}_2^3$	$\mathbb{Z}_2^6$	$\mathbb{Z}_2^{12}$	$\mathbb{Z}_2^4$
Z_4		$\mathbb{Z}_2^3$	$\mathbb{Z}_2^2$	$\mathbb{Z}_2^3 \times \mathbb{Z}_4$
Z_6		$\mathbb{Z}_2^3$	$\mathbb{Z}_2^2$	$\mathbb{Z}_2^3 \times \mathbb{Z}_6$
$Z_2 \times Z_2$	$\mathbb{Z}_2^2$	$\mathbb{Z}_2^9$	$\mathbb{Z}_2^8$	$\mathbb{Z}_2^6$
$Z_4 \times Z_4$	$\mathbb{Z}_2^2$	$\mathbb{Z}_2^9$	$\mathbb{Z}_2^7 \times \mathbb{Z}_4$	$\mathbb{Z}_2^4 \times \mathbb{Z}_4^2$
$Z_6 \times Z_6$	$\mathbb{Z}_2^2$	$\mathbb{Z}_2^9$	$\mathbb{Z}_2^7 \times \mathbb{Z}_6$	$\mathbb{Z}_2^4 \times \mathbb{Z}_6^2$
$U(1)$		$\mathbb{Z}_2$		$\mathbb{Z} \times \mathbb{Z}_2^3$
$SU(2)$		$\mathbb{Z}_2$		$\mathbb{Z}_2^2$

TABLE II (Continued.)

G_0	$E^3_{3,\infty}$	$E^2_{2,\infty}$	$E^1_{1,\infty}$	$E^0_{0,\infty}$
$SO(3)$		$\mathbb{Z}_2$	$\mathbb{Z}_2^4$	$\mathbb{Z}_2^2$
Space group #35 $Cmm2$				
$\varnothing$		$\mathbb{Z}_2^2$	$\mathbb{Z}_2^2$	$\mathbb{Z}_2^5$
Z_2^T	$\mathbb{Z}_2$	$\mathbb{Z}_2^4$	$\mathbb{Z}_2^{15}$	$\mathbb{Z}_2^5$
Z_2		$\mathbb{Z}_2^8$	$\mathbb{Z}_2^9$	$\mathbb{Z}_2^8$
$Z_2 \times Z_2^T$	$\mathbb{Z}_2^3$	$\mathbb{Z}_2^{16}$	$\mathbb{Z}_2^{28}$	$\mathbb{Z}_2^8$
Z_4		$\mathbb{Z}_2^8$	$\mathbb{Z}_2^9$	$\mathbb{Z}_2^7 \times \mathbb{Z}_4$
Z_6		$\mathbb{Z}_2^8$	$\mathbb{Z}_2^9$	$\mathbb{Z}_2^7 \times \mathbb{Z}_6$
$Z_2 \times Z_2$	$\mathbb{Z}_2^2$	$\mathbb{Z}_2^{20}$	$\mathbb{Z}_2^{22}$	$\mathbb{Z}_2^{11}$
$Z_4 \times Z_4$	$\mathbb{Z}_2^2$	$\mathbb{Z}_2^{20}$	$\mathbb{Z}_2^{21} \times \mathbb{Z}_4$	$\mathbb{Z}_2^9 \times \mathbb{Z}_4^2$
$Z_6 \times Z_6$	$\mathbb{Z}_2^2$	$\mathbb{Z}_2^{20}$	$\mathbb{Z}_2^{21} \times \mathbb{Z}_6$	$\mathbb{Z}_2^9 \times \mathbb{Z}_6^2$
$U(1)$		$\mathbb{Z}_2^5$	$\mathbb{Z}_2^2$	$\mathbb{Z} \times \mathbb{Z}_2^7$
$SU(2)$		$\mathbb{Z}_2^3$	$\mathbb{Z}_2^2$	$\mathbb{Z}_2^5$
$SO(3)$		$\mathbb{Z}_2^5$	$\mathbb{Z}_2^8$	$\mathbb{Z}_2^5$
Space group #36 $Cmc2_1$				
$\varnothing$		$\mathbb{Z}_2$		$\mathbb{Z}_2$
Z_2^T	$\mathbb{Z}_2$	$\mathbb{Z}_2^2$	$\mathbb{Z}_2^5$	$\mathbb{Z}_2$
Z_2		$\mathbb{Z}_2^5$	$\mathbb{Z}_2^2$	$\mathbb{Z}_2^2$
$Z_2 \times Z_2^T$	$\mathbb{Z}_2^3$	$\mathbb{Z}_2^{10}$	$\mathbb{Z}_2^{10}$	$\mathbb{Z}_2^2$
Z_4		$\mathbb{Z}_2^5$	$\mathbb{Z}_2^2$	$\mathbb{Z}_2 \times \mathbb{Z}_4$
Z_6		$\mathbb{Z}_2^5$	$\mathbb{Z}_2^2$	$\mathbb{Z}_2 \times \mathbb{Z}_6$
$Z_2 \times Z_2$	$\mathbb{Z}_2^2$	$\mathbb{Z}_2^{13}$	$\mathbb{Z}_2^7$	$\mathbb{Z}_2^3$
$Z_4 \times Z_4$	$\mathbb{Z}_2^2$	$\mathbb{Z}_2^{13}$	$\mathbb{Z}_2^6 \times \mathbb{Z}_4$	$\mathbb{Z}_2 \times \mathbb{Z}_4^2$
$Z_6 \times Z_6$	$\mathbb{Z}_2^2$	$\mathbb{Z}_2^{13}$	$\mathbb{Z}_2^6 \times \mathbb{Z}_6$	$\mathbb{Z}_2 \times \mathbb{Z}_6^2$
$U(1)$		$\mathbb{Z}_2^3$		$\mathbb{Z} \times \mathbb{Z}_2$
$SU(2)$		$\mathbb{Z}_2^2$		$\mathbb{Z}_2$
$SO(3)$		$\mathbb{Z}_2^3$	$\mathbb{Z}_2^3$	$\mathbb{Z}_2$
Space group #37 $Ccc2$				
$\varnothing$				$\mathbb{Z}_2^3$
Z_2^T	$\mathbb{Z}_2$		$\mathbb{Z}_2^8$	$\mathbb{Z}_2^3$
Z_2		$\mathbb{Z}_2^3$	$\mathbb{Z}_2^3$	$\mathbb{Z}_2^6$
$Z_2 \times Z_2^T$	$\mathbb{Z}_2^3$	$\mathbb{Z}_2^6$	$\mathbb{Z}_2^{16}$	$\mathbb{Z}_2^6$
Z_4		$\mathbb{Z}_2^3$	$\mathbb{Z}_2^3$	$\mathbb{Z}_2^5 \times \mathbb{Z}_4$
Z_6		$\mathbb{Z}_2^3$	$\mathbb{Z}_2^3$	$\mathbb{Z}_2^5 \times \mathbb{Z}_6$
$Z_2 \times Z_2$	$\mathbb{Z}_2^2$	$\mathbb{Z}_2^9$	$\mathbb{Z}_2^{11}$	$\mathbb{Z}_2^9$
$Z_4 \times Z_4$	$\mathbb{Z}_2^2$	$\mathbb{Z}_2^9$	$\mathbb{Z}_2^{10} \times \mathbb{Z}_4$	$\mathbb{Z}_2^7 \times \mathbb{Z}_4^2$
$Z_6 \times Z_6$	$\mathbb{Z}_2^2$	$\mathbb{Z}_2^9$	$\mathbb{Z}_2^{10} \times \mathbb{Z}_6$	$\mathbb{Z}_2^7 \times \mathbb{Z}_6^2$
$U(1)$		$\mathbb{Z}_2$		$\mathbb{Z} \times \mathbb{Z}_2^5$
$SU(2)$		$\mathbb{Z}_2$		$\mathbb{Z}_2^3$
$SO(3)$		$\mathbb{Z}_2$	$\mathbb{Z}_2^5$	$\mathbb{Z}_2^3$
Space group #38 $Amm2$				
$\varnothing$		$\mathbb{Z}_2^3$	$\mathbb{Z}_2^2$	$\mathbb{Z}_2^4$
Z_2^T	$\mathbb{Z}_2$	$\mathbb{Z}_2^6$	$\mathbb{Z}_2^{14}$	$\mathbb{Z}_2^4$
Z_2		$\mathbb{Z}_2^{10}$	$\mathbb{Z}_2^9$	$\mathbb{Z}_2^6$
$Z_2 \times Z_2^T$	$\mathbb{Z}_2^3$	$\mathbb{Z}_2^{20}$	$\mathbb{Z}_2^{26}$	$\mathbb{Z}_2^6$
Z_4		$\mathbb{Z}_2^{10}$	$\mathbb{Z}_2^9$	$\mathbb{Z}_2^5 \times \mathbb{Z}_4$
Z_6		$\mathbb{Z}_2^{10}$	$\mathbb{Z}_2^9$	$\mathbb{Z}_2^5 \times \mathbb{Z}_6$
$Z_2 \times Z_2$	$\mathbb{Z}_2^2$	$\mathbb{Z}_2^{24}$	$\mathbb{Z}_2^{21}$	$\mathbb{Z}_2^8$
$Z_4 \times Z_4$	$\mathbb{Z}_2^2$	$\mathbb{Z}_2^{24}$	$\mathbb{Z}_2^{20} \times \mathbb{Z}_4$	$\mathbb{Z}_2^6 \times \mathbb{Z}_4^2$

TABLE II (Continued.)

G_0	$E^3_{3,\infty}$	$E^2_{2,\infty}$	$E^1_{1,\infty}$	$E^0_{0,\infty}$
$Z_6\times Z_6$	$\mathbb{Z}_2^2$	$\mathbb{Z}_2^{24}$	$\mathbb{Z}_2^{20}\times\mathbb{Z}_6$	$\mathbb{Z}_2^6\times\mathbb{Z}_6^2$
$U(1)$		$\mathbb{Z}_2^7$	$\mathbb{Z}_2^2$	$\mathbb{Z}\times\mathbb{Z}_2^5$
$SU(2)$		$\mathbb{Z}_2^4$	$\mathbb{Z}_2^2$	$\mathbb{Z}_2^4$
$SO(3)$		$\mathbb{Z}_2^7$	$\mathbb{Z}_2^7$	$\mathbb{Z}_2^4$
Space group #39 $Aem2$				
$\varnothing$		$\mathbb{Z}_2$		$\mathbb{Z}_2^3$
Z_2^T	$\mathbb{Z}_2$	$\mathbb{Z}_2^2$	$\mathbb{Z}_2^{10}$	$\mathbb{Z}_2^3$
Z_2		$\mathbb{Z}_2^6$	$\mathbb{Z}_2^4$	$\mathbb{Z}_2^6$
$Z_2\times Z_2^T$	$\mathbb{Z}_2^3$	$\mathbb{Z}_2^{12}$	$\mathbb{Z}_2^{20}$	$\mathbb{Z}_2^6$
Z_4		$\mathbb{Z}_2^6$	$\mathbb{Z}_2^4$	$\mathbb{Z}_2^5\times\mathbb{Z}_4$
Z_6		$\mathbb{Z}_2^6$	$\mathbb{Z}_2^4$	$\mathbb{Z}_2^5\times\mathbb{Z}_6$
$Z_2\times Z_2$	$\mathbb{Z}_2^2$	$\mathbb{Z}_2^{16}$	$\mathbb{Z}_2^{14}$	$\mathbb{Z}_2^9$
$Z_4\times Z_4$	$\mathbb{Z}_2^2$	$\mathbb{Z}_2^{16}$	$\mathbb{Z}_2^{13}\times\mathbb{Z}_4$	$\mathbb{Z}_2^7\times\mathbb{Z}_4^2$
$Z_6\times Z_6$	$\mathbb{Z}_2^2$	$\mathbb{Z}_2^{16}$	$\mathbb{Z}_2^{13}\times\mathbb{Z}_6$	$\mathbb{Z}_2^7\times\mathbb{Z}_6^2$
$U(1)$		$\mathbb{Z}_2^3$		$\mathbb{Z}\times\mathbb{Z}_2^5$
$SU(2)$		$\mathbb{Z}_2^2$		$\mathbb{Z}_2^3$
$SO(3)$		$\mathbb{Z}_2^3$	$\mathbb{Z}_2^6$	$\mathbb{Z}_2^3$
Space group #40 $Ama2$				
$\varnothing$		$\mathbb{Z}_2$		$\mathbb{Z}_2^2$
Z_2^T	$\mathbb{Z}_2$	$\mathbb{Z}_2^2$	$\mathbb{Z}_2^7$	$\mathbb{Z}_2^2$
Z_2		$\mathbb{Z}_2^5$	$\mathbb{Z}_2^3$	$\mathbb{Z}_2^4$
$Z_2\times Z_2^T$	$\mathbb{Z}_2^3$	$\mathbb{Z}_2^{10}$	$\mathbb{Z}_2^{14}$	$\mathbb{Z}_2^4$
Z_4		$\mathbb{Z}_2^5$	$\mathbb{Z}_2^3$	$\mathbb{Z}_2^3\times\mathbb{Z}_4$
Z_6		$\mathbb{Z}_2^5$	$\mathbb{Z}_2^3$	$\mathbb{Z}_2^3\times\mathbb{Z}_6$
$Z_2\times Z_2$	$\mathbb{Z}_2^2$	$\mathbb{Z}_2^{13}$	$\mathbb{Z}_2^{10}$	$\mathbb{Z}_2^6$
$Z_4\times Z_4$	$\mathbb{Z}_2^2$	$\mathbb{Z}_2^{13}$	$\mathbb{Z}_2^9\times\mathbb{Z}_4$	$\mathbb{Z}_2^4\times\mathbb{Z}_4^2$
$Z_6\times Z_6$	$\mathbb{Z}_2^2$	$\mathbb{Z}_2^{13}$	$\mathbb{Z}_2^9\times\mathbb{Z}_6$	$\mathbb{Z}_2^4\times\mathbb{Z}_6^2$
$U(1)$		$\mathbb{Z}_2^3$		$\mathbb{Z}\times\mathbb{Z}_2^3$
$SU(2)$		$\mathbb{Z}_2^2$		$\mathbb{Z}_2^2$
$SO(3)$		$\mathbb{Z}_2^3$	$\mathbb{Z}_2^4$	$\mathbb{Z}_2^2$
Space group #41 $Aea2$				
$\varnothing$				$\mathbb{Z}_2$
Z_2^T	$\mathbb{Z}_2$		$\mathbb{Z}_2^4$	$\mathbb{Z}_2$
Z_2		$\mathbb{Z}_2^3$	$\mathbb{Z}_2$	$\mathbb{Z}_2^2$
$Z_2\times Z_2^T$	$\mathbb{Z}_2^3$	$\mathbb{Z}_2^6$	$\mathbb{Z}_2^8$	$\mathbb{Z}_2^2$
Z_4		$\mathbb{Z}_2^3$	$\mathbb{Z}_2$	$\mathbb{Z}_2\times\mathbb{Z}_4$
Z_6		$\mathbb{Z}_2^3$	$\mathbb{Z}_2$	$\mathbb{Z}_2\times\mathbb{Z}_6$
$Z_2\times Z_2$	$\mathbb{Z}_2^2$	$\mathbb{Z}_2^9$	$\mathbb{Z}_2^5$	$\mathbb{Z}_2^3$
$Z_4\times Z_4$	$\mathbb{Z}_2^2$	$\mathbb{Z}_2^9$	$\mathbb{Z}_2^4\times\mathbb{Z}_4$	$\mathbb{Z}_2\times\mathbb{Z}_4^2$
$Z_6\times Z_6$	$\mathbb{Z}_2^2$	$\mathbb{Z}_2^9$	$\mathbb{Z}_2^4\times\mathbb{Z}_6$	$\mathbb{Z}_2\times\mathbb{Z}_6^2$
$U(1)$		$\mathbb{Z}_2$		$\mathbb{Z}\times\mathbb{Z}_2$
$SU(2)$		$\mathbb{Z}_2$		$\mathbb{Z}_2$
$SO(3)$		$\mathbb{Z}_2$	$\mathbb{Z}_2^3$	$\mathbb{Z}_2$
Space group #42 $Fmm2$				
$\varnothing$		$\mathbb{Z}_2^2$	$\mathbb{Z}_2$	$\mathbb{Z}_2^3$
Z_2^T	$\mathbb{Z}_2$	$\mathbb{Z}_2^4$	$\mathbb{Z}_2^{11}$	$\mathbb{Z}_2^3$
Z_2		$\mathbb{Z}_2^8$	$\mathbb{Z}_2^6$	$\mathbb{Z}_2^5$
$Z_2\times Z_2^T$	$\mathbb{Z}_2^3$	$\mathbb{Z}_2^{16}$	$\mathbb{Z}_2^{21}$	$\mathbb{Z}_2^5$
Z_4		$\mathbb{Z}_2^8$	$\mathbb{Z}_2^6$	$\mathbb{Z}_2^4\times\mathbb{Z}_4$

TABLE II (Continued.)

G_0	$E^3_{3,\infty}$	$E^2_{2,\infty}$	$E^1_{1,\infty}$	$E^0_{0,\infty}$
Z_6		$\mathbb{Z}_2^8$	$\mathbb{Z}_2^6$	$\mathbb{Z}_2^4\times\mathbb{Z}_6$
$Z_2\times Z_2$	$\mathbb{Z}_2^2$	$\mathbb{Z}_2^{20}$	$\mathbb{Z}_2^{16}$	$\mathbb{Z}_2^7$
$Z_4\times Z_4$	$\mathbb{Z}_2^2$	$\mathbb{Z}_2^{20}$	$\mathbb{Z}_2^{15}\times\mathbb{Z}_4$	$\mathbb{Z}_2^5\times\mathbb{Z}_4^2$
$Z_6\times Z_6$	$\mathbb{Z}_2^2$	$\mathbb{Z}_2^{20}$	$\mathbb{Z}_2^{15}\times\mathbb{Z}_6$	$\mathbb{Z}_2^5\times\mathbb{Z}_6^2$
$U(1)$		$\mathbb{Z}_2^5$	$\mathbb{Z}_2$	$\mathbb{Z}\times\mathbb{Z}_2^4$
$SU(2)$		$\mathbb{Z}_2^3$	$\mathbb{Z}_2$	$\mathbb{Z}_2^3$
$SO(3)$		$\mathbb{Z}_2^5$	$\mathbb{Z}_2^6$	$\mathbb{Z}_2^3$
Space group #43 $Fdd2$				
$\varnothing$				$\mathbb{Z}_2$
Z_2^T	$\mathbb{Z}_2$		$\mathbb{Z}_2^3$	$\mathbb{Z}_2$
Z_2		$\mathbb{Z}_2^2$	$\mathbb{Z}_2$	$\mathbb{Z}_2^2$
$Z_2\times Z_2^T$	$\mathbb{Z}_2^3$	$\mathbb{Z}_2^4$	$\mathbb{Z}_2^6$	$\mathbb{Z}_2^2$
Z_4		$\mathbb{Z}_2^2$	$\mathbb{Z}_2$	$\mathbb{Z}_2\times\mathbb{Z}_4$
Z_6		$\mathbb{Z}_2^2$	$\mathbb{Z}_2$	$\mathbb{Z}_2\times\mathbb{Z}_6$
$Z_2\times Z_2$	$\mathbb{Z}_2^2$	$\mathbb{Z}_2^6$	$\mathbb{Z}_2^4$	$\mathbb{Z}_2^3$
$Z_4\times Z_4$	$\mathbb{Z}_2^2$	$\mathbb{Z}_2^6$	$\mathbb{Z}_2^3\times\mathbb{Z}_4$	$\mathbb{Z}_2\times\mathbb{Z}_4^2$
$Z_6\times Z_6$	$\mathbb{Z}_2^2$	$\mathbb{Z}_2^6$	$\mathbb{Z}_2^3\times\mathbb{Z}_6$	$\mathbb{Z}_2\times\mathbb{Z}_6^2$
$U(1)$		$\mathbb{Z}_2$		$\mathbb{Z}\times\mathbb{Z}_2$
$SU(2)$		$\mathbb{Z}_2$		$\mathbb{Z}_2$
$SO(3)$		$\mathbb{Z}_2$	$\mathbb{Z}_2^2$	$\mathbb{Z}_2$
Space group #44 $Imm2$				
$\varnothing$		$\mathbb{Z}_2^2$	$\mathbb{Z}_2^2$	$\mathbb{Z}_2^4$
Z_2^T	$\mathbb{Z}_2$	$\mathbb{Z}_2^4$	$\mathbb{Z}_2^{12}$	$\mathbb{Z}_2^4$
Z_2		$\mathbb{Z}_2^7$	$\mathbb{Z}_2^8$	$\mathbb{Z}_2^6$
$Z_2\times Z_2^T$	$\mathbb{Z}_2^3$	$\mathbb{Z}_2^{14}$	$\mathbb{Z}_2^{22}$	$\mathbb{Z}_2^6$
Z_4		$\mathbb{Z}_2^6\times\mathbb{Z}_4$	$\mathbb{Z}_2^8$	$\mathbb{Z}_2^5\times\mathbb{Z}_4$
Z_6		$\mathbb{Z}_2^7$	$\mathbb{Z}_2^8$	$\mathbb{Z}_2^5\times\mathbb{Z}_6$
$Z_2\times Z_2$	$\mathbb{Z}_2^2$	$\mathbb{Z}_2^{17}$	$\mathbb{Z}_2^{18}$	$\mathbb{Z}_2^8$
$Z_4\times Z_4$	$\mathbb{Z}_2^2$	$\mathbb{Z}_2^{14}\times\mathbb{Z}_4^3$	$\mathbb{Z}_2^{16}\times\mathbb{Z}_4^2$	$\mathbb{Z}_2^6\times\mathbb{Z}_4^2$
$Z_6\times Z_6$	$\mathbb{Z}_2^2$	$\mathbb{Z}_2^{17}$	$\mathbb{Z}_2^{17}\times\mathbb{Z}_6$	$\mathbb{Z}_2^6\times\mathbb{Z}_6^2$
$U(1)$		$\mathbb{Z}_2^5$	$\mathbb{Z}_2^2$	$\mathbb{Z}\times\mathbb{Z}_2^5$
$SU(2)$		$\mathbb{Z}_2^3$	$\mathbb{Z}_2^2$	$\mathbb{Z}_2^4$
$SO(3)$		$\mathbb{Z}_2^5$	$\mathbb{Z}_2^6$	$\mathbb{Z}_2^4$
Space group #45 $Iba2$				
$\varnothing$				$\mathbb{Z}_2^2$
Z_2^T	$\mathbb{Z}_2$		$\mathbb{Z}_2^6$	$\mathbb{Z}_2^2$
Z_2		$\mathbb{Z}_2^3$	$\mathbb{Z}_2^2$	$\mathbb{Z}_2^4$
$Z_2\times Z_2^T$	$\mathbb{Z}_2^3$	$\mathbb{Z}_2^6$	$\mathbb{Z}_2^{12}$	$\mathbb{Z}_2^4$
Z_4		$\mathbb{Z}_2^3$	$\mathbb{Z}_2^2$	$\mathbb{Z}_2^3\times\mathbb{Z}_4$
Z_6		$\mathbb{Z}_2^3$	$\mathbb{Z}_2^2$	$\mathbb{Z}_2^3\times\mathbb{Z}_6$
$Z_2\times Z_2$	$\mathbb{Z}_2^2$	$\mathbb{Z}_2^9$	$\mathbb{Z}_2^8$	$\mathbb{Z}_2^6$
$Z_4\times Z_4$	$\mathbb{Z}_2^2$	$\mathbb{Z}_2^9$	$\mathbb{Z}_2^7\times\mathbb{Z}_4$	$\mathbb{Z}_2^4\times\mathbb{Z}_4^2$
$Z_6\times Z_6$	$\mathbb{Z}_2^2$	$\mathbb{Z}_2^9$	$\mathbb{Z}_2^7\times\mathbb{Z}_6$	$\mathbb{Z}_2^4\times\mathbb{Z}_6^2$
$U(1)$		$\mathbb{Z}_2$		$\mathbb{Z}\times\mathbb{Z}_2^3$
$SU(2)$		$\mathbb{Z}_2$		$\mathbb{Z}_2^2$
$SO(3)$		$\mathbb{Z}_2$	$\mathbb{Z}_2^4$	$\mathbb{Z}_2^2$
Space group #46 $Ima2$				
$\varnothing$		$\mathbb{Z}_2$		$\mathbb{Z}_2^2$
Z_2^T	$\mathbb{Z}_2$	$\mathbb{Z}_2^2$	$\mathbb{Z}_2^7$	$\mathbb{Z}_2^2$

TABLE II (Continued.)

G_0	$E^3_{3,\infty}$	$E^2_{2,\infty}$	$E^1_{1,\infty}$	$E^0_{0,\infty}$
Z_2		$\mathbb{Z}_2^5$	$\mathbb{Z}_2^3$	$\mathbb{Z}_2^4$
$Z_2 \times Z_2^T$	$\mathbb{Z}_2^3$	$\mathbb{Z}_2^{10}$	$\mathbb{Z}_2^{14}$	$\mathbb{Z}_2^4$
Z_4		$\mathbb{Z}_2^5$	$\mathbb{Z}_2^3$	$\mathbb{Z}_2^3 \times \mathbb{Z}_4$
Z_6		$\mathbb{Z}_2^5$	$\mathbb{Z}_2^3$	$\mathbb{Z}_2^3 \times \mathbb{Z}_6$
$Z_2 \times Z_2$	$\mathbb{Z}_2^2$	$\mathbb{Z}_2^{13}$	$\mathbb{Z}_2^{10}$	$\mathbb{Z}_2^6$
$Z_4 \times Z_4$	$\mathbb{Z}_2^2$	$\mathbb{Z}_2^{13}$	$\mathbb{Z}_2^9 \times \mathbb{Z}_4$	$\mathbb{Z}_2^4 \times \mathbb{Z}_4^2$
$Z_6 \times Z_6$	$\mathbb{Z}_2^2$	$\mathbb{Z}_2^{13}$	$\mathbb{Z}_2^9 \times \mathbb{Z}_6$	$\mathbb{Z}_2^4 \times \mathbb{Z}_6^2$
$U(1)$		$\mathbb{Z}_2^3$		$\mathbb{Z} \times \mathbb{Z}_2^3$
$SU(2)$		$\mathbb{Z}_2^2$		$\mathbb{Z}_2^2$
$SO(3)$		$\mathbb{Z}_2^3$	$\mathbb{Z}_2^4$	$\mathbb{Z}_2^2$
Space group #47 *Pmmm*				
$\varnothing$		$\mathbb{Z}_2^6$	$\mathbb{Z}_2^{12}$	$\mathbb{Z}_2^{24}$
Z_2^T	$\mathbb{Z}_2$	$\mathbb{Z}_2^{12}$	$\mathbb{Z}_2^{48}$	$\mathbb{Z}_2^{24}$
Z_2		$\mathbb{Z}_2^{18}$	$\mathbb{Z}_2^{36}$	$\mathbb{Z}_2^{32}$
$Z_2 \times Z_2^T$	$\mathbb{Z}_2^3$	$\mathbb{Z}_2^{36}$	$\mathbb{Z}_2^{84}$	$\mathbb{Z}_2^{32}$
Z_4		$\mathbb{Z}_2^{18}$	$\mathbb{Z}_2^{36}$	$\mathbb{Z}_2^{31} \times \mathbb{Z}_4$
Z_6		$\mathbb{Z}_2^{18}$	$\mathbb{Z}_2^{36}$	$\mathbb{Z}_2^{31} \times \mathbb{Z}_6$
$Z_2 \times Z_2$	$\mathbb{Z}_2^2$	$\mathbb{Z}_2^{42}$	$\mathbb{Z}_2^{72}$	$\mathbb{Z}_2^{40}$
$Z_4 \times Z_4$	$\mathbb{Z}_2^2$	$\mathbb{Z}_2^{42}$	$\mathbb{Z}_2^{72}$	$\mathbb{Z}_2^{38} \times \mathbb{Z}_4^2$
$Z_6 \times Z_6$	$\mathbb{Z}_2^2$	$\mathbb{Z}_2^{42}$	$\mathbb{Z}_2^{72}$	$\mathbb{Z}_2^{38} \times \mathbb{Z}_6^2$
$U(1)$		$\mathbb{Z}_2^{13}$	$\mathbb{Z}_2^{12}$	$\mathbb{Z} \times \mathbb{Z}_2^{31}$
$SU(2)$		$\mathbb{Z}_2^7$	$\mathbb{Z}_2^{12}$	$\mathbb{Z}_2^{24}$
$SO(3)$		$\mathbb{Z}_2^{13}$	$\mathbb{Z}_2^{24}$	$\mathbb{Z}_2^{24}$
Space group #48 *Pnnn*				
$\varnothing$				$\mathbb{Z}_2^{10}$
Z_2^T	$\mathbb{Z}_2$		$\mathbb{Z}_2^{14}$	$\mathbb{Z}_2^{10}$
Z_2		$\mathbb{Z}_2^4$	$\mathbb{Z}_2^6$	$\mathbb{Z}_2^{16}$
$Z_2 \times Z_2^T$	$\mathbb{Z}_2^3$	$\mathbb{Z}_2^8$	$\mathbb{Z}_2^{28}$	$\mathbb{Z}_2^{16}$
Z_4		$\mathbb{Z}_2^4$	$\mathbb{Z}_2^6$	$\mathbb{Z}_2^{15} \times \mathbb{Z}_4$
Z_6		$\mathbb{Z}_2^4$	$\mathbb{Z}_2^6$	$\mathbb{Z}_2^{15} \times \mathbb{Z}_6$
$Z_2 \times Z_2$	$\mathbb{Z}_2^2$	$\mathbb{Z}_2^{12}$	$\mathbb{Z}_2^{20}$	$\mathbb{Z}_2^{22}$
$Z_4 \times Z_4$	$\mathbb{Z}_2^2$	$\mathbb{Z}_2^{12}$	$\mathbb{Z}_2^{20}$	$\mathbb{Z}_2^{20} \times \mathbb{Z}_4^2$
$Z_6 \times Z_6$	$\mathbb{Z}_2^2$	$\mathbb{Z}_2^{12}$	$\mathbb{Z}_2^{20}$	$\mathbb{Z}_2^{20} \times \mathbb{Z}_6^2$
$U(1)$		$\mathbb{Z}_2$		$\mathbb{Z} \times \mathbb{Z}_2^{15}$
$SU(2)$		$\mathbb{Z}_2$		$\mathbb{Z}_2^{10}$
$SO(3)$		$\mathbb{Z}_2$	$\mathbb{Z}_2^8$	$\mathbb{Z}_2^{10}$
Space group #49 *Pccm*				
$\varnothing$		$\mathbb{Z}_2$		$\mathbb{Z}_2^{16}$
Z_2^T	$\mathbb{Z}_2$	$\mathbb{Z}_2^2$	$\mathbb{Z}_2^{22}$	$\mathbb{Z}_2^{16}$
Z_2		$\mathbb{Z}_2^7$	$\mathbb{Z}_2^{11}$	$\mathbb{Z}_2^{24}$
$Z_2 \times Z_2^T$	$\mathbb{Z}_2^3$	$\mathbb{Z}_2^{14}$	$\mathbb{Z}_2^{44}$	$\mathbb{Z}_2^{24}$
Z_4		$\mathbb{Z}_2^7$	$\mathbb{Z}_2^{11}$	$\mathbb{Z}_2^{23} \times \mathbb{Z}_4$
Z_6		$\mathbb{Z}_2^7$	$\mathbb{Z}_2^{11}$	$\mathbb{Z}_2^{23} \times \mathbb{Z}_6$
$Z_2 \times Z_2$	$\mathbb{Z}_2^2$	$\mathbb{Z}_2^{19}$	$\mathbb{Z}_2^{33}$	$\mathbb{Z}_2^{32}$
$Z_4 \times Z_4$	$\mathbb{Z}_2^2$	$\mathbb{Z}_2^{19}$	$\mathbb{Z}_2^{33}$	$\mathbb{Z}_2^{30} \times \mathbb{Z}_4^2$
$Z_6 \times Z_6$	$\mathbb{Z}_2^2$	$\mathbb{Z}_2^{19}$	$\mathbb{Z}_2^{33}$	$\mathbb{Z}_2^{30} \times \mathbb{Z}_6^2$
$U(1)$		$\mathbb{Z}_2^3$		$\mathbb{Z} \times \mathbb{Z}_2^{23}$
$SU(2)$		$\mathbb{Z}_2^2$		$\mathbb{Z}_2^{16}$
$SO(3)$		$\mathbb{Z}_2^3$	$\mathbb{Z}_2^{11}$	$\mathbb{Z}_2^{16}$

TABLE II (Continued.)

G_0	$E^3_{3,\infty}$	$E^2_{2,\infty}$	$E^1_{1,\infty}$	$E^0_{0,\infty}$
Space group #50 *Pban*				
$\varnothing$				$\mathbb{Z}_2^{10}$
Z_2^T	$\mathbb{Z}_2$		$\mathbb{Z}_2^{14}$	$\mathbb{Z}_2^{10}$
Z_2		$\mathbb{Z}_2^4$	$\mathbb{Z}_2^6$	$\mathbb{Z}_2^{16}$
$Z_2 \times Z_2^T$	$\mathbb{Z}_2^3$	$\mathbb{Z}_2^8$	$\mathbb{Z}_2^{28}$	$\mathbb{Z}_2^{16}$
Z_4		$\mathbb{Z}_2^4$	$\mathbb{Z}_2^6$	$\mathbb{Z}_2^{15} \times \mathbb{Z}_4$
Z_6		$\mathbb{Z}_2^4$	$\mathbb{Z}_2^6$	$\mathbb{Z}_2^{15} \times \mathbb{Z}_6$
$Z_2 \times Z_2$	$\mathbb{Z}_2^2$	$\mathbb{Z}_2^{12}$	$\mathbb{Z}_2^{20}$	$\mathbb{Z}_2^{22}$
$Z_4 \times Z_4$	$\mathbb{Z}_2^2$	$\mathbb{Z}_2^{12}$	$\mathbb{Z}_2^{20}$	$\mathbb{Z}_2^{20} \times \mathbb{Z}_4^2$
$Z_6 \times Z_6$	$\mathbb{Z}_2^2$	$\mathbb{Z}_2^{12}$	$\mathbb{Z}_2^{20}$	$\mathbb{Z}_2^{20} \times \mathbb{Z}_6^2$
$U(1)$		$\mathbb{Z}_2$		$\mathbb{Z} \times \mathbb{Z}_2^{15}$
$SU(2)$		$\mathbb{Z}_2$		$\mathbb{Z}_2^{10}$
$SO(3)$		$\mathbb{Z}_2$	$\mathbb{Z}_2^8$	$\mathbb{Z}_2^{10}$
Space group #51 *Pmma*				
$\varnothing$		$\mathbb{Z}_2^3$	$\mathbb{Z}_2^2$	$\mathbb{Z}_2^{12}$
Z_2^T	$\mathbb{Z}_2$	$\mathbb{Z}_2^6$	$\mathbb{Z}_2^{22}$	$\mathbb{Z}_2^{12}$
Z_2		$\mathbb{Z}_2^{11}$	$\mathbb{Z}_2^{13}$	$\mathbb{Z}_2^{18}$
$Z_2 \times Z_2^T$	$\mathbb{Z}_2^3$	$\mathbb{Z}_2^{22}$	$\mathbb{Z}_2^{42}$	$\mathbb{Z}_2^{18}$
Z_4		$\mathbb{Z}_2^{11}$	$\mathbb{Z}_2^{13}$	$\mathbb{Z}_2^{17} \times \mathbb{Z}_4$
Z_6		$\mathbb{Z}_2^{11}$	$\mathbb{Z}_2^{13}$	$\mathbb{Z}_2^{17} \times \mathbb{Z}_6$
$Z_2 \times Z_2$	$\mathbb{Z}_2^2$	$\mathbb{Z}_2^{27}$	$\mathbb{Z}_2^{33}$	$\mathbb{Z}_2^{24}$
$Z_4 \times Z_4$	$\mathbb{Z}_2^2$	$\mathbb{Z}_2^{27}$	$\mathbb{Z}_2^{33}$	$\mathbb{Z}_2^{22} \times \mathbb{Z}_4^2$
$Z_6 \times Z_6$	$\mathbb{Z}_2^2$	$\mathbb{Z}_2^{27}$	$\mathbb{Z}_2^{33}$	$\mathbb{Z}_2^{22} \times \mathbb{Z}_6^2$
$U(1)$		$\mathbb{Z}_2^7$	$\mathbb{Z}_2^2$	$\mathbb{Z} \times \mathbb{Z}_2^{17}$
$SU(2)$		$\mathbb{Z}_2^4$	$\mathbb{Z}_2^2$	$\mathbb{Z}_2^{12}$
$SO(3)$		$\mathbb{Z}_2^7$	$\mathbb{Z}_2^{11}$	$\mathbb{Z}_2^{12}$
Space group #52 *Pnna*				
$\varnothing$				$\mathbb{Z}_2^4$
Z_2^T	$\mathbb{Z}_2$		$\mathbb{Z}_2^7$	$\mathbb{Z}_2^4$
Z_2		$\mathbb{Z}_2^3$	$\mathbb{Z}_2^2$	$\mathbb{Z}_2^8$
$Z_2 \times Z_2^T$	$\mathbb{Z}_2^3$	$\mathbb{Z}_2^6$	$\mathbb{Z}_2^{14}$	$\mathbb{Z}_2^8$
Z_4		$\mathbb{Z}_2^3$	$\mathbb{Z}_2^2$	$\mathbb{Z}_2^7 \times \mathbb{Z}_4$
Z_6		$\mathbb{Z}_2^3$	$\mathbb{Z}_2^2$	$\mathbb{Z}_2^7 \times \mathbb{Z}_6$
$Z_2 \times Z_2$	$\mathbb{Z}_2^2$	$\mathbb{Z}_2^9$	$\mathbb{Z}_2^9$	$\mathbb{Z}_2^{12}$
$Z_4 \times Z_4$	$\mathbb{Z}_2^2$	$\mathbb{Z}_2^9$	$\mathbb{Z}_2^9$	$\mathbb{Z}_2^{10} \times \mathbb{Z}_4^2$
$Z_6 \times Z_6$	$\mathbb{Z}_2^2$	$\mathbb{Z}_2^9$	$\mathbb{Z}_2^9$	$\mathbb{Z}_2^{10} \times \mathbb{Z}_6^2$
$U(1)$		$\mathbb{Z}_2$		$\mathbb{Z} \times \mathbb{Z}_2^7$
$SU(2)$		$\mathbb{Z}_2$		$\mathbb{Z}_2^4$
$SO(3)$		$\mathbb{Z}_2$	$\mathbb{Z}_2^5$	$\mathbb{Z}_2^4$
Space group #53 *Pmna*				
$\varnothing$		$\mathbb{Z}_2$		$\mathbb{Z}_2^9$
Z_2^T	$\mathbb{Z}_2$	$\mathbb{Z}_2^2$	$\mathbb{Z}_2^{13}$	$\mathbb{Z}_2^9$
Z_2		$\mathbb{Z}_2^6$	$\mathbb{Z}_2^6$	$\mathbb{Z}_2^{14}$
$Z_2 \times Z_2^T$	$\mathbb{Z}_2^3$	$\mathbb{Z}_2^{12}$	$\mathbb{Z}_2^{26}$	$\mathbb{Z}_2^{14}$
Z_4		$\mathbb{Z}_2^6$	$\mathbb{Z}_2^6$	$\mathbb{Z}_2^{13} \times \mathbb{Z}_4$
Z_6		$\mathbb{Z}_2^6$	$\mathbb{Z}_2^6$	$\mathbb{Z}_2^{13} \times \mathbb{Z}_6$
$Z_2 \times Z_2$	$\mathbb{Z}_2^2$	$\mathbb{Z}_2^{16}$	$\mathbb{Z}_2^{19}$	$\mathbb{Z}_2^{19}$
$Z_4 \times Z_4$	$\mathbb{Z}_2^2$	$\mathbb{Z}_2^{16}$	$\mathbb{Z}_2^{19}$	$\mathbb{Z}_2^{17} \times \mathbb{Z}_4^2$
$Z_6 \times Z_6$	$\mathbb{Z}_2^2$	$\mathbb{Z}_2^{16}$	$\mathbb{Z}_2^{19}$	$\mathbb{Z}_2^{17} \times \mathbb{Z}_6^2$

TABLE II (Continued.)

G_0	$E^3_{3,\infty}$	$E^2_{2,\infty}$	$E^1_{1,\infty}$	$E^0_{0,\infty}$
$U(1)$		$\mathbb{Z}_2^3$		$\mathbb{Z}\times\mathbb{Z}_2^{13}$
$SU(2)$		$\mathbb{Z}_2^2$		$\mathbb{Z}_2^9$
$SO(3)$		$\mathbb{Z}_2^3$	$\mathbb{Z}_2^7$	$\mathbb{Z}_2^9$
Space group #54 $Pcca$				
$\varnothing$				$\mathbb{Z}_2^5$
Z_2^T	$\mathbb{Z}_2$		$\mathbb{Z}_2^{10}$	$\mathbb{Z}_2^5$
Z_2		$\mathbb{Z}_2^4$	$\mathbb{Z}_2^3$	$\mathbb{Z}_2^{10}$
$Z_2\times Z_2^T$	$\mathbb{Z}_2^3$	$\mathbb{Z}_2^8$	$\mathbb{Z}_2^{20}$	$\mathbb{Z}_2^{10}$
Z_4		$\mathbb{Z}_2^4$	$\mathbb{Z}_2^3$	$\mathbb{Z}_2^9\times\mathbb{Z}_4$
Z_6		$\mathbb{Z}_2^4$	$\mathbb{Z}_2^3$	$\mathbb{Z}_2^9\times\mathbb{Z}_6$
$Z_2\times Z_2$	$\mathbb{Z}_2^2$	$\mathbb{Z}_2^{12}$	$\mathbb{Z}_2^{13}$	$\mathbb{Z}_2^{15}$
$Z_4\times Z_4$	$\mathbb{Z}_2^2$	$\mathbb{Z}_2^{12}$	$\mathbb{Z}_2^{13}$	$\mathbb{Z}_2^{13}\times\mathbb{Z}_4^2$
$Z_6\times Z_6$	$\mathbb{Z}_2^2$	$\mathbb{Z}_2^{12}$	$\mathbb{Z}_2^{13}$	$\mathbb{Z}_2^{13}\times\mathbb{Z}_6^2$
$U(1)$		$\mathbb{Z}_2$		$\mathbb{Z}\times\mathbb{Z}_2^9$
$SU(2)$		$\mathbb{Z}_2$		$\mathbb{Z}_2^5$
$SO(3)$		$\mathbb{Z}_2$	$\mathbb{Z}_2^7$	$\mathbb{Z}_2^5$
Space group #55 $Pbam$				
$\varnothing$		$\mathbb{Z}_2^2$		$\mathbb{Z}_2^8$
Z_2^T	$\mathbb{Z}_2$	$\mathbb{Z}_2^4$	$\mathbb{Z}_2^{12}$	$\mathbb{Z}_2^8$
Z_2		$\mathbb{Z}_2^8$	$\mathbb{Z}_2^6$	$\mathbb{Z}_2^{12}$
$Z_2\times Z_2^T$	$\mathbb{Z}_2^3$	$\mathbb{Z}_2^{16}$	$\mathbb{Z}_2^{24}$	$\mathbb{Z}_2^{12}$
Z_4		$\mathbb{Z}_2^8$	$\mathbb{Z}_2^6$	$\mathbb{Z}_2^{11}\times\mathbb{Z}_4$
Z_6		$\mathbb{Z}_2^8$	$\mathbb{Z}_2^6$	$\mathbb{Z}_2^{11}\times\mathbb{Z}_6$
$Z_2\times Z_2$	$\mathbb{Z}_2^2$	$\mathbb{Z}_2^{20}$	$\mathbb{Z}_2^{18}$	$\mathbb{Z}_2^{16}$
$Z_4\times Z_4$	$\mathbb{Z}_2^2$	$\mathbb{Z}_2^{20}$	$\mathbb{Z}_2^{18}$	$\mathbb{Z}_2^{14}\times\mathbb{Z}_4^2$
$Z_6\times Z_6$	$\mathbb{Z}_2^2$	$\mathbb{Z}_2^{20}$	$\mathbb{Z}_2^{18}$	$\mathbb{Z}_2^{14}\times\mathbb{Z}_6^2$
$U(1)$		$\mathbb{Z}_2^5$		$\mathbb{Z}\times\mathbb{Z}_2^{11}$
$SU(2)$		$\mathbb{Z}_2^3$		$\mathbb{Z}_2^8$
$SO(3)$		$\mathbb{Z}_2^5$	$\mathbb{Z}_2^6$	$\mathbb{Z}_2^8$
Space group #56 $Pccn$				
$\varnothing$				$\mathbb{Z}_2^4$
Z_2^T	$\mathbb{Z}_2$		$\mathbb{Z}_2^7$	$\mathbb{Z}_2^4$
Z_2		$\mathbb{Z}_2^3$	$\mathbb{Z}_2^2$	$\mathbb{Z}_2^8$
$Z_2\times Z_2^T$	$\mathbb{Z}_2^3$	$\mathbb{Z}_2^6$	$\mathbb{Z}_2^{14}$	$\mathbb{Z}_2^8$
Z_4		$\mathbb{Z}_2^2\times\mathbb{Z}_4$	$\mathbb{Z}_2^2$	$\mathbb{Z}_2^7\times\mathbb{Z}_4$
Z_6		$\mathbb{Z}_2^3$	$\mathbb{Z}_2^2$	$\mathbb{Z}_2^7\times\mathbb{Z}_6$
$Z_2\times Z_2$	$\mathbb{Z}_2^2$	$\mathbb{Z}_2^9$	$\mathbb{Z}_2^9$	$\mathbb{Z}_2^{12}$
$Z_4\times Z_4$	$\mathbb{Z}_2^2$	$\mathbb{Z}_2^6\times\mathbb{Z}_4^3$	$\mathbb{Z}_2^8\times\mathbb{Z}_4$	$\mathbb{Z}_2^{10}\times\mathbb{Z}_4^2$
$Z_6\times Z_6$	$\mathbb{Z}_2^2$	$\mathbb{Z}_2^9$	$\mathbb{Z}_2^9$	$\mathbb{Z}_2^{10}\times\mathbb{Z}_6^2$
$U(1)$		$\mathbb{Z}_2$		$\mathbb{Z}\times\mathbb{Z}_2^7$
$SU(2)$		$\mathbb{Z}_2$		$\mathbb{Z}_2^4$
$SO(3)$		$\mathbb{Z}_2$	$\mathbb{Z}_2^5$	$\mathbb{Z}_2^4$
Space group #57 $Pbcm$				
$\varnothing$		$\mathbb{Z}_2$		$\mathbb{Z}_2^4$
Z_2^T	$\mathbb{Z}_2$	$\mathbb{Z}_2^2$	$\mathbb{Z}_2^9$	$\mathbb{Z}_2^4$
Z_2		$\mathbb{Z}_2^6$	$\mathbb{Z}_2^3$	$\mathbb{Z}_2^8$
$Z_2\times Z_2^T$	$\mathbb{Z}_2^3$	$\mathbb{Z}_2^{12}$	$\mathbb{Z}_2^{18}$	$\mathbb{Z}_2^8$
Z_4		$\mathbb{Z}_2^6$	$\mathbb{Z}_2^3$	$\mathbb{Z}_2^7\times\mathbb{Z}_4$
Z_6		$\mathbb{Z}_2^6$	$\mathbb{Z}_2^3$	$\mathbb{Z}_2^7\times\mathbb{Z}_6$

TABLE II (Continued.)

G_0	$E^3_{3,\infty}$	$E^2_{2,\infty}$	$E^1_{1,\infty}$	$E^0_{0,\infty}$
$Z_2\times Z_2$	$\mathbb{Z}_2^2$	$\mathbb{Z}_2^{16}$	$\mathbb{Z}_2^{12}$	$\mathbb{Z}_2^{12}$
$Z_4\times Z_4$	$\mathbb{Z}_2^2$	$\mathbb{Z}_2^{16}$	$\mathbb{Z}_2^{12}$	$\mathbb{Z}_2^{10}\times\mathbb{Z}_4^2$
$Z_6\times Z_6$	$\mathbb{Z}_2^2$	$\mathbb{Z}_2^{16}$	$\mathbb{Z}_2^{12}$	$\mathbb{Z}_2^{10}\times\mathbb{Z}_6^2$
$U(1)$		$\mathbb{Z}_2^3$		$\mathbb{Z}\times\mathbb{Z}_2^7$
$SU(2)$		$\mathbb{Z}_2^2$		$\mathbb{Z}_2^4$
$SO(3)$		$\mathbb{Z}_2^3$	$\mathbb{Z}_2^6$	$\mathbb{Z}_2^4$
Space group #58 $Pnnm$				
$\varnothing$		$\mathbb{Z}_2$		$\mathbb{Z}_2^8$
Z_2^T	$\mathbb{Z}_2$	$\mathbb{Z}_2^2$	$\mathbb{Z}_2^{10}$	$\mathbb{Z}_2^8$
Z_2		$\mathbb{Z}_2^5$	$\mathbb{Z}_2^5$	$\mathbb{Z}_2^{12}$
$Z_2\times Z_2^T$	$\mathbb{Z}_2^3$	$\mathbb{Z}_2^{10}$	$\mathbb{Z}_2^{20}$	$\mathbb{Z}_2^{12}$
Z_4		$\mathbb{Z}_2^4\times\mathbb{Z}_4$	$\mathbb{Z}_2^5$	$\mathbb{Z}_2^{11}\times\mathbb{Z}_4$
Z_6		$\mathbb{Z}_2^5$	$\mathbb{Z}_2^5$	$\mathbb{Z}_2^{11}\times\mathbb{Z}_6$
$Z_2\times Z_2$	$\mathbb{Z}_2^2$	$\mathbb{Z}_2^{13}$	$\mathbb{Z}_2^{15}$	$\mathbb{Z}_2^{16}$
$Z_4\times Z_4$	$\mathbb{Z}_2^2$	$\mathbb{Z}_2^{10}\times\mathbb{Z}_4^3$	$\mathbb{Z}_2^{14}\times\mathbb{Z}_4$	$\mathbb{Z}_2^{14}\times\mathbb{Z}_4^2$
$Z_6\times Z_6$	$\mathbb{Z}_2^2$	$\mathbb{Z}_2^{13}$	$\mathbb{Z}_2^{15}$	$\mathbb{Z}_2^{14}\times\mathbb{Z}_6^2$
$U(1)$		$\mathbb{Z}_2^3$		$\mathbb{Z}\times\mathbb{Z}_2^{11}$
$SU(2)$		$\mathbb{Z}_2^2$		$\mathbb{Z}_2^8$
$SO(3)$		$\mathbb{Z}_2^3$	$\mathbb{Z}_2^5$	$\mathbb{Z}_2^8$
Space group #59 $Pmmn$				
$\varnothing$		$\mathbb{Z}_2^2$	$\mathbb{Z}_2^2$	$\mathbb{Z}_2^6$
Z_2^T	$\mathbb{Z}_2$	$\mathbb{Z}_2^4$	$\mathbb{Z}_2^{14}$	$\mathbb{Z}_2^6$
Z_2		$\mathbb{Z}_2^8$	$\mathbb{Z}_2^8$	$\mathbb{Z}_2^{10}$
$Z_2\times Z_2^T$	$\mathbb{Z}_2^3$	$\mathbb{Z}_2^{16}$	$\mathbb{Z}_2^{26}$	$\mathbb{Z}_2^{10}$
Z_4		$\mathbb{Z}_2^7\times\mathbb{Z}_4$	$\mathbb{Z}_2^8$	$\mathbb{Z}_2^9\times\mathbb{Z}_4$
Z_6		$\mathbb{Z}_2^8$	$\mathbb{Z}_2^8$	$\mathbb{Z}_2^9\times\mathbb{Z}_6$
$Z_2\times Z_2$	$\mathbb{Z}_2^2$	$\mathbb{Z}_2^{20}$	$\mathbb{Z}_2^{20}$	$\mathbb{Z}_2^{14}$
$Z_4\times Z_4$	$\mathbb{Z}_2^2$	$\mathbb{Z}_2^{17}\times\mathbb{Z}_4^3$	$\mathbb{Z}_2^{19}\times\mathbb{Z}_4$	$\mathbb{Z}_2^{12}\times\mathbb{Z}_4^2$
$Z_6\times Z_6$	$\mathbb{Z}_2^2$	$\mathbb{Z}_2^{20}$	$\mathbb{Z}_2^{20}$	$\mathbb{Z}_2^{12}\times\mathbb{Z}_6^2$
$U(1)$		$\mathbb{Z}_2^5$	$\mathbb{Z}_2^2$	$\mathbb{Z}\times\mathbb{Z}_2^9$
$SU(2)$		$\mathbb{Z}_2^3$	$\mathbb{Z}_2^2$	$\mathbb{Z}_2^6$
$SO(3)$		$\mathbb{Z}_2^5$	$\mathbb{Z}_2^8$	$\mathbb{Z}_2^6$
Space group #60 $Pbcn$				
$\varnothing$				$\mathbb{Z}_2^3$
Z_2^T	$\mathbb{Z}_2$		$\mathbb{Z}_2^5$	$\mathbb{Z}_2^3$
Z_2		$\mathbb{Z}_2^3$	$\mathbb{Z}_2$	$\mathbb{Z}_2^6$
$Z_2\times Z_2^T$	$\mathbb{Z}_2^3$	$\mathbb{Z}_2^6$	$\mathbb{Z}_2^{10}$	$\mathbb{Z}_2^6$
Z_4		$\mathbb{Z}_2^3$	$\mathbb{Z}_2$	$\mathbb{Z}_2^5\times\mathbb{Z}_4$
Z_6		$\mathbb{Z}_2^3$	$\mathbb{Z}_2$	$\mathbb{Z}_2^5\times\mathbb{Z}_6$
$Z_2\times Z_2$	$\mathbb{Z}_2^2$	$\mathbb{Z}_2^9$	$\mathbb{Z}_2^6$	$\mathbb{Z}_2^9$
$Z_4\times Z_4$	$\mathbb{Z}_2^2$	$\mathbb{Z}_2^9$	$\mathbb{Z}_2^6$	$\mathbb{Z}_2^7\times\mathbb{Z}_4^2$
$Z_6\times Z_6$	$\mathbb{Z}_2^2$	$\mathbb{Z}_2^9$	$\mathbb{Z}_2^6$	$\mathbb{Z}_2^7\times\mathbb{Z}_6^2$
$U(1)$		$\mathbb{Z}_2$		$\mathbb{Z}\times\mathbb{Z}_2^5$
$SU(2)$		$\mathbb{Z}_2$		$\mathbb{Z}_2^3$
$SO(3)$		$\mathbb{Z}_2$	$\mathbb{Z}_2^4$	$\mathbb{Z}_2^3$
Space group #61 $Pbca$				
$\varnothing$				$\mathbb{Z}_2^2$
Z_2^T	$\mathbb{Z}_2$		$\mathbb{Z}_2^3$	$\mathbb{Z}_2^2$
Z_2		$\mathbb{Z}_2^3$		$\mathbb{Z}_2^4$

TABLE II (Continued.)

G_0	$E^3_{3,\infty}$	$E^2_{2,\infty}$	$E^1_{1,\infty}$	$E^0_{0,\infty}$
$Z_2 \times Z_2^T$	$\mathbb{Z}_2^3$	$\mathbb{Z}_2^6$	$\mathbb{Z}_2^6$	$\mathbb{Z}_2^4$
Z_4		$\mathbb{Z}_2^3$		$\mathbb{Z}_2^3 \times \mathbb{Z}_4$
Z_6		$\mathbb{Z}_2^3$		$\mathbb{Z}_2^3 \times \mathbb{Z}_6$
$Z_2 \times Z_2$	$\mathbb{Z}_2^2$	$\mathbb{Z}_2^9$	$\mathbb{Z}_2^3$	$\mathbb{Z}_2^6$
$Z_4 \times Z_4$	$\mathbb{Z}_2^2$	$\mathbb{Z}_2^9$	$\mathbb{Z}_2^3$	$\mathbb{Z}_2^4 \times \mathbb{Z}_4^2$
$Z_6 \times Z_6$	$\mathbb{Z}_2^2$	$\mathbb{Z}_2^9$	$\mathbb{Z}_2^3$	$\mathbb{Z}_2^4 \times \mathbb{Z}_6^2$
$U(1)$		$\mathbb{Z}_2$		$\mathbb{Z} \times \mathbb{Z}_2^3$
$SU(2)$		$\mathbb{Z}_2$		$\mathbb{Z}_2^2$
$SO(3)$		$\mathbb{Z}_2$	$\mathbb{Z}_2^3$	$\mathbb{Z}_2^2$
Space group #62 $Pnma$				
$\varnothing$		$\mathbb{Z}_2$		$\mathbb{Z}_2^3$
Z_2^T	$\mathbb{Z}_2$	$\mathbb{Z}_2^2$	$\mathbb{Z}_2^6$	$\mathbb{Z}_2^3$
Z_2		$\mathbb{Z}_2^5$	$\mathbb{Z}_2^2$	$\mathbb{Z}_2^6$
$Z_2 \times Z_2^T$	$\mathbb{Z}_2^3$	$\mathbb{Z}_2^{10}$	$\mathbb{Z}_2^{12}$	$\mathbb{Z}_2^6$
Z_4		$\mathbb{Z}_2^4 \times \mathbb{Z}_4$	$\mathbb{Z}_2^2$	$\mathbb{Z}_2^5 \times \mathbb{Z}_4$
Z_6		$\mathbb{Z}_2^5$	$\mathbb{Z}_2^2$	$\mathbb{Z}_2^5 \times \mathbb{Z}_6$
$Z_2 \times Z_2$	$\mathbb{Z}_2^2$	$\mathbb{Z}_2^{13}$	$\mathbb{Z}_2^8$	$\mathbb{Z}_2^9$
$Z_4 \times Z_4$	$\mathbb{Z}_2^2$	$\mathbb{Z}_2^{10} \times \mathbb{Z}_4^3$	$\mathbb{Z}_2^7 \times \mathbb{Z}_4$	$\mathbb{Z}_2^7 \times \mathbb{Z}_4^2$
$Z_6 \times Z_6$	$\mathbb{Z}_2^2$	$\mathbb{Z}_2^{13}$	$\mathbb{Z}_2^8$	$\mathbb{Z}_2^7 \times \mathbb{Z}_6^2$
$U(1)$		$\mathbb{Z}_2^3$		$\mathbb{Z} \times \mathbb{Z}_2^5$
$SU(2)$		$\mathbb{Z}_2^2$		$\mathbb{Z}_2^3$
$SO(3)$		$\mathbb{Z}_2^3$	$\mathbb{Z}_2^4$	$\mathbb{Z}_2^3$
Space group #63 $Cmcm$				
$\varnothing$		$\mathbb{Z}_2^2$	$\mathbb{Z}_2$	$\mathbb{Z}_2^7$
Z_2^T	$\mathbb{Z}_2$	$\mathbb{Z}_2^4$	$\mathbb{Z}_2^{13}$	$\mathbb{Z}_2^7$
Z_2		$\mathbb{Z}_2^8$	$\mathbb{Z}_2^7$	$\mathbb{Z}_2^{11}$
$Z_2 \times Z_2^T$	$\mathbb{Z}_2^3$	$\mathbb{Z}_2^{16}$	$\mathbb{Z}_2^{25}$	$\mathbb{Z}_2^{11}$
Z_4		$\mathbb{Z}_2^8$	$\mathbb{Z}_2^7$	$\mathbb{Z}_2^{10} \times \mathbb{Z}_4$
Z_6		$\mathbb{Z}_2^8$	$\mathbb{Z}_2^7$	$\mathbb{Z}_2^{10} \times \mathbb{Z}_6$
$Z_2 \times Z_2$	$\mathbb{Z}_2^2$	$\mathbb{Z}_2^{20}$	$\mathbb{Z}_2^{19}$	$\mathbb{Z}_2^{15}$
$Z_4 \times Z_4$	$\mathbb{Z}_2^2$	$\mathbb{Z}_2^{20}$	$\mathbb{Z}_2^{19}$	$\mathbb{Z}_2^{13} \times \mathbb{Z}_4^2$
$Z_6 \times Z_6$	$\mathbb{Z}_2^2$	$\mathbb{Z}_2^{20}$	$\mathbb{Z}_2^{19}$	$\mathbb{Z}_2^{13} \times \mathbb{Z}_6^2$
$U(1)$		$\mathbb{Z}_2^5$	$\mathbb{Z}_2$	$\mathbb{Z} \times \mathbb{Z}_2^{10}$
$SU(2)$		$\mathbb{Z}_2^3$	$\mathbb{Z}_2$	$\mathbb{Z}_2^7$
$SO(3)$		$\mathbb{Z}_2^5$	$\mathbb{Z}_2^7$	$\mathbb{Z}_2^7$
Space group #64 $Cmce$				
$\varnothing$		$\mathbb{Z}_2$		$\mathbb{Z}_2^6$
Z_2^T	$\mathbb{Z}_2$	$\mathbb{Z}_2^2$	$\mathbb{Z}_2^{10}$	$\mathbb{Z}_2^6$
Z_2		$\mathbb{Z}_2^6$	$\mathbb{Z}_2^4$	$\mathbb{Z}_2^{10}$
$Z_2 \times Z_2^T$	$\mathbb{Z}_2^3$	$\mathbb{Z}_2^{12}$	$\mathbb{Z}_2^{20}$	$\mathbb{Z}_2^{10}$
Z_4		$\mathbb{Z}_2^6$	$\mathbb{Z}_2^4$	$\mathbb{Z}_2^9 \times \mathbb{Z}_4$
Z_6		$\mathbb{Z}_2^6$	$\mathbb{Z}_2^4$	$\mathbb{Z}_2^9 \times \mathbb{Z}_6$
$Z_2 \times Z_2$	$\mathbb{Z}_2^2$	$\mathbb{Z}_2^{16}$	$\mathbb{Z}_2^{14}$	$\mathbb{Z}_2^{14}$
$Z_4 \times Z_4$	$\mathbb{Z}_2^2$	$\mathbb{Z}_2^{16}$	$\mathbb{Z}_2^{14}$	$\mathbb{Z}_2^{12} \times \mathbb{Z}_4^2$
$Z_6 \times Z_6$	$\mathbb{Z}_2^2$	$\mathbb{Z}_2^{16}$	$\mathbb{Z}_2^{14}$	$\mathbb{Z}_2^{12} \times \mathbb{Z}_6^2$
$U(1)$		$\mathbb{Z}_2^3$		$\mathbb{Z} \times \mathbb{Z}_2^9$
$SU(2)$		$\mathbb{Z}_2^2$		$\mathbb{Z}_2^6$
$SO(3)$		$\mathbb{Z}_2^3$	$\mathbb{Z}_2^6$	$\mathbb{Z}_2^6$
Space group #65 $Cmmm$				

TABLE II (Continued.)

G_0	$E^3_{3,\infty}$	$E^2_{2,\infty}$	$E^1_{1,\infty}$	$E^0_{0,\infty}$
$\varnothing$		$\mathbb{Z}_2^4$	$\mathbb{Z}_2^6$	$\mathbb{Z}_2^{16}$
Z_2^T	$\mathbb{Z}_2$	$\mathbb{Z}_2^8$	$\mathbb{Z}_2^{30}$	$\mathbb{Z}_2^{16}$
Z_2		$\mathbb{Z}_2^{13}$	$\mathbb{Z}_2^{21}$	$\mathbb{Z}_2^{22}$
$Z_2 \times Z_2^T$	$\mathbb{Z}_2^3$	$\mathbb{Z}_2^{26}$	$\mathbb{Z}_2^{54}$	$\mathbb{Z}_2^{22}$
Z_4		$\mathbb{Z}_2^{13}$	$\mathbb{Z}_2^{21}$	$\mathbb{Z}_2^{21} \times \mathbb{Z}_4$
Z_6		$\mathbb{Z}_2^{13}$	$\mathbb{Z}_2^{21}$	$\mathbb{Z}_2^{21} \times \mathbb{Z}_6$
$Z_2 \times Z_2$	$\mathbb{Z}_2^2$	$\mathbb{Z}_2^{31}$	$\mathbb{Z}_2^{45}$	$\mathbb{Z}_2^{28}$
$Z_4 \times Z_4$	$\mathbb{Z}_2^2$	$\mathbb{Z}_2^{31}$	$\mathbb{Z}_2^{45}$	$\mathbb{Z}_2^{26} \times \mathbb{Z}_4^2$
$Z_6 \times Z_6$	$\mathbb{Z}_2^2$	$\mathbb{Z}_2^{31}$	$\mathbb{Z}_2^{45}$	$\mathbb{Z}_2^{26} \times \mathbb{Z}_6^2$
$U(1)$		$\mathbb{Z}_2^9$	$\mathbb{Z}_2^6$	$\mathbb{Z} \times \mathbb{Z}_2^{21}$
$SU(2)$		$\mathbb{Z}_2^5$	$\mathbb{Z}_2^6$	$\mathbb{Z}_2^{16}$
$SO(3)$		$\mathbb{Z}_2^9$	$\mathbb{Z}_2^{15}$	$\mathbb{Z}_2^{16}$
Space group #66 $Cccm$				
$\varnothing$		$\mathbb{Z}_2$		$\mathbb{Z}_2^{12}$
Z_2^T	$\mathbb{Z}_2$	$\mathbb{Z}_2^2$	$\mathbb{Z}_2^{16}$	$\mathbb{Z}_2^{12}$
Z_2		$\mathbb{Z}_2^6$	$\mathbb{Z}_2^8$	$\mathbb{Z}_2^{18}$
$Z_2 \times Z_2^T$	$\mathbb{Z}_2^3$	$\mathbb{Z}_2^{12}$	$\mathbb{Z}_2^{32}$	$\mathbb{Z}_2^{18}$
Z_4		$\mathbb{Z}_2^6$	$\mathbb{Z}_2^8$	$\mathbb{Z}_2^{17} \times \mathbb{Z}_4$
Z_6		$\mathbb{Z}_2^6$	$\mathbb{Z}_2^8$	$\mathbb{Z}_2^{17} \times \mathbb{Z}_6$
$Z_2 \times Z_2$	$\mathbb{Z}_2^2$	$\mathbb{Z}_2^{16}$	$\mathbb{Z}_2^{24}$	$\mathbb{Z}_2^{24}$
$Z_4 \times Z_4$	$\mathbb{Z}_2^2$	$\mathbb{Z}_2^{16}$	$\mathbb{Z}_2^{24}$	$\mathbb{Z}_2^{22} \times \mathbb{Z}_4^2$
$Z_6 \times Z_6$	$\mathbb{Z}_2^2$	$\mathbb{Z}_2^{16}$	$\mathbb{Z}_2^{24}$	$\mathbb{Z}_2^{22} \times \mathbb{Z}_6^2$
$U(1)$		$\mathbb{Z}_2^3$		$\mathbb{Z} \times \mathbb{Z}_2^{17}$
$SU(2)$		$\mathbb{Z}_2^2$		$\mathbb{Z}_2^{12}$
$SO(3)$		$\mathbb{Z}_2^3$	$\mathbb{Z}_2^8$	$\mathbb{Z}_2^{12}$
Space group #67 $Cmme$				
$\varnothing$		$\mathbb{Z}_2^2$	$\mathbb{Z}_2$	$\mathbb{Z}_2^{14}$
Z_2^T	$\mathbb{Z}_2$	$\mathbb{Z}_2^4$	$\mathbb{Z}_2^{22}$	$\mathbb{Z}_2^{14}$
Z_2		$\mathbb{Z}_2^9$	$\mathbb{Z}_2^{12}$	$\mathbb{Z}_2^{21}$
$Z_2 \times Z_2^T$	$\mathbb{Z}_2^3$	$\mathbb{Z}_2^{18}$	$\mathbb{Z}_2^{43}$	$\mathbb{Z}_2^{21}$
Z_4		$\mathbb{Z}_2^9$	$\mathbb{Z}_2^{12}$	$\mathbb{Z}_2^{20} \times \mathbb{Z}_4$
Z_6		$\mathbb{Z}_2^9$	$\mathbb{Z}_2^{12}$	$\mathbb{Z}_2^{20} \times \mathbb{Z}_6$
$Z_2 \times Z_2$	$\mathbb{Z}_2^2$	$\mathbb{Z}_2^{23}$	$\mathbb{Z}_2^{33}$	$\mathbb{Z}_2^{28}$
$Z_4 \times Z_4$	$\mathbb{Z}_2^2$	$\mathbb{Z}_2^{23}$	$\mathbb{Z}_2^{33}$	$\mathbb{Z}_2^{26} \times \mathbb{Z}_4^2$
$Z_6 \times Z_6$	$\mathbb{Z}_2^2$	$\mathbb{Z}_2^{23}$	$\mathbb{Z}_2^{33}$	$\mathbb{Z}_2^{26} \times \mathbb{Z}_6^2$
$U(1)$		$\mathbb{Z}_2^5$	$\mathbb{Z}_2$	$\mathbb{Z} \times \mathbb{Z}_2^{20}$
$SU(2)$		$\mathbb{Z}_2^3$	$\mathbb{Z}_2$	$\mathbb{Z}_2^{14}$
$SO(3)$		$\mathbb{Z}_2^5$	$\mathbb{Z}_2^{11}$	$\mathbb{Z}_2^{14}$
Space group #68 $Ccce$				
$\varnothing$				$\mathbb{Z}_2^7$
Z_2^T	$\mathbb{Z}_2$		$\mathbb{Z}_2^{11}$	$\mathbb{Z}_2^7$
Z_2		$\mathbb{Z}_2^4$	$\mathbb{Z}_2^4$	$\mathbb{Z}_2^{12}$
$Z_2 \times Z_2^T$	$\mathbb{Z}_2^3$	$\mathbb{Z}_2^8$	$\mathbb{Z}_2^{22}$	$\mathbb{Z}_2^{12}$
Z_4		$\mathbb{Z}_2^4$	$\mathbb{Z}_2^4$	$\mathbb{Z}_2^{11} \times \mathbb{Z}_4$
Z_6		$\mathbb{Z}_2^4$	$\mathbb{Z}_2^4$	$\mathbb{Z}_2^{11} \times \mathbb{Z}_6$
$Z_2 \times Z_2$	$\mathbb{Z}_2^2$	$\mathbb{Z}_2^{12}$	$\mathbb{Z}_2^{15}$	$\mathbb{Z}_2^{17}$
$Z_4 \times Z_4$	$\mathbb{Z}_2^2$	$\mathbb{Z}_2^{12}$	$\mathbb{Z}_2^{15}$	$\mathbb{Z}_2^{15} \times \mathbb{Z}_4^2$
$Z_6 \times Z_6$	$\mathbb{Z}_2^2$	$\mathbb{Z}_2^{12}$	$\mathbb{Z}_2^{15}$	$\mathbb{Z}_2^{15} \times \mathbb{Z}_6^2$
$U(1)$		$\mathbb{Z}_2$		$\mathbb{Z} \times \mathbb{Z}_2^{11}$

TABLE II (Continued.)

G_0	$E^3_{3,\infty}$	$E^2_{2,\infty}$	$E^1_{1,\infty}$	$E^0_{0,\infty}$
$SU(2)$		$\mathbb{Z}_2$		$\mathbb{Z}_2^7$
$SO(3)$		$\mathbb{Z}_2$	$\mathbb{Z}_2^7$	$\mathbb{Z}_2^7$
Space group #69 $Fmmm$				
$\varnothing$		$\mathbb{Z}_2^3$	$\mathbb{Z}_2^3$	$\mathbb{Z}_2^{14}$
Z_2^T	$\mathbb{Z}_2$	$\mathbb{Z}_2^6$	$\mathbb{Z}_2^{24}$	$\mathbb{Z}_2^{14}$
Z_2		$\mathbb{Z}_2^{11}$	$\mathbb{Z}_2^{15}$	$\mathbb{Z}_2^{20}$
$Z_2\times Z_2^T$	$\mathbb{Z}_2^3$	$\mathbb{Z}_2^{22}$	$\mathbb{Z}_2^{45}$	$\mathbb{Z}_2^{20}$
Z_4		$\mathbb{Z}_2^{11}$	$\mathbb{Z}_2^{15}$	$\mathbb{Z}_2^{19}\times\mathbb{Z}_4$
Z_6		$\mathbb{Z}_2^{11}$	$\mathbb{Z}_2^{15}$	$\mathbb{Z}_2^{19}\times\mathbb{Z}_6$
$Z_2\times Z_2$	$\mathbb{Z}_2^2$	$\mathbb{Z}_2^{27}$	$\mathbb{Z}_2^{36}$	$\mathbb{Z}_2^{26}$
$Z_4\times Z_4$	$\mathbb{Z}_2^2$	$\mathbb{Z}_2^{27}$	$\mathbb{Z}_2^{36}$	$\mathbb{Z}_2^{24}\times\mathbb{Z}_4^2$
$Z_6\times Z_6$	$\mathbb{Z}_2^2$	$\mathbb{Z}_2^{27}$	$\mathbb{Z}_2^{36}$	$\mathbb{Z}_2^{24}\times\mathbb{Z}_6^2$
$U(1)$		$\mathbb{Z}_2^7$	$\mathbb{Z}_2^3$	$\mathbb{Z}\times\mathbb{Z}_2^{19}$
$SU(2)$		$\mathbb{Z}_2^4$	$\mathbb{Z}_2^3$	$\mathbb{Z}_2^{14}$
$SO(3)$		$\mathbb{Z}_2^7$	$\mathbb{Z}_2^{12}$	$\mathbb{Z}_2^{14}$
Space group #70 $Fddd$				
$\varnothing$				$\mathbb{Z}_2^6$
Z_2^T	$\mathbb{Z}_2$		$\mathbb{Z}_2^8$	$\mathbb{Z}_2^6$
Z_2		$\mathbb{Z}_2^3$	$\mathbb{Z}_2^3$	$\mathbb{Z}_2^{10}$
$Z_2\times Z_2^T$	$\mathbb{Z}_2^3$	$\mathbb{Z}_2^6$	$\mathbb{Z}_2^{16}$	$\mathbb{Z}_2^{10}$
Z_4		$\mathbb{Z}_2^3$	$\mathbb{Z}_2^3$	$\mathbb{Z}_2^9\times\mathbb{Z}_4$
Z_6		$\mathbb{Z}_2^3$	$\mathbb{Z}_2^3$	$\mathbb{Z}_2^9\times\mathbb{Z}_6$
$Z_2\times Z_2$	$\mathbb{Z}_2^2$	$\mathbb{Z}_2^9$	$\mathbb{Z}_2^{11}$	$\mathbb{Z}_2^{14}$
$Z_4\times Z_4$	$\mathbb{Z}_2^2$	$\mathbb{Z}_2^9$	$\mathbb{Z}_2^{11}$	$\mathbb{Z}_2^{12}\times\mathbb{Z}_4^2$
$Z_6\times Z_6$	$\mathbb{Z}_2^2$	$\mathbb{Z}_2^9$	$\mathbb{Z}_2^{11}$	$\mathbb{Z}_2^{12}\times\mathbb{Z}_6^2$
$U(1)$		$\mathbb{Z}_2$		$\mathbb{Z}\times\mathbb{Z}_2^9$
$SU(2)$		$\mathbb{Z}_2$		$\mathbb{Z}_2^6$
$SO(3)$		$\mathbb{Z}_2$	$\mathbb{Z}_2^5$	$\mathbb{Z}_2^6$
Space group #71 $Immm$				
$\varnothing$		$\mathbb{Z}_2^3$	$\mathbb{Z}_2^6$	$\mathbb{Z}_2^{13}$
Z_2^T	$\mathbb{Z}_2$	$\mathbb{Z}_2^6$	$\mathbb{Z}_2^{25}$	$\mathbb{Z}_2^{13}$
Z_2		$\mathbb{Z}_2^{10}$	$\mathbb{Z}_2^{18}$	$\mathbb{Z}_2^{18}$
$Z_2\times Z_2^T$	$\mathbb{Z}_2^3$	$\mathbb{Z}_2^{20}$	$\mathbb{Z}_2^{44}$	$\mathbb{Z}_2^{18}$
Z_4		$\mathbb{Z}_2^9\times\mathbb{Z}_4$	$\mathbb{Z}_2^{18}$	$\mathbb{Z}_2^{17}\times\mathbb{Z}_4$
Z_6		$\mathbb{Z}_2^{10}$	$\mathbb{Z}_2^{18}$	$\mathbb{Z}_2^{17}\times\mathbb{Z}_6$
$Z_2\times Z_2$	$\mathbb{Z}_2^2$	$\mathbb{Z}_2^{24}$	$\mathbb{Z}_2^{37}$	$\mathbb{Z}_2^{23}$
$Z_4\times Z_4$	$\mathbb{Z}_2^2$	$\mathbb{Z}_2^{21}\times\mathbb{Z}_4^3$	$\mathbb{Z}_2^{36}\times\mathbb{Z}_4$	$\mathbb{Z}_2^{21}\times\mathbb{Z}_4^2$
$Z_6\times Z_6$	$\mathbb{Z}_2^2$	$\mathbb{Z}_2^{24}$	$\mathbb{Z}_2^{37}$	$\mathbb{Z}_2^{21}\times\mathbb{Z}_6^2$
$U(1)$		$\mathbb{Z}_2^7$	$\mathbb{Z}_2^6$	$\mathbb{Z}\times\mathbb{Z}_2^{17}$
$SU(2)$		$\mathbb{Z}_2^4$	$\mathbb{Z}_2^6$	$\mathbb{Z}_2^{13}$
$SO(3)$		$\mathbb{Z}_2^7$	$\mathbb{Z}_2^{13}$	$\mathbb{Z}_2^{13}$
Space group #72 $Ibam$				
$\varnothing$		$\mathbb{Z}_2$		$\mathbb{Z}_2^9$
Z_2^T	$\mathbb{Z}_2$	$\mathbb{Z}_2^2$	$\mathbb{Z}_2^{13}$	$\mathbb{Z}_2^9$
Z_2		$\mathbb{Z}_2^6$	$\mathbb{Z}_2^6$	$\mathbb{Z}_2^{14}$
$Z_2\times Z_2^T$	$\mathbb{Z}_2^3$	$\mathbb{Z}_2^{12}$	$\mathbb{Z}_2^{26}$	$\mathbb{Z}_2^{14}$
Z_4		$\mathbb{Z}_2^6$	$\mathbb{Z}_2^6$	$\mathbb{Z}_2^{13}\times\mathbb{Z}_4$
Z_6		$\mathbb{Z}_2^6$	$\mathbb{Z}_2^6$	$\mathbb{Z}_2^{13}\times\mathbb{Z}_6$
$Z_2\times Z_2$	$\mathbb{Z}_2^2$	$\mathbb{Z}_2^{16}$	$\mathbb{Z}_2^{19}$	$\mathbb{Z}_2^{19}$

TABLE II (Continued.)

G_0	$E^3_{3,\infty}$	$E^2_{2,\infty}$	$E^1_{1,\infty}$	$E^0_{0,\infty}$
$Z_4\times Z_4$	$\mathbb{Z}_2^2$	$\mathbb{Z}_2^{16}$	$\mathbb{Z}_2^{19}$	$\mathbb{Z}_2^{17}\times\mathbb{Z}_4^2$
$Z_6\times Z_6$	$\mathbb{Z}_2^2$	$\mathbb{Z}_2^{16}$	$\mathbb{Z}_2^{19}$	$\mathbb{Z}_2^{17}\times\mathbb{Z}_6^2$
$U(1)$		$\mathbb{Z}_2^3$		$\mathbb{Z}\times\mathbb{Z}_2^{13}$
$SU(2)$		$\mathbb{Z}_2^2$		$\mathbb{Z}_2^9$
$SO(3)$		$\mathbb{Z}_2^3$	$\mathbb{Z}_2^7$	$\mathbb{Z}_2^9$
Space group #73 $Ibca$				
$\varnothing$				$\mathbb{Z}_2^5$
Z_2^T	$\mathbb{Z}_2$		$\mathbb{Z}_2^{10}$	$\mathbb{Z}_2^5$
Z_2		$\mathbb{Z}_2^4$	$\mathbb{Z}_2^3$	$\mathbb{Z}_2^{10}$
$Z_2\times Z_2^T$	$\mathbb{Z}_2^3$	$\mathbb{Z}_2^8$	$\mathbb{Z}_2^{20}$	$\mathbb{Z}_2^{10}$
Z_4		$\mathbb{Z}_2^4$	$\mathbb{Z}_2^3$	$\mathbb{Z}_2^9\times\mathbb{Z}_4$
Z_6		$\mathbb{Z}_2^4$	$\mathbb{Z}_2^3$	$\mathbb{Z}_2^9\times\mathbb{Z}_6$
$Z_2\times Z_2$	$\mathbb{Z}_2^2$	$\mathbb{Z}_2^{12}$	$\mathbb{Z}_2^{13}$	$\mathbb{Z}_2^{15}$
$Z_4\times Z_4$	$\mathbb{Z}_2^2$	$\mathbb{Z}_2^{12}$	$\mathbb{Z}_2^{13}$	$\mathbb{Z}_2^{13}\times\mathbb{Z}_4^2$
$Z_6\times Z_6$	$\mathbb{Z}_2^2$	$\mathbb{Z}_2^{12}$	$\mathbb{Z}_2^{13}$	$\mathbb{Z}_2^{13}\times\mathbb{Z}_6^2$
$U(1)$		$\mathbb{Z}_2$		$\mathbb{Z}\times\mathbb{Z}_2^9$
$SU(2)$		$\mathbb{Z}_2$		$\mathbb{Z}_2^5$
$SO(3)$		$\mathbb{Z}_2$	$\mathbb{Z}_2^7$	$\mathbb{Z}_2^5$
Space group #74 $Imma$				
$\varnothing$		$\mathbb{Z}_2^2$	$\mathbb{Z}_2$	$\mathbb{Z}_2^{10}$
Z_2^T	$\mathbb{Z}_2$	$\mathbb{Z}_2^4$	$\mathbb{Z}_2^{16}$	$\mathbb{Z}_2^{10}$
Z_2		$\mathbb{Z}_2^8$	$\mathbb{Z}_2^9$	$\mathbb{Z}_2^{15}$
$Z_2\times Z_2^T$	$\mathbb{Z}_2^3$	$\mathbb{Z}_2^{16}$	$\mathbb{Z}_2^{31}$	$\mathbb{Z}_2^{15}$
Z_4		$\mathbb{Z}_2^8$	$\mathbb{Z}_2^9$	$\mathbb{Z}_2^{14}\times\mathbb{Z}_4$
Z_6		$\mathbb{Z}_2^8$	$\mathbb{Z}_2^9$	$\mathbb{Z}_2^{14}\times\mathbb{Z}_6$
$Z_2\times Z_2$	$\mathbb{Z}_2^2$	$\mathbb{Z}_2^{20}$	$\mathbb{Z}_2^{24}$	$\mathbb{Z}_2^{20}$
$Z_4\times Z_4$	$\mathbb{Z}_2^2$	$\mathbb{Z}_2^{20}$	$\mathbb{Z}_2^{24}$	$\mathbb{Z}_2^{18}\times\mathbb{Z}_4^2$
$Z_6\times Z_6$	$\mathbb{Z}_2^2$	$\mathbb{Z}_2^{20}$	$\mathbb{Z}_2^{24}$	$\mathbb{Z}_2^{18}\times\mathbb{Z}_6^2$
$U(1)$		$\mathbb{Z}_2^5$	$\mathbb{Z}_2$	$\mathbb{Z}\times\mathbb{Z}_2^{14}$
$SU(2)$		$\mathbb{Z}_2^3$	$\mathbb{Z}_2$	$\mathbb{Z}_2^{10}$
$SO(3)$		$\mathbb{Z}_2^5$	$\mathbb{Z}_2^8$	$\mathbb{Z}_2^{10}$
Space group #75 $P4$				
$\varnothing$				$\mathbb{Z}_2\times\mathbb{Z}_4^2$
Z_2^T	$\mathbb{Z}_2$		$\mathbb{Z}_2^8$	$\mathbb{Z}_2^3$
Z_2		$\mathbb{Z}_2^3$	$\mathbb{Z}_2^3$	$\mathbb{Z}_2^4\times\mathbb{Z}_4^2$
$Z_2\times Z_2^T$	$\mathbb{Z}_2^3$	$\mathbb{Z}_2^6$	$\mathbb{Z}_2^{16}$	$\mathbb{Z}_2^6$
Z_4		$\mathbb{Z}_2\times\mathbb{Z}_4^2$	$\mathbb{Z}_2\times\mathbb{Z}_4^2$	$\mathbb{Z}_2^2\times\mathbb{Z}_4^4$
Z_6		$\mathbb{Z}_2^2\times\mathbb{Z}_6$	$\mathbb{Z}_2^3$	$\mathbb{Z}_2^3\times\mathbb{Z}_4^2\times\mathbb{Z}_6$
$Z_2\times Z_2$	$\mathbb{Z}_2^2$	$\mathbb{Z}_2^9$	$\mathbb{Z}_2^{11}$	$\mathbb{Z}_2^7\times\mathbb{Z}_4^2$
$Z_4\times Z_4$	$\mathbb{Z}_4^2$	$\mathbb{Z}_2^3\times\mathbb{Z}_4^6$	$\mathbb{Z}_2^4\times\mathbb{Z}_4^7$	$\mathbb{Z}_2^3\times\mathbb{Z}_4^6$
$Z_6\times Z_6$	$\mathbb{Z}_6^2$	$\mathbb{Z}_2^6\times\mathbb{Z}_6^3$	$\mathbb{Z}_2^{10}\times\mathbb{Z}_6$	$\mathbb{Z}_2^5\times\mathbb{Z}_4^2\times\mathbb{Z}_6^2$
$U(1)$		$\mathbb{Z}$		$\mathbb{Z}\times\mathbb{Z}_2^2\times\mathbb{Z}_4^3$
$SU(2)$		$\mathbb{Z}$		$\mathbb{Z}_2\times\mathbb{Z}_4^2$
$SO(3)$		$\mathbb{Z}$	$\mathbb{Z}_2^5$	$\mathbb{Z}_2\times\mathbb{Z}_4^2$
Space group #76 $P4_1$				
$\varnothing$				
Z_2^T	$\mathbb{Z}_2$		$\mathbb{Z}_2^2$	
Z_2		$\mathbb{Z}_2^2$		$\mathbb{Z}_2$
$Z_2\times Z_2^T$	$\mathbb{Z}_2^3$	$\mathbb{Z}_2^4$	$\mathbb{Z}_2^4$	$\mathbb{Z}_2$

TABLE II (Continued.)

G_0	$E_{3,\infty}^3$	$E_{2,\infty}^2$	$E_{1,\infty}^1$	$E_{0,\infty}^0$
Z_4		$\mathbb{Z}_2\times\mathbb{Z}_4$		$\mathbb{Z}_4$
Z_6		$\mathbb{Z}_2\times\mathbb{Z}_6$		$\mathbb{Z}_6$
$Z_2\times Z_2$	$\mathbb{Z}_2^2$	$\mathbb{Z}_2^6$	$\mathbb{Z}_2^2$	$\mathbb{Z}_2^2$
$Z_4\times Z_4$	$\mathbb{Z}_4^2$	$\mathbb{Z}_2^3\times\mathbb{Z}_4^3$	$\mathbb{Z}_2\times\mathbb{Z}_4$	$\mathbb{Z}_4^2$
$Z_6\times Z_6$	$\mathbb{Z}_6^2$	$\mathbb{Z}_2^3\times\mathbb{Z}_6^3$	$\mathbb{Z}_2\times\mathbb{Z}_6$	$\mathbb{Z}_6^2$
$U(1)$		$\mathbb{Z}$		$\mathbb{Z}$
$SU(2)$		$\mathbb{Z}$		
$SO(3)$		$\mathbb{Z}$	$\mathbb{Z}_2^2$	
Space group #77 $P4_2$				
$\varnothing$				$\mathbb{Z}_2^3$
Z_2^T	$\mathbb{Z}_2$		$\mathbb{Z}_2^8$	$\mathbb{Z}_2^3$
Z_2		$\mathbb{Z}_2^3$	$\mathbb{Z}_2^3$	$\mathbb{Z}_2^6$
$Z_2\times Z_2^T$	$\mathbb{Z}_2^3$	$\mathbb{Z}_2^6$	$\mathbb{Z}_2^{16}$	$\mathbb{Z}_2^6$
Z_4		$\mathbb{Z}_2^2\times\mathbb{Z}_4$	$\mathbb{Z}_2^3$	$\mathbb{Z}_2^5\times\mathbb{Z}_4$
Z_6		$\mathbb{Z}_2^2\times\mathbb{Z}_6$	$\mathbb{Z}_2^3$	$\mathbb{Z}_2^5\times\mathbb{Z}_6$
$Z_2\times Z_2$	$\mathbb{Z}_2^2$	$\mathbb{Z}_2^9$	$\mathbb{Z}_2^{11}$	$\mathbb{Z}_2^9$
$Z_4\times Z_4$	$\mathbb{Z}_4^2$	$\mathbb{Z}_2^6\times\mathbb{Z}_4^3$	$\mathbb{Z}_2^{10}\times\mathbb{Z}_4$	$\mathbb{Z}_2^7\times\mathbb{Z}_4^2$
$Z_6\times Z_6$	$\mathbb{Z}_6^2$	$\mathbb{Z}_2^6\times\mathbb{Z}_6^3$	$\mathbb{Z}_2^{10}\times\mathbb{Z}_6$	$\mathbb{Z}_2^7\times\mathbb{Z}_6^2$
$U(1)$		$\mathbb{Z}$		$\mathbb{Z}\times\mathbb{Z}_2^5$
$SU(2)$		$\mathbb{Z}$		$\mathbb{Z}_2^3$
$SO(3)$		$\mathbb{Z}$	$\mathbb{Z}_2^5$	$\mathbb{Z}_2^3$
Space group #78 $P4_3$				
$\varnothing$				
Z_2^T	$\mathbb{Z}_2$		$\mathbb{Z}_2^2$	
Z_2		$\mathbb{Z}_2^2$		$\mathbb{Z}_2$
$Z_2\times Z_2^T$	$\mathbb{Z}_2^3$	$\mathbb{Z}_2^4$	$\mathbb{Z}_2^4$	$\mathbb{Z}_2$
Z_4		$\mathbb{Z}_2\times\mathbb{Z}_4$		$\mathbb{Z}_4$
Z_6		$\mathbb{Z}_2\times\mathbb{Z}_6$		$\mathbb{Z}_6$
$Z_2\times Z_2$	$\mathbb{Z}_2^2$	$\mathbb{Z}_2^6$	$\mathbb{Z}_2^2$	$\mathbb{Z}_2^2$
$Z_4\times Z_4$	$\mathbb{Z}_4^2$	$\mathbb{Z}_2^3\times\mathbb{Z}_4^3$	$\mathbb{Z}_2\times\mathbb{Z}_4$	$\mathbb{Z}_4^2$
$Z_6\times Z_6$	$\mathbb{Z}_6^2$	$\mathbb{Z}_2^3\times\mathbb{Z}_6^3$	$\mathbb{Z}_2\times\mathbb{Z}_6$	$\mathbb{Z}_6^2$
$U(1)$		$\mathbb{Z}$		$\mathbb{Z}$
$SU(2)$		$\mathbb{Z}$		
$SO(3)$		$\mathbb{Z}$	$\mathbb{Z}_2^2$	
Space group #79 $I4$				
$\varnothing$				$\mathbb{Z}_2\times\mathbb{Z}_4$
Z_2^T	$\mathbb{Z}_2$		$\mathbb{Z}_2^5$	$\mathbb{Z}_2^2$
Z_2		$\mathbb{Z}_2^2$	$\mathbb{Z}_2^2$	$\mathbb{Z}_2^3\times\mathbb{Z}_4$
$Z_2\times Z_2^T$	$\mathbb{Z}_2^3$	$\mathbb{Z}_2^4$	$\mathbb{Z}_2^{10}$	$\mathbb{Z}_2^4$
Z_4		$\mathbb{Z}_4^2$	$\mathbb{Z}_2\times\mathbb{Z}_4$	$\mathbb{Z}_2^2\times\mathbb{Z}_4^2$
Z_6		$\mathbb{Z}_2\times\mathbb{Z}_6$	$\mathbb{Z}_2^2$	$\mathbb{Z}_2^2\times\mathbb{Z}_4\times\mathbb{Z}_6$
$Z_2\times Z_2$	$\mathbb{Z}_2^2$	$\mathbb{Z}_2^6$	$\mathbb{Z}_2^7$	$\mathbb{Z}_2^5\times\mathbb{Z}_4$
$Z_4\times Z_4$	$\mathbb{Z}_4^2$	$\mathbb{Z}_4^6$	$\mathbb{Z}_2^3\times\mathbb{Z}_4^4$	$\mathbb{Z}_2^3\times\mathbb{Z}_4^3$
$Z_6\times Z_6$	$\mathbb{Z}_6^2$	$\mathbb{Z}_2^3\times\mathbb{Z}_6^3$	$\mathbb{Z}_2^6\times\mathbb{Z}_6$	$\mathbb{Z}_2^3\times\mathbb{Z}_4\times\mathbb{Z}_6^2$
$U(1)$		$\mathbb{Z}$		$\mathbb{Z}\times\mathbb{Z}_2^2\times\mathbb{Z}_4$
$SU(2)$		$\mathbb{Z}$		$\mathbb{Z}_2\times\mathbb{Z}_4$
$SO(3)$		$\mathbb{Z}$	$\mathbb{Z}_2^3$	$\mathbb{Z}_2\times\mathbb{Z}_4$
Space group #80 $I4_1$				
$\varnothing$				$\mathbb{Z}_2$

TABLE II (Continued.)

G_0	$E_{3,\infty}^3$	$E_{2,\infty}^2$	$E_{1,\infty}^1$	$E_{0,\infty}^0$
Z_2^T	$\mathbb{Z}_2$		$\mathbb{Z}_2^3$	$\mathbb{Z}_2$
Z_2		$\mathbb{Z}_2^2$	$\mathbb{Z}_2$	$\mathbb{Z}_2^2$
$Z_2\times Z_2^T$	$\mathbb{Z}_2^3$	$\mathbb{Z}_2^4$	$\mathbb{Z}_2^6$	$\mathbb{Z}_2^2$
Z_4		$\mathbb{Z}_2\times\mathbb{Z}_4$	$\mathbb{Z}_2$	$\mathbb{Z}_2\times\mathbb{Z}_4$
Z_6		$\mathbb{Z}_2\times\mathbb{Z}_6$	$\mathbb{Z}_2$	$\mathbb{Z}_2\times\mathbb{Z}_6$
$Z_2\times Z_2$	$\mathbb{Z}_2^2$	$\mathbb{Z}_2^6$	$\mathbb{Z}_2^4$	$\mathbb{Z}_2^3$
$Z_4\times Z_4$	$\mathbb{Z}_4^2$	$\mathbb{Z}_2^3\times\mathbb{Z}_4^3$	$\mathbb{Z}_2^3\times\mathbb{Z}_4$	$\mathbb{Z}_2\times\mathbb{Z}_4^2$
$Z_6\times Z_6$	$\mathbb{Z}_6^2$	$\mathbb{Z}_2^3\times\mathbb{Z}_6^3$	$\mathbb{Z}_2^3\times\mathbb{Z}_6$	$\mathbb{Z}_2\times\mathbb{Z}_6^2$
$U(1)$		$\mathbb{Z}$		$\mathbb{Z}\times\mathbb{Z}_2$
$SU(2)$		$\mathbb{Z}$		$\mathbb{Z}_2$
$SO(3)$		$\mathbb{Z}$	$\mathbb{Z}_2^2$	$\mathbb{Z}_2$
Space group #81 $P\bar{4}$				
$\varnothing$				$\mathbb{Z}_2^3\times\mathbb{Z}_4^2$
Z_2^T	$\mathbb{Z}_2$		$\mathbb{Z}_2^8$	$\mathbb{Z}_2^3$
Z_2		$\mathbb{Z}_2^3$	$\mathbb{Z}_2$	$\mathbb{Z}_2^8\times\mathbb{Z}_4^2$
$Z_2\times Z_2^T$	$\mathbb{Z}_2^3$	$\mathbb{Z}_2^6$	$\mathbb{Z}_2^{14}$	$\mathbb{Z}_2^8$
Z_4		$\mathbb{Z}_2^2\times\mathbb{Z}_4$	$\mathbb{Z}_2$	$\mathbb{Z}_2^6\times\mathbb{Z}_4^4$
Z_6		$\mathbb{Z}_2^2\times\mathbb{Z}_6$	$\mathbb{Z}_2$	$\mathbb{Z}_2^7\times\mathbb{Z}_4^2\times\mathbb{Z}_6$
$Z_2\times Z_2$	$\mathbb{Z}_2^2$	$\mathbb{Z}_2^9$	$\mathbb{Z}_2^7$	$\mathbb{Z}_2^{13}\times\mathbb{Z}_4^2$
$Z_4\times Z_4$	$\mathbb{Z}_2^2$	$\mathbb{Z}_2^6\times\mathbb{Z}_4^3$	$\mathbb{Z}_2^6\times\mathbb{Z}_4$	$\mathbb{Z}_2^9\times\mathbb{Z}_4^6$
$Z_6\times Z_6$	$\mathbb{Z}_2^2$	$\mathbb{Z}_2^6\times\mathbb{Z}_6^3$	$\mathbb{Z}_2^7$	$\mathbb{Z}_2^{11}\times\mathbb{Z}_4^2\times\mathbb{Z}_6^2$
$U(1)$		$\mathbb{Z}\times\mathbb{Z}_2$		$\mathbb{Z}\times\mathbb{Z}_2^6\times\mathbb{Z}_4^3$
$SU(2)$		$\mathbb{Z}\times\mathbb{Z}_2$		$\mathbb{Z}_2^3\times\mathbb{Z}_4^2$
$SO(3)$		$\mathbb{Z}\times\mathbb{Z}_2$	$\mathbb{Z}_2^5$	$\mathbb{Z}_2^3\times\mathbb{Z}_4^2$
Space group #82 $I\bar{4}$				
$\varnothing$				$\mathbb{Z}_2^2\times\mathbb{Z}_4^2$
Z_2^T	$\mathbb{Z}_2$		$\mathbb{Z}_2^5$	$\mathbb{Z}_2^2$
Z_2		$\mathbb{Z}_2^2$		$\mathbb{Z}_2^6\times\mathbb{Z}_4^2$
$Z_2\times Z_2^T$	$\mathbb{Z}_2^3$	$\mathbb{Z}_2^4$	$\mathbb{Z}_2^8$	$\mathbb{Z}_2^6$
Z_4		$\mathbb{Z}_2\times\mathbb{Z}_4$		$\mathbb{Z}_2^4\times\mathbb{Z}_4^4$
Z_6		$\mathbb{Z}_2\times\mathbb{Z}_6$		$\mathbb{Z}_2^5\times\mathbb{Z}_4^2\times\mathbb{Z}_6$
$Z_2\times Z_2$	$\mathbb{Z}_2^2$	$\mathbb{Z}_2^6$	$\mathbb{Z}_2^3$	$\mathbb{Z}_2^{10}\times\mathbb{Z}_4^2$
$Z_4\times Z_4$	$\mathbb{Z}_2^2$	$\mathbb{Z}_2^3\times\mathbb{Z}_4^3$	$\mathbb{Z}_2^2\times\mathbb{Z}_4$	$\mathbb{Z}_2^6\times\mathbb{Z}_4^6$
$Z_6\times Z_6$	$\mathbb{Z}_2^2$	$\mathbb{Z}_2^3\times\mathbb{Z}_6^3$	$\mathbb{Z}_2^3$	$\mathbb{Z}_2^8\times\mathbb{Z}_4^2\times\mathbb{Z}_6^2$
$U(1)$		$\mathbb{Z}\times\mathbb{Z}_2$		$\mathbb{Z}\times\mathbb{Z}_2^4\times\mathbb{Z}_4^3$
$SU(2)$		$\mathbb{Z}\times\mathbb{Z}_2$		$\mathbb{Z}_2^2\times\mathbb{Z}_4^2$
$SO(3)$		$\mathbb{Z}\times\mathbb{Z}_2$	$\mathbb{Z}_2^3$	$\mathbb{Z}_2^2\times\mathbb{Z}_4^2$
Space group #83 $P4/m$				
$\varnothing$		$\mathbb{Z}_2^2$		$\mathbb{Z}_2^{10}\times\mathbb{Z}_4^2$
Z_2^T	$\mathbb{Z}_2$	$\mathbb{Z}_2^4$	$\mathbb{Z}_2^{14}$	$\mathbb{Z}_2^{12}$
Z_2		$\mathbb{Z}_2^8$	$\mathbb{Z}_2^7$	$\mathbb{Z}_2^{16}\times\mathbb{Z}_4^2$
$Z_2\times Z_2^T$	$\mathbb{Z}_2^3$	$\mathbb{Z}_2^{16}$	$\mathbb{Z}_2^{28}$	$\mathbb{Z}_2^{18}$
Z_4		$\mathbb{Z}_2^7\times\mathbb{Z}_4$	$\mathbb{Z}_2^7$	$\mathbb{Z}_2^{14}\times\mathbb{Z}_4^4$
Z_6		$\mathbb{Z}_2^7\times\mathbb{Z}_6$	$\mathbb{Z}_2^7$	$\mathbb{Z}_2^{15}\times\mathbb{Z}_4^2\times\mathbb{Z}_6$
$Z_2\times Z_2$	$\mathbb{Z}_2^2$	$\mathbb{Z}_2^{20}$	$\mathbb{Z}_2^{21}$	$\mathbb{Z}_2^{22}\times\mathbb{Z}_4^2$
$Z_4\times Z_4$	$\mathbb{Z}_2^2$	$\mathbb{Z}_2^{17}\times\mathbb{Z}_4^3$	$\mathbb{Z}_2^{20}\times\mathbb{Z}_4$	$\mathbb{Z}_2^{18}\times\mathbb{Z}_4^6$
$Z_6\times Z_6$	$\mathbb{Z}_2^2$	$\mathbb{Z}_2^{17}\times\mathbb{Z}_6^3$	$\mathbb{Z}_2^{21}$	$\mathbb{Z}_2^{20}\times\mathbb{Z}_4^2\times\mathbb{Z}_6^2$
$U(1)$		$\mathbb{Z}\times\mathbb{Z}_2^5$		$\mathbb{Z}\times\mathbb{Z}_2^{14}\times\mathbb{Z}_4^3$
$SU(2)$		$\mathbb{Z}\times\mathbb{Z}_2^3$		$\mathbb{Z}_2^{10}\times\mathbb{Z}_4^2$

TABLE II (Continued.)

G_0	$E^3_{3,\infty}$	$E^2_{2,\infty}$	$E^1_{1,\infty}$	$E^0_{0,\infty}$
$SO(3)$		$\mathbb{Z}\times\mathbb{Z}_2^5$	$\mathbb{Z}_2^7$	$\mathbb{Z}_2^{10}\times\mathbb{Z}_4^2$
Space group #84 $P4_2/m$				
$\varnothing$		$\mathbb{Z}_2$		$\mathbb{Z}_2^{10}$
Z_2^T	$\mathbb{Z}_2$	$\mathbb{Z}_2^2$	$\mathbb{Z}_2^{12}$	$\mathbb{Z}_2^8$
Z_2		$\mathbb{Z}_2^5$	$\mathbb{Z}_2^4$	$\mathbb{Z}_2^{16}$
$Z_2\times Z_2^T$	$\mathbb{Z}_2^3$	$\mathbb{Z}_2^{10}$	$\mathbb{Z}_2^{22}$	$\mathbb{Z}_2^{14}$
Z_4		$\mathbb{Z}_2^4\times\mathbb{Z}_4$	$\mathbb{Z}_2^4$	$\mathbb{Z}_2^{15}\times\mathbb{Z}_4$
Z_6		$\mathbb{Z}_2^4\times\mathbb{Z}_6$	$\mathbb{Z}_2^4$	$\mathbb{Z}_2^{15}\times\mathbb{Z}_6$
$Z_2\times Z_2$	$\mathbb{Z}_2^2$	$\mathbb{Z}_2^{13}$	$\mathbb{Z}_2^{14}$	$\mathbb{Z}_2^{22}$
$Z_4\times Z_4$	$\mathbb{Z}_2^2$	$\mathbb{Z}_2^{10}\times\mathbb{Z}_4^3$	$\mathbb{Z}_2^{14}$	$\mathbb{Z}_2^{20}\times\mathbb{Z}_4^2$
$Z_6\times Z_6$	$\mathbb{Z}_2^2$	$\mathbb{Z}_2^{10}\times\mathbb{Z}_6^3$	$\mathbb{Z}_2^{14}$	$\mathbb{Z}_2^{20}\times\mathbb{Z}_6^2$
$U(1)$		$\mathbb{Z}\times\mathbb{Z}_2^3$		$\mathbb{Z}\times\mathbb{Z}_2^{15}$
$SU(2)$		$\mathbb{Z}\times\mathbb{Z}_2^2$		$\mathbb{Z}_2^{10}$
$SO(3)$		$\mathbb{Z}\times\mathbb{Z}_2^3$	$\mathbb{Z}_2^6$	$\mathbb{Z}_2^{10}$
Space group #85 $P4/n$				
$\varnothing$				$\mathbb{Z}_2^3\times\mathbb{Z}_4^2$
Z_2^T	$\mathbb{Z}_2$		$\mathbb{Z}_2^7$	$\mathbb{Z}_2^4$
Z_2		$\mathbb{Z}_2^3$	$\mathbb{Z}_2$	$\mathbb{Z}_2^8\times\mathbb{Z}_4^2$
$Z_2\times Z_2^T$	$\mathbb{Z}_2^3$	$\mathbb{Z}_2^6$	$\mathbb{Z}_2^{13}$	$\mathbb{Z}_2^9$
Z_4		$\mathbb{Z}_2\times\mathbb{Z}_4^2$	$\mathbb{Z}_4$	$\mathbb{Z}_2^6\times\mathbb{Z}_4^4$
Z_6		$\mathbb{Z}_2^2\times\mathbb{Z}_6$	$\mathbb{Z}_2$	$\mathbb{Z}_2^7\times\mathbb{Z}_4^2\times\mathbb{Z}_6$
$Z_2\times Z_2$	$\mathbb{Z}_2^2$	$\mathbb{Z}_2^9$	$\mathbb{Z}_2^7$	$\mathbb{Z}_2^{13}\times\mathbb{Z}_4^2$
$Z_4\times Z_4$	$\mathbb{Z}_2^2$	$\mathbb{Z}_2^3\times\mathbb{Z}_4^6$	$\mathbb{Z}_2^3\times\mathbb{Z}_4^4$	$\mathbb{Z}_2^9\times\mathbb{Z}_4^6$
$Z_6\times Z_6$	$\mathbb{Z}_2^2$	$\mathbb{Z}_2^6\times\mathbb{Z}_6^3$	$\mathbb{Z}_2^7$	$\mathbb{Z}_2^{11}\times\mathbb{Z}_4^2\times\mathbb{Z}_6^2$
$U(1)$		$\mathbb{Z}\times\mathbb{Z}_2$		$\mathbb{Z}\times\mathbb{Z}_2^6\times\mathbb{Z}_4^3$
$SU(2)$		$\mathbb{Z}\times\mathbb{Z}_2$		$\mathbb{Z}_2^3\times\mathbb{Z}_4^2$
$SO(3)$		$\mathbb{Z}\times\mathbb{Z}_2$	$\mathbb{Z}_2^5$	$\mathbb{Z}_2^3\times\mathbb{Z}_4^2$
Space group #86 $P4_2/n$				
$\varnothing$				$\mathbb{Z}_2^4\times\mathbb{Z}_4$
Z_2^T	$\mathbb{Z}_2$		$\mathbb{Z}_2^7$	$\mathbb{Z}_2^4$
Z_2		$\mathbb{Z}_2^3$	$\mathbb{Z}_2$	$\mathbb{Z}_2^9\times\mathbb{Z}_4$
$Z_2\times Z_2^T$	$\mathbb{Z}_2^3$	$\mathbb{Z}_2^6$	$\mathbb{Z}_2^{13}$	$\mathbb{Z}_2^9$
Z_4		$\mathbb{Z}_2^2\times\mathbb{Z}_4$	$\mathbb{Z}_2$	$\mathbb{Z}_2^8\times\mathbb{Z}_4^2$
Z_6		$\mathbb{Z}_2^2\times\mathbb{Z}_6$	$\mathbb{Z}_2$	$\mathbb{Z}_2^8\times\mathbb{Z}_4\times\mathbb{Z}_6$
$Z_2\times Z_2$	$\mathbb{Z}_2^2$	$\mathbb{Z}_2^9$	$\mathbb{Z}_2^7$	$\mathbb{Z}_2^{14}\times\mathbb{Z}_4$
$Z_4\times Z_4$	$\mathbb{Z}_2^2$	$\mathbb{Z}_2^6\times\mathbb{Z}_4^3$	$\mathbb{Z}_2^7$	$\mathbb{Z}_2^{12}\times\mathbb{Z}_4^3$
$Z_6\times Z_6$	$\mathbb{Z}_2^2$	$\mathbb{Z}_2^6\times\mathbb{Z}_6^3$	$\mathbb{Z}_2^7$	$\mathbb{Z}_2^{12}\times\mathbb{Z}_4\times\mathbb{Z}_6^2$
$U(1)$		$\mathbb{Z}\times\mathbb{Z}_2$		$\mathbb{Z}\times\mathbb{Z}_2^8\times\mathbb{Z}_4$
$SU(2)$		$\mathbb{Z}\times\mathbb{Z}_2$		$\mathbb{Z}_2^4\times\mathbb{Z}_4$
$SO(3)$		$\mathbb{Z}\times\mathbb{Z}_2$	$\mathbb{Z}_2^5$	$\mathbb{Z}_2^4\times\mathbb{Z}_4$
Space group #87 $I4/m$				
$\varnothing$		$\mathbb{Z}_2$		$\mathbb{Z}_2^7\times\mathbb{Z}_4$
Z_2^T	$\mathbb{Z}_2$	$\mathbb{Z}_2^2$	$\mathbb{Z}_2^9$	$\mathbb{Z}_2^7$
Z_2		$\mathbb{Z}_2^5$	$\mathbb{Z}_2^3$	$\mathbb{Z}_2^{12}\times\mathbb{Z}_4$
$Z_2\times Z_2^T$	$\mathbb{Z}_2^3$	$\mathbb{Z}_2^{10}$	$\mathbb{Z}_2^{17}$	$\mathbb{Z}_2^{12}$
Z_4		$\mathbb{Z}_2^4\times\mathbb{Z}_4$	$\mathbb{Z}_2^3$	$\mathbb{Z}_2^{11}\times\mathbb{Z}_4^2$
Z_6		$\mathbb{Z}_2^4\times\mathbb{Z}_6$	$\mathbb{Z}_2^3$	$\mathbb{Z}_2^{11}\times\mathbb{Z}_4\times\mathbb{Z}_6$
$Z_2\times Z_2$	$\mathbb{Z}_2^2$	$\mathbb{Z}_2^{13}$	$\mathbb{Z}_2^{11}$	$\mathbb{Z}_2^{17}\times\mathbb{Z}_4$
$Z_4\times Z_4$	$\mathbb{Z}_2^2$	$\mathbb{Z}_2^{10}\times\mathbb{Z}_4^3$	$\mathbb{Z}_2^{11}$	$\mathbb{Z}_2^{15}\times\mathbb{Z}_4^3$

TABLE II (Continued.)

G_0	$E^3_{3,\infty}$	$E^2_{2,\infty}$	$E^1_{1,\infty}$	$E^0_{0,\infty}$
$Z_6\times Z_6$	$\mathbb{Z}_2^2$	$\mathbb{Z}_2^{10}\times\mathbb{Z}_6^3$	$\mathbb{Z}_2^{11}$	$\mathbb{Z}_2^{15}\times\mathbb{Z}_4\times\mathbb{Z}_6^2$
$U(1)$		$\mathbb{Z}\times\mathbb{Z}_2^3$		$\mathbb{Z}\times\mathbb{Z}_2^{11}\times\mathbb{Z}_4$
$SU(2)$		$\mathbb{Z}\times\mathbb{Z}_2^2$		$\mathbb{Z}_2^7\times\mathbb{Z}_4$
$SO(3)$		$\mathbb{Z}\times\mathbb{Z}_2^3$	$\mathbb{Z}_2^5$	$\mathbb{Z}_2^7\times\mathbb{Z}_4$
Space group #88 $I4_1/a$				
$\varnothing$				$\mathbb{Z}_2^3\times\mathbb{Z}_4$
Z_2^T	$\mathbb{Z}_2$		$\mathbb{Z}_2^4$	$\mathbb{Z}_2^3$
Z_2		$\mathbb{Z}_2^2$		$\mathbb{Z}_2^7\times\mathbb{Z}_4$
$Z_2\times Z_2^T$	$\mathbb{Z}_2^3$	$\mathbb{Z}_2^4$	$\mathbb{Z}_2^7$	$\mathbb{Z}_2^7$
Z_4		$\mathbb{Z}_2\times\mathbb{Z}_4$		$\mathbb{Z}_2^6\times\mathbb{Z}_4^2$
Z_6		$\mathbb{Z}_2\times\mathbb{Z}_6$		$\mathbb{Z}_2^6\times\mathbb{Z}_4\times\mathbb{Z}_6$
$Z_2\times Z_2$	$\mathbb{Z}_2^2$	$\mathbb{Z}_2^6$	$\mathbb{Z}_2^3$	$\mathbb{Z}_2^{11}\times\mathbb{Z}_4$
$Z_4\times Z_4$	$\mathbb{Z}_2^2$	$\mathbb{Z}_2^3\times\mathbb{Z}_4^3$	$\mathbb{Z}_2^3$	$\mathbb{Z}_2^9\times\mathbb{Z}_4^3$
$Z_6\times Z_6$	$\mathbb{Z}_2^2$	$\mathbb{Z}_2^3\times\mathbb{Z}_6^3$	$\mathbb{Z}_2^3$	$\mathbb{Z}_2^9\times\mathbb{Z}_4\times\mathbb{Z}_6^2$
$U(1)$		$\mathbb{Z}\times\mathbb{Z}_2$		$\mathbb{Z}\times\mathbb{Z}_2^6\times\mathbb{Z}_4$
$SU(2)$		$\mathbb{Z}\times\mathbb{Z}_2$		$\mathbb{Z}_2^3\times\mathbb{Z}_4$
$SO(3)$		$\mathbb{Z}\times\mathbb{Z}_2$	$\mathbb{Z}_2^3$	$\mathbb{Z}_2^3\times\mathbb{Z}_4$
Space group #89 $P422$				
$\varnothing$				$\mathbb{Z}_2^{12}$
Z_2^T	$\mathbb{Z}_2$		$\mathbb{Z}_2^{18}$	$\mathbb{Z}_2^{12}$
Z_2		$\mathbb{Z}_2^4$	$\mathbb{Z}_2^9$	$\mathbb{Z}_2^{18}$
$Z_2\times Z_2^T$	$\mathbb{Z}_2^3$	$\mathbb{Z}_2^8$	$\mathbb{Z}_2^{36}$	$\mathbb{Z}_2^{18}$
Z_4		$\mathbb{Z}_2^4$	$\mathbb{Z}_2^7\times\mathbb{Z}_4^2$	$\mathbb{Z}_2^{16}\times\mathbb{Z}_4^2$
Z_6		$\mathbb{Z}_2^4$	$\mathbb{Z}_2^9$	$\mathbb{Z}_2^{17}\times\mathbb{Z}_6$
$Z_2\times Z_2$	$\mathbb{Z}_2^2$	$\mathbb{Z}_2^{12}$	$\mathbb{Z}_2^{27}$	$\mathbb{Z}_2^{24}$
$Z_4\times Z_4$	$\mathbb{Z}_4^2$	$\mathbb{Z}_2^{12}$	$\mathbb{Z}_2^{22}\times\mathbb{Z}_4^5$	$\mathbb{Z}_2^{20}\times\mathbb{Z}_4^4$
$Z_6\times Z_6$	$\mathbb{Z}_6^2$	$\mathbb{Z}_2^{12}$	$\mathbb{Z}_2^{27}$	$\mathbb{Z}_2^{22}\times\mathbb{Z}_6^2$
$U(1)$				$\mathbb{Z}\times\mathbb{Z}_2^{16}\times\mathbb{Z}_4$
$SU(2)$				$\mathbb{Z}_2^{12}$
$SO(3)$			$\mathbb{Z}_2^9$	$\mathbb{Z}_2^{12}$
Space group #90 $P42_12$				
$\varnothing$				$\mathbb{Z}_2^4\times\mathbb{Z}_4$
Z_2^T	$\mathbb{Z}_2$		$\mathbb{Z}_2^9$	$\mathbb{Z}_2^5$
Z_2		$\mathbb{Z}_2^3$	$\mathbb{Z}_2^4$	$\mathbb{Z}_2^7\times\mathbb{Z}_4$
$Z_2\times Z_2^T$	$\mathbb{Z}_2^3$	$\mathbb{Z}_2^6$	$\mathbb{Z}_2^{18}$	$\mathbb{Z}_2^8$
Z_4		$\mathbb{Z}_2^2\times\mathbb{Z}_4$	$\mathbb{Z}_2^3\times\mathbb{Z}_4$	$\mathbb{Z}_2^5\times\mathbb{Z}_4^3$
Z_6		$\mathbb{Z}_2^3$	$\mathbb{Z}_2^4$	$\mathbb{Z}_2^6\times\mathbb{Z}_4\times\mathbb{Z}_6$
$Z_2\times Z_2$	$\mathbb{Z}_2^2$	$\mathbb{Z}_2^9$	$\mathbb{Z}_2^{13}$	$\mathbb{Z}_2^{10}\times\mathbb{Z}_4$
$Z_4\times Z_4$	$\mathbb{Z}_4^2$	$\mathbb{Z}_2^6\times\mathbb{Z}_4^3$	$\mathbb{Z}_2^9\times\mathbb{Z}_4^4$	$\mathbb{Z}_2^6\times\mathbb{Z}_4^5$
$Z_6\times Z_6$	$\mathbb{Z}_6^2$	$\mathbb{Z}_2^9$	$\mathbb{Z}_2^{13}$	$\mathbb{Z}_2^8\times\mathbb{Z}_4\times\mathbb{Z}_6^2$
$U(1)$				$\mathbb{Z}\times\mathbb{Z}_2^5\times\mathbb{Z}_4^2$
$SU(2)$				$\mathbb{Z}_2^4\times\mathbb{Z}_4$
$SO(3)$			$\mathbb{Z}_2^5$	$\mathbb{Z}_2^4\times\mathbb{Z}_4$
Space group #91 $P4_122$				
$\varnothing$				$\mathbb{Z}_2^3$
Z_2^T	$\mathbb{Z}_2$		$\mathbb{Z}_2^8$	$\mathbb{Z}_2^3$
Z_2		$\mathbb{Z}_2^3$	$\mathbb{Z}_2^3$	$\mathbb{Z}_2^6$
$Z_2\times Z_2^T$	$\mathbb{Z}_2^3$	$\mathbb{Z}_2^6$	$\mathbb{Z}_2^{16}$	$\mathbb{Z}_2^6$
Z_4		$\mathbb{Z}_2^3$	$\mathbb{Z}_2^3$	$\mathbb{Z}_2^5\times\mathbb{Z}_4$

TABLE II (Continued.)

G_0	$E_{3,\infty}^3$	$E_{2,\infty}^2$	$E_{1,\infty}^1$	$E_{0,\infty}^0$
Z_6		$\mathbb{Z}_2^3$	$\mathbb{Z}_2^3$	$\mathbb{Z}_2^5 \times \mathbb{Z}_6$
$Z_2 \times Z_2$	$\mathbb{Z}_2^2$	$\mathbb{Z}_2^9$	$\mathbb{Z}_2^{11}$	$\mathbb{Z}_2^9$
$Z_4 \times Z_4$	$\mathbb{Z}_4^2$	$\mathbb{Z}_2^9$	$\mathbb{Z}_2^{11}$	$\mathbb{Z}_2^7 \times \mathbb{Z}_4^2$
$Z_6 \times Z_6$	$\mathbb{Z}_6^2$	$\mathbb{Z}_2^9$	$\mathbb{Z}_2^{11}$	$\mathbb{Z}_2^7 \times \mathbb{Z}_6^2$
$U(1)$				$\mathbb{Z} \times \mathbb{Z}_2^5$
$SU(2)$				$\mathbb{Z}_2^3$
$SO(3)$			$\mathbb{Z}_2^5$	$\mathbb{Z}_2^3$
Space group #92 $P4_12_12$				
$\varnothing$				$\mathbb{Z}_2$
Z_2^T	$\mathbb{Z}_2$		$\mathbb{Z}_2^3$	$\mathbb{Z}_2$
Z_2		$\mathbb{Z}_2^2$	$\mathbb{Z}_2$	$\mathbb{Z}_2^2$
$Z_2 \times Z_2^T$	$\mathbb{Z}_2^3$	$\mathbb{Z}_2^4$	$\mathbb{Z}_2^6$	$\mathbb{Z}_2^2$
Z_4		$\mathbb{Z}_2 \times \mathbb{Z}_4$	$\mathbb{Z}_2$	$\mathbb{Z}_2 \times \mathbb{Z}_4$
Z_6		$\mathbb{Z}_2^2$	$\mathbb{Z}_2$	$\mathbb{Z}_2 \times \mathbb{Z}_6$
$Z_2 \times Z_2$	$\mathbb{Z}_2^2$	$\mathbb{Z}_2^6$	$\mathbb{Z}_2^4$	$\mathbb{Z}_2^3$
$Z_4 \times Z_4$	$\mathbb{Z}_4^2$	$\mathbb{Z}_2^3 \times \mathbb{Z}_4^3$	$\mathbb{Z}_2^3 \times \mathbb{Z}_4$	$\mathbb{Z}_2 \times \mathbb{Z}_4^2$
$Z_6 \times Z_6$	$\mathbb{Z}_6^2$	$\mathbb{Z}_2^6$	$\mathbb{Z}_2^4$	$\mathbb{Z}_2 \times \mathbb{Z}_6^2$
$U(1)$				$\mathbb{Z} \times \mathbb{Z}_2$
$SU(2)$				$\mathbb{Z}_2$
$SO(3)$			$\mathbb{Z}_2^2$	$\mathbb{Z}_2$
Space group #93 $P4_222$				
$\varnothing$				$\mathbb{Z}_2^{12}$
Z_2^T	$\mathbb{Z}_2$		$\mathbb{Z}_2^{18}$	$\mathbb{Z}_2^{12}$
Z_2		$\mathbb{Z}_2^4$	$\mathbb{Z}_2^9$	$\mathbb{Z}_2^{18}$
$Z_2 \times Z_2^T$	$\mathbb{Z}_2^3$	$\mathbb{Z}_2^8$	$\mathbb{Z}_2^{36}$	$\mathbb{Z}_2^{18}$
Z_4		$\mathbb{Z}_2^4$	$\mathbb{Z}_2^9$	$\mathbb{Z}_2^{17} \times \mathbb{Z}_4$
Z_6		$\mathbb{Z}_2^4$	$\mathbb{Z}_2^9$	$\mathbb{Z}_2^{17} \times \mathbb{Z}_6$
$Z_2 \times Z_2$	$\mathbb{Z}_2^2$	$\mathbb{Z}_2^{12}$	$\mathbb{Z}_2^{27}$	$\mathbb{Z}_2^{24}$
$Z_4 \times Z_4$	$\mathbb{Z}_4^2$	$\mathbb{Z}_2^{12}$	$\mathbb{Z}_2^{27}$	$\mathbb{Z}_2^{22} \times \mathbb{Z}_4^2$
$Z_6 \times Z_6$	$\mathbb{Z}_6^2$	$\mathbb{Z}_2^{12}$	$\mathbb{Z}_2^{27}$	$\mathbb{Z}_2^{22} \times \mathbb{Z}_6^2$
$U(1)$				$\mathbb{Z} \times \mathbb{Z}_2^{17}$
$SU(2)$				$\mathbb{Z}_2^{12}$
$SO(3)$			$\mathbb{Z}_2^9$	$\mathbb{Z}_2^{12}$
Space group #94 $P4_22_12$				
$\varnothing$				$\mathbb{Z}_2^5$
Z_2^T	$\mathbb{Z}_2$		$\mathbb{Z}_2^9$	$\mathbb{Z}_2^5$
Z_2		$\mathbb{Z}_2^3$	$\mathbb{Z}_2^4$	$\mathbb{Z}_2^8$
$Z_2 \times Z_2^T$	$\mathbb{Z}_2^3$	$\mathbb{Z}_2^6$	$\mathbb{Z}_2^{18}$	$\mathbb{Z}_2^8$
Z_4		$\mathbb{Z}_2^2 \times \mathbb{Z}_4$	$\mathbb{Z}_2^4$	$\mathbb{Z}_2^7 \times \mathbb{Z}_4$
Z_6		$\mathbb{Z}_2^3$	$\mathbb{Z}_2^4$	$\mathbb{Z}_2^7 \times \mathbb{Z}_6$
$Z_2 \times Z_2$	$\mathbb{Z}_2^2$	$\mathbb{Z}_2^9$	$\mathbb{Z}_2^{13}$	$\mathbb{Z}_2^{11}$
$Z_4 \times Z_4$	$\mathbb{Z}_4^2$	$\mathbb{Z}_2^6 \times \mathbb{Z}_4^3$	$\mathbb{Z}_2^{12} \times \mathbb{Z}_4$	$\mathbb{Z}_2^9 \times \mathbb{Z}_4^2$
$Z_6 \times Z_6$	$\mathbb{Z}_6^2$	$\mathbb{Z}_2^9$	$\mathbb{Z}_2^{13}$	$\mathbb{Z}_2^9 \times \mathbb{Z}_6^2$
$U(1)$				$\mathbb{Z} \times \mathbb{Z}_2^7$
$SU(2)$				$\mathbb{Z}_2^5$
$SO(3)$			$\mathbb{Z}_2^5$	$\mathbb{Z}_2^5$
Space group #95 $P4_322$				
$\varnothing$				$\mathbb{Z}_2^3$
Z_2^T	$\mathbb{Z}_2$		$\mathbb{Z}_2^8$	$\mathbb{Z}_2^3$

TABLE II (Continued.)

G_0	$E_{3,\infty}^3$	$E_{2,\infty}^2$	$E_{1,\infty}^1$	$E_{0,\infty}^0$
Z_2		$\mathbb{Z}_2^3$	$\mathbb{Z}_2^3$	$\mathbb{Z}_2^6$
$Z_2 \times Z_2^T$	$\mathbb{Z}_2^3$	$\mathbb{Z}_2^6$	$\mathbb{Z}_2^{16}$	$\mathbb{Z}_2^6$
Z_4		$\mathbb{Z}_2^3$	$\mathbb{Z}_2^3$	$\mathbb{Z}_2^5 \times \mathbb{Z}_4$
Z_6		$\mathbb{Z}_2^3$	$\mathbb{Z}_2^3$	$\mathbb{Z}_2^5 \times \mathbb{Z}_6$
$Z_2 \times Z_2$	$\mathbb{Z}_2^2$	$\mathbb{Z}_2^9$	$\mathbb{Z}_2^{11}$	$\mathbb{Z}_2^9$
$Z_4 \times Z_4$	$\mathbb{Z}_4^2$	$\mathbb{Z}_2^9$	$\mathbb{Z}_2^{11}$	$\mathbb{Z}_2^7 \times \mathbb{Z}_4^2$
$Z_6 \times Z_6$	$\mathbb{Z}_6^2$	$\mathbb{Z}_2^9$	$\mathbb{Z}_2^{11}$	$\mathbb{Z}_2^7 \times \mathbb{Z}_6^2$
$U(1)$				$\mathbb{Z} \times \mathbb{Z}_2^5$
$SU(2)$				$\mathbb{Z}_2^3$
$SO(3)$			$\mathbb{Z}_2^5$	$\mathbb{Z}_2^3$
Space group #96 $P4_32_12$				
$\varnothing$				$\mathbb{Z}_2$
Z_2^T	$\mathbb{Z}_2$		$\mathbb{Z}_2^3$	$\mathbb{Z}_2$
Z_2		$\mathbb{Z}_2^2$	$\mathbb{Z}_2$	$\mathbb{Z}_2^2$
$Z_2 \times Z_2^T$	$\mathbb{Z}_2^3$	$\mathbb{Z}_2^4$	$\mathbb{Z}_2^6$	$\mathbb{Z}_2^2$
Z_4		$\mathbb{Z}_2 \times \mathbb{Z}_4$	$\mathbb{Z}_2$	$\mathbb{Z}_2 \times \mathbb{Z}_4$
Z_6		$\mathbb{Z}_2^2$	$\mathbb{Z}_2$	$\mathbb{Z}_2 \times \mathbb{Z}_6$
$Z_2 \times Z_2$	$\mathbb{Z}_2^2$	$\mathbb{Z}_2^6$	$\mathbb{Z}_2^4$	$\mathbb{Z}_2^3$
$Z_4 \times Z_4$	$\mathbb{Z}_4^2$	$\mathbb{Z}_2^3 \times \mathbb{Z}_4^3$	$\mathbb{Z}_2^3 \times \mathbb{Z}_4$	$\mathbb{Z}_2 \times \mathbb{Z}_4^2$
$Z_6 \times Z_6$	$\mathbb{Z}_6^2$	$\mathbb{Z}_2^6$	$\mathbb{Z}_2^4$	$\mathbb{Z}_2 \times \mathbb{Z}_6^2$
$U(1)$				$\mathbb{Z} \times \mathbb{Z}_2$
$SU(2)$				$\mathbb{Z}_2$
$SO(3)$			$\mathbb{Z}_2^2$	$\mathbb{Z}_2$
Space group #97 $I422$				
$\varnothing$				$\mathbb{Z}_2^8$
Z_2^T	$\mathbb{Z}_2$		$\mathbb{Z}_2^{12}$	$\mathbb{Z}_2^8$
Z_2		$\mathbb{Z}_2^3$	$\mathbb{Z}_2^6$	$\mathbb{Z}_2^{12}$
$Z_2 \times Z_2^T$	$\mathbb{Z}_2^3$	$\mathbb{Z}_2^6$	$\mathbb{Z}_2^{24}$	$\mathbb{Z}_2^{12}$
Z_4		$\mathbb{Z}_2^3$	$\mathbb{Z}_2^5 \times \mathbb{Z}_4$	$\mathbb{Z}_2^{11} \times \mathbb{Z}_4$
Z_6		$\mathbb{Z}_2^3$	$\mathbb{Z}_2^6$	$\mathbb{Z}_2^{11} \times \mathbb{Z}_6$
$Z_2 \times Z_2$	$\mathbb{Z}_2^2$	$\mathbb{Z}_2^9$	$\mathbb{Z}_2^{18}$	$\mathbb{Z}_2^{16}$
$Z_4 \times Z_4$	$\mathbb{Z}_4^2$	$\mathbb{Z}_2^9$	$\mathbb{Z}_2^{16} \times \mathbb{Z}_4^2$	$\mathbb{Z}_2^{14} \times \mathbb{Z}_4^2$
$Z_6 \times Z_6$	$\mathbb{Z}_6^2$	$\mathbb{Z}_2^9$	$\mathbb{Z}_2^{18}$	$\mathbb{Z}_2^{14} \times \mathbb{Z}_6^2$
$U(1)$				$\mathbb{Z} \times \mathbb{Z}_2^{11}$
$SU(2)$				$\mathbb{Z}_2^8$
$SO(3)$			$\mathbb{Z}_2^6$	$\mathbb{Z}_2^8$
Space group #98 $I4_122$				
$\varnothing$				$\mathbb{Z}_2^5$
Z_2^T	$\mathbb{Z}_2$		$\mathbb{Z}_2^9$	$\mathbb{Z}_2^5$
Z_2		$\mathbb{Z}_2^3$	$\mathbb{Z}_2^4$	$\mathbb{Z}_2^8$
$Z_2 \times Z_2^T$	$\mathbb{Z}_2^3$	$\mathbb{Z}_2^6$	$\mathbb{Z}_2^{18}$	$\mathbb{Z}_2^8$
Z_4		$\mathbb{Z}_2^3$	$\mathbb{Z}_2^4$	$\mathbb{Z}_2^7 \times \mathbb{Z}_4$
Z_6		$\mathbb{Z}_2^3$	$\mathbb{Z}_2^4$	$\mathbb{Z}_2^7 \times \mathbb{Z}_6$
$Z_2 \times Z_2$	$\mathbb{Z}_2^2$	$\mathbb{Z}_2^9$	$\mathbb{Z}_2^{13}$	$\mathbb{Z}_2^{11}$
$Z_4 \times Z_4$	$\mathbb{Z}_4^2$	$\mathbb{Z}_2^9$	$\mathbb{Z}_2^{13}$	$\mathbb{Z}_2^9 \times \mathbb{Z}_4^2$
$Z_6 \times Z_6$	$\mathbb{Z}_6^2$	$\mathbb{Z}_2^9$	$\mathbb{Z}_2^{13}$	$\mathbb{Z}_2^9 \times \mathbb{Z}_6^2$
$U(1)$				$\mathbb{Z} \times \mathbb{Z}_2^7$
$SU(2)$				$\mathbb{Z}_2^5$
$SO(3)$			$\mathbb{Z}_2^5$	$\mathbb{Z}_2^5$

TABLE II (Continued.)

G_0	$E_{3,\infty}^{3}$	$E_{2,\infty}^{2}$	$E_{1,\infty}^{1}$	$E_{0,\infty}^{0}$
Space group #99 $P4mm$				
$\varnothing$		$\mathbb{Z}_2^{3}$	$\mathbb{Z}_2^{3}$	$\mathbb{Z}_2^{6}$
Z_2^T	$\mathbb{Z}_2$	$\mathbb{Z}_2^{6}$	$\mathbb{Z}_2^{18}$	$\mathbb{Z}_2^{6}$
Z_2		$\mathbb{Z}_2^{10}$	$\mathbb{Z}_2^{12}$	$\mathbb{Z}_2^{9}$
$Z_2 \times Z_2^T$	$\mathbb{Z}_2^{3}$	$\mathbb{Z}_2^{20}$	$\mathbb{Z}_2^{33}$	$\mathbb{Z}_2^{9}$
Z_4		$\mathbb{Z}_2^{9} \times \mathbb{Z}_4$	$\mathbb{Z}_2^{12}$	$\mathbb{Z}_2^{7} \times \mathbb{Z}_4^{2}$
Z_6		$\mathbb{Z}_2^{10}$	$\mathbb{Z}_2^{12}$	$\mathbb{Z}_2^{8} \times \mathbb{Z}_6$
$Z_2 \times Z_2$	$\mathbb{Z}_2^{2}$	$\mathbb{Z}_2^{24}$	$\mathbb{Z}_2^{27}$	$\mathbb{Z}_2^{12}$
$Z_4 \times Z_4$	$\mathbb{Z}_2^{2}$	$\mathbb{Z}_2^{21} \times \mathbb{Z}_4^{3}$	$\mathbb{Z}_2^{24} \times \mathbb{Z}_4^{3}$	$\mathbb{Z}_2^{8} \times \mathbb{Z}_4^{4}$
$Z_6 \times Z_6$	$\mathbb{Z}_2^{2}$	$\mathbb{Z}_2^{24}$	$\mathbb{Z}_2^{26} \times \mathbb{Z}_6$	$\mathbb{Z}_2^{10} \times \mathbb{Z}_6^{2}$
$U(1)$		$\mathbb{Z}_2^{7}$	$\mathbb{Z}_2^{3}$	$\mathbb{Z} \times \mathbb{Z}_2^{7} \times \mathbb{Z}_4$
$SU(2)$		$\mathbb{Z}_2^{4}$	$\mathbb{Z}_2^{3}$	$\mathbb{Z}_2^{6}$
$SO(3)$		$\mathbb{Z}_2^{7}$	$\mathbb{Z}_2^{9}$	$\mathbb{Z}_2^{6}$
Space group #100 $P4bm$				
$\varnothing$		$\mathbb{Z}_2$	$\mathbb{Z}_2$	$\mathbb{Z}_2^{2} \times \mathbb{Z}_4$
Z_2^T	$\mathbb{Z}_2$	$\mathbb{Z}_2^{2}$	$\mathbb{Z}_2^{9}$	$\mathbb{Z}_2^{3}$
Z_2		$\mathbb{Z}_2^{5}$	$\mathbb{Z}_2^{5}$	$\mathbb{Z}_2^{4} \times \mathbb{Z}_4$
$Z_2 \times Z_2^T$	$\mathbb{Z}_2^{3}$	$\mathbb{Z}_2^{10}$	$\mathbb{Z}_2^{17}$	$\mathbb{Z}_2^{5}$
Z_4		$\mathbb{Z}_2^{4} \times \mathbb{Z}_4$	$\mathbb{Z}_2^{4} \times \mathbb{Z}_4$	$\mathbb{Z}_2^{2} \times \mathbb{Z}_4^{3}$
Z_6		$\mathbb{Z}_2^{5}$	$\mathbb{Z}_2^{5}$	$\mathbb{Z}_2^{3} \times \mathbb{Z}_4 \times \mathbb{Z}_6$
$Z_2 \times Z_2$	$\mathbb{Z}_2^{2}$	$\mathbb{Z}_2^{13}$	$\mathbb{Z}_2^{13}$	$\mathbb{Z}_2^{6} \times \mathbb{Z}_4$
$Z_4 \times Z_4$	$\mathbb{Z}_2^{2}$	$\mathbb{Z}_2^{10} \times \mathbb{Z}_4^{3}$	$\mathbb{Z}_2^{8} \times \mathbb{Z}_4^{5}$	$\mathbb{Z}_2^{2} \times \mathbb{Z}_4^{5}$
$Z_6 \times Z_6$	$\mathbb{Z}_2^{2}$	$\mathbb{Z}_2^{13}$	$\mathbb{Z}_2^{12} \times \mathbb{Z}_6$	$\mathbb{Z}_2^{4} \times \mathbb{Z}_4 \times \mathbb{Z}_6^{2}$
$U(1)$		$\mathbb{Z}_2^{3}$	$\mathbb{Z}_2$	$\mathbb{Z} \times \mathbb{Z}_2^{2} \times \mathbb{Z}_4^{2}$
$SU(2)$		$\mathbb{Z}_2^{2}$	$\mathbb{Z}_2$	$\mathbb{Z}_2^{2} \times \mathbb{Z}_4$
$SO(3)$		$\mathbb{Z}_2^{3}$	$\mathbb{Z}_2^{5}$	$\mathbb{Z}_2^{2} \times \mathbb{Z}_4$
Space group #101 $P4_2cm$				
$\varnothing$		$\mathbb{Z}_2$	$\mathbb{Z}_2^{2}$	$\mathbb{Z}_2^{3}$
Z_2^T	$\mathbb{Z}_2$	$\mathbb{Z}_2^{2}$	$\mathbb{Z}_2^{11}$	$\mathbb{Z}_2^{3}$
Z_2		$\mathbb{Z}_2^{5}$	$\mathbb{Z}_2^{6}$	$\mathbb{Z}_2^{6}$
$Z_2 \times Z_2^T$	$\mathbb{Z}_2^{3}$	$\mathbb{Z}_2^{10}$	$\mathbb{Z}_2^{20}$	$\mathbb{Z}_2^{6}$
Z_4		$\mathbb{Z}_2^{4} \times \mathbb{Z}_4$	$\mathbb{Z}_2^{6}$	$\mathbb{Z}_2^{5} \times \mathbb{Z}_4$
Z_6		$\mathbb{Z}_2^{5}$	$\mathbb{Z}_2^{6}$	$\mathbb{Z}_2^{5} \times \mathbb{Z}_6$
$Z_2 \times Z_2$	$\mathbb{Z}_2^{2}$	$\mathbb{Z}_2^{13}$	$\mathbb{Z}_2^{15}$	$\mathbb{Z}_2^{9}$
$Z_4 \times Z_4$	$\mathbb{Z}_2^{2}$	$\mathbb{Z}_2^{10} \times \mathbb{Z}_4^{3}$	$\mathbb{Z}_2^{13} \times \mathbb{Z}_4^{2}$	$\mathbb{Z}_2^{7} \times \mathbb{Z}_4^{2}$
$Z_6 \times Z_6$	$\mathbb{Z}_2^{2}$	$\mathbb{Z}_2^{13}$	$\mathbb{Z}_2^{14} \times \mathbb{Z}_6$	$\mathbb{Z}_2^{7} \times \mathbb{Z}_6^{2}$
$U(1)$		$\mathbb{Z}_2^{3}$	$\mathbb{Z}_2^{2}$	$\mathbb{Z} \times \mathbb{Z}_2^{5}$
$SU(2)$		$\mathbb{Z}_2^{2}$	$\mathbb{Z}_2^{2}$	$\mathbb{Z}_2^{3}$
$SO(3)$		$\mathbb{Z}_2^{3}$	$\mathbb{Z}_2^{7}$	$\mathbb{Z}_2^{3}$
Space group #102 $P4_2cm$				
$\varnothing$		$\mathbb{Z}_2$	$\mathbb{Z}_2$	$\mathbb{Z}_2^{3}$
Z_2^T	$\mathbb{Z}_2$	$\mathbb{Z}_2^{2}$	$\mathbb{Z}_2^{9}$	$\mathbb{Z}_2^{3}$
Z_2		$\mathbb{Z}_2^{5}$	$\mathbb{Z}_2^{5}$	$\mathbb{Z}_2^{5}$
$Z_2 \times Z_2^T$	$\mathbb{Z}_2^{3}$	$\mathbb{Z}_2^{10}$	$\mathbb{Z}_2^{17}$	$\mathbb{Z}_2^{5}$
Z_4		$\mathbb{Z}_2^{4} \times \mathbb{Z}_4$	$\mathbb{Z}_2^{5}$	$\mathbb{Z}_2^{4} \times \mathbb{Z}_4$
Z_6		$\mathbb{Z}_2^{5}$	$\mathbb{Z}_2^{5}$	$\mathbb{Z}_2^{4} \times \mathbb{Z}_6$
$Z_2 \times Z_2$	$\mathbb{Z}_2^{2}$	$\mathbb{Z}_2^{13}$	$\mathbb{Z}_2^{13}$	$\mathbb{Z}_2^{7}$
$Z_4 \times Z_4$	$\mathbb{Z}_2^{2}$	$\mathbb{Z}_2^{10} \times \mathbb{Z}_4^{3}$	$\mathbb{Z}_2^{11} \times \mathbb{Z}_4^{2}$	$\mathbb{Z}_2^{5} \times \mathbb{Z}_4^{2}$
$Z_6 \times Z_6$	$\mathbb{Z}_2^{2}$	$\mathbb{Z}_2^{13}$	$\mathbb{Z}_2^{12} \times \mathbb{Z}_6$	$\mathbb{Z}_2^{5} \times \mathbb{Z}_6^{2}$

TABLE II (Continued.)

G_0	$E_{3,\infty}^{3}$	$E_{2,\infty}^{2}$	$E_{1,\infty}^{1}$	$E_{0,\infty}^{0}$
$U(1)$		$\mathbb{Z}_2^{3}$	$\mathbb{Z}_2$	$\mathbb{Z} \times \mathbb{Z}_2^{4}$
$SU(2)$		$\mathbb{Z}_2^{2}$	$\mathbb{Z}_2$	$\mathbb{Z}_2^{3}$
$SO(3)$		$\mathbb{Z}_2^{3}$	$\mathbb{Z}_2^{5}$	$\mathbb{Z}_2^{3}$
Space group #103 $P4cc$				
$\varnothing$				$\mathbb{Z}_2^{3}$
Z_2^T	$\mathbb{Z}_2$		$\mathbb{Z}_2^{8}$	$\mathbb{Z}_2^{3}$
Z_2		$\mathbb{Z}_2^{3}$	$\mathbb{Z}_2^{3}$	$\mathbb{Z}_2^{6}$
$Z_2 \times Z_2^T$	$\mathbb{Z}_2^{3}$	$\mathbb{Z}_2^{6}$	$\mathbb{Z}_2^{16}$	$\mathbb{Z}_2^{6}$
Z_4		$\mathbb{Z}_2^{2} \times \mathbb{Z}_4$	$\mathbb{Z}_2^{3}$	$\mathbb{Z}_2^{4} \times \mathbb{Z}_4^{2}$
Z_6		$\mathbb{Z}_2^{3}$	$\mathbb{Z}_2^{3}$	$\mathbb{Z}_2^{5} \times \mathbb{Z}_6$
$Z_2 \times Z_2$	$\mathbb{Z}_2^{2}$	$\mathbb{Z}_2^{9}$	$\mathbb{Z}_2^{11}$	$\mathbb{Z}_2^{9}$
$Z_4 \times Z_4$	$\mathbb{Z}_2^{2}$	$\mathbb{Z}_2^{6} \times \mathbb{Z}_4^{3}$	$\mathbb{Z}_2^{8} \times \mathbb{Z}_4^{3}$	$\mathbb{Z}_2^{5} \times \mathbb{Z}_4^{4}$
$Z_6 \times Z_6$	$\mathbb{Z}_2^{2}$	$\mathbb{Z}_2^{9}$	$\mathbb{Z}_2^{10} \times \mathbb{Z}_6$	$\mathbb{Z}_2^{7} \times \mathbb{Z}_6^{2}$
$U(1)$		$\mathbb{Z}_2$		$\mathbb{Z} \times \mathbb{Z}_2^{4} \times \mathbb{Z}_4$
$SU(2)$		$\mathbb{Z}_2$		$\mathbb{Z}_2^{3}$
$SO(3)$		$\mathbb{Z}_2$	$\mathbb{Z}_2^{5}$	$\mathbb{Z}_2^{3}$
Space group #104 $P4nc$				
$\varnothing$				$\mathbb{Z}_2 \times \mathbb{Z}_4$
Z_2^T	$\mathbb{Z}_2$		$\mathbb{Z}_2^{5}$	$\mathbb{Z}_2^{2}$
Z_2		$\mathbb{Z}_2^{2}$	$\mathbb{Z}_2^{2}$	$\mathbb{Z}_2^{3} \times \mathbb{Z}_4$
$Z_2 \times Z_2^T$	$\mathbb{Z}_2^{3}$	$\mathbb{Z}_2^{4}$	$\mathbb{Z}_2^{10}$	$\mathbb{Z}_2^{4}$
Z_4		$\mathbb{Z}_2 \times \mathbb{Z}_4$	$\mathbb{Z}_2 \times \mathbb{Z}_4$	$\mathbb{Z}_2^{2} \times \mathbb{Z}_4^{2}$
Z_6		$\mathbb{Z}_2^{2}$	$\mathbb{Z}_2^{2}$	$\mathbb{Z}_2^{2} \times \mathbb{Z}_4 \times \mathbb{Z}_6$
$Z_2 \times Z_2$	$\mathbb{Z}_2^{2}$	$\mathbb{Z}_2^{6}$	$\mathbb{Z}_2^{7}$	$\mathbb{Z}_2^{5} \times \mathbb{Z}_4$
$Z_4 \times Z_4$	$\mathbb{Z}_2^{2}$	$\mathbb{Z}_2^{3} \times \mathbb{Z}_4^{3}$	$\mathbb{Z}_2^{3} \times \mathbb{Z}_4^{4}$	$\mathbb{Z}_2^{3} \times \mathbb{Z}_4^{3}$
$Z_6 \times Z_6$	$\mathbb{Z}_2^{2}$	$\mathbb{Z}_2^{6}$	$\mathbb{Z}_2^{6} \times \mathbb{Z}_6$	$\mathbb{Z}_2^{3} \times \mathbb{Z}_4 \times \mathbb{Z}_6^{2}$
$U(1)$		$\mathbb{Z}_2$		$\mathbb{Z} \times \mathbb{Z}_2^{2} \times \mathbb{Z}_4$
$SU(2)$		$\mathbb{Z}_2$		$\mathbb{Z}_2 \times \mathbb{Z}_4$
$SO(3)$		$\mathbb{Z}_2$	$\mathbb{Z}_2^{3}$	$\mathbb{Z}_2 \times \mathbb{Z}_4$
Space group #105 $P4_2mc$				
$\varnothing$		$\mathbb{Z}_2^{2}$	$\mathbb{Z}_2^{3}$	$\mathbb{Z}_2^{4}$
Z_2^T	$\mathbb{Z}_2$	$\mathbb{Z}_2^{4}$	$\mathbb{Z}_2^{14}$	$\mathbb{Z}_2^{4}$
Z_2		$\mathbb{Z}_2^{7}$	$\mathbb{Z}_2^{9}$	$\mathbb{Z}_2^{7}$
$Z_2 \times Z_2^T$	$\mathbb{Z}_2^{3}$	$\mathbb{Z}_2^{14}$	$\mathbb{Z}_2^{25}$	$\mathbb{Z}_2^{7}$
Z_4		$\mathbb{Z}_2^{6} \times \mathbb{Z}_4$	$\mathbb{Z}_2^{9}$	$\mathbb{Z}_2^{6} \times \mathbb{Z}_4$
Z_6		$\mathbb{Z}_2^{7}$	$\mathbb{Z}_2^{9}$	$\mathbb{Z}_2^{6} \times \mathbb{Z}_6$
$Z_2 \times Z_2$	$\mathbb{Z}_2^{2}$	$\mathbb{Z}_2^{17}$	$\mathbb{Z}_2^{20}$	$\mathbb{Z}_2^{10}$
$Z_4 \times Z_4$	$\mathbb{Z}_2^{2}$	$\mathbb{Z}_2^{14} \times \mathbb{Z}_4^{3}$	$\mathbb{Z}_2^{18} \times \mathbb{Z}_4^{2}$	$\mathbb{Z}_2^{8} \times \mathbb{Z}_4^{2}$
$Z_6 \times Z_6$	$\mathbb{Z}_2^{2}$	$\mathbb{Z}_2^{17}$	$\mathbb{Z}_2^{19} \times \mathbb{Z}_6$	$\mathbb{Z}_2^{8} \times \mathbb{Z}_6^{2}$
$U(1)$		$\mathbb{Z}_2^{5}$	$\mathbb{Z}_2^{3}$	$\mathbb{Z} \times \mathbb{Z}_2^{6}$
$SU(2)$		$\mathbb{Z}_2^{3}$	$\mathbb{Z}_2^{3}$	$\mathbb{Z}_2^{4}$
$SO(3)$		$\mathbb{Z}_2^{5}$	$\mathbb{Z}_2^{8}$	$\mathbb{Z}_2^{4}$
Space group #106 $P4_2bc$				
$\varnothing$				$\mathbb{Z}_2^{2}$
Z_2^T	$\mathbb{Z}_2$		$\mathbb{Z}_2^{5}$	$\mathbb{Z}_2^{2}$
Z_2		$\mathbb{Z}_2^{2}$	$\mathbb{Z}_2^{2}$	$\mathbb{Z}_2^{4}$
$Z_2 \times Z_2^T$	$\mathbb{Z}_2^{3}$	$\mathbb{Z}_2^{4}$	$\mathbb{Z}_2^{10}$	$\mathbb{Z}_2^{4}$
Z_4		$\mathbb{Z}_2 \times \mathbb{Z}_4$	$\mathbb{Z}_2^{2}$	$\mathbb{Z}_2^{3} \times \mathbb{Z}_4$
Z_6		$\mathbb{Z}_2^{2}$	$\mathbb{Z}_2^{2}$	$\mathbb{Z}_2^{3} \times \mathbb{Z}_6$

TABLE II (Continued.)

G_0	$E_{3,\infty}^3$	$E_{2,\infty}^2$	$E_{1,\infty}^1$	$E_{0,\infty}^0$
$Z_2 \times Z_2$	$\mathbb{Z}_2^2$	$\mathbb{Z}_2^6$	$\mathbb{Z}_2^7$	$\mathbb{Z}_2^6$
$Z_4 \times Z_4$	$\mathbb{Z}_2^2$	$\mathbb{Z}_2^3 \times \mathbb{Z}_4^3$	$\mathbb{Z}_2^5 \times \mathbb{Z}_4^2$	$\mathbb{Z}_2^4 \times \mathbb{Z}_4^2$
$Z_6 \times Z_6$	$\mathbb{Z}_2^2$	$\mathbb{Z}_2^6$	$\mathbb{Z}_2^6 \times \mathbb{Z}_6$	$\mathbb{Z}_2^4 \times \mathbb{Z}_6^2$
$U(1)$		$\mathbb{Z}_2$		$\mathbb{Z} \times \mathbb{Z}_2^3$
$SU(2)$		$\mathbb{Z}_2$		$\mathbb{Z}_2^2$
$SO(3)$		$\mathbb{Z}_2$	$\mathbb{Z}_2^3$	$\mathbb{Z}_2^2$
Space group #107 $I4mm$				
$\varnothing$		$\mathbb{Z}_2^2$	$\mathbb{Z}_2^2$	$\mathbb{Z}_2^3$
Z_2^T	$\mathbb{Z}_2$	$\mathbb{Z}_2^4$	$\mathbb{Z}_2^{11}$	$\mathbb{Z}_2^3$
Z_2		$\mathbb{Z}_2^7$	$\mathbb{Z}_2^7$	$\mathbb{Z}_2^5$
$Z_2 \times Z_2^T$	$\mathbb{Z}_2^3$	$\mathbb{Z}_2^{14}$	$\mathbb{Z}_2^{20}$	$\mathbb{Z}_2^5$
Z_4		$\mathbb{Z}_2^6 \times \mathbb{Z}_4$	$\mathbb{Z}_2^7$	$\mathbb{Z}_2^4 \times \mathbb{Z}_4$
Z_6		$\mathbb{Z}_2^7$	$\mathbb{Z}_2^7$	$\mathbb{Z}_2^4 \times \mathbb{Z}_6$
$Z_2 \times Z_2$	$\mathbb{Z}_2^2$	$\mathbb{Z}_2^{17}$	$\mathbb{Z}_2^{16}$	$\mathbb{Z}_2^7$
$Z_4 \times Z_4$	$\mathbb{Z}_2^2$	$\mathbb{Z}_2^{14} \times \mathbb{Z}_4^3$	$\mathbb{Z}_2^{14} \times \mathbb{Z}_4^2$	$\mathbb{Z}_2^5 \times \mathbb{Z}_4^2$
$Z_6 \times Z_6$	$\mathbb{Z}_2^2$	$\mathbb{Z}_2^{17}$	$\mathbb{Z}_2^{15} \times \mathbb{Z}_6$	$\mathbb{Z}_2^5 \times \mathbb{Z}_6^2$
$U(1)$		$\mathbb{Z}_2^5$	$\mathbb{Z}_2^2$	$\mathbb{Z} \times \mathbb{Z}_2^4$
$SU(2)$		$\mathbb{Z}_2^3$	$\mathbb{Z}_2^2$	$\mathbb{Z}_2^3$
$SO(3)$		$\mathbb{Z}_2^5$	$\mathbb{Z}_2^6$	$\mathbb{Z}_2^3$
Space group #108 $I4cm$				
$\varnothing$		$\mathbb{Z}_2$	$\mathbb{Z}_2$	$\mathbb{Z}_2^2$
Z_2^T	$\mathbb{Z}_2$	$\mathbb{Z}_2^2$	$\mathbb{Z}_2^8$	$\mathbb{Z}_2^2$
Z_2		$\mathbb{Z}_2^5$	$\mathbb{Z}_2^4$	$\mathbb{Z}_2^4$
$Z_2 \times Z_2^T$	$\mathbb{Z}_2^3$	$\mathbb{Z}_2^{10}$	$\mathbb{Z}_2^{15}$	$\mathbb{Z}_2^4$
Z_4		$\mathbb{Z}_2^4 \times \mathbb{Z}_4$	$\mathbb{Z}_2^4$	$\mathbb{Z}_2^2 \times \mathbb{Z}_4^2$
Z_6		$\mathbb{Z}_2^5$	$\mathbb{Z}_2^4$	$\mathbb{Z}_2^3 \times \mathbb{Z}_6$
$Z_2 \times Z_2$	$\mathbb{Z}_2^2$	$\mathbb{Z}_2^{13}$	$\mathbb{Z}_2^{11}$	$\mathbb{Z}_2^6$
$Z_4 \times Z_4$	$\mathbb{Z}_2^2$	$\mathbb{Z}_2^{10} \times \mathbb{Z}_4^3$	$\mathbb{Z}_2^8 \times \mathbb{Z}_4^3$	$\mathbb{Z}_2^2 \times \mathbb{Z}_4^4$
$Z_6 \times Z_6$	$\mathbb{Z}_2^2$	$\mathbb{Z}_2^{13}$	$\mathbb{Z}_2^{10} \times \mathbb{Z}_6$	$\mathbb{Z}_2^4 \times \mathbb{Z}_6^2$
$U(1)$		$\mathbb{Z}_2^3$	$\mathbb{Z}_2$	$\mathbb{Z} \times \mathbb{Z}_2^2 \times \mathbb{Z}_4$
$SU(2)$		$\mathbb{Z}_2^2$	$\mathbb{Z}_2$	$\mathbb{Z}_2^2$
$SO(3)$		$\mathbb{Z}_2^3$	$\mathbb{Z}_2^5$	$\mathbb{Z}_2^2$
Space group #109 $I4_1md$				
$\varnothing$		$\mathbb{Z}_2$	$\mathbb{Z}_2$	$\mathbb{Z}_2^2$
Z_2^T	$\mathbb{Z}_2$	$\mathbb{Z}_2^2$	$\mathbb{Z}_2^6$	$\mathbb{Z}_2^2$
Z_2		$\mathbb{Z}_2^4$	$\mathbb{Z}_2^4$	$\mathbb{Z}_2^3$
$Z_2 \times Z_2^T$	$\mathbb{Z}_2^3$	$\mathbb{Z}_2^8$	$\mathbb{Z}_2^{11}$	$\mathbb{Z}_2^3$
Z_4		$\mathbb{Z}_2^3 \times \mathbb{Z}_4$	$\mathbb{Z}_2^4$	$\mathbb{Z}_2^2 \times \mathbb{Z}_4$
Z_6		$\mathbb{Z}_2^4$	$\mathbb{Z}_2^4$	$\mathbb{Z}_2^2 \times \mathbb{Z}_6$
$Z_2 \times Z_2$	$\mathbb{Z}_2^2$	$\mathbb{Z}_2^{10}$	$\mathbb{Z}_2^9$	$\mathbb{Z}_2^4$
$Z_4 \times Z_4$	$\mathbb{Z}_2^2$	$\mathbb{Z}_2^7 \times \mathbb{Z}_4^3$	$\mathbb{Z}_2^7 \times \mathbb{Z}_4^2$	$\mathbb{Z}_2^2 \times \mathbb{Z}_4^2$
$Z_6 \times Z_6$	$\mathbb{Z}_2^2$	$\mathbb{Z}_2^{10}$	$\mathbb{Z}_2^8 \times \mathbb{Z}_6$	$\mathbb{Z}_2^2 \times \mathbb{Z}_6^2$
$U(1)$		$\mathbb{Z}_2^3$	$\mathbb{Z}_2$	$\mathbb{Z} \times \mathbb{Z}_2^2$
$SU(2)$		$\mathbb{Z}_2^2$	$\mathbb{Z}_2$	$\mathbb{Z}_2^2$
$SO(3)$		$\mathbb{Z}_2^3$	$\mathbb{Z}_2^3$	$\mathbb{Z}_2^2$
Space group #110 $I4_1cd$				
$\varnothing$				$\mathbb{Z}_2$
Z_2^T	$\mathbb{Z}_2$		$\mathbb{Z}_2^3$	$\mathbb{Z}_2$
Z_2		$\mathbb{Z}_2^2$	$\mathbb{Z}_2$	$\mathbb{Z}_2^2$

TABLE II (Continued.)

G_0	$E_{3,\infty}^3$	$E_{2,\infty}^2$	$E_{1,\infty}^1$	$E_{0,\infty}^0$
$Z_2 \times Z_2^T$	$\mathbb{Z}_2^3$	$\mathbb{Z}_2^4$	$\mathbb{Z}_2^6$	$\mathbb{Z}_2^2$
Z_4		$\mathbb{Z}_2 \times \mathbb{Z}_4$	$\mathbb{Z}_2$	$\mathbb{Z}_2 \times \mathbb{Z}_4$
Z_6		$\mathbb{Z}_2^2$	$\mathbb{Z}_2$	$\mathbb{Z}_2 \times \mathbb{Z}_6$
$Z_2 \times Z_2$	$\mathbb{Z}_2^2$	$\mathbb{Z}_2^6$	$\mathbb{Z}_2^4$	$\mathbb{Z}_2^3$
$Z_4 \times Z_4$	$\mathbb{Z}_2^2$	$\mathbb{Z}_2^3 \times \mathbb{Z}_4^3$	$\mathbb{Z}_2^2 \times \mathbb{Z}_4^2$	$\mathbb{Z}_2 \times \mathbb{Z}_4^2$
$Z_6 \times Z_6$	$\mathbb{Z}_2^2$	$\mathbb{Z}_2^6$	$\mathbb{Z}_2^3 \times \mathbb{Z}_6$	$\mathbb{Z}_2 \times \mathbb{Z}_6^2$
$U(1)$		$\mathbb{Z}_2$		$\mathbb{Z} \times \mathbb{Z}_2$
$SU(2)$		$\mathbb{Z}_2$		$\mathbb{Z}_2$
$SO(3)$		$\mathbb{Z}_2$	$\mathbb{Z}_2^2$	$\mathbb{Z}_2$
Space group #111 $P\bar{4}2m$				
$\varnothing$		$\mathbb{Z}_2$	$\mathbb{Z}_2^2$	$\mathbb{Z}_2^{10}$
Z_2^T	$\mathbb{Z}_2$	$\mathbb{Z}_2^2$	$\mathbb{Z}_2^{18}$	$\mathbb{Z}_2^{10}$
Z_2		$\mathbb{Z}_2^6$	$\mathbb{Z}_2^{10}$	$\mathbb{Z}_2^{16}$
$Z_2 \times Z_2^T$	$\mathbb{Z}_2^3$	$\mathbb{Z}_2^{12}$	$\mathbb{Z}_2^{34}$	$\mathbb{Z}_2^{16}$
Z_4		$\mathbb{Z}_2^6$	$\mathbb{Z}_2^{10}$	$\mathbb{Z}_2^{14} \times \mathbb{Z}_4^2$
Z_6		$\mathbb{Z}_2^6$	$\mathbb{Z}_2^{10}$	$\mathbb{Z}_2^{15} \times \mathbb{Z}_6$
$Z_2 \times Z_2$	$\mathbb{Z}_2^2$	$\mathbb{Z}_2^{16}$	$\mathbb{Z}_2^{26}$	$\mathbb{Z}_2^{22}$
$Z_4 \times Z_4$	$\mathbb{Z}_2^2$	$\mathbb{Z}_2^{16}$	$\mathbb{Z}_2^{25} \times \mathbb{Z}_4$	$\mathbb{Z}_2^{18} \times \mathbb{Z}_4^4$
$Z_6 \times Z_6$	$\mathbb{Z}_2^2$	$\mathbb{Z}_2^{16}$	$\mathbb{Z}_2^{26}$	$\mathbb{Z}_2^{20} \times \mathbb{Z}_6^2$
$U(1)$		$\mathbb{Z}_2^3$	$\mathbb{Z}_2^2$	$\mathbb{Z} \times \mathbb{Z}_2^{14} \times \mathbb{Z}_4$
$SU(2)$		$\mathbb{Z}_2^2$	$\mathbb{Z}_2^2$	$\mathbb{Z}_2^{10}$
$SO(3)$		$\mathbb{Z}_2^3$	$\mathbb{Z}_2^{10}$	$\mathbb{Z}_2^{10}$
Space group #112 $P\bar{4}2c$				
$\varnothing$				$\mathbb{Z}_2^{10}$
Z_2^T	$\mathbb{Z}_2$		$\mathbb{Z}_2^{14}$	$\mathbb{Z}_2^8$
Z_2		$\mathbb{Z}_2^3$	$\mathbb{Z}_2^5$	$\mathbb{Z}_2^{16}$
$Z_2 \times Z_2^T$	$\mathbb{Z}_2^3$	$\mathbb{Z}_2^6$	$\mathbb{Z}_2^{26}$	$\mathbb{Z}_2^{14}$
Z_4		$\mathbb{Z}_2^3$	$\mathbb{Z}_2^5$	$\mathbb{Z}_2^{15} \times \mathbb{Z}_4$
Z_6		$\mathbb{Z}_2^3$	$\mathbb{Z}_2^5$	$\mathbb{Z}_2^{15} \times \mathbb{Z}_6$
$Z_2 \times Z_2$	$\mathbb{Z}_2^2$	$\mathbb{Z}_2^9$	$\mathbb{Z}_2^{17}$	$\mathbb{Z}_2^{22}$
$Z_4 \times Z_4$	$\mathbb{Z}_2^2$	$\mathbb{Z}_2^9$	$\mathbb{Z}_2^{17}$	$\mathbb{Z}_2^{20} \times \mathbb{Z}_4^2$
$Z_6 \times Z_6$	$\mathbb{Z}_2^2$	$\mathbb{Z}_2^9$	$\mathbb{Z}_2^{17}$	$\mathbb{Z}_2^{20} \times \mathbb{Z}_6^2$
$U(1)$		$\mathbb{Z}_2$		$\mathbb{Z} \times \mathbb{Z}_2^{15}$
$SU(2)$		$\mathbb{Z}_2$		$\mathbb{Z}_2^{10}$
$SO(3)$		$\mathbb{Z}_2$	$\mathbb{Z}_2^7$	$\mathbb{Z}_2^{10}$
Space group #113 $P\bar{4}2_1m$				
$\varnothing$		$\mathbb{Z}_2$	$\mathbb{Z}_2$	$\mathbb{Z}_2^3 \times \mathbb{Z}_4$
Z_2^T	$\mathbb{Z}_2$	$\mathbb{Z}_2^2$	$\mathbb{Z}_2^9$	$\mathbb{Z}_2^3$
Z_2		$\mathbb{Z}_2^5$	$\mathbb{Z}_2^4$	$\mathbb{Z}_2^6 \times \mathbb{Z}_4$
$Z_2 \times Z_2^T$	$\mathbb{Z}_2^3$	$\mathbb{Z}_2^{10}$	$\mathbb{Z}_2^{16}$	$\mathbb{Z}_2^6$
Z_4		$\mathbb{Z}_2^4 \times \mathbb{Z}_4$	$\mathbb{Z}_2^4$	$\mathbb{Z}_2^4 \times \mathbb{Z}_4^3$
Z_6		$\mathbb{Z}_2^5$	$\mathbb{Z}_2^4$	$\mathbb{Z}_2^5 \times \mathbb{Z}_4 \times \mathbb{Z}_6$
$Z_2 \times Z_2$	$\mathbb{Z}_2^2$	$\mathbb{Z}_2^{13}$	$\mathbb{Z}_2^{11}$	$\mathbb{Z}_2^9 \times \mathbb{Z}_4$
$Z_4 \times Z_4$	$\mathbb{Z}_2^2$	$\mathbb{Z}_2^{10} \times \mathbb{Z}_4^3$	$\mathbb{Z}_2^9 \times \mathbb{Z}_4^2$	$\mathbb{Z}_2^5 \times \mathbb{Z}_4^5$
$Z_6 \times Z_6$	$\mathbb{Z}_2^2$	$\mathbb{Z}_2^{13}$	$\mathbb{Z}_2^{11}$	$\mathbb{Z}_2^7 \times \mathbb{Z}_4 \times \mathbb{Z}_6^2$
$U(1)$		$\mathbb{Z}_2^3$	$\mathbb{Z}_2$	$\mathbb{Z} \times \mathbb{Z}_2^4 \times \mathbb{Z}_4^2$
$SU(2)$		$\mathbb{Z}_2^2$	$\mathbb{Z}_2$	$\mathbb{Z}_2^3 \times \mathbb{Z}_4$
$SO(3)$		$\mathbb{Z}_2^3$	$\mathbb{Z}_2^5$	$\mathbb{Z}_2^3 \times \mathbb{Z}_4$
Space group #114 $P\bar{4}2_1c$				

TABLE II (Continued.)

G_0	$E^3_{3,\infty}$	$E^2_{2,\infty}$	$E^1_{1,\infty}$	$E^0_{0,\infty}$
$\varnothing$				$\mathbb{Z}_2^2 \times \mathbb{Z}_4$
Z_2^T	$\mathbb{Z}_2$		$\mathbb{Z}_2^5$	$\mathbb{Z}_2^2$
Z_2		$\mathbb{Z}_2^2$	$\mathbb{Z}_2$	$\mathbb{Z}_2^5 \times \mathbb{Z}_4$
$Z_2 \times Z_2^T$	$\mathbb{Z}_2^3$	$\mathbb{Z}_2^4$	$\mathbb{Z}_2^9$	$\mathbb{Z}_2^5$
Z_4		$\mathbb{Z}_2 \times \mathbb{Z}_4$	$\mathbb{Z}_2$	$\mathbb{Z}_2^4 \times \mathbb{Z}_4^2$
Z_6		$\mathbb{Z}_2^2$	$\mathbb{Z}_2$	$\mathbb{Z}_2^4 \times \mathbb{Z}_4 \times \mathbb{Z}_6$
$Z_2 \times Z_2$	$\mathbb{Z}_2^2$	$\mathbb{Z}_2^6$	$\mathbb{Z}_2^5$	$\mathbb{Z}_2^8 \times \mathbb{Z}_4$
$Z_4 \times Z_4$	$\mathbb{Z}_2^2$	$\mathbb{Z}_2^3 \times \mathbb{Z}_4^3$	$\mathbb{Z}_2^4 \times \mathbb{Z}_4$	$\mathbb{Z}_2^6 \times \mathbb{Z}_4^3$
$Z_6 \times Z_6$	$\mathbb{Z}_2^2$	$\mathbb{Z}_2^6$	$\mathbb{Z}_2^5$	$\mathbb{Z}_2^6 \times \mathbb{Z}_4 \times \mathbb{Z}_6^2$
$U(1)$		$\mathbb{Z}_2$		$\mathbb{Z} \times \mathbb{Z}_2^4 \times \mathbb{Z}_4$
$SU(2)$		$\mathbb{Z}_2$		$\mathbb{Z}_2^2 \times \mathbb{Z}_4$
$SO(3)$		$\mathbb{Z}_2$	$\mathbb{Z}_2^3$	$\mathbb{Z}_2^2 \times \mathbb{Z}_4$
Space group #115 $P\bar{4}m2$				
$\varnothing$		$\mathbb{Z}_2^2$	$\mathbb{Z}_2^3$	$\mathbb{Z}_2^8$
Z_2^T	$\mathbb{Z}_2$	$\mathbb{Z}_2^4$	$\mathbb{Z}_2^{18}$	$\mathbb{Z}_2^8$
Z_2		$\mathbb{Z}_2^8$	$\mathbb{Z}_2^{11}$	$\mathbb{Z}_2^{13}$
$Z_2 \times Z_2^T$	$\mathbb{Z}_2^3$	$\mathbb{Z}_2^{16}$	$\mathbb{Z}_2^{33}$	$\mathbb{Z}_2^{13}$
Z_4		$\mathbb{Z}_2^8$	$\mathbb{Z}_2^{11}$	$\mathbb{Z}_2^{11} \times \mathbb{Z}_4^2$
Z_6		$\mathbb{Z}_2^8$	$\mathbb{Z}_2^{11}$	$\mathbb{Z}_2^{12} \times \mathbb{Z}_6$
$Z_2 \times Z_2$	$\mathbb{Z}_2^2$	$\mathbb{Z}_2^{20}$	$\mathbb{Z}_2^{26}$	$\mathbb{Z}_2^{18}$
$Z_4 \times Z_4$	$\mathbb{Z}_2^2$	$\mathbb{Z}_2^{20}$	$\mathbb{Z}_2^{25} \times \mathbb{Z}_4$	$\mathbb{Z}_2^{14} \times \mathbb{Z}_4^4$
$Z_6 \times Z_6$	$\mathbb{Z}_2^2$	$\mathbb{Z}_2^{20}$	$\mathbb{Z}_2^{26}$	$\mathbb{Z}_2^{16} \times \mathbb{Z}_6^2$
$U(1)$		$\mathbb{Z}_2^5$	$\mathbb{Z}_2^3$	$\mathbb{Z} \times \mathbb{Z}_2^{11} \times \mathbb{Z}_4$
$SU(2)$		$\mathbb{Z}_2^3$	$\mathbb{Z}_2^3$	$\mathbb{Z}_2^8$
$SO(3)$		$\mathbb{Z}_2^5$	$\mathbb{Z}_2^{10}$	$\mathbb{Z}_2^8$
Space group #116 $P\bar{4}c2$				
$\varnothing$				$\mathbb{Z}_2^7$
Z_2^T	$\mathbb{Z}_2$		$\mathbb{Z}_2^{11}$	$\mathbb{Z}_2^5$
Z_2		$\mathbb{Z}_2^3$	$\mathbb{Z}_2^3$	$\mathbb{Z}_2^{12}$
$Z_2 \times Z_2^T$	$\mathbb{Z}_2^3$	$\mathbb{Z}_2^6$	$\mathbb{Z}_2^{20}$	$\mathbb{Z}_2^{10}$
Z_4		$\mathbb{Z}_2^3$	$\mathbb{Z}_2^3$	$\mathbb{Z}_2^{11} \times \mathbb{Z}_4$
Z_6		$\mathbb{Z}_2^3$	$\mathbb{Z}_2^3$	$\mathbb{Z}_2^{11} \times \mathbb{Z}_6$
$Z_2 \times Z_2$	$\mathbb{Z}_2^2$	$\mathbb{Z}_2^9$	$\mathbb{Z}_2^{12}$	$\mathbb{Z}_2^{17}$
$Z_4 \times Z_4$	$\mathbb{Z}_2^2$	$\mathbb{Z}_2^9$	$\mathbb{Z}_2^{12}$	$\mathbb{Z}_2^{15} \times \mathbb{Z}_4^2$
$Z_6 \times Z_6$	$\mathbb{Z}_2^2$	$\mathbb{Z}_2^9$	$\mathbb{Z}_2^{12}$	$\mathbb{Z}_2^{15} \times \mathbb{Z}_6^2$
$U(1)$		$\mathbb{Z}_2$		$\mathbb{Z} \times \mathbb{Z}_2^{11}$
$SU(2)$		$\mathbb{Z}_2$		$\mathbb{Z}_2^7$
$SO(3)$		$\mathbb{Z}_2$	$\mathbb{Z}_2^6$	$\mathbb{Z}_2^7$
Space group #117 $P\bar{4}b2$				
$\varnothing$				$\mathbb{Z}_2^5 \times \mathbb{Z}_4$
Z_2^T	$\mathbb{Z}_2$		$\mathbb{Z}_2^9$	$\mathbb{Z}_2^5$
Z_2		$\mathbb{Z}_2^3$	$\mathbb{Z}_2^3$	$\mathbb{Z}_2^9 \times \mathbb{Z}_4$
$Z_2 \times Z_2^T$	$\mathbb{Z}_2^3$	$\mathbb{Z}_2^6$	$\mathbb{Z}_2^{17}$	$\mathbb{Z}_2^9$
Z_4		$\mathbb{Z}_2^3$	$\mathbb{Z}_2^3$	$\mathbb{Z}_2^7 \times \mathbb{Z}_4^3$
Z_6		$\mathbb{Z}_2^3$	$\mathbb{Z}_2^3$	$\mathbb{Z}_2^8 \times \mathbb{Z}_4 \times \mathbb{Z}_6$
$Z_2 \times Z_2$	$\mathbb{Z}_2^2$	$\mathbb{Z}_2^9$	$\mathbb{Z}_2^{11}$	$\mathbb{Z}_2^{13} \times \mathbb{Z}_4$
$Z_4 \times Z_4$	$\mathbb{Z}_2^2$	$\mathbb{Z}_2^9$	$\mathbb{Z}_2^{10} \times \mathbb{Z}_4$	$\mathbb{Z}_2^9 \times \mathbb{Z}_4^5$
$Z_6 \times Z_6$	$\mathbb{Z}_2^2$	$\mathbb{Z}_2^9$	$\mathbb{Z}_2^{11}$	$\mathbb{Z}_2^{11} \times \mathbb{Z}_4 \times \mathbb{Z}_6^2$
$U(1)$		$\mathbb{Z}_2$		$\mathbb{Z} \times \mathbb{Z}_2^7 \times \mathbb{Z}_4^2$

TABLE II (Continued.)

G_0	$E^3_{3,\infty}$	$E^2_{2,\infty}$	$E^1_{1,\infty}$	$E^0_{0,\infty}$
$SU(2)$		$\mathbb{Z}_2$		$\mathbb{Z}_2^5 \times \mathbb{Z}_4$
$SO(3)$		$\mathbb{Z}_2$	$\mathbb{Z}_2^5$	$\mathbb{Z}_2^5 \times \mathbb{Z}_4$
Space group #118 $P\bar{4}n2$				
$\varnothing$				$\mathbb{Z}_2^5 \times \mathbb{Z}_4$
Z_2^T	$\mathbb{Z}_2$		$\mathbb{Z}_2^9$	$\mathbb{Z}_2^5$
Z_2		$\mathbb{Z}_2^3$	$\mathbb{Z}_2^3$	$\mathbb{Z}_2^9 \times \mathbb{Z}_4$
$Z_2 \times Z_2^T$	$\mathbb{Z}_2^3$	$\mathbb{Z}_2^6$	$\mathbb{Z}_2^{17}$	$\mathbb{Z}_2^9$
Z_4		$\mathbb{Z}_2^3$	$\mathbb{Z}_2^3$	$\mathbb{Z}_2^7 \times \mathbb{Z}_4^3$
Z_6		$\mathbb{Z}_2^3$	$\mathbb{Z}_2^3$	$\mathbb{Z}_2^8 \times \mathbb{Z}_4 \times \mathbb{Z}_6$
$Z_2 \times Z_2$	$\mathbb{Z}_2^2$	$\mathbb{Z}_2^9$	$\mathbb{Z}_2^{11}$	$\mathbb{Z}_2^{13} \times \mathbb{Z}_4$
$Z_4 \times Z_4$	$\mathbb{Z}_2^2$	$\mathbb{Z}_2^9$	$\mathbb{Z}_2^{10} \times \mathbb{Z}_4$	$\mathbb{Z}_2^9 \times \mathbb{Z}_4^5$
$Z_6 \times Z_6$	$\mathbb{Z}_2^2$	$\mathbb{Z}_2^9$	$\mathbb{Z}_2^{11}$	$\mathbb{Z}_2^{11} \times \mathbb{Z}_4 \times \mathbb{Z}_6^2$
$U(1)$		$\mathbb{Z}_2$		$\mathbb{Z} \times \mathbb{Z}_2^7 \times \mathbb{Z}_4^2$
$SU(2)$		$\mathbb{Z}_2$		$\mathbb{Z}_2^5 \times \mathbb{Z}_4$
$SO(3)$		$\mathbb{Z}_2$	$\mathbb{Z}_2^5$	$\mathbb{Z}_2^5 \times \mathbb{Z}_4$
Space group #119 $I\bar{4}m2$				
$\varnothing$		$\mathbb{Z}_2$	$\mathbb{Z}_2^2$	$\mathbb{Z}_2^6$
Z_2^T	$\mathbb{Z}_2$	$\mathbb{Z}_2^2$	$\mathbb{Z}_2^{12}$	$\mathbb{Z}_2^6$
Z_2		$\mathbb{Z}_2^5$	$\mathbb{Z}_2^7$	$\mathbb{Z}_2^{10}$
$Z_2 \times Z_2^T$	$\mathbb{Z}_2^3$	$\mathbb{Z}_2^{10}$	$\mathbb{Z}_2^{22}$	$\mathbb{Z}_2^{10}$
Z_4		$\mathbb{Z}_2^5$	$\mathbb{Z}_2^7$	$\mathbb{Z}_2^8 \times \mathbb{Z}_4^2$
Z_6		$\mathbb{Z}_2^5$	$\mathbb{Z}_2^7$	$\mathbb{Z}_2^9 \times \mathbb{Z}_6$
$Z_2 \times Z_2$	$\mathbb{Z}_2^2$	$\mathbb{Z}_2^{13}$	$\mathbb{Z}_2^{17}$	$\mathbb{Z}_2^{14}$
$Z_4 \times Z_4$	$\mathbb{Z}_2^2$	$\mathbb{Z}_2^{13}$	$\mathbb{Z}_2^{16} \times \mathbb{Z}_4$	$\mathbb{Z}_2^{10} \times \mathbb{Z}_4^4$
$Z_6 \times Z_6$	$\mathbb{Z}_2^2$	$\mathbb{Z}_2^{13}$	$\mathbb{Z}_2^{17}$	$\mathbb{Z}_2^{12} \times \mathbb{Z}_6^2$
$U(1)$		$\mathbb{Z}_2^3$	$\mathbb{Z}_2^2$	$\mathbb{Z} \times \mathbb{Z}_2^8 \times \mathbb{Z}_4$
$SU(2)$		$\mathbb{Z}_2^2$	$\mathbb{Z}_2^2$	$\mathbb{Z}_2^6$
$SO(3)$		$\mathbb{Z}_2^3$	$\mathbb{Z}_2^7$	$\mathbb{Z}_2^6$
Space group #120 $I\bar{4}c2$				
$\varnothing$				$\mathbb{Z}_2^6$
Z_2^T	$\mathbb{Z}_2$		$\mathbb{Z}_2^9$	$\mathbb{Z}_2^4$
Z_2		$\mathbb{Z}_2^3$	$\mathbb{Z}_2^2$	$\mathbb{Z}_2^{10}$
$Z_2 \times Z_2^T$	$\mathbb{Z}_2^3$	$\mathbb{Z}_2^6$	$\mathbb{Z}_2^{16}$	$\mathbb{Z}_2^8$
Z_4		$\mathbb{Z}_2^3$	$\mathbb{Z}_2^2$	$\mathbb{Z}_2^8 \times \mathbb{Z}_4^2$
Z_6		$\mathbb{Z}_2^3$	$\mathbb{Z}_2^2$	$\mathbb{Z}_2^9 \times \mathbb{Z}_6$
$Z_2 \times Z_2$	$\mathbb{Z}_2^2$	$\mathbb{Z}_2^9$	$\mathbb{Z}_2^9$	$\mathbb{Z}_2^{14}$
$Z_4 \times Z_4$	$\mathbb{Z}_2^2$	$\mathbb{Z}_2^9$	$\mathbb{Z}_2^8 \times \mathbb{Z}_4$	$\mathbb{Z}_2^{10} \times \mathbb{Z}_4^4$
$Z_6 \times Z_6$	$\mathbb{Z}_2^2$	$\mathbb{Z}_2^9$	$\mathbb{Z}_2^9$	$\mathbb{Z}_2^{12} \times \mathbb{Z}_6^2$
$U(1)$		$\mathbb{Z}_2$		$\mathbb{Z} \times \mathbb{Z}_2^8 \times \mathbb{Z}_4$
$SU(2)$		$\mathbb{Z}_2$		$\mathbb{Z}_2^6$
$SO(3)$		$\mathbb{Z}_2$	$\mathbb{Z}_2^5$	$\mathbb{Z}_2^6$
Space group #121 $I\bar{4}2m$				
$\varnothing$		$\mathbb{Z}_2$	$\mathbb{Z}_2$	$\mathbb{Z}_2^6$
Z_2^T	$\mathbb{Z}_2$	$\mathbb{Z}_2^2$	$\mathbb{Z}_2^{11}$	$\mathbb{Z}_2^5$
Z_2		$\mathbb{Z}_2^5$	$\mathbb{Z}_2^5$	$\mathbb{Z}_2^{10}$
$Z_2 \times Z_2^T$	$\mathbb{Z}_2^3$	$\mathbb{Z}_2^{10}$	$\mathbb{Z}_2^{20}$	$\mathbb{Z}_2^9$
Z_4		$\mathbb{Z}_2^5$	$\mathbb{Z}_2^5$	$\mathbb{Z}_2^9 \times \mathbb{Z}_4$
Z_6		$\mathbb{Z}_2^5$	$\mathbb{Z}_2^5$	$\mathbb{Z}_2^9 \times \mathbb{Z}_6$
$Z_2 \times Z_2$	$\mathbb{Z}_2^2$	$\mathbb{Z}_2^{13}$	$\mathbb{Z}_2^{14}$	$\mathbb{Z}_2^{14}$

TABLE II (Continued.)

G_0	$E^3_{3,\infty}$	$E^2_{2,\infty}$	$E^1_{1,\infty}$	$E^0_{0,\infty}$
$Z_4 \times Z_4$	$\mathbb{Z}_2^2$	$\mathbb{Z}_2^{13}$	$\mathbb{Z}_2^{14}$	$\mathbb{Z}_2^{12} \times \mathbb{Z}_4^2$
$Z_6 \times Z_6$	$\mathbb{Z}_2^2$	$\mathbb{Z}_2^{13}$	$\mathbb{Z}_2^{14}$	$\mathbb{Z}_2^{12} \times \mathbb{Z}_6^2$
$U(1)$		$\mathbb{Z}_2^3$	$\mathbb{Z}_2$	$\mathbb{Z} \times \mathbb{Z}_2^9$
$SU(2)$		$\mathbb{Z}_2^2$	$\mathbb{Z}_2$	$\mathbb{Z}_2^6$
$SO(3)$		$\mathbb{Z}_2^3$	$\mathbb{Z}_2^6$	$\mathbb{Z}_2^6$
Space group #122 $I\bar{4}2d$				
$\varnothing$				$\mathbb{Z}_2^2 \times \mathbb{Z}_4$
Z_2^T	$\mathbb{Z}_2$		$\mathbb{Z}_2^5$	$\mathbb{Z}_2^2$
Z_2		$\mathbb{Z}_2^2$	$\mathbb{Z}_2$	$\mathbb{Z}_2^5 \times \mathbb{Z}_4$
$Z_2 \times Z_2^T$	$\mathbb{Z}_2^3$	$\mathbb{Z}_2^4$	$\mathbb{Z}_2^9$	$\mathbb{Z}_2^5$
Z_4		$\mathbb{Z}_2^2$	$\mathbb{Z}_2$	$\mathbb{Z}_2^4 \times \mathbb{Z}_4^2$
Z_6		$\mathbb{Z}_2^2$	$\mathbb{Z}_2$	$\mathbb{Z}_2^4 \times \mathbb{Z}_4 \times \mathbb{Z}_6$
$Z_2 \times Z_2$	$\mathbb{Z}_2^2$	$\mathbb{Z}_2^6$	$\mathbb{Z}_2^5$	$\mathbb{Z}_2^8 \times \mathbb{Z}_4$
$Z_4 \times Z_4$	$\mathbb{Z}_2^2$	$\mathbb{Z}_2^6$	$\mathbb{Z}_2^5$	$\mathbb{Z}_2^6 \times \mathbb{Z}_4^3$
$Z_6 \times Z_6$	$\mathbb{Z}_2^2$	$\mathbb{Z}_2^6$	$\mathbb{Z}_2^5$	$\mathbb{Z}_2^6 \times \mathbb{Z}_4 \times \mathbb{Z}_6^2$
$U(1)$		$\mathbb{Z}_2$		$\mathbb{Z} \times \mathbb{Z}_2^4 \times \mathbb{Z}_4$
$SU(2)$		$\mathbb{Z}_2$		$\mathbb{Z}_2^2 \times \mathbb{Z}_4$
$SO(3)$		$\mathbb{Z}_2$	$\mathbb{Z}_2^3$	$\mathbb{Z}_2^2 \times \mathbb{Z}_4$
Space group #123 $P4/mmm$				
$\varnothing$		$\mathbb{Z}_2^5$	$\mathbb{Z}_2^9$	$\mathbb{Z}_2^{18}$
Z_2^T	$\mathbb{Z}_2$	$\mathbb{Z}_2^{10}$	$\mathbb{Z}_2^{36}$	$\mathbb{Z}_2^{18}$
Z_2		$\mathbb{Z}_2^{15}$	$\mathbb{Z}_2^{27}$	$\mathbb{Z}_2^{24}$
$Z_2 \times Z_2^T$	$\mathbb{Z}_2^3$	$\mathbb{Z}_2^{30}$	$\mathbb{Z}_2^{63}$	$\mathbb{Z}_2^{24}$
Z_4		$\mathbb{Z}_2^{15}$	$\mathbb{Z}_2^{27}$	$\mathbb{Z}_2^{22} \times \mathbb{Z}_4^2$
Z_6		$\mathbb{Z}_2^{15}$	$\mathbb{Z}_2^{27}$	$\mathbb{Z}_2^{23} \times \mathbb{Z}_6$
$Z_2 \times Z_2$	$\mathbb{Z}_2^2$	$\mathbb{Z}_2^{35}$	$\mathbb{Z}_2^{54}$	$\mathbb{Z}_2^{30}$
$Z_4 \times Z_4$	$\mathbb{Z}_2^2$	$\mathbb{Z}_2^{35}$	$\mathbb{Z}_2^{53} \times \mathbb{Z}_4$	$\mathbb{Z}_2^{26} \times \mathbb{Z}_4^4$
$Z_6 \times Z_6$	$\mathbb{Z}_2^2$	$\mathbb{Z}_2^{35}$	$\mathbb{Z}_2^{54}$	$\mathbb{Z}_2^{28} \times \mathbb{Z}_6^2$
$U(1)$		$\mathbb{Z}_2^{11}$	$\mathbb{Z}_2^9$	$\mathbb{Z} \times \mathbb{Z}_2^{22} \times \mathbb{Z}_4$
$SU(2)$		$\mathbb{Z}_2^6$	$\mathbb{Z}_2^9$	$\mathbb{Z}_2^{18}$
$SO(3)$		$\mathbb{Z}_2^{11}$	$\mathbb{Z}_2^{18}$	$\mathbb{Z}_2^{18}$
Space group #124 $P4/mcc$				
$\varnothing$		$\mathbb{Z}_2$		$\mathbb{Z}_2^{12}$
Z_2^T	$\mathbb{Z}_2$	$\mathbb{Z}_2^2$	$\mathbb{Z}_2^{16}$	$\mathbb{Z}_2^{12}$
Z_2		$\mathbb{Z}_2^6$	$\mathbb{Z}_2^8$	$\mathbb{Z}_2^{18}$
$Z_2 \times Z_2^T$	$\mathbb{Z}_2^3$	$\mathbb{Z}_2^{12}$	$\mathbb{Z}_2^{32}$	$\mathbb{Z}_2^{18}$
Z_4		$\mathbb{Z}_2^6$	$\mathbb{Z}_2^8$	$\mathbb{Z}_2^{16} \times \mathbb{Z}_4^2$
Z_6		$\mathbb{Z}_2^6$	$\mathbb{Z}_2^8$	$\mathbb{Z}_2^{17} \times \mathbb{Z}_6$
$Z_2 \times Z_2$	$\mathbb{Z}_2^2$	$\mathbb{Z}_2^{16}$	$\mathbb{Z}_2^{24}$	$\mathbb{Z}_2^{24}$
$Z_4 \times Z_4$	$\mathbb{Z}_2^2$	$\mathbb{Z}_2^{16}$	$\mathbb{Z}_2^{23} \times \mathbb{Z}_4$	$\mathbb{Z}_2^{20} \times \mathbb{Z}_4^4$
$Z_6 \times Z_6$	$\mathbb{Z}_2^2$	$\mathbb{Z}_2^{16}$	$\mathbb{Z}_2^{24}$	$\mathbb{Z}_2^{22} \times \mathbb{Z}_6^2$
$U(1)$		$\mathbb{Z}_2^3$		$\mathbb{Z} \times \mathbb{Z}_2^{16} \times \mathbb{Z}_4$
$SU(2)$		$\mathbb{Z}_2^2$		$\mathbb{Z}_2^{12}$
$SO(3)$		$\mathbb{Z}_2^3$	$\mathbb{Z}_2^8$	$\mathbb{Z}_2^{12}$
Space group #125 $P4/nbm$				
$\varnothing$		$\mathbb{Z}_2$	$\mathbb{Z}_2$	$\mathbb{Z}_2^{11}$
Z_2^T	$\mathbb{Z}_2$	$\mathbb{Z}_2^2$	$\mathbb{Z}_2^{17}$	$\mathbb{Z}_2^{11}$
Z_2		$\mathbb{Z}_2^6$	$\mathbb{Z}_2^9$	$\mathbb{Z}_2^{17}$
$Z_2 \times Z_2^T$	$\mathbb{Z}_2^3$	$\mathbb{Z}_2^{12}$	$\mathbb{Z}_2^{33}$	$\mathbb{Z}_2^{17}$

TABLE II (Continued.)

G_0	$E^3_{3,\infty}$	$E^2_{2,\infty}$	$E^1_{1,\infty}$	$E^0_{0,\infty}$
Z_4		$\mathbb{Z}_2^6$	$\mathbb{Z}_2^8 \times \mathbb{Z}_4$	$\mathbb{Z}_2^{15} \times \mathbb{Z}_4^2$
Z_6		$\mathbb{Z}_2^6$	$\mathbb{Z}_2^9$	$\mathbb{Z}_2^{16} \times \mathbb{Z}_6$
$Z_2 \times Z_2$	$\mathbb{Z}_2^2$	$\mathbb{Z}_2^{16}$	$\mathbb{Z}_2^{25}$	$\mathbb{Z}_2^{23}$
$Z_4 \times Z_4$	$\mathbb{Z}_2^2$	$\mathbb{Z}_2^{16}$	$\mathbb{Z}_2^{22} \times \mathbb{Z}_4^3$	$\mathbb{Z}_2^{19} \times \mathbb{Z}_4^4$
$Z_6 \times Z_6$	$\mathbb{Z}_2^2$	$\mathbb{Z}_2^{16}$	$\mathbb{Z}_2^{25}$	$\mathbb{Z}_2^{21} \times \mathbb{Z}_6^2$
$U(1)$		$\mathbb{Z}_2^3$	$\mathbb{Z}_2$	$\mathbb{Z} \times \mathbb{Z}_2^{15} \times \mathbb{Z}_4$
$SU(2)$		$\mathbb{Z}_2^2$	$\mathbb{Z}_2$	$\mathbb{Z}_2^{11}$
$SO(3)$		$\mathbb{Z}_2^3$	$\mathbb{Z}_2^9$	$\mathbb{Z}_2^{11}$
Space group #126 $P4/nnc$				
$\varnothing$				$\mathbb{Z}_2^8$
Z_2^T	$\mathbb{Z}_2$		$\mathbb{Z}_2^{11}$	$\mathbb{Z}_2^7$
Z_2		$\mathbb{Z}_2^3$	$\mathbb{Z}_2^4$	$\mathbb{Z}_2^{13}$
$Z_2 \times Z_2^T$	$\mathbb{Z}_2^3$	$\mathbb{Z}_2^6$	$\mathbb{Z}_2^{21}$	$\mathbb{Z}_2^{12}$
Z_4		$\mathbb{Z}_2^3$	$\mathbb{Z}_2^3 \times \mathbb{Z}_4$	$\mathbb{Z}_2^{12} \times \mathbb{Z}_4$
Z_6		$\mathbb{Z}_2^3$	$\mathbb{Z}_2^4$	$\mathbb{Z}_2^{12} \times \mathbb{Z}_6$
$Z_2 \times Z_2$	$\mathbb{Z}_2^2$	$\mathbb{Z}_2^9$	$\mathbb{Z}_2^{14}$	$\mathbb{Z}_2^{18}$
$Z_4 \times Z_4$	$\mathbb{Z}_2^2$	$\mathbb{Z}_2^9$	$\mathbb{Z}_2^{12} \times \mathbb{Z}_4^2$	$\mathbb{Z}_2^{16} \times \mathbb{Z}_4^2$
$Z_6 \times Z_6$	$\mathbb{Z}_2^2$	$\mathbb{Z}_2^9$	$\mathbb{Z}_2^{14}$	$\mathbb{Z}_2^{16} \times \mathbb{Z}_6^2$
$U(1)$		$\mathbb{Z}_2$		$\mathbb{Z} \times \mathbb{Z}_2^{12}$
$SU(2)$		$\mathbb{Z}_2$		$\mathbb{Z}_2^8$
$SO(3)$		$\mathbb{Z}_2$	$\mathbb{Z}_2^6$	$\mathbb{Z}_2^8$
Space group #127 $P4/mbm$				
$\varnothing$		$\mathbb{Z}_2^3$	$\mathbb{Z}_2^3$	$\mathbb{Z}_2^9 \times \mathbb{Z}_4$
Z_2^T	$\mathbb{Z}_2$	$\mathbb{Z}_2^6$	$\mathbb{Z}_2^{18}$	$\mathbb{Z}_2^{10}$
Z_2		$\mathbb{Z}_2^{10}$	$\mathbb{Z}_2^{12}$	$\mathbb{Z}_2^{13} \times \mathbb{Z}_4$
$Z_2 \times Z_2^T$	$\mathbb{Z}_2^3$	$\mathbb{Z}_2^{20}$	$\mathbb{Z}_2^{33}$	$\mathbb{Z}_2^{14}$
Z_4		$\mathbb{Z}_2^{10}$	$\mathbb{Z}_2^{12}$	$\mathbb{Z}_2^{11} \times \mathbb{Z}_4^3$
Z_6		$\mathbb{Z}_2^{10}$	$\mathbb{Z}_2^{12}$	$\mathbb{Z}_2^{12} \times \mathbb{Z}_4 \times \mathbb{Z}_6$
$Z_2 \times Z_2$	$\mathbb{Z}_2^2$	$\mathbb{Z}_2^{24}$	$\mathbb{Z}_2^{27}$	$\mathbb{Z}_2^{17} \times \mathbb{Z}_4$
$Z_4 \times Z_4$	$\mathbb{Z}_2^2$	$\mathbb{Z}_2^{24}$	$\mathbb{Z}_2^{26} \times \mathbb{Z}_4$	$\mathbb{Z}_2^{13} \times \mathbb{Z}_4^5$
$Z_6 \times Z_6$	$\mathbb{Z}_2^2$	$\mathbb{Z}_2^{24}$	$\mathbb{Z}_2^{27}$	$\mathbb{Z}_2^{15} \times \mathbb{Z}_4 \times \mathbb{Z}_6^2$
$U(1)$		$\mathbb{Z}_2^7$	$\mathbb{Z}_2^3$	$\mathbb{Z} \times \mathbb{Z}_2^{11} \times \mathbb{Z}_4^2$
$SU(2)$		$\mathbb{Z}_2^4$	$\mathbb{Z}_2^3$	$\mathbb{Z}_2^9 \times \mathbb{Z}_4$
$SO(3)$		$\mathbb{Z}_2^7$	$\mathbb{Z}_2^9$	$\mathbb{Z}_2^9 \times \mathbb{Z}_4$
Space group #128 $P4/mnc$				
$\varnothing$		$\mathbb{Z}_2$		$\mathbb{Z}_2^7 \times \mathbb{Z}_4$
Z_2^T	$\mathbb{Z}_2$	$\mathbb{Z}_2^2$	$\mathbb{Z}_2^{10}$	$\mathbb{Z}_2^8$
Z_2		$\mathbb{Z}_2^5$	$\mathbb{Z}_2^5$	$\mathbb{Z}_2^{11} \times \mathbb{Z}_4$
$Z_2 \times Z_2^T$	$\mathbb{Z}_2^3$	$\mathbb{Z}_2^{10}$	$\mathbb{Z}_2^{20}$	$\mathbb{Z}_2^{12}$
Z_4		$\mathbb{Z}_2^5$	$\mathbb{Z}_2^5$	$\mathbb{Z}_2^{10} \times \mathbb{Z}_4^2$
Z_6		$\mathbb{Z}_2^5$	$\mathbb{Z}_2^5$	$\mathbb{Z}_2^{10} \times \mathbb{Z}_4 \times \mathbb{Z}_6$
$Z_2 \times Z_2$	$\mathbb{Z}_2^2$	$\mathbb{Z}_2^{13}$	$\mathbb{Z}_2^{15}$	$\mathbb{Z}_2^{15} \times \mathbb{Z}_4$
$Z_4 \times Z_4$	$\mathbb{Z}_2^2$	$\mathbb{Z}_2^{13}$	$\mathbb{Z}_2^{15}$	$\mathbb{Z}_2^{13} \times \mathbb{Z}_4^3$
$Z_6 \times Z_6$	$\mathbb{Z}_2^2$	$\mathbb{Z}_2^{13}$	$\mathbb{Z}_2^{15}$	$\mathbb{Z}_2^{13} \times \mathbb{Z}_4 \times \mathbb{Z}_6^2$
$U(1)$		$\mathbb{Z}_2^3$		$\mathbb{Z} \times \mathbb{Z}_2^{10} \times \mathbb{Z}_4$
$SU(2)$		$\mathbb{Z}_2^2$		$\mathbb{Z}_2^7 \times \mathbb{Z}_4$
$SO(3)$		$\mathbb{Z}_2^3$	$\mathbb{Z}_2^5$	$\mathbb{Z}_2^7 \times \mathbb{Z}_4$
Space group #129 $P4/nmm$				
$\varnothing$		$\mathbb{Z}_2^2$	$\mathbb{Z}_2^2$	$\mathbb{Z}_2^9$

TABLE II (Continued.)

G_0	$E^3_{3,\infty}$	$E^2_{2,\infty}$	$E^1_{1,\infty}$	$E^0_{0,\infty}$
Z_2^T	$\mathbb{Z}_2$	$\mathbb{Z}_2^4$	$\mathbb{Z}_2^{17}$	$\mathbb{Z}_2^9$
Z_2		$\mathbb{Z}_2^8$	$\mathbb{Z}_2^{10}$	$\mathbb{Z}_2^{14}$
$Z_2\times Z_2^T$	$\mathbb{Z}_2^3$	$\mathbb{Z}_2^{16}$	$\mathbb{Z}_2^{32}$	$\mathbb{Z}_2^{14}$
Z_4		$\mathbb{Z}_2^7\times\mathbb{Z}_4$	$\mathbb{Z}_2^{10}$	$\mathbb{Z}_2^{12}\times\mathbb{Z}_4^2$
Z_6		$\mathbb{Z}_2^8$	$\mathbb{Z}_2^{10}$	$\mathbb{Z}_2^{13}\times\mathbb{Z}_6$
$Z_2\times Z_2$	$\mathbb{Z}_2^2$	$\mathbb{Z}_2^{20}$	$\mathbb{Z}_2^{25}$	$\mathbb{Z}_2^{19}$
$Z_4\times Z_4$	$\mathbb{Z}_2^2$	$\mathbb{Z}_2^{17}\times\mathbb{Z}_4^3$	$\mathbb{Z}_2^{23}\times\mathbb{Z}_4^2$	$\mathbb{Z}_2^{15}\times\mathbb{Z}_4^4$
$Z_6\times Z_6$	$\mathbb{Z}_2^2$	$\mathbb{Z}_2^{20}$	$\mathbb{Z}_2^{25}$	$\mathbb{Z}_2^{17}\times\mathbb{Z}_6^2$
$U(1)$		$\mathbb{Z}_2^5$	$\mathbb{Z}_2^2$	$\mathbb{Z}\times\mathbb{Z}_2^{12}\times\mathbb{Z}_4$
$SU(2)$		$\mathbb{Z}_2^3$	$\mathbb{Z}_2^2$	$\mathbb{Z}_2^9$
$SO(3)$		$\mathbb{Z}_2^5$	$\mathbb{Z}_2^9$	$\mathbb{Z}_2^9$
Space group #130 $P4/ncc$				
$\varnothing$				$\mathbb{Z}_2^5$
Z_2^T	$\mathbb{Z}_2$		$\mathbb{Z}_2^8$	$\mathbb{Z}_2^4$
Z_2		$\mathbb{Z}_2^3$	$\mathbb{Z}_2^2$	$\mathbb{Z}_2^9$
$Z_2\times Z_2^T$	$\mathbb{Z}_2^3$	$\mathbb{Z}_2^6$	$\mathbb{Z}_2^{15}$	$\mathbb{Z}_2^8$
Z_4		$\mathbb{Z}_2^2\times\mathbb{Z}_4$	$\mathbb{Z}_2^2$	$\mathbb{Z}_2^7\times\mathbb{Z}_4^2$
Z_6		$\mathbb{Z}_2^3$	$\mathbb{Z}_2^2$	$\mathbb{Z}_2^8\times\mathbb{Z}_6$
$Z_2\times Z_2$	$\mathbb{Z}_2^2$	$\mathbb{Z}_2^9$	$\mathbb{Z}_2^9$	$\mathbb{Z}_2^{13}$
$Z_4\times Z_4$	$\mathbb{Z}_2^2$	$\mathbb{Z}_2^6\times\mathbb{Z}_4^3$	$\mathbb{Z}_2^7\times\mathbb{Z}_4^2$	$\mathbb{Z}_2^9\times\mathbb{Z}_4^4$
$Z_6\times Z_6$	$\mathbb{Z}_2^2$	$\mathbb{Z}_2^9$	$\mathbb{Z}_2^9$	$\mathbb{Z}_2^{11}\times\mathbb{Z}_6^2$
$U(1)$		$\mathbb{Z}_2$		$\mathbb{Z}\times\mathbb{Z}_2^7\times\mathbb{Z}_4$
$SU(2)$		$\mathbb{Z}_2$		$\mathbb{Z}_2^5$
$SO(3)$		$\mathbb{Z}_2$	$\mathbb{Z}_2^5$	$\mathbb{Z}_2^5$
Space group #131 $P4_2/mmc$				
$\varnothing$		$\mathbb{Z}_2^3$	$\mathbb{Z}_2^7$	$\mathbb{Z}_2^{14}$
Z_2^T	$\mathbb{Z}_2$	$\mathbb{Z}_2^6$	$\mathbb{Z}_2^{28}$	$\mathbb{Z}_2^{14}$
Z_2		$\mathbb{Z}_2^{10}$	$\mathbb{Z}_2^{20}$	$\mathbb{Z}_2^{20}$
$Z_2\times Z_2^T$	$\mathbb{Z}_2^3$	$\mathbb{Z}_2^{20}$	$\mathbb{Z}_2^{49}$	$\mathbb{Z}_2^{20}$
Z_4		$\mathbb{Z}_2^{10}$	$\mathbb{Z}_2^{20}$	$\mathbb{Z}_2^{19}\times\mathbb{Z}_4$
Z_6		$\mathbb{Z}_2^{10}$	$\mathbb{Z}_2^{20}$	$\mathbb{Z}_2^{19}\times\mathbb{Z}_6$
$Z_2\times Z_2$	$\mathbb{Z}_2^2$	$\mathbb{Z}_2^{24}$	$\mathbb{Z}_2^{41}$	$\mathbb{Z}_2^{26}$
$Z_4\times Z_4$	$\mathbb{Z}_2^2$	$\mathbb{Z}_2^{24}$	$\mathbb{Z}_2^{41}$	$\mathbb{Z}_2^{24}\times\mathbb{Z}_4^2$
$Z_6\times Z_6$	$\mathbb{Z}_2^2$	$\mathbb{Z}_2^{24}$	$\mathbb{Z}_2^{41}$	$\mathbb{Z}_2^{24}\times\mathbb{Z}_6^2$
$U(1)$		$\mathbb{Z}_2^7$	$\mathbb{Z}_2^7$	$\mathbb{Z}\times\mathbb{Z}_2^{19}$
$SU(2)$		$\mathbb{Z}_2^4$	$\mathbb{Z}_2^7$	$\mathbb{Z}_2^{14}$
$SO(3)$		$\mathbb{Z}_2^7$	$\mathbb{Z}_2^{15}$	$\mathbb{Z}_2^{14}$
Space group #132 $P4_2/mcm$				
$\varnothing$		$\mathbb{Z}_2^2$	$\mathbb{Z}_2^4$	$\mathbb{Z}_2^{12}$
Z_2^T	$\mathbb{Z}_2$	$\mathbb{Z}_2^4$	$\mathbb{Z}_2^{22}$	$\mathbb{Z}_2^{12}$
Z_2		$\mathbb{Z}_2^8$	$\mathbb{Z}_2^{14}$	$\mathbb{Z}_2^{18}$
$Z_2\times Z_2^T$	$\mathbb{Z}_2^3$	$\mathbb{Z}_2^{16}$	$\mathbb{Z}_2^{40}$	$\mathbb{Z}_2^{18}$
Z_4		$\mathbb{Z}_2^8$	$\mathbb{Z}_2^{14}$	$\mathbb{Z}_2^{17}\times\mathbb{Z}_4$
Z_6		$\mathbb{Z}_2^8$	$\mathbb{Z}_2^{14}$	$\mathbb{Z}_2^{17}\times\mathbb{Z}_6$
$Z_2\times Z_2$	$\mathbb{Z}_2^2$	$\mathbb{Z}_2^{20}$	$\mathbb{Z}_2^{32}$	$\mathbb{Z}_2^{24}$
$Z_4\times Z_4$	$\mathbb{Z}_2^2$	$\mathbb{Z}_2^{20}$	$\mathbb{Z}_2^{32}$	$\mathbb{Z}_2^{22}\times\mathbb{Z}_4^2$
$Z_6\times Z_6$	$\mathbb{Z}_2^2$	$\mathbb{Z}_2^{20}$	$\mathbb{Z}_2^{32}$	$\mathbb{Z}_2^{22}\times\mathbb{Z}_6^2$
$U(1)$		$\mathbb{Z}_2^5$	$\mathbb{Z}_2^4$	$\mathbb{Z}\times\mathbb{Z}_2^{17}$
$SU(2)$		$\mathbb{Z}_2^3$	$\mathbb{Z}_2^4$	$\mathbb{Z}_2^{12}$

TABLE II (Continued.)

G_0	$E^3_{3,\infty}$	$E^2_{2,\infty}$	$E^1_{1,\infty}$	$E^0_{0,\infty}$
$SO(3)$		$\mathbb{Z}_2^5$	$\mathbb{Z}_2^{12}$	$\mathbb{Z}_2^{12}$
Space group #133 $P4_2/nbc$				
$\varnothing$				$\mathbb{Z}_2^8$
Z_2^T	$\mathbb{Z}_2$		$\mathbb{Z}_2^{11}$	$\mathbb{Z}_2^7$
Z_2		$\mathbb{Z}_2^3$	$\mathbb{Z}_2^4$	$\mathbb{Z}_2^{13}$
$Z_2\times Z_2^T$	$\mathbb{Z}_2^3$	$\mathbb{Z}_2^6$	$\mathbb{Z}_2^{21}$	$\mathbb{Z}_2^{12}$
Z_4		$\mathbb{Z}_2^3$	$\mathbb{Z}_2^4$	$\mathbb{Z}_2^{12}\times\mathbb{Z}_4$
Z_6		$\mathbb{Z}_2^3$	$\mathbb{Z}_2^4$	$\mathbb{Z}_2^{12}\times\mathbb{Z}_6$
$Z_2\times Z_2$	$\mathbb{Z}_2^2$	$\mathbb{Z}_2^9$	$\mathbb{Z}_2^{14}$	$\mathbb{Z}_2^{18}$
$Z_4\times Z_4$	$\mathbb{Z}_2^2$	$\mathbb{Z}_2^9$	$\mathbb{Z}_2^{14}$	$\mathbb{Z}_2^{16}\times\mathbb{Z}_4^2$
$Z_6\times Z_6$	$\mathbb{Z}_2^2$	$\mathbb{Z}_2^9$	$\mathbb{Z}_2^{14}$	$\mathbb{Z}_2^{16}\times\mathbb{Z}_6^2$
$U(1)$		$\mathbb{Z}_2$		$\mathbb{Z}\times\mathbb{Z}_2^{12}$
$SU(2)$		$\mathbb{Z}_2$		$\mathbb{Z}_2^8$
$SO(3)$		$\mathbb{Z}_2$	$\mathbb{Z}_2^6$	$\mathbb{Z}_2^8$
Space group #134 $P4_2/nnm$				
$\varnothing$		$\mathbb{Z}_2$	$\mathbb{Z}_2$	$\mathbb{Z}_2^{11}$
Z_2^T	$\mathbb{Z}_2$	$\mathbb{Z}_2^2$	$\mathbb{Z}_2^{17}$	$\mathbb{Z}_2^{11}$
Z_2		$\mathbb{Z}_2^6$	$\mathbb{Z}_2^9$	$\mathbb{Z}_2^{17}$
$Z_2\times Z_2^T$	$\mathbb{Z}_2^3$	$\mathbb{Z}_2^{12}$	$\mathbb{Z}_2^{33}$	$\mathbb{Z}_2^{17}$
Z_4		$\mathbb{Z}_2^6$	$\mathbb{Z}_2^9$	$\mathbb{Z}_2^{16}\times\mathbb{Z}_4$
Z_6		$\mathbb{Z}_2^6$	$\mathbb{Z}_2^9$	$\mathbb{Z}_2^{16}\times\mathbb{Z}_6$
$Z_2\times Z_2$	$\mathbb{Z}_2^2$	$\mathbb{Z}_2^{16}$	$\mathbb{Z}_2^{25}$	$\mathbb{Z}_2^{23}$
$Z_4\times Z_4$	$\mathbb{Z}_2^2$	$\mathbb{Z}_2^{16}$	$\mathbb{Z}_2^{25}$	$\mathbb{Z}_2^{21}\times\mathbb{Z}_4^2$
$Z_6\times Z_6$	$\mathbb{Z}_2^2$	$\mathbb{Z}_2^{16}$	$\mathbb{Z}_2^{25}$	$\mathbb{Z}_2^{21}\times\mathbb{Z}_6^2$
$U(1)$		$\mathbb{Z}_2^3$	$\mathbb{Z}_2$	$\mathbb{Z}\times\mathbb{Z}_2^{16}$
$SU(2)$		$\mathbb{Z}_2^2$	$\mathbb{Z}_2$	$\mathbb{Z}_2^{11}$
$SO(3)$		$\mathbb{Z}_2^3$	$\mathbb{Z}_2^9$	$\mathbb{Z}_2^{11}$
Space group #135 $P4_2/mbc$				
$\varnothing$		$\mathbb{Z}_2$		$\mathbb{Z}_2^7$
Z_2^T	$\mathbb{Z}_2$	$\mathbb{Z}_2^2$	$\mathbb{Z}_2^{10}$	$\mathbb{Z}_2^6$
Z_2		$\mathbb{Z}_2^5$	$\mathbb{Z}_2^4$	$\mathbb{Z}_2^{11}$
$Z_2\times Z_2^T$	$\mathbb{Z}_2^3$	$\mathbb{Z}_2^{10}$	$\mathbb{Z}_2^{19}$	$\mathbb{Z}_2^{10}$
Z_4		$\mathbb{Z}_2^5$	$\mathbb{Z}_2^4$	$\mathbb{Z}_2^{10}\times\mathbb{Z}_4$
Z_6		$\mathbb{Z}_2^5$	$\mathbb{Z}_2^4$	$\mathbb{Z}_2^{10}\times\mathbb{Z}_6$
$Z_2\times Z_2$	$\mathbb{Z}_2^2$	$\mathbb{Z}_2^{13}$	$\mathbb{Z}_2^{13}$	$\mathbb{Z}_2^{15}$
$Z_4\times Z_4$	$\mathbb{Z}_2^2$	$\mathbb{Z}_2^{13}$	$\mathbb{Z}_2^{13}$	$\mathbb{Z}_2^{13}\times\mathbb{Z}_4^2$
$Z_6\times Z_6$	$\mathbb{Z}_2^2$	$\mathbb{Z}_2^{13}$	$\mathbb{Z}_2^{13}$	$\mathbb{Z}_2^{13}\times\mathbb{Z}_6^2$
$U(1)$		$\mathbb{Z}_2^3$		$\mathbb{Z}\times\mathbb{Z}_2^{10}$
$SU(2)$		$\mathbb{Z}_2^2$		$\mathbb{Z}_2^7$
$SO(3)$		$\mathbb{Z}_2^3$	$\mathbb{Z}_2^5$	$\mathbb{Z}_2^7$
Space group #136 $P4_2/mnm$				
$\varnothing$		$\mathbb{Z}_2^2$	$\mathbb{Z}_2^3$	$\mathbb{Z}_2^9$
Z_2^T	$\mathbb{Z}_2$	$\mathbb{Z}_2^4$	$\mathbb{Z}_2^{16}$	$\mathbb{Z}_2^8$
Z_2		$\mathbb{Z}_2^7$	$\mathbb{Z}_2^{10}$	$\mathbb{Z}_2^{13}$
$Z_2\times Z_2^T$	$\mathbb{Z}_2^3$	$\mathbb{Z}_2^{14}$	$\mathbb{Z}_2^{28}$	$\mathbb{Z}_2^{12}$
Z_4		$\mathbb{Z}_2^6\times\mathbb{Z}_4$	$\mathbb{Z}_2^{10}$	$\mathbb{Z}_2^{12}\times\mathbb{Z}_4$
Z_6		$\mathbb{Z}_2^7$	$\mathbb{Z}_2^{10}$	$\mathbb{Z}_2^{12}\times\mathbb{Z}_6$
$Z_2\times Z_2$	$\mathbb{Z}_2^2$	$\mathbb{Z}_2^{17}$	$\mathbb{Z}_2^{22}$	$\mathbb{Z}_2^{17}$
$Z_4\times Z_4$	$\mathbb{Z}_2^2$	$\mathbb{Z}_2^{14}\times\mathbb{Z}_4^3$	$\mathbb{Z}_2^{21}\times\mathbb{Z}_4$	$\mathbb{Z}_2^{15}\times\mathbb{Z}_4^2$

TABLE II (Continued.)

G_0	$E_{3,\infty}^3$	$E_{2,\infty}^2$	$E_{1,\infty}^1$	$E_{0,\infty}^0$
$Z_6 \times Z_6$	$\mathbb{Z}_2^2$	$\mathbb{Z}_2^{17}$	$\mathbb{Z}_2^{22}$	$\mathbb{Z}_2^{15} \times \mathbb{Z}_6^2$
$U(1)$		$\mathbb{Z}_2^5$	$\mathbb{Z}_2^3$	$\mathbb{Z} \times \mathbb{Z}_2^{12}$
$SU(2)$		$\mathbb{Z}_2^3$	$\mathbb{Z}_2^3$	$\mathbb{Z}_2^9$
$SO(3)$		$\mathbb{Z}_2^5$	$\mathbb{Z}_2^8$	$\mathbb{Z}_2^9$

Space group #137 $P4_2/nmc$

G_0	$E_{3,\infty}^3$	$E_{2,\infty}^2$	$E_{1,\infty}^1$	$E_{0,\infty}^0$
$\varnothing$		$\mathbb{Z}_2$	$\mathbb{Z}_2^2$	$\mathbb{Z}_2^5$
Z_2^T	$\mathbb{Z}_2$	$\mathbb{Z}_2^2$	$\mathbb{Z}_2^{11}$	$\mathbb{Z}_2^5$
Z_2		$\mathbb{Z}_2^5$	$\mathbb{Z}_2^6$	$\mathbb{Z}_2^9$
$Z_2 \times Z_2^T$	$\mathbb{Z}_2^3$	$\mathbb{Z}_2^{10}$	$\mathbb{Z}_2^{20}$	$\mathbb{Z}_2^9$
Z_4		$\mathbb{Z}_2^4 \times \mathbb{Z}_4$	$\mathbb{Z}_2^6$	$\mathbb{Z}_2^8 \times \mathbb{Z}_4$
Z_6		$\mathbb{Z}_2^5$	$\mathbb{Z}_2^6$	$\mathbb{Z}_2^8 \times \mathbb{Z}_6$
$Z_2 \times Z_2$	$\mathbb{Z}_2^2$	$\mathbb{Z}_2^{13}$	$\mathbb{Z}_2^{15}$	$\mathbb{Z}_2^{13}$
$Z_4 \times Z_4$	$\mathbb{Z}_2^2$	$\mathbb{Z}_2^{10} \times \mathbb{Z}_4^3$	$\mathbb{Z}_2^{14} \times \mathbb{Z}_4$	$\mathbb{Z}_2^{11} \times \mathbb{Z}_4^2$
$Z_6 \times Z_6$	$\mathbb{Z}_2^2$	$\mathbb{Z}_2^{13}$	$\mathbb{Z}_2^{15}$	$\mathbb{Z}_2^{11} \times \mathbb{Z}_6^2$
$U(1)$		$\mathbb{Z}_2^3$	$\mathbb{Z}_2^2$	$\mathbb{Z} \times \mathbb{Z}_2^8$
$SU(2)$		$\mathbb{Z}_2^2$	$\mathbb{Z}_2^2$	$\mathbb{Z}_2^5$
$SO(3)$		$\mathbb{Z}_2^3$	$\mathbb{Z}_2^7$	$\mathbb{Z}_2^5$

Space group #138 $P4_2/ncm$

G_0	$E_{3,\infty}^3$	$E_{2,\infty}^2$	$E_{1,\infty}^1$	$E_{0,\infty}^0$
$\varnothing$		$\mathbb{Z}_2$	$\mathbb{Z}_2$	$\mathbb{Z}_2^8$
Z_2^T	$\mathbb{Z}_2$	$\mathbb{Z}_2^2$	$\mathbb{Z}_2^{13}$	$\mathbb{Z}_2^7$
Z_2		$\mathbb{Z}_2^5$	$\mathbb{Z}_2^6$	$\mathbb{Z}_2^{13}$
$Z_2 \times Z_2^T$	$\mathbb{Z}_2^3$	$\mathbb{Z}_2^{10}$	$\mathbb{Z}_2^{24}$	$\mathbb{Z}_2^{12}$
Z_4		$\mathbb{Z}_2^4 \times \mathbb{Z}_4$	$\mathbb{Z}_2^6$	$\mathbb{Z}_2^{12} \times \mathbb{Z}_4$
Z_6		$\mathbb{Z}_2^5$	$\mathbb{Z}_2^6$	$\mathbb{Z}_2^{12} \times \mathbb{Z}_6$
$Z_2 \times Z_2$	$\mathbb{Z}_2^2$	$\mathbb{Z}_2^{13}$	$\mathbb{Z}_2^{17}$	$\mathbb{Z}_2^{18}$
$Z_4 \times Z_4$	$\mathbb{Z}_2^2$	$\mathbb{Z}_2^{10} \times \mathbb{Z}_4^3$	$\mathbb{Z}_2^{16} \times \mathbb{Z}_4$	$\mathbb{Z}_2^{16} \times \mathbb{Z}_4^2$
$Z_6 \times Z_6$	$\mathbb{Z}_2^2$	$\mathbb{Z}_2^{13}$	$\mathbb{Z}_2^{17}$	$\mathbb{Z}_2^{16} \times \mathbb{Z}_6^2$
$U(1)$		$\mathbb{Z}_2^3$	$\mathbb{Z}_2$	$\mathbb{Z} \times \mathbb{Z}_2^{12}$
$SU(2)$		$\mathbb{Z}_2^2$	$\mathbb{Z}_2$	$\mathbb{Z}_2^8$
$SO(3)$		$\mathbb{Z}_2^3$	$\mathbb{Z}_2^7$	$\mathbb{Z}_2^8$

Space group #139 $I4/mmm$

G_0	$E_{3,\infty}^3$	$E_{2,\infty}^2$	$E_{1,\infty}^1$	$E_{0,\infty}^0$
$\varnothing$		$\mathbb{Z}_2^3$	$\mathbb{Z}_2^5$	$\mathbb{Z}_2^{12}$
Z_2^T	$\mathbb{Z}_2$	$\mathbb{Z}_2^6$	$\mathbb{Z}_2^{23}$	$\mathbb{Z}_2^{12}$
Z_2		$\mathbb{Z}_2^{10}$	$\mathbb{Z}_2^{16}$	$\mathbb{Z}_2^{17}$
$Z_2 \times Z_2^T$	$\mathbb{Z}_2^3$	$\mathbb{Z}_2^{20}$	$\mathbb{Z}_2^{41}$	$\mathbb{Z}_2^{17}$
Z_4		$\mathbb{Z}_2^{10}$	$\mathbb{Z}_2^{16}$	$\mathbb{Z}_2^{16} \times \mathbb{Z}_4$
Z_6		$\mathbb{Z}_2^{10}$	$\mathbb{Z}_2^{16}$	$\mathbb{Z}_2^{16} \times \mathbb{Z}_6$
$Z_2 \times Z_2$	$\mathbb{Z}_2^2$	$\mathbb{Z}_2^{24}$	$\mathbb{Z}_2^{34}$	$\mathbb{Z}_2^{22}$
$Z_4 \times Z_4$	$\mathbb{Z}_2^2$	$\mathbb{Z}_2^{24}$	$\mathbb{Z}_2^{34}$	$\mathbb{Z}_2^{20} \times \mathbb{Z}_4^2$
$Z_6 \times Z_6$	$\mathbb{Z}_2^2$	$\mathbb{Z}_2^{24}$	$\mathbb{Z}_2^{34}$	$\mathbb{Z}_2^{20} \times \mathbb{Z}_6^2$
$U(1)$		$\mathbb{Z}_2^7$	$\mathbb{Z}_2^5$	$\mathbb{Z} \times \mathbb{Z}_2^{16}$
$SU(2)$		$\mathbb{Z}_2^4$	$\mathbb{Z}_2^5$	$\mathbb{Z}_2^{12}$
$SO(3)$		$\mathbb{Z}_2^7$	$\mathbb{Z}_2^{12}$	$\mathbb{Z}_2^{12}$

Space group #140 $I4/mcm$

G_0	$E_{3,\infty}^3$	$E_{2,\infty}^2$	$E_{1,\infty}^1$	$E_{0,\infty}^0$
$\varnothing$		$\mathbb{Z}_2^2$	$\mathbb{Z}_2^2$	$\mathbb{Z}_2^{10}$
Z_2^T	$\mathbb{Z}_2$	$\mathbb{Z}_2^4$	$\mathbb{Z}_2^{17}$	$\mathbb{Z}_2^{10}$
Z_2		$\mathbb{Z}_2^8$	$\mathbb{Z}_2^{10}$	$\mathbb{Z}_2^{15}$
$Z_2 \times Z_2^T$	$\mathbb{Z}_2^3$	$\mathbb{Z}_2^{16}$	$\mathbb{Z}_2^{32}$	$\mathbb{Z}_2^{15}$
Z_4		$\mathbb{Z}_2^8$	$\mathbb{Z}_2^{10}$	$\mathbb{Z}_2^{13} \times \mathbb{Z}_4^2$

TABLE II (Continued.)

G_0	$E_{3,\infty}^3$	$E_{2,\infty}^2$	$E_{1,\infty}^1$	$E_{0,\infty}^0$
Z_6		$\mathbb{Z}_2^8$	$\mathbb{Z}_2^{10}$	$\mathbb{Z}_2^{14} \times \mathbb{Z}_6$
$Z_2 \times Z_2$	$\mathbb{Z}_2^2$	$\mathbb{Z}_2^{20}$	$\mathbb{Z}_2^{25}$	$\mathbb{Z}_2^{20}$
$Z_4 \times Z_4$	$\mathbb{Z}_2^2$	$\mathbb{Z}_2^{20}$	$\mathbb{Z}_2^{24} \times \mathbb{Z}_4$	$\mathbb{Z}_2^{16} \times \mathbb{Z}_4^4$
$Z_6 \times Z_6$	$\mathbb{Z}_2^2$	$\mathbb{Z}_2^{20}$	$\mathbb{Z}_2^{25}$	$\mathbb{Z}_2^{18} \times \mathbb{Z}_6^2$
$U(1)$		$\mathbb{Z}_2^5$	$\mathbb{Z}_2^2$	$\mathbb{Z} \times \mathbb{Z}_2^{13} \times \mathbb{Z}_4$
$SU(2)$		$\mathbb{Z}_2^3$	$\mathbb{Z}_2^2$	$\mathbb{Z}_2^{10}$
$SO(3)$		$\mathbb{Z}_2^5$	$\mathbb{Z}_2^9$	$\mathbb{Z}_2^{10}$

Space group #141 $I4_1/amd$

G_0	$E_{3,\infty}^3$	$E_{2,\infty}^2$	$E_{1,\infty}^1$	$E_{0,\infty}^0$
$\varnothing$		$\mathbb{Z}_2$	$\mathbb{Z}_2$	$\mathbb{Z}_2^7$
Z_2^T	$\mathbb{Z}_2$	$\mathbb{Z}_2^2$	$\mathbb{Z}_2^{11}$	$\mathbb{Z}_2^7$
Z_2		$\mathbb{Z}_2^5$	$\mathbb{Z}_2^6$	$\mathbb{Z}_2^{11}$
$Z_2 \times Z_2^T$	$\mathbb{Z}_2^3$	$\mathbb{Z}_2^{10}$	$\mathbb{Z}_2^{21}$	$\mathbb{Z}_2^{11}$
Z_4		$\mathbb{Z}_2^5$	$\mathbb{Z}_2^6$	$\mathbb{Z}_2^{10} \times \mathbb{Z}_4$
Z_6		$\mathbb{Z}_2^5$	$\mathbb{Z}_2^6$	$\mathbb{Z}_2^{10} \times \mathbb{Z}_6$
$Z_2 \times Z_2$	$\mathbb{Z}_2^2$	$\mathbb{Z}_2^{13}$	$\mathbb{Z}_2^{16}$	$\mathbb{Z}_2^{15}$
$Z_4 \times Z_4$	$\mathbb{Z}_2^2$	$\mathbb{Z}_2^{13}$	$\mathbb{Z}_2^{16}$	$\mathbb{Z}_2^{13} \times \mathbb{Z}_4^2$
$Z_6 \times Z_6$	$\mathbb{Z}_2^2$	$\mathbb{Z}_2^{13}$	$\mathbb{Z}_2^{16}$	$\mathbb{Z}_2^{13} \times \mathbb{Z}_6^2$
$U(1)$		$\mathbb{Z}_2^3$	$\mathbb{Z}_2$	$\mathbb{Z} \times \mathbb{Z}_2^{10}$
$SU(2)$		$\mathbb{Z}_2^2$	$\mathbb{Z}_2$	$\mathbb{Z}_2^7$
$SO(3)$		$\mathbb{Z}_2^3$	$\mathbb{Z}_2^6$	$\mathbb{Z}_2^7$

Space group #142 $I4_1/acd$

G_0	$E_{3,\infty}^3$	$E_{2,\infty}^2$	$E_{1,\infty}^1$	$E_{0,\infty}^0$
$\varnothing$				$\mathbb{Z}_2^5$
Z_2^T	$\mathbb{Z}_2$		$\mathbb{Z}_2^8$	$\mathbb{Z}_2^4$
Z_2		$\mathbb{Z}_2^3$	$\mathbb{Z}_2^2$	$\mathbb{Z}_2^9$
$Z_2 \times Z_2^T$	$\mathbb{Z}_2^3$	$\mathbb{Z}_2^6$	$\mathbb{Z}_2^{15}$	$\mathbb{Z}_2^8$
Z_4		$\mathbb{Z}_2^3$	$\mathbb{Z}_2^2$	$\mathbb{Z}_2^8 \times \mathbb{Z}_4$
Z_6		$\mathbb{Z}_2^3$	$\mathbb{Z}_2^2$	$\mathbb{Z}_2^8 \times \mathbb{Z}_6$
$Z_2 \times Z_2$	$\mathbb{Z}_2^2$	$\mathbb{Z}_2^9$	$\mathbb{Z}_2^9$	$\mathbb{Z}_2^{13}$
$Z_4 \times Z_4$	$\mathbb{Z}_2^2$	$\mathbb{Z}_2^9$	$\mathbb{Z}_2^9$	$\mathbb{Z}_2^{11} \times \mathbb{Z}_4^2$
$Z_6 \times Z_6$	$\mathbb{Z}_2^2$	$\mathbb{Z}_2^9$	$\mathbb{Z}_2^9$	$\mathbb{Z}_2^{11} \times \mathbb{Z}_6^2$
$U(1)$		$\mathbb{Z}_2$		$\mathbb{Z} \times \mathbb{Z}_2^8$
$SU(2)$		$\mathbb{Z}_2$		$\mathbb{Z}_2^5$
$SO(3)$		$\mathbb{Z}_2$	$\mathbb{Z}_2^5$	$\mathbb{Z}_2^5$

Space group #143 $P3$

G_0	$E_{3,\infty}^3$	$E_{2,\infty}^2$	$E_{1,\infty}^1$	$E_{0,\infty}^0$
$\varnothing$				$\mathbb{Z}_3^3$
Z_2^T	$\mathbb{Z}_2$		$\mathbb{Z}_2$	
Z_2		$\mathbb{Z}_2$		$\mathbb{Z}_2 \times \mathbb{Z}_3^3$
$Z_2 \times Z_2^T$	$\mathbb{Z}_2^3$	$\mathbb{Z}_2^2$	$\mathbb{Z}_2^2$	$\mathbb{Z}_2$
Z_4		$\mathbb{Z}_4$		$\mathbb{Z}_3^3 \times \mathbb{Z}_4$
Z_6		$\mathbb{Z}_3^2 \times \mathbb{Z}_6$	$\mathbb{Z}_3^3$	$\mathbb{Z}_3^5 \times \mathbb{Z}_6$
$Z_2 \times Z_2$	$\mathbb{Z}_2^2$	$\mathbb{Z}_2^3$	$\mathbb{Z}_2$	$\mathbb{Z}_2^2 \times \mathbb{Z}_3^3$
$Z_4 \times Z_4$	$\mathbb{Z}_4^2$	$\mathbb{Z}_4^3$	$\mathbb{Z}_4$	$\mathbb{Z}_3^3 \times \mathbb{Z}_4^2$
$Z_6 \times Z_6$	$\mathbb{Z}_6^2$	$\mathbb{Z}_3^6 \times \mathbb{Z}_6^3$	$\mathbb{Z}_3^{10} \times \mathbb{Z}_6$	$\mathbb{Z}_3^7 \times \mathbb{Z}_6^2$
$U(1)$		$\mathbb{Z}$		$\mathbb{Z} \times \mathbb{Z}_3^5$
$SU(2)$		$\mathbb{Z}$		$\mathbb{Z}_3^3$
$SO(3)$		$\mathbb{Z}$	$\mathbb{Z}_2$	$\mathbb{Z}_3^3$

Space group #144 $P3_1$

G_0	$E_{3,\infty}^3$	$E_{2,\infty}^2$	$E_{1,\infty}^1$	$E_{0,\infty}^0$
$\varnothing$				
Z_2^T	$\mathbb{Z}_2$		$\mathbb{Z}_2$	

TABLE II (Continued.)

G_0	$E_{3,\infty}^3$	$E_{2,\infty}^2$	$E_{1,\infty}^1$	$E_{0,\infty}^0$
Z_2		$\mathbb{Z}_2$		$\mathbb{Z}_2$
$Z_2 \times Z_2^T$	$\mathbb{Z}_2^3$	$\mathbb{Z}_2^2$	$\mathbb{Z}_2^2$	$\mathbb{Z}_2$
Z_4		$\mathbb{Z}_4$		$\mathbb{Z}_4$
Z_6		$\mathbb{Z}_3 \times \mathbb{Z}_6$		$\mathbb{Z}_6$
$Z_2 \times Z_2$	$\mathbb{Z}_2^2$	$\mathbb{Z}_2^3$	$\mathbb{Z}_2$	$\mathbb{Z}_2^2$
$Z_4 \times Z_4$	$\mathbb{Z}_4^2$	$\mathbb{Z}_4^3$	$\mathbb{Z}_4$	$\mathbb{Z}_4^2$
$Z_6 \times Z_6$	$\mathbb{Z}_6^2$	$\mathbb{Z}_3^3 \times \mathbb{Z}_6^3$	$\mathbb{Z}_3 \times \mathbb{Z}_6$	$\mathbb{Z}_6^2$
$U(1)$		$\mathbb{Z}$		$\mathbb{Z}$
$SU(2)$		$\mathbb{Z}$		
$SO(3)$		$\mathbb{Z}$	$\mathbb{Z}_2$	
Space group #145 $P3_2$				
$\varnothing$				
Z_2^T	$\mathbb{Z}_2$		$\mathbb{Z}_2$	
Z_2		$\mathbb{Z}_2$		$\mathbb{Z}_2$
$Z_2 \times Z_2^T$	$\mathbb{Z}_2^3$	$\mathbb{Z}_2^2$	$\mathbb{Z}_2^2$	$\mathbb{Z}_2$
Z_4		$\mathbb{Z}_4$		$\mathbb{Z}_4$
Z_6		$\mathbb{Z}_3 \times \mathbb{Z}_6$		$\mathbb{Z}_6$
$Z_2 \times Z_2$	$\mathbb{Z}_2^2$	$\mathbb{Z}_2^3$	$\mathbb{Z}_2$	$\mathbb{Z}_2^2$
$Z_4 \times Z_4$	$\mathbb{Z}_4^2$	$\mathbb{Z}_4^3$	$\mathbb{Z}_4$	$\mathbb{Z}_4^2$
$Z_6 \times Z_6$	$\mathbb{Z}_6^2$	$\mathbb{Z}_3^3 \times \mathbb{Z}_6^3$	$\mathbb{Z}_3 \times \mathbb{Z}_6$	$\mathbb{Z}_6^2$
$U(1)$		$\mathbb{Z}$		$\mathbb{Z}$
$SU(2)$		$\mathbb{Z}$		
$SO(3)$		$\mathbb{Z}$	$\mathbb{Z}_2$	
Space group #146 $R3$				
$\varnothing$				$\mathbb{Z}_3$
Z_2^T	$\mathbb{Z}_2$		$\mathbb{Z}_2$	
Z_2		$\mathbb{Z}_2$		$\mathbb{Z}_2 \times \mathbb{Z}_3$
$Z_2 \times Z_2^T$	$\mathbb{Z}_2^3$	$\mathbb{Z}_2^2$	$\mathbb{Z}_2^2$	$\mathbb{Z}_2$
Z_4		$\mathbb{Z}_4$		$\mathbb{Z}_3 \times \mathbb{Z}_4$
Z_6		$\mathbb{Z}_3 \times \mathbb{Z}_6$	$\mathbb{Z}_3$	$\mathbb{Z}_3 \times \mathbb{Z}_6$
$Z_2 \times Z_2$	$\mathbb{Z}_2^2$	$\mathbb{Z}_2^3$	$\mathbb{Z}_2$	$\mathbb{Z}_2^2 \times \mathbb{Z}_3$
$Z_4 \times Z_4$	$\mathbb{Z}_4^2$	$\mathbb{Z}_4^3$	$\mathbb{Z}_4$	$\mathbb{Z}_3 \times \mathbb{Z}_4^2$
$Z_6 \times Z_6$	$\mathbb{Z}_6^2$	$\mathbb{Z}_3^3 \times \mathbb{Z}_6^3$	$\mathbb{Z}_3^3 \times \mathbb{Z}_6$	$\mathbb{Z}_3 \times \mathbb{Z}_6^2$
$U(1)$		$\mathbb{Z}$		$\mathbb{Z} \times \mathbb{Z}_3$
$SU(2)$		$\mathbb{Z}$		$\mathbb{Z}_3$
$SO(3)$		$\mathbb{Z}$	$\mathbb{Z}_2$	$\mathbb{Z}_3$
Space group #147 $P\bar{3}$				
$\varnothing$				$\mathbb{Z}_2^4 \times \mathbb{Z}_3^2$
Z_2^T	$\mathbb{Z}_2$		$\mathbb{Z}_2^3$	$\mathbb{Z}_2^4$
Z_2		$\mathbb{Z}_2^2$		$\mathbb{Z}_2^8 \times \mathbb{Z}_3^2$
$Z_2 \times Z_2^T$	$\mathbb{Z}_2^3$	$\mathbb{Z}_2^4$	$\mathbb{Z}_2^6$	$\mathbb{Z}_2^8$
Z_4		$\mathbb{Z}_2 \times \mathbb{Z}_4$		$\mathbb{Z}_2^7 \times \mathbb{Z}_3^2 \times \mathbb{Z}_4$
Z_6		$\mathbb{Z}_6^2$	$\mathbb{Z}_3$	$\mathbb{Z}_2^6 \times \mathbb{Z}_3^2 \times \mathbb{Z}_6^2$
$Z_2 \times Z_2$	$\mathbb{Z}_2^2$	$\mathbb{Z}_2^6$	$\mathbb{Z}_2^3$	$\mathbb{Z}_2^{12} \times \mathbb{Z}_3^2$
$Z_4 \times Z_4$	$\mathbb{Z}_2^2$	$\mathbb{Z}_2^3 \times \mathbb{Z}_4^3$	$\mathbb{Z}_2^3$	$\mathbb{Z}_2^{10} \times \mathbb{Z}_3^2 \times \mathbb{Z}_4^2$
$Z_6 \times Z_6$	$\mathbb{Z}_2^2$	$\mathbb{Z}_6^6$	$\mathbb{Z}_2 \times \mathbb{Z}_3^2 \times \mathbb{Z}_6^2$	$\mathbb{Z}_2^8 \times \mathbb{Z}_3^2 \times \mathbb{Z}_6^4$
$U(1)$		$\mathbb{Z} \times \mathbb{Z}_2$		$\mathbb{Z} \times \mathbb{Z}_2^6 \times$ $\mathbb{Z}_3^2 \times \mathbb{Z}_6$
$SU(2)$		$\mathbb{Z} \times \mathbb{Z}_2$		$\mathbb{Z}_2^4 \times \mathbb{Z}_3^2$
$SO(3)$		$\mathbb{Z} \times \mathbb{Z}_2$	$\mathbb{Z}_2^3$	$\mathbb{Z}_2^4 \times \mathbb{Z}_3^2$

TABLE II (Continued.)

G_0	$E_{3,\infty}^3$	$E_{2,\infty}^2$	$E_{1,\infty}^1$	$E_{0,\infty}^0$
Space group #148 $R\bar{3}$				
$\varnothing$				$\mathbb{Z}_2^4 \times \mathbb{Z}_3$
Z_2^T	$\mathbb{Z}_2$		$\mathbb{Z}_2^3$	$\mathbb{Z}_2^4$
Z_2		$\mathbb{Z}_2^2$		$\mathbb{Z}_2^8 \times \mathbb{Z}_3$
$Z_2 \times Z_2^T$	$\mathbb{Z}_2^3$	$\mathbb{Z}_2^4$	$\mathbb{Z}_2^6$	$\mathbb{Z}_2^8$
Z_4		$\mathbb{Z}_2 \times \mathbb{Z}_4$		$\mathbb{Z}_2^7 \times \mathbb{Z}_3 \times \mathbb{Z}_4$
Z_6		$\mathbb{Z}_2 \times \mathbb{Z}_6$		$\mathbb{Z}_2^7 \times \mathbb{Z}_3 \times \mathbb{Z}_6$
$Z_2 \times Z_2$	$\mathbb{Z}_2^2$	$\mathbb{Z}_2^6$	$\mathbb{Z}_2^3$	$\mathbb{Z}_2^{12} \times \mathbb{Z}_3$
$Z_4 \times Z_4$	$\mathbb{Z}_2^2$	$\mathbb{Z}_2^3 \times \mathbb{Z}_4^3$	$\mathbb{Z}_2^3$	$\mathbb{Z}_2^{10} \times \mathbb{Z}_3 \times \mathbb{Z}_4^2$
$Z_6 \times Z_6$	$\mathbb{Z}_2^2$	$\mathbb{Z}_2^3 \times \mathbb{Z}_6^3$	$\mathbb{Z}_2^3$	$\mathbb{Z}_2^{10} \times \mathbb{Z}_3 \times \mathbb{Z}_6^2$
$U(1)$		$\mathbb{Z} \times \mathbb{Z}_2$		$\mathbb{Z} \times \mathbb{Z}_2^7 \times \mathbb{Z}_3$
$SU(2)$		$\mathbb{Z} \times \mathbb{Z}_2$		$\mathbb{Z}_2^4 \times \mathbb{Z}_3$
$SO(3)$		$\mathbb{Z} \times \mathbb{Z}_2$	$\mathbb{Z}_2^3$	$\mathbb{Z}_2^4 \times \mathbb{Z}_3$
Space group #149 $P312$				
$\varnothing$				$\mathbb{Z}_2^2$
Z_2^T	$\mathbb{Z}_2$		$\mathbb{Z}_2^5$	$\mathbb{Z}_2^2$
Z_2		$\mathbb{Z}_2^2$	$\mathbb{Z}_2^2$	$\mathbb{Z}_2^4$
$Z_2 \times Z_2^T$	$\mathbb{Z}_2^3$	$\mathbb{Z}_2^4$	$\mathbb{Z}_2^{10}$	$\mathbb{Z}_2^4$
Z_4		$\mathbb{Z}_2^2$	$\mathbb{Z}_2^2$	$\mathbb{Z}_2^3 \times \mathbb{Z}_4$
Z_6		$\mathbb{Z}_2^2$	$\mathbb{Z}_3 \times \mathbb{Z}_6^2$	$\mathbb{Z}_2^2 \times \mathbb{Z}_3 \times \mathbb{Z}_6^2$
$Z_2 \times Z_2$	$\mathbb{Z}_2^2$	$\mathbb{Z}_2^6$	$\mathbb{Z}_2^7$	$\mathbb{Z}_2^6$
$Z_4 \times Z_4$	$\mathbb{Z}_4^2$	$\mathbb{Z}_2^6$	$\mathbb{Z}_2^7$	$\mathbb{Z}_2^4 \times \mathbb{Z}_4^2$
$Z_6 \times Z_6$	$\mathbb{Z}_6^2$	$\mathbb{Z}_2^6$	$\mathbb{Z}_2 \times \mathbb{Z}_3^3 \times \mathbb{Z}_6^6$	$\mathbb{Z}_2^2 \times \mathbb{Z}_3^2 \times \mathbb{Z}_6^4$
$U(1)$				$\mathbb{Z} \times \mathbb{Z}_2^2 \times$ $\mathbb{Z}_3 \times \mathbb{Z}_6$
$SU(2)$				$\mathbb{Z}_2^2$
$SO(3)$			$\mathbb{Z}_2^3$	$\mathbb{Z}_2^2$
Space group #150 $P321$				
$\varnothing$				$\mathbb{Z}_2^2 \times \mathbb{Z}_3$
Z_2^T	$\mathbb{Z}_2$		$\mathbb{Z}_2^5$	$\mathbb{Z}_2^2$
Z_2		$\mathbb{Z}_2^2$	$\mathbb{Z}_2^2$	$\mathbb{Z}_2^4 \times \mathbb{Z}_3$
$Z_2 \times Z_2^T$	$\mathbb{Z}_2^3$	$\mathbb{Z}_2^4$	$\mathbb{Z}_2^{10}$	$\mathbb{Z}_2^4$
Z_4		$\mathbb{Z}_2^2$	$\mathbb{Z}_2^2$	$\mathbb{Z}_2^3 \times \mathbb{Z}_3 \times \mathbb{Z}_4$
Z_6		$\mathbb{Z}_2 \times \mathbb{Z}_6$	$\mathbb{Z}_6^2$	$\mathbb{Z}_2^2 \times \mathbb{Z}_3 \times \mathbb{Z}_6^2$
$Z_2 \times Z_2$	$\mathbb{Z}_2^2$	$\mathbb{Z}_2^6$	$\mathbb{Z}_2^7$	$\mathbb{Z}_2^6 \times \mathbb{Z}_3$
$Z_4 \times Z_4$	$\mathbb{Z}_4^2$	$\mathbb{Z}_2^6$	$\mathbb{Z}_2^7$	$\mathbb{Z}_2^4 \times \mathbb{Z}_3 \times \mathbb{Z}_4^2$
$Z_6 \times Z_6$	$\mathbb{Z}_6^2$	$\mathbb{Z}_2^3 \times \mathbb{Z}_6^3$	$\mathbb{Z}_2 \times \mathbb{Z}_6^6$	$\mathbb{Z}_2^2 \times \mathbb{Z}_3 \times \mathbb{Z}_6^4$
$U(1)$				$\mathbb{Z} \times \mathbb{Z}_2^2 \times$ $\mathbb{Z}_3 \times \mathbb{Z}_6$
$SU(2)$				$\mathbb{Z}_2^2 \times \mathbb{Z}_3$
$SO(3)$			$\mathbb{Z}_2^3$	$\mathbb{Z}_2^2 \times \mathbb{Z}_3$
Space group #151 $P3_112$				
$\varnothing$				$\mathbb{Z}_2^2$
Z_2^T	$\mathbb{Z}_2$		$\mathbb{Z}_2^5$	$\mathbb{Z}_2^2$
Z_2		$\mathbb{Z}_2^2$	$\mathbb{Z}_2^2$	$\mathbb{Z}_2^4$
$Z_2 \times Z_2^T$	$\mathbb{Z}_2^3$	$\mathbb{Z}_2^4$	$\mathbb{Z}_2^{10}$	$\mathbb{Z}_2^4$
Z_4		$\mathbb{Z}_2^2$	$\mathbb{Z}_2^2$	$\mathbb{Z}_2^3 \times \mathbb{Z}_4$
Z_6		$\mathbb{Z}_2^2$	$\mathbb{Z}_2^2$	$\mathbb{Z}_2^3 \times \mathbb{Z}_6$
$Z_2 \times Z_2$	$\mathbb{Z}_2^2$	$\mathbb{Z}_2^6$	$\mathbb{Z}_2^7$	$\mathbb{Z}_2^6$
$Z_4 \times Z_4$	$\mathbb{Z}_4^2$	$\mathbb{Z}_2^6$	$\mathbb{Z}_2^7$	$\mathbb{Z}_2^4 \times \mathbb{Z}_4^2$

TABLE II (Continued.)

G_0	$E_{3,\infty}^3$	$E_{2,\infty}^2$	$E_{1,\infty}^1$	$E_{0,\infty}^0$
$Z_6 \times Z_6$	$\mathbb{Z}_6^2$	$\mathbb{Z}_2^6$	$\mathbb{Z}_2^7$	$\mathbb{Z}_2^4 \times \mathbb{Z}_6^2$
$U(1)$				$\mathbb{Z} \times \mathbb{Z}_2^3$
$SU(2)$				$\mathbb{Z}_2^2$
$SO(3)$			$\mathbb{Z}_2^3$	$\mathbb{Z}_2^2$
Space group #152 $P3_121$				
$\varnothing$				$\mathbb{Z}_2^2$
Z_2^T	$\mathbb{Z}_2$		$\mathbb{Z}_2^5$	$\mathbb{Z}_2^2$
Z_2		$\mathbb{Z}_2^2$	$\mathbb{Z}_2^2$	$\mathbb{Z}_2^4$
$Z_2 \times Z_2^T$	$\mathbb{Z}_2^3$	$\mathbb{Z}_2^4$	$\mathbb{Z}_2^{10}$	$\mathbb{Z}_2^4$
Z_4		$\mathbb{Z}_2^2$	$\mathbb{Z}_2^2$	$\mathbb{Z}_2^3 \times \mathbb{Z}_4$
Z_6		$\mathbb{Z}_2 \times \mathbb{Z}_6$	$\mathbb{Z}_2^2$	$\mathbb{Z}_2^3 \times \mathbb{Z}_6$
$Z_2 \times Z_2$	$\mathbb{Z}_2^2$	$\mathbb{Z}_2^6$	$\mathbb{Z}_2^7$	$\mathbb{Z}_2^6$
$Z_4 \times Z_4$	$\mathbb{Z}_4^2$	$\mathbb{Z}_2^6$	$\mathbb{Z}_2^7$	$\mathbb{Z}_2^4 \times \mathbb{Z}_4^2$
$Z_6 \times Z_6$	$\mathbb{Z}_6^2$	$\mathbb{Z}_2^3 \times \mathbb{Z}_6^3$	$\mathbb{Z}_2^6 \times \mathbb{Z}_6$	$\mathbb{Z}_2^4 \times \mathbb{Z}_6^2$
$U(1)$				$\mathbb{Z} \times \mathbb{Z}_2^3$
$SU(2)$				$\mathbb{Z}_2^2$
$SO(3)$			$\mathbb{Z}_2^3$	$\mathbb{Z}_2^2$
Space group #153 $P3_212$				
$\varnothing$				$\mathbb{Z}_2^2$
Z_2^T	$\mathbb{Z}_2$		$\mathbb{Z}_2^5$	$\mathbb{Z}_2^2$
Z_2		$\mathbb{Z}_2^2$	$\mathbb{Z}_2^2$	$\mathbb{Z}_2^4$
$Z_2 \times Z_2^T$	$\mathbb{Z}_2^3$	$\mathbb{Z}_2^4$	$\mathbb{Z}_2^{10}$	$\mathbb{Z}_2^4$
Z_4		$\mathbb{Z}_2^2$	$\mathbb{Z}_2^2$	$\mathbb{Z}_2^3 \times \mathbb{Z}_4$
Z_6		$\mathbb{Z}_2^2$	$\mathbb{Z}_2^2$	$\mathbb{Z}_2^3 \times \mathbb{Z}_6$
$Z_2 \times Z_2$	$\mathbb{Z}_2^2$	$\mathbb{Z}_2^6$	$\mathbb{Z}_2^7$	$\mathbb{Z}_2^6$
$Z_4 \times Z_4$	$\mathbb{Z}_4^2$	$\mathbb{Z}_2^6$	$\mathbb{Z}_2^7$	$\mathbb{Z}_2^4 \times \mathbb{Z}_4^2$
$Z_6 \times Z_6$	$\mathbb{Z}_6^2$	$\mathbb{Z}_2^6$	$\mathbb{Z}_2^7$	$\mathbb{Z}_2^4 \times \mathbb{Z}_6^2$
$U(1)$				$\mathbb{Z} \times \mathbb{Z}_2^3$
$SU(2)$				$\mathbb{Z}_2^2$
$SO(3)$			$\mathbb{Z}_2^3$	$\mathbb{Z}_2^2$
Space group #154 $P3_221$				
$\varnothing$				$\mathbb{Z}_2^2$
Z_2^T	$\mathbb{Z}_2$		$\mathbb{Z}_2^5$	$\mathbb{Z}_2^2$
Z_2		$\mathbb{Z}_2^2$	$\mathbb{Z}_2^2$	$\mathbb{Z}_2^4$
$Z_2 \times Z_2^T$	$\mathbb{Z}_2^3$	$\mathbb{Z}_2^4$	$\mathbb{Z}_2^{10}$	$\mathbb{Z}_2^4$
Z_4		$\mathbb{Z}_2^2$	$\mathbb{Z}_2^2$	$\mathbb{Z}_2^3 \times \mathbb{Z}_4$
Z_6		$\mathbb{Z}_2 \times \mathbb{Z}_6$	$\mathbb{Z}_2^2$	$\mathbb{Z}_2^3 \times \mathbb{Z}_6$
$Z_2 \times Z_2$	$\mathbb{Z}_2^2$	$\mathbb{Z}_2^6$	$\mathbb{Z}_2^7$	$\mathbb{Z}_2^6$
$Z_4 \times Z_4$	$\mathbb{Z}_4^2$	$\mathbb{Z}_2^6$	$\mathbb{Z}_2^7$	$\mathbb{Z}_2^4 \times \mathbb{Z}_4^2$
$Z_6 \times Z_6$	$\mathbb{Z}_6^2$	$\mathbb{Z}_2^3 \times \mathbb{Z}_6^3$	$\mathbb{Z}_2^6 \times \mathbb{Z}_6$	$\mathbb{Z}_2^4 \times \mathbb{Z}_6^2$
$U(1)$				$\mathbb{Z} \times \mathbb{Z}_2^3$
$SU(2)$				$\mathbb{Z}_2^2$
$SO(3)$			$\mathbb{Z}_2^3$	$\mathbb{Z}_2^2$
Space group #155 $R32$				
$\varnothing$				$\mathbb{Z}_2^2$
Z_2^T	$\mathbb{Z}_2$		$\mathbb{Z}_2^5$	$\mathbb{Z}_2^2$
Z_2		$\mathbb{Z}_2^2$	$\mathbb{Z}_2^2$	$\mathbb{Z}_2^4$
$Z_2 \times Z_2^T$	$\mathbb{Z}_2^3$	$\mathbb{Z}_2^4$	$\mathbb{Z}_2^{10}$	$\mathbb{Z}_2^4$
Z_4		$\mathbb{Z}_2^2$	$\mathbb{Z}_2^2$	$\mathbb{Z}_2^3 \times \mathbb{Z}_4$

TABLE II (Continued.)

G_0	$E_{3,\infty}^3$	$E_{2,\infty}^2$	$E_{1,\infty}^1$	$E_{0,\infty}^0$
Z_6		$\mathbb{Z}_2^2$	$\mathbb{Z}_2 \times \mathbb{Z}_6$	$\mathbb{Z}_2^3 \times \mathbb{Z}_6$
$Z_2 \times Z_2$	$\mathbb{Z}_2^2$	$\mathbb{Z}_2^6$	$\mathbb{Z}_2^7$	$\mathbb{Z}_2^6$
$Z_4 \times Z_4$	$\mathbb{Z}_4^2$	$\mathbb{Z}_2^6$	$\mathbb{Z}_2^7$	$\mathbb{Z}_2^4 \times \mathbb{Z}_4^2$
$Z_6 \times Z_6$	$\mathbb{Z}_6^2$	$\mathbb{Z}_2^6$	$\mathbb{Z}_2^5 \times \mathbb{Z}_6^2$	$\mathbb{Z}_2^4 \times \mathbb{Z}_6^2$
$U(1)$				$\mathbb{Z} \times \mathbb{Z}_2^3$
$SU(2)$				$\mathbb{Z}_2^2$
$SO(3)$			$\mathbb{Z}_2^3$	$\mathbb{Z}_2^2$
Space group #156 $P3m1$				
$\varnothing$		$\mathbb{Z}_2$		$\mathbb{Z}_2$
Z_2^T	$\mathbb{Z}_2$	$\mathbb{Z}_2^2$	$\mathbb{Z}_2^4$	$\mathbb{Z}_2$
Z_2		$\mathbb{Z}_2^4$	$\mathbb{Z}_2^2$	$\mathbb{Z}_2^2$
$Z_2 \times Z_2^T$	$\mathbb{Z}_2^3$	$\mathbb{Z}_2^8$	$\mathbb{Z}_2^8$	$\mathbb{Z}_2^2$
Z_4		$\mathbb{Z}_2^4$	$\mathbb{Z}_2^2$	$\mathbb{Z}_2 \times \mathbb{Z}_4$
Z_6		$\mathbb{Z}_2^2 \times \mathbb{Z}_6^2$	$\mathbb{Z}_2^2$	$\mathbb{Z}_2 \times \mathbb{Z}_3^2 \times \mathbb{Z}_6$
$Z_2 \times Z_2$	$\mathbb{Z}_2^2$	$\mathbb{Z}_2^{10}$	$\mathbb{Z}_2^6$	$\mathbb{Z}_2^3$
$Z_4 \times Z_4$	$\mathbb{Z}_2^2$	$\mathbb{Z}_2^{10}$	$\mathbb{Z}_2^5 \times \mathbb{Z}_4$	$\mathbb{Z}_2 \times \mathbb{Z}_4^2$
$Z_6 \times Z_6$	$\mathbb{Z}_2^2$	$\mathbb{Z}_2^4 \times \mathbb{Z}_6^6$	$\mathbb{Z}_2^4 \times \mathbb{Z}_3^3 \times \mathbb{Z}_6^2$	$\mathbb{Z}_2 \times \mathbb{Z}_3^4 \times \mathbb{Z}_6^2$
$U(1)$		$\mathbb{Z}_2^3$		$\mathbb{Z} \times \mathbb{Z}_2 \times \mathbb{Z}_3^2$
$SU(2)$		$\mathbb{Z}_2^2$		$\mathbb{Z}_2$
$SO(3)$		$\mathbb{Z}_2^3$	$\mathbb{Z}_2^2$	$\mathbb{Z}_2$
Space group #157 $P31m$				
$\varnothing$		$\mathbb{Z}_2$		$\mathbb{Z}_2 \times \mathbb{Z}_3$
Z_2^T	$\mathbb{Z}_2$	$\mathbb{Z}_2^2$	$\mathbb{Z}_2^4$	$\mathbb{Z}_2$
Z_2		$\mathbb{Z}_2^4$	$\mathbb{Z}_2^2$	$\mathbb{Z}_2^2 \times \mathbb{Z}_3$
$Z_2 \times Z_2^T$	$\mathbb{Z}_2^3$	$\mathbb{Z}_2^8$	$\mathbb{Z}_2^8$	$\mathbb{Z}_2^2$
Z_4		$\mathbb{Z}_2^4$	$\mathbb{Z}_2^2$	$\mathbb{Z}_2 \times \mathbb{Z}_3 \times \mathbb{Z}_4$
Z_6		$\mathbb{Z}_2^3 \times \mathbb{Z}_6$	$\mathbb{Z}_2 \times \mathbb{Z}_6$	$\mathbb{Z}_2 \times \mathbb{Z}_3^2 \times \mathbb{Z}_6$
$Z_2 \times Z_2$	$\mathbb{Z}_2^2$	$\mathbb{Z}_2^{10}$	$\mathbb{Z}_2^6$	$\mathbb{Z}_2^3 \times \mathbb{Z}_3$
$Z_4 \times Z_4$	$\mathbb{Z}_2^2$	$\mathbb{Z}_2^{10}$	$\mathbb{Z}_2^5 \times \mathbb{Z}_4$	$\mathbb{Z}_2 \times \mathbb{Z}_3 \times \mathbb{Z}_4^2$
$Z_6 \times Z_6$	$\mathbb{Z}_2^2$	$\mathbb{Z}_2^7 \times \mathbb{Z}_6^3$	$\mathbb{Z}_2^2 \times \mathbb{Z}_3 \times \mathbb{Z}_6^4$	$\mathbb{Z}_2 \times \mathbb{Z}_3^3 \times \mathbb{Z}_6^2$
$U(1)$		$\mathbb{Z}_2^3$		$\mathbb{Z} \times \mathbb{Z}_2 \times \mathbb{Z}_3^2$
$SU(2)$		$\mathbb{Z}_2^2$		$\mathbb{Z}_2 \times \mathbb{Z}_3$
$SO(3)$		$\mathbb{Z}_2^3$	$\mathbb{Z}_2^2$	$\mathbb{Z}_2 \times \mathbb{Z}_3$
Space group #158 $P3c1$				
$\varnothing$				
Z_2^T	$\mathbb{Z}_2$		$\mathbb{Z}_2$	
Z_2		$\mathbb{Z}_2$		$\mathbb{Z}_2$
$Z_2 \times Z_2^T$	$\mathbb{Z}_2^3$	$\mathbb{Z}_2^2$	$\mathbb{Z}_2^2$	$\mathbb{Z}_2$
Z_4		$\mathbb{Z}_2$		$\mathbb{Z}_4$
Z_6		$\mathbb{Z}_3 \times \mathbb{Z}_6$		$\mathbb{Z}_3^2 \times \mathbb{Z}_6$
$Z_2 \times Z_2$	$\mathbb{Z}_2^2$	$\mathbb{Z}_2^3$	$\mathbb{Z}_2$	$\mathbb{Z}_2^2$
$Z_4 \times Z_4$	$\mathbb{Z}_2^2$	$\mathbb{Z}_2^3$	$\mathbb{Z}_4$	$\mathbb{Z}_4^2$
$Z_6 \times Z_6$	$\mathbb{Z}_2^2$	$\mathbb{Z}_3^3 \times \mathbb{Z}_6^3$	$\mathbb{Z}_3^4 \times \mathbb{Z}_6$	$\mathbb{Z}_3^4 \times \mathbb{Z}_6^2$
$U(1)$		$\mathbb{Z}_2$		$\mathbb{Z} \times \mathbb{Z}_3^2$
$SU(2)$		$\mathbb{Z}_2$		
$SO(3)$		$\mathbb{Z}_2$	$\mathbb{Z}_2$	
Space group #159 $P31c$				
$\varnothing$				$\mathbb{Z}_3$
Z_2^T	$\mathbb{Z}_2$		$\mathbb{Z}_2$	

TABLE II (Continued.)

G_0	$E^3_{3,\infty}$	$E^2_{2,\infty}$	$E^1_{1,\infty}$	$E^0_{0,\infty}$
Z_2		$\mathbb{Z}_2$		$\mathbb{Z}_2 \times \mathbb{Z}_3$
$Z_2 \times Z_2^T$	$\mathbb{Z}_2^3$	$\mathbb{Z}_2^2$	$\mathbb{Z}_2^2$	$\mathbb{Z}_2$
Z_4		$\mathbb{Z}_2$		$\mathbb{Z}_3 \times \mathbb{Z}_4$
Z_6		$\mathbb{Z}_6$	$\mathbb{Z}_3$	$\mathbb{Z}_3^2 \times \mathbb{Z}_6$
$Z_2 \times Z_2$	$\mathbb{Z}_2^2$	$\mathbb{Z}_2^3$	$\mathbb{Z}_2$	$\mathbb{Z}_2^2 \times \mathbb{Z}_3$
$Z_4 \times Z_4$	$\mathbb{Z}_2^2$	$\mathbb{Z}_2^3$	$\mathbb{Z}_4$	$\mathbb{Z}_3 \times \mathbb{Z}_4^2$
$Z_6 \times Z_6$	$\mathbb{Z}_2^2$	$\mathbb{Z}_6^3$	$\mathbb{Z}_3^4 \times \mathbb{Z}_6$	$\mathbb{Z}_3^3 \times \mathbb{Z}_6^2$
$U(1)$		$\mathbb{Z}_2$		$\mathbb{Z} \times \mathbb{Z}_3^2$
$SU(2)$		$\mathbb{Z}_2$		$\mathbb{Z}_3$
$SO(3)$		$\mathbb{Z}_2$	$\mathbb{Z}_2$	$\mathbb{Z}_3$
Space group #160 $R3m$				
$\varnothing$		$\mathbb{Z}_2$		$\mathbb{Z}_2$
Z_2^T	$\mathbb{Z}_2$	$\mathbb{Z}_2^2$	$\mathbb{Z}_2^4$	$\mathbb{Z}_2$
Z_2		$\mathbb{Z}_2^4$	$\mathbb{Z}_2^2$	$\mathbb{Z}_2^2$
$Z_2 \times Z_2^T$	$\mathbb{Z}_2^3$	$\mathbb{Z}_2^8$	$\mathbb{Z}_2^8$	$\mathbb{Z}_2^2$
Z_4		$\mathbb{Z}_2^4$	$\mathbb{Z}_2^2$	$\mathbb{Z}_2 \times \mathbb{Z}_4$
Z_6		$\mathbb{Z}_2^3 \times \mathbb{Z}_6$	$\mathbb{Z}_2^2$	$\mathbb{Z}_2 \times \mathbb{Z}_6$
$Z_2 \times Z_2$	$\mathbb{Z}_2^2$	$\mathbb{Z}_2^{10}$	$\mathbb{Z}_2^6$	$\mathbb{Z}_2^3$
$Z_4 \times Z_4$	$\mathbb{Z}_2^2$	$\mathbb{Z}_2^{10}$	$\mathbb{Z}_2^5 \times \mathbb{Z}_4$	$\mathbb{Z}_2 \times \mathbb{Z}_4^2$
$Z_6 \times Z_6$	$\mathbb{Z}_2^2$	$\mathbb{Z}_2^7 \times \mathbb{Z}_6^3$	$\mathbb{Z}_2^4 \times \mathbb{Z}_6^2$	$\mathbb{Z}_2 \times \mathbb{Z}_6^2$
$U(1)$		$\mathbb{Z}_2^3$		$\mathbb{Z} \times \mathbb{Z}_2$
$SU(2)$		$\mathbb{Z}_2^2$		$\mathbb{Z}_2$
$SO(3)$		$\mathbb{Z}_2^3$	$\mathbb{Z}_2^2$	$\mathbb{Z}_2$
Space group #161 $R3c$				
$\varnothing$				
Z_2^T	$\mathbb{Z}_2$		$\mathbb{Z}_2$	
Z_2		$\mathbb{Z}_2$		$\mathbb{Z}_2$
$Z_2 \times Z_2^T$	$\mathbb{Z}_2^3$	$\mathbb{Z}_2^2$	$\mathbb{Z}_2^2$	$\mathbb{Z}_2$
Z_4		$\mathbb{Z}_2$		$\mathbb{Z}_4$
Z_6		$\mathbb{Z}_6$		$\mathbb{Z}_6$
$Z_2 \times Z_2$	$\mathbb{Z}_2^2$	$\mathbb{Z}_2^3$	$\mathbb{Z}_2$	$\mathbb{Z}_2^2$
$Z_4 \times Z_4$	$\mathbb{Z}_2^2$	$\mathbb{Z}_2^3$	$\mathbb{Z}_4$	$\mathbb{Z}_4^2$
$Z_6 \times Z_6$	$\mathbb{Z}_2^2$	$\mathbb{Z}_6^3$	$\mathbb{Z}_3 \times \mathbb{Z}_6$	$\mathbb{Z}_6^2$
$U(1)$		$\mathbb{Z}_2$		$\mathbb{Z}$
$SU(2)$		$\mathbb{Z}_2$		
$SO(3)$		$\mathbb{Z}_2$	$\mathbb{Z}_2$	
Space group #162 $P\bar{3}1m$				
$\varnothing$		$\mathbb{Z}_2$		$\mathbb{Z}_2^8$
Z_2^T	$\mathbb{Z}_2$	$\mathbb{Z}_2^2$	$\mathbb{Z}_2^{10}$	$\mathbb{Z}_2^8$
Z_2		$\mathbb{Z}_2^5$	$\mathbb{Z}_2^5$	$\mathbb{Z}_2^{12}$
$Z_2 \times Z_2^T$	$\mathbb{Z}_2^3$	$\mathbb{Z}_2^{10}$	$\mathbb{Z}_2^{20}$	$\mathbb{Z}_2^{12}$
Z_4		$\mathbb{Z}_2^5$	$\mathbb{Z}_2^5$	$\mathbb{Z}_2^{11} \times \mathbb{Z}_4$
Z_6		$\mathbb{Z}_2^5$	$\mathbb{Z}_2^4 \times \mathbb{Z}_6$	$\mathbb{Z}_2^{10} \times \mathbb{Z}_6^2$
$Z_2 \times Z_2$	$\mathbb{Z}_2^2$	$\mathbb{Z}_2^{13}$	$\mathbb{Z}_2^{15}$	$\mathbb{Z}_2^{16}$
$Z_4 \times Z_4$	$\mathbb{Z}_2^2$	$\mathbb{Z}_2^{13}$	$\mathbb{Z}_2^{15}$	$\mathbb{Z}_2^{14} \times \mathbb{Z}_4^2$
$Z_6 \times Z_6$	$\mathbb{Z}_2^2$	$\mathbb{Z}_2^{13}$	$\mathbb{Z}_2^{12} \times \mathbb{Z}_6^3$	$\mathbb{Z}_2^{12} \times \mathbb{Z}_6^4$
$U(1)$		$\mathbb{Z}_2^3$		$\mathbb{Z} \times \mathbb{Z}_2^{10} \times \mathbb{Z}_6$
$SU(2)$		$\mathbb{Z}_2^2$		$\mathbb{Z}_2^8$
$SO(3)$		$\mathbb{Z}_2^3$	$\mathbb{Z}_2^5$	$\mathbb{Z}_2^8$

TABLE II (Continued.)

G_0	$E^3_{3,\infty}$	$E^2_{2,\infty}$	$E^1_{1,\infty}$	$E^0_{0,\infty}$
Space group #163 $P\bar{3}1c$				
$\varnothing$				$\mathbb{Z}_2^3$
Z_2^T	$\mathbb{Z}_2$		$\mathbb{Z}_2^4$	$\mathbb{Z}_2^3$
Z_2		$\mathbb{Z}_2^2$	$\mathbb{Z}_2$	$\mathbb{Z}_2^6$
$Z_2 \times Z_2^T$	$\mathbb{Z}_2^3$	$\mathbb{Z}_2^4$	$\mathbb{Z}_2^8$	$\mathbb{Z}_2^6$
Z_4		$\mathbb{Z}_2^2$	$\mathbb{Z}_2$	$\mathbb{Z}_2^5 \times \mathbb{Z}_4$
Z_6		$\mathbb{Z}_2^2$	$\mathbb{Z}_6$	$\mathbb{Z}_2^4 \times \mathbb{Z}_6^2$
$Z_2 \times Z_2$	$\mathbb{Z}_2^2$	$\mathbb{Z}_2^6$	$\mathbb{Z}_2^5$	$\mathbb{Z}_2^9$
$Z_4 \times Z_4$	$\mathbb{Z}_2^2$	$\mathbb{Z}_2^6$	$\mathbb{Z}_2^5$	$\mathbb{Z}_2^7 \times \mathbb{Z}_4^2$
$Z_6 \times Z_6$	$\mathbb{Z}_2^2$	$\mathbb{Z}_2^6$	$\mathbb{Z}_2^2 \times \mathbb{Z}_6^3$	$\mathbb{Z}_2^5 \times \mathbb{Z}_6^4$
$U(1)$		$\mathbb{Z}_2$		$\mathbb{Z} \times \mathbb{Z}_2^4 \times \mathbb{Z}_6$
$SU(2)$		$\mathbb{Z}_2$		$\mathbb{Z}_2^3$
$SO(3)$		$\mathbb{Z}_2$	$\mathbb{Z}_2^3$	$\mathbb{Z}_2^3$
Space group #164 $P\bar{3}m1$				
$\varnothing$		$\mathbb{Z}_2$		$\mathbb{Z}_2^8$
Z_2^T	$\mathbb{Z}_2$	$\mathbb{Z}_2^2$	$\mathbb{Z}_2^{10}$	$\mathbb{Z}_2^8$
Z_2		$\mathbb{Z}_2^5$	$\mathbb{Z}_2^5$	$\mathbb{Z}_2^{12}$
$Z_2 \times Z_2^T$	$\mathbb{Z}_2^3$	$\mathbb{Z}_2^{10}$	$\mathbb{Z}_2^{20}$	$\mathbb{Z}_2^{12}$
Z_4		$\mathbb{Z}_2^5$	$\mathbb{Z}_2^5$	$\mathbb{Z}_2^{11} \times \mathbb{Z}_4$
Z_6		$\mathbb{Z}_2^4 \times \mathbb{Z}_6$	$\mathbb{Z}_2^5$	$\mathbb{Z}_2^{10} \times \mathbb{Z}_6^2$
$Z_2 \times Z_2$	$\mathbb{Z}_2^2$	$\mathbb{Z}_2^{13}$	$\mathbb{Z}_2^{15}$	$\mathbb{Z}_2^{16}$
$Z_4 \times Z_4$	$\mathbb{Z}_2^2$	$\mathbb{Z}_2^{13}$	$\mathbb{Z}_2^{15}$	$\mathbb{Z}_2^{14} \times \mathbb{Z}_4^2$
$Z_6 \times Z_6$	$\mathbb{Z}_2^2$	$\mathbb{Z}_2^{10} \times \mathbb{Z}_6^3$	$\mathbb{Z}_2^{13} \times \mathbb{Z}_6^2$	$\mathbb{Z}_2^{12} \times \mathbb{Z}_6^4$
$U(1)$		$\mathbb{Z}_2^3$		$\mathbb{Z} \times \mathbb{Z}_2^{10} \times \mathbb{Z}_6$
$SU(2)$		$\mathbb{Z}_2^2$		$\mathbb{Z}_2^8$
$SO(3)$		$\mathbb{Z}_2^3$	$\mathbb{Z}_2^5$	$\mathbb{Z}_2^8$
Space group #165 $P\bar{3}c1$				
$\varnothing$				$\mathbb{Z}_2^3$
Z_2^T	$\mathbb{Z}_2$		$\mathbb{Z}_2^4$	$\mathbb{Z}_2^3$
Z_2		$\mathbb{Z}_2^2$	$\mathbb{Z}_2$	$\mathbb{Z}_2^6$
$Z_2 \times Z_2^T$	$\mathbb{Z}_2^3$	$\mathbb{Z}_2^4$	$\mathbb{Z}_2^8$	$\mathbb{Z}_2^6$
Z_4		$\mathbb{Z}_2^2$	$\mathbb{Z}_2$	$\mathbb{Z}_2^5 \times \mathbb{Z}_4$
Z_6		$\mathbb{Z}_2 \times \mathbb{Z}_6$	$\mathbb{Z}_2$	$\mathbb{Z}_2^4 \times \mathbb{Z}_6^2$
$Z_2 \times Z_2$	$\mathbb{Z}_2^2$	$\mathbb{Z}_2^6$	$\mathbb{Z}_2^5$	$\mathbb{Z}_2^9$
$Z_4 \times Z_4$	$\mathbb{Z}_2^2$	$\mathbb{Z}_2^6$	$\mathbb{Z}_2^5$	$\mathbb{Z}_2^7 \times \mathbb{Z}_4^2$
$Z_6 \times Z_6$	$\mathbb{Z}_2^2$	$\mathbb{Z}_2^3 \times \mathbb{Z}_6^3$	$\mathbb{Z}_2^3 \times \mathbb{Z}_6^2$	$\mathbb{Z}_2^5 \times \mathbb{Z}_6^4$
$U(1)$		$\mathbb{Z}_2$		$\mathbb{Z} \times \mathbb{Z}_2^4 \times \mathbb{Z}_6$
$SU(2)$		$\mathbb{Z}_2$		$\mathbb{Z}_2^3$
$SO(3)$		$\mathbb{Z}_2$	$\mathbb{Z}_2^3$	$\mathbb{Z}_2^3$
Space group #166 $R\bar{3}m$				
$\varnothing$		$\mathbb{Z}_2$		$\mathbb{Z}_2^8$
Z_2^T	$\mathbb{Z}_2$	$\mathbb{Z}_2^2$	$\mathbb{Z}_2^{10}$	$\mathbb{Z}_2^8$
Z_2		$\mathbb{Z}_2^5$	$\mathbb{Z}_2^5$	$\mathbb{Z}_2^{12}$
$Z_2 \times Z_2^T$	$\mathbb{Z}_2^3$	$\mathbb{Z}_2^{10}$	$\mathbb{Z}_2^{20}$	$\mathbb{Z}_2^{12}$
Z_4		$\mathbb{Z}_2^5$	$\mathbb{Z}_2^5$	$\mathbb{Z}_2^{11} \times \mathbb{Z}_4$
Z_6		$\mathbb{Z}_2^5$	$\mathbb{Z}_2^5$	$\mathbb{Z}_2^{11} \times \mathbb{Z}_6$
$Z_2 \times Z_2$	$\mathbb{Z}_2^2$	$\mathbb{Z}_2^{13}$	$\mathbb{Z}_2^{15}$	$\mathbb{Z}_2^{16}$
$Z_4 \times Z_4$	$\mathbb{Z}_2^2$	$\mathbb{Z}_2^{13}$	$\mathbb{Z}_2^{15}$	$\mathbb{Z}_2^{14} \times \mathbb{Z}_4^2$
$Z_6 \times Z_6$	$\mathbb{Z}_2^2$	$\mathbb{Z}_2^{13}$	$\mathbb{Z}_2^{15}$	$\mathbb{Z}_2^{14} \times \mathbb{Z}_6^2$

TABLE II (Continued.)

G_0	$E_{3,\infty}^3$	$E_{2,\infty}^2$	$E_{1,\infty}^1$	$E_{0,\infty}^0$
$U(1)$		$\mathbb{Z}_2^3$		$\mathbb{Z}\times\mathbb{Z}_2^{11}$
$SU(2)$		$\mathbb{Z}_2^2$		$\mathbb{Z}_2^8$
$SO(3)$		$\mathbb{Z}_2^3$	$\mathbb{Z}_2^5$	$\mathbb{Z}_2^8$
Space group #167 $R\bar{3}c$				
$\varnothing$				$\mathbb{Z}_2^3$
Z_2^T	$\mathbb{Z}_2$		$\mathbb{Z}_2^4$	$\mathbb{Z}_2^3$
Z_2		$\mathbb{Z}_2^2$	$\mathbb{Z}_2$	$\mathbb{Z}_2^6$
$Z_2\times Z_2^T$	$\mathbb{Z}_2^3$	$\mathbb{Z}_2^4$	$\mathbb{Z}_2^8$	$\mathbb{Z}_2^6$
Z_4		$\mathbb{Z}_2^2$	$\mathbb{Z}_2$	$\mathbb{Z}_2^5\times\mathbb{Z}_4$
Z_6		$\mathbb{Z}_2^2$	$\mathbb{Z}_2$	$\mathbb{Z}_2^5\times\mathbb{Z}_6$
$Z_2\times Z_2$	$\mathbb{Z}_2^2$	$\mathbb{Z}_2^6$	$\mathbb{Z}_2^5$	$\mathbb{Z}_2^9$
$Z_4\times Z_4$	$\mathbb{Z}_2^2$	$\mathbb{Z}_2^6$	$\mathbb{Z}_2^5$	$\mathbb{Z}_2^7\times\mathbb{Z}_4^2$
$Z_6\times Z_6$	$\mathbb{Z}_2^2$	$\mathbb{Z}_2^6$	$\mathbb{Z}_2^5$	$\mathbb{Z}_2^7\times\mathbb{Z}_6^2$
$U(1)$		$\mathbb{Z}_2$		$\mathbb{Z}\times\mathbb{Z}_2^5$
$SU(2)$		$\mathbb{Z}_2$		$\mathbb{Z}_2^3$
$SO(3)$		$\mathbb{Z}_2$	$\mathbb{Z}_2^3$	$\mathbb{Z}_2^3$
Space group #168 $P6$				
$\varnothing$				$\mathbb{Z}_2^2\times\mathbb{Z}_3^2$
Z_2^T	$\mathbb{Z}_2$		$\mathbb{Z}_2^5$	$\mathbb{Z}_2^2$
Z_2		$\mathbb{Z}_2^2$	$\mathbb{Z}_2^2$	$\mathbb{Z}_2^4\times\mathbb{Z}_3^2$
$Z_2\times Z_2^T$	$\mathbb{Z}_2^3$	$\mathbb{Z}_2^4$	$\mathbb{Z}_2^{10}$	$\mathbb{Z}_2^4$
Z_4		$\mathbb{Z}_2\times\mathbb{Z}_4$	$\mathbb{Z}_2^2$	$\mathbb{Z}_2^3\times\mathbb{Z}_3^2\times\mathbb{Z}_4$
Z_6		$\mathbb{Z}_6^2$	$\mathbb{Z}_6^2$	$\mathbb{Z}_2^2\times\mathbb{Z}_3^2\times\mathbb{Z}_6^2$
$Z_2\times Z_2$	$\mathbb{Z}_2^2$	$\mathbb{Z}_2^6$	$\mathbb{Z}_2^7$	$\mathbb{Z}_2^6\times\mathbb{Z}_3^2$
$Z_4\times Z_4$	$\mathbb{Z}_4^2$	$\mathbb{Z}_2^3\times\mathbb{Z}_4^3$	$\mathbb{Z}_2^6\times\mathbb{Z}_4$	$\mathbb{Z}_2^4\times\mathbb{Z}_3^2\times\mathbb{Z}_4^2$
$Z_6\times Z_6$	$\mathbb{Z}_6^2$	$\mathbb{Z}_6^6$	$\mathbb{Z}_6^7$	$\mathbb{Z}_2^2\times\mathbb{Z}_3^2\times\mathbb{Z}_6^4$
$U(1)$		$\mathbb{Z}$		$\mathbb{Z}\times\mathbb{Z}_2^2\times$ $\mathbb{Z}_3^2\times\mathbb{Z}_6$
$SU(2)$		$\mathbb{Z}$		$\mathbb{Z}_2^2\times\mathbb{Z}_3^2$
$SO(3)$		$\mathbb{Z}$	$\mathbb{Z}_2^3$	$\mathbb{Z}_2^2\times\mathbb{Z}_3^2$
Space group #169 $P6_1$				
$\varnothing$				
Z_2^T	$\mathbb{Z}_2$		$\mathbb{Z}_2$	
Z_2		$\mathbb{Z}_2$		$\mathbb{Z}_2$
$Z_2\times Z_2^T$	$\mathbb{Z}_2^3$	$\mathbb{Z}_2^2$	$\mathbb{Z}_2^2$	$\mathbb{Z}_2$
Z_4		$\mathbb{Z}_4$		$\mathbb{Z}_4$
Z_6		$\mathbb{Z}_6$		$\mathbb{Z}_6$
$Z_2\times Z_2$	$\mathbb{Z}_2^2$	$\mathbb{Z}_2^3$	$\mathbb{Z}_2$	$\mathbb{Z}_2^2$
$Z_4\times Z_4$	$\mathbb{Z}_4^2$	$\mathbb{Z}_4^3$	$\mathbb{Z}_4$	$\mathbb{Z}_4^2$
$Z_6\times Z_6$	$\mathbb{Z}_6^2$	$\mathbb{Z}_6^3$	$\mathbb{Z}_6$	$\mathbb{Z}_6^2$
$U(1)$		$\mathbb{Z}$		$\mathbb{Z}$
$SU(2)$		$\mathbb{Z}$		
$SO(3)$		$\mathbb{Z}$	$\mathbb{Z}_2$	
Space group #170 $P6_5$				
$\varnothing$				
Z_2^T	$\mathbb{Z}_2$		$\mathbb{Z}_2$	
Z_2		$\mathbb{Z}_2$		$\mathbb{Z}_2$
$Z_2\times Z_2^T$	$\mathbb{Z}_2^3$	$\mathbb{Z}_2^2$	$\mathbb{Z}_2^2$	$\mathbb{Z}_2$
Z_4		$\mathbb{Z}_4$		$\mathbb{Z}_4$

TABLE II (Continued.)

G_0	$E_{3,\infty}^3$	$E_{2,\infty}^2$	$E_{1,\infty}^1$	$E_{0,\infty}^0$
Z_6		$\mathbb{Z}_6$		$\mathbb{Z}_6$
$Z_2\times Z_2$	$\mathbb{Z}_2^2$	$\mathbb{Z}_2^3$	$\mathbb{Z}_2$	$\mathbb{Z}_2^2$
$Z_4\times Z_4$	$\mathbb{Z}_4^2$	$\mathbb{Z}_4^3$	$\mathbb{Z}_4$	$\mathbb{Z}_4^2$
$Z_6\times Z_6$	$\mathbb{Z}_6^2$	$\mathbb{Z}_6^3$	$\mathbb{Z}_6$	$\mathbb{Z}_6^2$
$U(1)$		$\mathbb{Z}$		$\mathbb{Z}$
$SU(2)$		$\mathbb{Z}$		
$SO(3)$		$\mathbb{Z}$	$\mathbb{Z}_2$	
Space group #171 $P6_2$				
$\varnothing$				$\mathbb{Z}_2^2$
Z_2^T	$\mathbb{Z}_2$		$\mathbb{Z}_2^5$	$\mathbb{Z}_2^2$
Z_2		$\mathbb{Z}_2^2$	$\mathbb{Z}_2^2$	$\mathbb{Z}_2^4$
$Z_2\times Z_2^T$	$\mathbb{Z}_2^3$	$\mathbb{Z}_2^4$	$\mathbb{Z}_2^{10}$	$\mathbb{Z}_2^4$
Z_4		$\mathbb{Z}_2\times\mathbb{Z}_4$	$\mathbb{Z}_2^2$	$\mathbb{Z}_2^3\times\mathbb{Z}_4$
Z_6		$\mathbb{Z}_2\times\mathbb{Z}_6$	$\mathbb{Z}_2^2$	$\mathbb{Z}_2^3\times\mathbb{Z}_6$
$Z_2\times Z_2$	$\mathbb{Z}_2^2$	$\mathbb{Z}_2^6$	$\mathbb{Z}_2^7$	$\mathbb{Z}_2^6$
$Z_4\times Z_4$	$\mathbb{Z}_4^2$	$\mathbb{Z}_2^3\times\mathbb{Z}_4^3$	$\mathbb{Z}_2^6\times\mathbb{Z}_4$	$\mathbb{Z}_2^4\times\mathbb{Z}_4^2$
$Z_6\times Z_6$	$\mathbb{Z}_6^2$	$\mathbb{Z}_2^3\times\mathbb{Z}_6^3$	$\mathbb{Z}_2^6\times\mathbb{Z}_6$	$\mathbb{Z}_2^4\times\mathbb{Z}_6^2$
$U(1)$		$\mathbb{Z}$		$\mathbb{Z}\times\mathbb{Z}_2^3$
$SU(2)$		$\mathbb{Z}$		$\mathbb{Z}_2^2$
$SO(3)$		$\mathbb{Z}$	$\mathbb{Z}_2^3$	$\mathbb{Z}_2^2$
Space group #172 $P6_4$				
$\varnothing$				$\mathbb{Z}_2^2$
Z_2^T	$\mathbb{Z}_2$		$\mathbb{Z}_2^5$	$\mathbb{Z}_2^2$
Z_2		$\mathbb{Z}_2^2$	$\mathbb{Z}_2^2$	$\mathbb{Z}_2^4$
$Z_2\times Z_2^T$	$\mathbb{Z}_2^3$	$\mathbb{Z}_2^4$	$\mathbb{Z}_2^{10}$	$\mathbb{Z}_2^4$
Z_4		$\mathbb{Z}_2\times\mathbb{Z}_4$	$\mathbb{Z}_2^2$	$\mathbb{Z}_2^3\times\mathbb{Z}_4$
Z_6		$\mathbb{Z}_2\times\mathbb{Z}_6$	$\mathbb{Z}_2^2$	$\mathbb{Z}_2^3\times\mathbb{Z}_6$
$Z_2\times Z_2$	$\mathbb{Z}_2^2$	$\mathbb{Z}_2^6$	$\mathbb{Z}_2^7$	$\mathbb{Z}_2^6$
$Z_4\times Z_4$	$\mathbb{Z}_4^2$	$\mathbb{Z}_2^3\times\mathbb{Z}_4^3$	$\mathbb{Z}_2^6\times\mathbb{Z}_4$	$\mathbb{Z}_2^4\times\mathbb{Z}_4^2$
$Z_6\times Z_6$	$\mathbb{Z}_6^2$	$\mathbb{Z}_2^3\times\mathbb{Z}_6^3$	$\mathbb{Z}_2^6\times\mathbb{Z}_6$	$\mathbb{Z}_2^4\times\mathbb{Z}_6^2$
$U(1)$		$\mathbb{Z}$		$\mathbb{Z}\times\mathbb{Z}_2^3$
$SU(2)$		$\mathbb{Z}$		$\mathbb{Z}_2^2$
$SO(3)$		$\mathbb{Z}$	$\mathbb{Z}_2^3$	$\mathbb{Z}_2^2$
Space group #173 $P6_3$				
$\varnothing$				$\mathbb{Z}_3^2$
Z_2^T	$\mathbb{Z}_2$		$\mathbb{Z}_2$	
Z_2		$\mathbb{Z}_2$		$\mathbb{Z}_2\times\mathbb{Z}_3^2$
$Z_2\times Z_2^T$	$\mathbb{Z}_2^3$	$\mathbb{Z}_2^2$	$\mathbb{Z}_2^2$	$\mathbb{Z}_2$
Z_4		$\mathbb{Z}_4$		$\mathbb{Z}_3^2\times\mathbb{Z}_4$
Z_6		$\mathbb{Z}_3\times\mathbb{Z}_6$	$\mathbb{Z}_3^2$	$\mathbb{Z}_3^3\times\mathbb{Z}_6$
$Z_2\times Z_2$	$\mathbb{Z}_2^2$	$\mathbb{Z}_2^3$	$\mathbb{Z}_2$	$\mathbb{Z}_2^2\times\mathbb{Z}_3^2$
$Z_4\times Z_4$	$\mathbb{Z}_4^2$	$\mathbb{Z}_4^3$	$\mathbb{Z}_4$	$\mathbb{Z}_3^2\times\mathbb{Z}_4^2$
$Z_6\times Z_6$	$\mathbb{Z}_6^2$	$\mathbb{Z}_3^3\times\mathbb{Z}_6^3$	$\mathbb{Z}_3^6\times\mathbb{Z}_6$	$\mathbb{Z}_3^4\times\mathbb{Z}_6^2$
$U(1)$		$\mathbb{Z}$		$\mathbb{Z}\times\mathbb{Z}_3^3$
$SU(2)$		$\mathbb{Z}$		$\mathbb{Z}_3^2$
$SO(3)$		$\mathbb{Z}$	$\mathbb{Z}_2$	$\mathbb{Z}_3^2$
Space group #174 $P\bar{6}$				
$\varnothing$		$\mathbb{Z}_2^2$		$\mathbb{Z}_2^2\times\mathbb{Z}_3^3$
Z_2^T	$\mathbb{Z}_2$	$\mathbb{Z}_2^4$	$\mathbb{Z}_2$	$\mathbb{Z}_2^2$

TABLE II (Continued.)

G_0	$E^3_{3,\infty}$	$E^2_{2,\infty}$	$E^1_{1,\infty}$	$E^0_{0,\infty}$
Z_2		$\mathbb{Z}_2^6$		$\mathbb{Z}_2^4 \times \mathbb{Z}_3^3$
$Z_2 \times Z_2^T$	$\mathbb{Z}_2^3$	$\mathbb{Z}_2^{12}$	$\mathbb{Z}_2^2$	$\mathbb{Z}_2^4$
Z_4		$\mathbb{Z}_2^5 \times \mathbb{Z}_4$		$\mathbb{Z}_2^3 \times \mathbb{Z}_3^3 \times \mathbb{Z}_4$
Z_6		$\mathbb{Z}_2^5 \times \mathbb{Z}_6$		$\mathbb{Z}_2^2 \times \mathbb{Z}_3^4 \times \mathbb{Z}_6^2$
$Z_2 \times Z_2$	$\mathbb{Z}_2^2$	$\mathbb{Z}_2^{14}$	$\mathbb{Z}_2$	$\mathbb{Z}_2^6 \times \mathbb{Z}_3^3$
$Z_4 \times Z_4$	$\mathbb{Z}_2^2$	$\mathbb{Z}_2^{11} \times \mathbb{Z}_4^3$	$\mathbb{Z}_2$	$\mathbb{Z}_2^4 \times \mathbb{Z}_3^3 \times \mathbb{Z}_4^2$
$Z_6 \times Z_6$	$\mathbb{Z}_2^2$	$\mathbb{Z}_2^{11} \times \mathbb{Z}_6^3$	$\mathbb{Z}_3 \times \mathbb{Z}_6$	$\mathbb{Z}_2^2 \times \mathbb{Z}_3^5 \times \mathbb{Z}_6^4$
$U(1)$		$\mathbb{Z} \times \mathbb{Z}_2^5$		$\mathbb{Z} \times \mathbb{Z}_2^2 \times \mathbb{Z}_3^4 \times \mathbb{Z}_6$
$SU(2)$		$\mathbb{Z} \times \mathbb{Z}_2^3$		$\mathbb{Z}_2^2 \times \mathbb{Z}_3^3$
$SO(3)$		$\mathbb{Z} \times \mathbb{Z}_2^5$	$\mathbb{Z}_2$	$\mathbb{Z}_2^2 \times \mathbb{Z}_3^3$

Space group #175 $P6/m$

G_0	$E^3_{3,\infty}$	$E^2_{2,\infty}$	$E^1_{1,\infty}$	$E^0_{0,\infty}$
$\varnothing$		$\mathbb{Z}_2^2$		$\mathbb{Z}_2^8 \times \mathbb{Z}_3^2$
Z_2^T	$\mathbb{Z}_2$	$\mathbb{Z}_2^4$	$\mathbb{Z}_2^8$	$\mathbb{Z}_2^8$
Z_2		$\mathbb{Z}_2^7$	$\mathbb{Z}_2^4$	$\mathbb{Z}_2^{12} \times \mathbb{Z}_3^2$
$Z_2 \times Z_2^T$	$\mathbb{Z}_2^3$	$\mathbb{Z}_2^{14}$	$\mathbb{Z}_2^{16}$	$\mathbb{Z}_2^{12}$
Z_4		$\mathbb{Z}_2^6 \times \mathbb{Z}_4$	$\mathbb{Z}_2^4$	$\mathbb{Z}_2^{11} \times \mathbb{Z}_3^2 \times \mathbb{Z}_4$
Z_6		$\mathbb{Z}_2^6 \times \mathbb{Z}_6$	$\mathbb{Z}_2^4$	$\mathbb{Z}_2^{10} \times \mathbb{Z}_3^2 \times \mathbb{Z}_6^2$
$Z_2 \times Z_2$	$\mathbb{Z}_2^2$	$\mathbb{Z}_2^{17}$	$\mathbb{Z}_2^{12}$	$\mathbb{Z}_2^{16} \times \mathbb{Z}_3^2$
$Z_4 \times Z_4$	$\mathbb{Z}_2^2$	$\mathbb{Z}_2^{14} \times \mathbb{Z}_4^3$	$\mathbb{Z}_2^{12}$	$\mathbb{Z}_2^{14} \times \mathbb{Z}_3^2 \times \mathbb{Z}_4^2$
$Z_6 \times Z_6$	$\mathbb{Z}_2^2$	$\mathbb{Z}_2^{14} \times \mathbb{Z}_6^3$	$\mathbb{Z}_2^{11} \times \mathbb{Z}_6$	$\mathbb{Z}_2^{12} \times \mathbb{Z}_3^2 \times \mathbb{Z}_6^4$
$U(1)$		$\mathbb{Z} \times \mathbb{Z}_2^5$		$\mathbb{Z} \times \mathbb{Z}_2^{10} \times \mathbb{Z}_3^2 \times \mathbb{Z}_6$
$SU(2)$		$\mathbb{Z} \times \mathbb{Z}_2^3$		$\mathbb{Z}_2^8 \times \mathbb{Z}_3^2$
$SO(3)$		$\mathbb{Z} \times \mathbb{Z}_2^5$	$\mathbb{Z}_2^4$	$\mathbb{Z}_2^8 \times \mathbb{Z}_3^2$

Space group #176 $P6_3/m$

G_0	$E^3_{3,\infty}$	$E^2_{2,\infty}$	$E^1_{1,\infty}$	$E^0_{0,\infty}$
$\varnothing$		$\mathbb{Z}_2$		$\mathbb{Z}_2^3 \times \mathbb{Z}_3^3$
Z_2^T	$\mathbb{Z}_2$	$\mathbb{Z}_2^2$	$\mathbb{Z}_2^2$	$\mathbb{Z}_2^3$
Z_2		$\mathbb{Z}_2^4$		$\mathbb{Z}_2^6 \times \mathbb{Z}_3^2$
$Z_2 \times Z_2^T$	$\mathbb{Z}_2^3$	$\mathbb{Z}_2^8$	$\mathbb{Z}_2^4$	$\mathbb{Z}_2^6$
Z_4		$\mathbb{Z}_2^3 \times \mathbb{Z}_4$		$\mathbb{Z}_2^5 \times \mathbb{Z}_3^2 \times \mathbb{Z}_4$
Z_6		$\mathbb{Z}_2^3 \times \mathbb{Z}_6$		$\mathbb{Z}_2^4 \times \mathbb{Z}_3^2 \times \mathbb{Z}_6^2$
$Z_2 \times Z_2$	$\mathbb{Z}_2^2$	$\mathbb{Z}_2^{10}$	$\mathbb{Z}_2^2$	$\mathbb{Z}_2^9 \times \mathbb{Z}_3^2$
$Z_4 \times Z_4$	$\mathbb{Z}_2^2$	$\mathbb{Z}_2^7 \times \mathbb{Z}_4^3$	$\mathbb{Z}_2^2$	$\mathbb{Z}_2^7 \times \mathbb{Z}_3^2 \times \mathbb{Z}_4^2$
$Z_6 \times Z_6$	$\mathbb{Z}_2^2$	$\mathbb{Z}_2^7 \times \mathbb{Z}_6^3$	$\mathbb{Z}_2 \times \mathbb{Z}_6$	$\mathbb{Z}_2^5 \times \mathbb{Z}_3^2 \times \mathbb{Z}_6^4$
$U(1)$		$\mathbb{Z} \times \mathbb{Z}_2^3$		$\mathbb{Z} \times \mathbb{Z}_2^4 \times \mathbb{Z}_3^2 \times \mathbb{Z}_6$
$SU(2)$		$\mathbb{Z} \times \mathbb{Z}_2^2$		$\mathbb{Z}_2^3 \times \mathbb{Z}_3^3$
$SO(3)$		$\mathbb{Z} \times \mathbb{Z}_2^3$	$\mathbb{Z}_2^2$	$\mathbb{Z}_2^3 \times \mathbb{Z}_3^2$

Space group #177 $P622$

G_0	$E^3_{3,\infty}$	$E^2_{2,\infty}$	$E^1_{1,\infty}$	$E^0_{0,\infty}$
$\varnothing$				$\mathbb{Z}_2^8$
Z_2^T	$\mathbb{Z}_2$		$\mathbb{Z}_2^{12}$	$\mathbb{Z}_2^8$
Z_2		$\mathbb{Z}_2^3$	$\mathbb{Z}_2^6$	$\mathbb{Z}_2^{12}$
$Z_2 \times Z_2^T$	$\mathbb{Z}_2^3$	$\mathbb{Z}_2^6$	$\mathbb{Z}_2^{24}$	$\mathbb{Z}_2^{12}$
Z_4		$\mathbb{Z}_2^3$	$\mathbb{Z}_2^6$	$\mathbb{Z}_2^{11} \times \mathbb{Z}_4$
Z_6		$\mathbb{Z}_2^3$	$\mathbb{Z}_2^4 \times \mathbb{Z}_6^2$	$\mathbb{Z}_2^{10} \times \mathbb{Z}_6^2$
$Z_2 \times Z_2$	$\mathbb{Z}_2^2$	$\mathbb{Z}_2^9$	$\mathbb{Z}_2^{18}$	$\mathbb{Z}_2^{16}$
$Z_4 \times Z_4$	$\mathbb{Z}_4^2$	$\mathbb{Z}_2^9$	$\mathbb{Z}_2^{18}$	$\mathbb{Z}_2^{14} \times \mathbb{Z}_4^2$
$Z_6 \times Z_6$	$\mathbb{Z}_6^2$	$\mathbb{Z}_2^9$	$\mathbb{Z}_2^{13} \times \mathbb{Z}_6^5$	$\mathbb{Z}_2^{12} \times \mathbb{Z}_6^4$
$U(1)$				$\mathbb{Z} \times \mathbb{Z}_2^{10} \times \mathbb{Z}_6$

TABLE II (Continued.)

G_0	$E^3_{3,\infty}$	$E^2_{2,\infty}$	$E^1_{1,\infty}$	$E^0_{0,\infty}$
$SU(2)$				$\mathbb{Z}_2^8$
$SO(3)$			$\mathbb{Z}_2^6$	$\mathbb{Z}_2^8$

Space group #178 $P6_122$

G_0	$E^3_{3,\infty}$	$E^2_{2,\infty}$	$E^1_{1,\infty}$	$E^0_{0,\infty}$
$\varnothing$				$\mathbb{Z}_2^2$
Z_2^T	$\mathbb{Z}_2$		$\mathbb{Z}_2^5$	$\mathbb{Z}_2^2$
Z_2		$\mathbb{Z}_2^2$	$\mathbb{Z}_2^2$	$\mathbb{Z}_2^4$
$Z_2 \times Z_2^T$	$\mathbb{Z}_2^3$	$\mathbb{Z}_2^4$	$\mathbb{Z}_2^{10}$	$\mathbb{Z}_2^4$
Z_4		$\mathbb{Z}_2^2$	$\mathbb{Z}_2^2$	$\mathbb{Z}_2^3 \times \mathbb{Z}_4$
Z_6		$\mathbb{Z}_2^2$	$\mathbb{Z}_2^2$	$\mathbb{Z}_2^3 \times \mathbb{Z}_6$
$Z_2 \times Z_2$	$\mathbb{Z}_2^2$	$\mathbb{Z}_2^6$	$\mathbb{Z}_2^7$	$\mathbb{Z}_2^6$
$Z_4 \times Z_4$	$\mathbb{Z}_4^2$	$\mathbb{Z}_2^6$	$\mathbb{Z}_2^7$	$\mathbb{Z}_2^4 \times \mathbb{Z}_4^2$
$Z_6 \times Z_6$	$\mathbb{Z}_6^2$	$\mathbb{Z}_2^6$	$\mathbb{Z}_2^7$	$\mathbb{Z}_2^4 \times \mathbb{Z}_6^2$
$U(1)$				$\mathbb{Z} \times \mathbb{Z}_2^3$
$SU(2)$				$\mathbb{Z}_2^2$
$SO(3)$			$\mathbb{Z}_2^3$	$\mathbb{Z}_2^2$

Space group #179 $P6_522$

G_0	$E^3_{3,\infty}$	$E^2_{2,\infty}$	$E^1_{1,\infty}$	$E^0_{0,\infty}$
$\varnothing$				$\mathbb{Z}_2^2$
Z_2^T	$\mathbb{Z}_2$		$\mathbb{Z}_2^5$	$\mathbb{Z}_2^2$
Z_2		$\mathbb{Z}_2^2$	$\mathbb{Z}_2^2$	$\mathbb{Z}_2^4$
$Z_2 \times Z_2^T$	$\mathbb{Z}_2^3$	$\mathbb{Z}_2^4$	$\mathbb{Z}_2^{10}$	$\mathbb{Z}_2^4$
Z_4		$\mathbb{Z}_2^2$	$\mathbb{Z}_2^2$	$\mathbb{Z}_2^3 \times \mathbb{Z}_4$
Z_6		$\mathbb{Z}_2^2$	$\mathbb{Z}_2^2$	$\mathbb{Z}_2^3 \times \mathbb{Z}_6$
$Z_2 \times Z_2$	$\mathbb{Z}_2^2$	$\mathbb{Z}_2^6$	$\mathbb{Z}_2^7$	$\mathbb{Z}_2^6$
$Z_4 \times Z_4$	$\mathbb{Z}_4^2$	$\mathbb{Z}_2^6$	$\mathbb{Z}_2^7$	$\mathbb{Z}_2^4 \times \mathbb{Z}_4^2$
$Z_6 \times Z_6$	$\mathbb{Z}_6^2$	$\mathbb{Z}_2^6$	$\mathbb{Z}_2^7$	$\mathbb{Z}_2^4 \times \mathbb{Z}_6^2$
$U(1)$				$\mathbb{Z} \times \mathbb{Z}_2^3$
$SU(2)$				$\mathbb{Z}_2^2$
$SO(3)$			$\mathbb{Z}_2^3$	$\mathbb{Z}_2^2$

Space group #180 $P6_222$

G_0	$E^3_{3,\infty}$	$E^2_{2,\infty}$	$E^1_{1,\infty}$	$E^0_{0,\infty}$
$\varnothing$				$\mathbb{Z}_2^8$
Z_2^T	$\mathbb{Z}_2$		$\mathbb{Z}_2^{12}$	$\mathbb{Z}_2^8$
Z_2		$\mathbb{Z}_2^3$	$\mathbb{Z}_2^6$	$\mathbb{Z}_2^{12}$
$Z_2 \times Z_2^T$	$\mathbb{Z}_2^3$	$\mathbb{Z}_2^6$	$\mathbb{Z}_2^{24}$	$\mathbb{Z}_2^{12}$
Z_4		$\mathbb{Z}_2^3$	$\mathbb{Z}_2^6$	$\mathbb{Z}_2^{11} \times \mathbb{Z}_4$
Z_6		$\mathbb{Z}_2^3$	$\mathbb{Z}_2^6$	$\mathbb{Z}_2^{11} \times \mathbb{Z}_6$
$Z_2 \times Z_2$	$\mathbb{Z}_2^2$	$\mathbb{Z}_2^9$	$\mathbb{Z}_2^{18}$	$\mathbb{Z}_2^{16}$
$Z_4 \times Z_4$	$\mathbb{Z}_4^2$	$\mathbb{Z}_2^9$	$\mathbb{Z}_2^{18}$	$\mathbb{Z}_2^{14} \times \mathbb{Z}_4^2$
$Z_6 \times Z_6$	$\mathbb{Z}_6^2$	$\mathbb{Z}_2^9$	$\mathbb{Z}_2^{18}$	$\mathbb{Z}_2^{14} \times \mathbb{Z}_6^2$
$U(1)$				$\mathbb{Z} \times \mathbb{Z}_2^{11}$
$SU(2)$				$\mathbb{Z}_2^8$
$SO(3)$			$\mathbb{Z}_2^6$	$\mathbb{Z}_2^8$

Space group #181 $P6_422$

G_0	$E^3_{3,\infty}$	$E^2_{2,\infty}$	$E^1_{1,\infty}$	$E^0_{0,\infty}$
$\varnothing$				$\mathbb{Z}_2^8$
Z_2^T	$\mathbb{Z}_2$		$\mathbb{Z}_2^{12}$	$\mathbb{Z}_2^8$
Z_2		$\mathbb{Z}_2^3$	$\mathbb{Z}_2^6$	$\mathbb{Z}_2^{12}$
$Z_2 \times Z_2^T$	$\mathbb{Z}_2^3$	$\mathbb{Z}_2^6$	$\mathbb{Z}_2^{24}$	$\mathbb{Z}_2^{12}$
Z_4		$\mathbb{Z}_2^3$	$\mathbb{Z}_2^6$	$\mathbb{Z}_2^{11} \times \mathbb{Z}_4$
Z_6		$\mathbb{Z}_2^3$	$\mathbb{Z}_2^6$	$\mathbb{Z}_2^{11} \times \mathbb{Z}_6$
$Z_2 \times Z_2$	$\mathbb{Z}_2^2$	$\mathbb{Z}_2^9$	$\mathbb{Z}_2^{18}$	$\mathbb{Z}_2^{16}$

TABLE II (Continued.)

G_0	$E^3_{3,\infty}$	$E^2_{2,\infty}$	$E^1_{1,\infty}$	$E^0_{0,\infty}$
$Z_4 \times Z_4$	$\mathbb{Z}_4^2$	$\mathbb{Z}_2^9$	$\mathbb{Z}_2^{18}$	$\mathbb{Z}_2^{14} \times \mathbb{Z}_4^2$
$Z_6 \times Z_6$	$\mathbb{Z}_6^2$	$\mathbb{Z}_2^9$	$\mathbb{Z}_2^{18}$	$\mathbb{Z}_2^{14} \times \mathbb{Z}_6^2$
$U(1)$				$\mathbb{Z} \times \mathbb{Z}_2^{11}$
$SU(2)$				$\mathbb{Z}_2^8$
$SO(3)$			$\mathbb{Z}_2^6$	$\mathbb{Z}_2^8$
Space group #182 $P6_322$				
$\varnothing$				$\mathbb{Z}_2^2$
Z_2^T	$\mathbb{Z}_2$		$\mathbb{Z}_2^5$	$\mathbb{Z}_2^2$
Z_2		$\mathbb{Z}_2^2$	$\mathbb{Z}_2^2$	$\mathbb{Z}_2^4$
$Z_2 \times Z_2^T$	$\mathbb{Z}_2^3$	$\mathbb{Z}_2^4$	$\mathbb{Z}_2^{10}$	$\mathbb{Z}_2^4$
Z_4		$\mathbb{Z}_2^2$	$\mathbb{Z}_2^2$	$\mathbb{Z}_2^3 \times \mathbb{Z}_4$
Z_6		$\mathbb{Z}_2^2$	$\mathbb{Z}_6^2$	$\mathbb{Z}_2^2 \times \mathbb{Z}_6^2$
$Z_2 \times Z_2$	$\mathbb{Z}_2^2$	$\mathbb{Z}_2^6$	$\mathbb{Z}_2^7$	$\mathbb{Z}_2^6$
$Z_4 \times Z_4$	$\mathbb{Z}_4^2$	$\mathbb{Z}_2^6$	$\mathbb{Z}_2^7$	$\mathbb{Z}_2^4 \times \mathbb{Z}_4^2$
$Z_6 \times Z_6$	$\mathbb{Z}_6^2$	$\mathbb{Z}_2^6$	$\mathbb{Z}_2^2 \times \mathbb{Z}_6^5$	$\mathbb{Z}_2^2 \times \mathbb{Z}_6^4$
$U(1)$				$\mathbb{Z} \times \mathbb{Z}_2^2 \times \mathbb{Z}_6$
$SU(2)$				$\mathbb{Z}_2^2$
$SO(3)$			$\mathbb{Z}_2^3$	$\mathbb{Z}_2^2$
Space group #183 $P6mm$				
$\varnothing$		$\mathbb{Z}_2^2$	$\mathbb{Z}_2^2$	$\mathbb{Z}_2^4$
Z_2^T	$\mathbb{Z}_2$	$\mathbb{Z}_2^4$	$\mathbb{Z}_2^{12}$	$\mathbb{Z}_2^4$
Z_2		$\mathbb{Z}_2^7$	$\mathbb{Z}_2^8$	$\mathbb{Z}_2^6$
$Z_2 \times Z_2^T$	$\mathbb{Z}_2^3$	$\mathbb{Z}_2^{14}$	$\mathbb{Z}_2^{22}$	$\mathbb{Z}_2^6$
Z_4		$\mathbb{Z}_2^7$	$\mathbb{Z}_2^8$	$\mathbb{Z}_2^5 \times \mathbb{Z}_4$
Z_6		$\mathbb{Z}_2^6 \times \mathbb{Z}_6$	$\mathbb{Z}_2^8$	$\mathbb{Z}_2^4 \times \mathbb{Z}_6^2$
$Z_2 \times Z_2$	$\mathbb{Z}_2^2$	$\mathbb{Z}_2^{17}$	$\mathbb{Z}_2^{18}$	$\mathbb{Z}_2^8$
$Z_4 \times Z_4$	$\mathbb{Z}_2^2$	$\mathbb{Z}_2^{17}$	$\mathbb{Z}_2^{17} \times \mathbb{Z}_4$	$\mathbb{Z}_2^6 \times \mathbb{Z}_4^2$
$Z_6 \times Z_6$	$\mathbb{Z}_2^2$	$\mathbb{Z}_2^{14} \times \mathbb{Z}_6^3$	$\mathbb{Z}_2^{15} \times \mathbb{Z}_6^3$	$\mathbb{Z}_2^4 \times \mathbb{Z}_6^4$
$U(1)$		$\mathbb{Z}_2^5$	$\mathbb{Z}_2^2$	$\mathbb{Z} \times \mathbb{Z}_2^4 \times \mathbb{Z}_6$
$SU(2)$		$\mathbb{Z}_2^3$	$\mathbb{Z}_2^2$	$\mathbb{Z}_2^4$
$SO(3)$		$\mathbb{Z}_2^5$	$\mathbb{Z}_2^6$	$\mathbb{Z}_2^4$
Space group #184 $P6cc$				
$\varnothing$				$\mathbb{Z}_2^2$
Z_2^T	$\mathbb{Z}_2$		$\mathbb{Z}_2^5$	$\mathbb{Z}_2^2$
Z_2		$\mathbb{Z}_2^2$	$\mathbb{Z}_2^2$	$\mathbb{Z}_2^4$
$Z_2 \times Z_2^T$	$\mathbb{Z}_2^3$	$\mathbb{Z}_2^4$	$\mathbb{Z}_2^{10}$	$\mathbb{Z}_2^4$
Z_4		$\mathbb{Z}_2^2$	$\mathbb{Z}_2^2$	$\mathbb{Z}_2^3 \times \mathbb{Z}_4$
Z_6		$\mathbb{Z}_2 \times \mathbb{Z}_6$	$\mathbb{Z}_2^2$	$\mathbb{Z}_2^2 \times \mathbb{Z}_6^2$
$Z_2 \times Z_2$	$\mathbb{Z}_2^2$	$\mathbb{Z}_2^6$	$\mathbb{Z}_2^7$	$\mathbb{Z}_2^6$
$Z_4 \times Z_4$	$\mathbb{Z}_2^2$	$\mathbb{Z}_2^6$	$\mathbb{Z}_2^6 \times \mathbb{Z}_4$	$\mathbb{Z}_2^4 \times \mathbb{Z}_4^2$
$Z_6 \times Z_6$	$\mathbb{Z}_2^2$	$\mathbb{Z}_2^3 \times \mathbb{Z}_6^3$	$\mathbb{Z}_2^4 \times \mathbb{Z}_6^3$	$\mathbb{Z}_2^2 \times \mathbb{Z}_6^4$
$U(1)$		$\mathbb{Z}_2$		$\mathbb{Z} \times \mathbb{Z}_2^2 \times \mathbb{Z}_6$
$SU(2)$		$\mathbb{Z}_2$		$\mathbb{Z}_2^2$
$SO(3)$		$\mathbb{Z}_2$	$\mathbb{Z}_2^3$	$\mathbb{Z}_2^2$
Space group #185 $P6_3cm$				
$\varnothing$		$\mathbb{Z}_2$		$\mathbb{Z}_2$
Z_2^T	$\mathbb{Z}_2$	$\mathbb{Z}_2^2$	$\mathbb{Z}_2^4$	$\mathbb{Z}_2$
Z_2		$\mathbb{Z}_2^4$	$\mathbb{Z}_2^2$	$\mathbb{Z}_2^2$
$Z_2 \times Z_2^T$	$\mathbb{Z}_2^3$	$\mathbb{Z}_2^8$	$\mathbb{Z}_2^8$	$\mathbb{Z}_2^2$

TABLE II (Continued.)

G_0	$E^3_{3,\infty}$	$E^2_{2,\infty}$	$E^1_{1,\infty}$	$E^0_{0,\infty}$
Z_4		$\mathbb{Z}_2^4$	$\mathbb{Z}_2^2$	$\mathbb{Z}_2 \times \mathbb{Z}_4$
Z_6		$\mathbb{Z}_2^3 \times \mathbb{Z}_6$	$\mathbb{Z}_2^2$	$\mathbb{Z}_2 \times \mathbb{Z}_3 \times \mathbb{Z}_6$
$Z_2 \times Z_2$	$\mathbb{Z}_2^2$	$\mathbb{Z}_2^{10}$	$\mathbb{Z}_2^6$	$\mathbb{Z}_2^3$
$Z_4 \times Z_4$	$\mathbb{Z}_2^2$	$\mathbb{Z}_2^{10}$	$\mathbb{Z}_2^5 \times \mathbb{Z}_4$	$\mathbb{Z}_2 \times \mathbb{Z}_4^2$
$Z_6 \times Z_6$	$\mathbb{Z}_2^2$	$\mathbb{Z}_2^7 \times \mathbb{Z}_6^3$	$\mathbb{Z}_2^4 \times \mathbb{Z}_3 \times \mathbb{Z}_6^2$	$\mathbb{Z}_2 \times \mathbb{Z}_3^2 \times \mathbb{Z}_6^2$
$U(1)$		$\mathbb{Z}_2^3$		$\mathbb{Z} \times \mathbb{Z}_2 \times \mathbb{Z}_3$
$SU(2)$		$\mathbb{Z}_2^2$		$\mathbb{Z}_2$
$SO(3)$		$\mathbb{Z}_2^3$	$\mathbb{Z}_2^2$	$\mathbb{Z}_2$
Space group #186 $P6_3mc$				
$\varnothing$		$\mathbb{Z}_2$		$\mathbb{Z}_2$
Z_2^T	$\mathbb{Z}_2$	$\mathbb{Z}_2^2$	$\mathbb{Z}_2^4$	$\mathbb{Z}_2$
Z_2		$\mathbb{Z}_2^4$	$\mathbb{Z}_2^2$	$\mathbb{Z}_2^2$
$Z_2 \times Z_2^T$	$\mathbb{Z}_2^3$	$\mathbb{Z}_2^8$	$\mathbb{Z}_2^8$	$\mathbb{Z}_2^2$
Z_4		$\mathbb{Z}_2^4$	$\mathbb{Z}_2^2$	$\mathbb{Z}_2 \times \mathbb{Z}_4$
Z_6		$\mathbb{Z}_2^3 \times \mathbb{Z}_6$	$\mathbb{Z}_2^2$	$\mathbb{Z}_2 \times \mathbb{Z}_3 \times \mathbb{Z}_6$
$Z_2 \times Z_2$	$\mathbb{Z}_2^2$	$\mathbb{Z}_2^{10}$	$\mathbb{Z}_2^6$	$\mathbb{Z}_2^3$
$Z_4 \times Z_4$	$\mathbb{Z}_2^2$	$\mathbb{Z}_2^{10}$	$\mathbb{Z}_2^5 \times \mathbb{Z}_4$	$\mathbb{Z}_2 \times \mathbb{Z}_4^2$
$Z_6 \times Z_6$	$\mathbb{Z}_2^2$	$\mathbb{Z}_2^7 \times \mathbb{Z}_6^3$	$\mathbb{Z}_2^4 \times \mathbb{Z}_3 \times \mathbb{Z}_6^2$	$\mathbb{Z}_2 \times \mathbb{Z}_3^2 \times \mathbb{Z}_6^2$
$U(1)$		$\mathbb{Z}_2^3$		$\mathbb{Z} \times \mathbb{Z}_2 \times \mathbb{Z}_3$
$SU(2)$		$\mathbb{Z}_2^2$		$\mathbb{Z}_2$
$SO(3)$		$\mathbb{Z}_2^3$	$\mathbb{Z}_2^2$	$\mathbb{Z}_2$
Space group #187 $P\bar{6}m2$				
$\varnothing$		$\mathbb{Z}_2^3$	$\mathbb{Z}_2^2$	$\mathbb{Z}_2^4$
Z_2^T	$\mathbb{Z}_2$	$\mathbb{Z}_2^6$	$\mathbb{Z}_2^{10}$	$\mathbb{Z}_2^4$
Z_2		$\mathbb{Z}_2^9$	$\mathbb{Z}_2^7$	$\mathbb{Z}_2^6$
$Z_2 \times Z_2^T$	$\mathbb{Z}_2^3$	$\mathbb{Z}_2^{18}$	$\mathbb{Z}_2^{18}$	$\mathbb{Z}_2^6$
Z_4		$\mathbb{Z}_2^9$	$\mathbb{Z}_2^7$	$\mathbb{Z}_2^5 \times \mathbb{Z}_4$
Z_6		$\mathbb{Z}_2^9$	$\mathbb{Z}_2^7$	$\mathbb{Z}_2^4 \times \mathbb{Z}_3 \times \mathbb{Z}_6^2$
$Z_2 \times Z_2$	$\mathbb{Z}_2^2$	$\mathbb{Z}_2^{21}$	$\mathbb{Z}_2^{15}$	$\mathbb{Z}_2^8$
$Z_4 \times Z_4$	$\mathbb{Z}_2^2$	$\mathbb{Z}_2^{21}$	$\mathbb{Z}_2^{15}$	$\mathbb{Z}_2^6 \times \mathbb{Z}_4^2$
$Z_6 \times Z_6$	$\mathbb{Z}_2^2$	$\mathbb{Z}_2^{21}$	$\mathbb{Z}_2^{13} \times \mathbb{Z}_6^2$	$\mathbb{Z}_2^4 \times \mathbb{Z}_3^2 \times \mathbb{Z}_6^4$
$U(1)$		$\mathbb{Z}_2^7$	$\mathbb{Z}_2^2$	$\mathbb{Z} \times \mathbb{Z}_2^4 \times \mathbb{Z}_3 \times \mathbb{Z}_6$
$SU(2)$		$\mathbb{Z}_2^4$	$\mathbb{Z}_2^2$	$\mathbb{Z}_2^4$
$SO(3)$		$\mathbb{Z}_2^7$	$\mathbb{Z}_2^5$	$\mathbb{Z}_2^4$
Space group #188 $P\bar{6}c2$				
$\varnothing$		$\mathbb{Z}_2$		$\mathbb{Z}_2^2$
Z_2^T	$\mathbb{Z}_2$	$\mathbb{Z}_2^2$	$\mathbb{Z}_2^3$	$\mathbb{Z}_2^2$
Z_2		$\mathbb{Z}_2^4$	$\mathbb{Z}_2$	$\mathbb{Z}_2^4$
$Z_2 \times Z_2^T$	$\mathbb{Z}_2^3$	$\mathbb{Z}_2^8$	$\mathbb{Z}_2^6$	$\mathbb{Z}_2^4$
Z_4		$\mathbb{Z}_2^4$	$\mathbb{Z}_2$	$\mathbb{Z}_2^3 \times \mathbb{Z}_4$
Z_6		$\mathbb{Z}_2^4$	$\mathbb{Z}_2$	$\mathbb{Z}_2^2 \times \mathbb{Z}_3 \times \mathbb{Z}_6^2$
$Z_2 \times Z_2$	$\mathbb{Z}_2^2$	$\mathbb{Z}_2^{10}$	$\mathbb{Z}_2^4$	$\mathbb{Z}_2^6$
$Z_4 \times Z_4$	$\mathbb{Z}_2^2$	$\mathbb{Z}_2^{10}$	$\mathbb{Z}_2^4$	$\mathbb{Z}_2^4 \times \mathbb{Z}_4^2$
$Z_6 \times Z_6$	$\mathbb{Z}_2^2$	$\mathbb{Z}_2^{10}$	$\mathbb{Z}_2^2 \times \mathbb{Z}_6^2$	$\mathbb{Z}_2^2 \times \mathbb{Z}_3^2 \times \mathbb{Z}_6^4$
$U(1)$		$\mathbb{Z}_2^3$		$\mathbb{Z} \times \mathbb{Z}_2^2 \times \mathbb{Z}_3 \times \mathbb{Z}_6$
$SU(2)$		$\mathbb{Z}_2^2$		$\mathbb{Z}_2^2$
$SO(3)$		$\mathbb{Z}_2^3$	$\mathbb{Z}_2^2$	$\mathbb{Z}_2^2$
Space group #189 $P\bar{6}2m$				

TABLE II (Continued.)

G_0	$E_{3,\infty}^3$	$E_{2,\infty}^2$	$E_{1,\infty}^1$	$E_{0,\infty}^0$
$\varnothing$		$\mathbb{Z}_2^3$	$\mathbb{Z}_2^2$	$\mathbb{Z}_2^4 \times \mathbb{Z}_3$
Z_2^T	$\mathbb{Z}_2$	$\mathbb{Z}_2^6$	$\mathbb{Z}_2^{10}$	$\mathbb{Z}_2^4$
Z_2		$\mathbb{Z}_2^9$	$\mathbb{Z}_2^7$	$\mathbb{Z}_2^6 \times \mathbb{Z}_3$
$Z_2 \times Z_2^T$	$\mathbb{Z}_2^3$	$\mathbb{Z}_2^{18}$	$\mathbb{Z}_2^{18}$	$\mathbb{Z}_2^6$
Z_4		$\mathbb{Z}_2^9$	$\mathbb{Z}_2^7$	$\mathbb{Z}_2^5 \times \mathbb{Z}_3 \times \mathbb{Z}_4$
Z_6		$\mathbb{Z}_2^9$	$\mathbb{Z}_2^7$	$\mathbb{Z}_2^4 \times \mathbb{Z}_3 \times \mathbb{Z}_6^2$
$Z_2 \times Z_2$	$\mathbb{Z}_2^2$	$\mathbb{Z}_2^{21}$	$\mathbb{Z}_2^{15}$	$\mathbb{Z}_2^8 \times \mathbb{Z}_3$
$Z_4 \times Z_4$	$\mathbb{Z}_2^2$	$\mathbb{Z}_2^{21}$	$\mathbb{Z}_2^{15}$	$\mathbb{Z}_2^6 \times \mathbb{Z}_3 \times \mathbb{Z}_4^2$
$Z_6 \times Z_6$	$\mathbb{Z}_2^2$	$\mathbb{Z}_2^{21}$	$\mathbb{Z}_2^{14} \times \mathbb{Z}_6$	$\mathbb{Z}_2^4 \times \mathbb{Z}_3 \times \mathbb{Z}_6^4$
$U(1)$		$\mathbb{Z}_2^7$	$\mathbb{Z}_2^2$	$\mathbb{Z} \times \mathbb{Z}_2^4 \times \mathbb{Z}_3 \times \mathbb{Z}_6$
$SU(2)$		$\mathbb{Z}_2^4$	$\mathbb{Z}_2^2$	$\mathbb{Z}_2^4 \times \mathbb{Z}_3$
$SO(3)$		$\mathbb{Z}_2^7$	$\mathbb{Z}_2^5$	$\mathbb{Z}_2^4 \times \mathbb{Z}_3$
Space group #190 $P\bar{6}2c$				
$\varnothing$		$\mathbb{Z}_2$		$\mathbb{Z}_2^2 \times \mathbb{Z}_3$
Z_2^T	$\mathbb{Z}_2$	$\mathbb{Z}_2^2$	$\mathbb{Z}_2^3$	$\mathbb{Z}_2^2$
Z_2		$\mathbb{Z}_2^4$	$\mathbb{Z}_2$	$\mathbb{Z}_2^4 \times \mathbb{Z}_3$
$Z_2 \times Z_2^T$	$\mathbb{Z}_2^3$	$\mathbb{Z}_2^8$	$\mathbb{Z}_2^6$	$\mathbb{Z}_2^4$
Z_4		$\mathbb{Z}_2^4$	$\mathbb{Z}_2$	$\mathbb{Z}_2^3 \times \mathbb{Z}_3 \times \mathbb{Z}_4$
Z_6		$\mathbb{Z}_2^4$	$\mathbb{Z}_2$	$\mathbb{Z}_2^2 \times \mathbb{Z}_3 \times \mathbb{Z}_6^2$
$Z_2 \times Z_2$	$\mathbb{Z}_2^2$	$\mathbb{Z}_2^{10}$	$\mathbb{Z}_2^4$	$\mathbb{Z}_2^6 \times \mathbb{Z}_3$
$Z_4 \times Z_4$	$\mathbb{Z}_2^2$	$\mathbb{Z}_2^{10}$	$\mathbb{Z}_2^4$	$\mathbb{Z}_2^4 \times \mathbb{Z}_3 \times \mathbb{Z}_4^2$
$Z_6 \times Z_6$	$\mathbb{Z}_2^2$	$\mathbb{Z}_2^{10}$	$\mathbb{Z}_2^3 \times \mathbb{Z}_6$	$\mathbb{Z}_2^2 \times \mathbb{Z}_3 \times \mathbb{Z}_6^4$
$U(1)$		$\mathbb{Z}_2^3$		$\mathbb{Z} \times \mathbb{Z}_2^2 \times \mathbb{Z}_3 \times \mathbb{Z}_6$
$SU(2)$		$\mathbb{Z}_2^2$		$\mathbb{Z}_2^2 \times \mathbb{Z}_3$
$SO(3)$		$\mathbb{Z}_2^3$	$\mathbb{Z}_2^2$	$\mathbb{Z}_2^2 \times \mathbb{Z}_3$
Space group #191 $P6/mmm$				
$\varnothing$		$\mathbb{Z}_2^4$	$\mathbb{Z}_2^6$	$\mathbb{Z}_2^{12}$
Z_2^T	$\mathbb{Z}_2$	$\mathbb{Z}_2^8$	$\mathbb{Z}_2^{24}$	$\mathbb{Z}_2^{12}$
Z_2		$\mathbb{Z}_2^{12}$	$\mathbb{Z}_2^{18}$	$\mathbb{Z}_2^{16}$
$Z_2 \times Z_2^T$	$\mathbb{Z}_2^3$	$\mathbb{Z}_2^{24}$	$\mathbb{Z}_2^{42}$	$\mathbb{Z}_2^{16}$
Z_4		$\mathbb{Z}_2^{12}$	$\mathbb{Z}_2^{18}$	$\mathbb{Z}_2^{15} \times \mathbb{Z}_4$
Z_6		$\mathbb{Z}_2^{12}$	$\mathbb{Z}_2^{18}$	$\mathbb{Z}_2^{14} \times \mathbb{Z}_6^2$
$Z_2 \times Z_2$	$\mathbb{Z}_2^2$	$\mathbb{Z}_2^{28}$	$\mathbb{Z}_2^{36}$	$\mathbb{Z}_2^{20}$
$Z_4 \times Z_4$	$\mathbb{Z}_2^2$	$\mathbb{Z}_2^{28}$	$\mathbb{Z}_2^{36}$	$\mathbb{Z}_2^{18} \times \mathbb{Z}_4^2$
$Z_6 \times Z_6$	$\mathbb{Z}_2^2$	$\mathbb{Z}_2^{28}$	$\mathbb{Z}_2^{35} \times \mathbb{Z}_6$	$\mathbb{Z}_2^{16} \times \mathbb{Z}_6^4$
$U(1)$		$\mathbb{Z}_2^9$	$\mathbb{Z}_2^6$	$\mathbb{Z} \times \mathbb{Z}_2^{14} \times \mathbb{Z}_6$
$SU(2)$		$\mathbb{Z}_2^5$	$\mathbb{Z}_2^6$	$\mathbb{Z}_2^{12}$
$SO(3)$		$\mathbb{Z}_2^9$	$\mathbb{Z}_2^{12}$	$\mathbb{Z}_2^{12}$
Space group #192 $P6/mcc$				
$\varnothing$		$\mathbb{Z}_2$		$\mathbb{Z}_2^8$
Z_2^T	$\mathbb{Z}_2$	$\mathbb{Z}_2^2$	$\mathbb{Z}_2^{10}$	$\mathbb{Z}_2^8$
Z_2		$\mathbb{Z}_2^5$	$\mathbb{Z}_2^5$	$\mathbb{Z}_2^{12}$
$Z_2 \times Z_2^T$	$\mathbb{Z}_2^3$	$\mathbb{Z}_2^{10}$	$\mathbb{Z}_2^{20}$	$\mathbb{Z}_2^{12}$
Z_4		$\mathbb{Z}_2^5$	$\mathbb{Z}_2^5$	$\mathbb{Z}_2^{11} \times \mathbb{Z}_4$
Z_6		$\mathbb{Z}_2^5$	$\mathbb{Z}_2^5$	$\mathbb{Z}_2^{10} \times \mathbb{Z}_6^2$
$Z_2 \times Z_2$	$\mathbb{Z}_2^2$	$\mathbb{Z}_2^{13}$	$\mathbb{Z}_2^{15}$	$\mathbb{Z}_2^{16}$
$Z_4 \times Z_4$	$\mathbb{Z}_2^2$	$\mathbb{Z}_2^{13}$	$\mathbb{Z}_2^{15}$	$\mathbb{Z}_2^{14} \times \mathbb{Z}_4^2$
$Z_6 \times Z_6$	$\mathbb{Z}_2^2$	$\mathbb{Z}_2^{13}$	$\mathbb{Z}_2^{14} \times \mathbb{Z}_6$	$\mathbb{Z}_2^{12} \times \mathbb{Z}_6^4$

TABLE II (Continued.)

G_0	$E_{3,\infty}^3$	$E_{2,\infty}^2$	$E_{1,\infty}^1$	$E_{0,\infty}^0$
$U(1)$		$\mathbb{Z}_2^3$		$\mathbb{Z} \times \mathbb{Z}_2^{10} \times \mathbb{Z}_6$
$SU(2)$		$\mathbb{Z}_2^2$		$\mathbb{Z}_2^8$
$SO(3)$		$\mathbb{Z}_2^3$	$\mathbb{Z}_2^5$	$\mathbb{Z}_2^8$
Space group #193 $P6_3/mcm$				
$\varnothing$		$\mathbb{Z}_2^2$	$\mathbb{Z}_2$	$\mathbb{Z}_2^6$
Z_2^T	$\mathbb{Z}_2$	$\mathbb{Z}_2^4$	$\mathbb{Z}_2^{10}$	$\mathbb{Z}_2^6$
Z_2		$\mathbb{Z}_2^7$	$\mathbb{Z}_2^6$	$\mathbb{Z}_2^9$
$Z_2 \times Z_2^T$	$\mathbb{Z}_2^3$	$\mathbb{Z}_2^{14}$	$\mathbb{Z}_2^{19}$	$\mathbb{Z}_2^9$
Z_4		$\mathbb{Z}_2^7$	$\mathbb{Z}_2^6$	$\mathbb{Z}_2^8 \times \mathbb{Z}_4$
Z_6		$\mathbb{Z}_2^7$	$\mathbb{Z}_2^6$	$\mathbb{Z}_2^7 \times \mathbb{Z}_6^2$
$Z_2 \times Z_2$	$\mathbb{Z}_2^2$	$\mathbb{Z}_2^{17}$	$\mathbb{Z}_2^{15}$	$\mathbb{Z}_2^{12}$
$Z_4 \times Z_4$	$\mathbb{Z}_2^2$	$\mathbb{Z}_2^{17}$	$\mathbb{Z}_2^{15}$	$\mathbb{Z}_2^{10} \times \mathbb{Z}_4^2$
$Z_6 \times Z_6$	$\mathbb{Z}_2^2$	$\mathbb{Z}_2^{17}$	$\mathbb{Z}_2^{14} \times \mathbb{Z}_6$	$\mathbb{Z}_2^8 \times \mathbb{Z}_6^4$
$U(1)$		$\mathbb{Z}_2^5$	$\mathbb{Z}_2$	$\mathbb{Z} \times \mathbb{Z}_2^7 \times \mathbb{Z}_6$
$SU(2)$		$\mathbb{Z}_2^3$	$\mathbb{Z}_2$	$\mathbb{Z}_2^6$
$SO(3)$		$\mathbb{Z}_2^5$	$\mathbb{Z}_2^5$	$\mathbb{Z}_2^6$
Space group #194 $P6_3/mmc$				
$\varnothing$		$\mathbb{Z}_2^2$	$\mathbb{Z}_2$	$\mathbb{Z}_2^6$
Z_2^T	$\mathbb{Z}_2$	$\mathbb{Z}_2^4$	$\mathbb{Z}_2^{10}$	$\mathbb{Z}_2^6$
Z_2		$\mathbb{Z}_2^7$	$\mathbb{Z}_2^6$	$\mathbb{Z}_2^9$
$Z_2 \times Z_2^T$	$\mathbb{Z}_2^3$	$\mathbb{Z}_2^{14}$	$\mathbb{Z}_2^{19}$	$\mathbb{Z}_2^9$
Z_4		$\mathbb{Z}_2^7$	$\mathbb{Z}_2^6$	$\mathbb{Z}_2^8 \times \mathbb{Z}_4$
Z_6		$\mathbb{Z}_2^7$	$\mathbb{Z}_2^6$	$\mathbb{Z}_2^7 \times \mathbb{Z}_6^2$
$Z_2 \times Z_2$	$\mathbb{Z}_2^2$	$\mathbb{Z}_2^{17}$	$\mathbb{Z}_2^{15}$	$\mathbb{Z}_2^{12}$
$Z_4 \times Z_4$	$\mathbb{Z}_2^2$	$\mathbb{Z}_2^{17}$	$\mathbb{Z}_2^{15}$	$\mathbb{Z}_2^{10} \times \mathbb{Z}_4^2$
$Z_6 \times Z_6$	$\mathbb{Z}_2^2$	$\mathbb{Z}_2^{17}$	$\mathbb{Z}_2^{14} \times \mathbb{Z}_6$	$\mathbb{Z}_2^8 \times \mathbb{Z}_6^4$
$U(1)$		$\mathbb{Z}_2^5$	$\mathbb{Z}_2$	$\mathbb{Z} \times \mathbb{Z}_2^7 \times \mathbb{Z}_6$
$SU(2)$		$\mathbb{Z}_2^3$	$\mathbb{Z}_2$	$\mathbb{Z}_2^6$
$SO(3)$		$\mathbb{Z}_2^5$	$\mathbb{Z}_2^5$	$\mathbb{Z}_2^6$
Space group #195 $P23$				
$\varnothing$				$\mathbb{Z}_2^4 \times \mathbb{Z}_3$
Z_2^T	$\mathbb{Z}_2$		$\mathbb{Z}_2^8$	$\mathbb{Z}_2^4$
Z_2		$\mathbb{Z}_2$	$\mathbb{Z}_2^4$	$\mathbb{Z}_2^8 \times \mathbb{Z}_3$
$Z_2 \times Z_2^T$	$\mathbb{Z}_2^3$	$\mathbb{Z}_2^2$	$\mathbb{Z}_2^{16}$	$\mathbb{Z}_2^8$
Z_4		$\mathbb{Z}_2$	$\mathbb{Z}_2^4$	$\mathbb{Z}_2^7 \times \mathbb{Z}_3 \times \mathbb{Z}_4$
Z_6		$\mathbb{Z}_6$	$\mathbb{Z}_2^3 \times \mathbb{Z}_6$	$\mathbb{Z}_2^7 \times \mathbb{Z}_3 \times \mathbb{Z}_6$
$Z_2 \times Z_2$	$\mathbb{Z}_2^2$	$\mathbb{Z}_2^3$	$\mathbb{Z}_2^{12}$	$\mathbb{Z}_2^{12} \times \mathbb{Z}_3$
$Z_4 \times Z_4$	$\mathbb{Z}_4^2$	$\mathbb{Z}_2^3$	$\mathbb{Z}_2^{12}$	$\mathbb{Z}_2^{10} \times \mathbb{Z}_3 \times \mathbb{Z}_4^2$
$Z_6 \times Z_6$	$\mathbb{Z}_6^2$	$\mathbb{Z}_6^3$	$\mathbb{Z}_2^9 \times \mathbb{Z}_6^3$	$\mathbb{Z}_2^{10} \times \mathbb{Z}_3 \times \mathbb{Z}_6^2$
$U(1)$				$\mathbb{Z} \times \mathbb{Z}_2^7 \times \mathbb{Z}_3$
$SU(2)$				$\mathbb{Z}_2^4 \times \mathbb{Z}_3$
$SO(3)$			$\mathbb{Z}_2^4$	$\mathbb{Z}_2^4 \times \mathbb{Z}_3$
Space group #196 $F23$				
$\varnothing$				$\mathbb{Z}_3$
Z_2^T	$\mathbb{Z}_2$		$\mathbb{Z}_2^5$	
Z_2			$\mathbb{Z}_2^2$	$\mathbb{Z}_2^4 \times \mathbb{Z}_3$
$Z_2 \times Z_2^T$	$\mathbb{Z}_2^3$		$\mathbb{Z}_2^{10}$	$\mathbb{Z}_2^4$
Z_4			$\mathbb{Z}_2^2$	$\mathbb{Z}_2^2 \times \mathbb{Z}_3 \times \mathbb{Z}_4^2$
Z_6		$\mathbb{Z}_3$	$\mathbb{Z}_2 \times \mathbb{Z}_6$	$\mathbb{Z}_2^3 \times \mathbb{Z}_3 \times \mathbb{Z}_6$

TABLE II (Continued.)

G_0	$E_{3,\infty}^3$	$E_{2,\infty}^2$	$E_{1,\infty}^1$	$E_{0,\infty}^0$
$Z_2 \times Z_2$	$\mathbb{Z}_2^2$		$\mathbb{Z}_2^7$	$\mathbb{Z}_2^8 \times \mathbb{Z}_3$
$Z_4 \times Z_4$	$\mathbb{Z}_4^2$		$\mathbb{Z}_2^6 \times \mathbb{Z}_4$	$\mathbb{Z}_2^4 \times \mathbb{Z}_3 \times \mathbb{Z}_4^4$
$Z_6 \times Z_6$	$\mathbb{Z}_6^2$	$\mathbb{Z}_3^3$	$\mathbb{Z}_2^4 \times \mathbb{Z}_6^3$	$\mathbb{Z}_2^6 \times \mathbb{Z}_3 \times \mathbb{Z}_6^2$
$U(1)$				$\mathbb{Z} \times \mathbb{Z}_2^2 \times \mathbb{Z}_3 \times \mathbb{Z}_4$
$SU(2)$				$\mathbb{Z}_3$
$SO(3)$			$\mathbb{Z}_2^3$	$\mathbb{Z}_3$

Space group #197 $I23$

G_0	$E_{3,\infty}^3$	$E_{2,\infty}^2$	$E_{1,\infty}^1$	$E_{0,\infty}^0$
$\varnothing$				$\mathbb{Z}_2^2 \times \mathbb{Z}_3$
Z_2^T	$\mathbb{Z}_2$		$\mathbb{Z}_2^4$	$\mathbb{Z}_2^2$
Z_2		$\mathbb{Z}_2$	$\mathbb{Z}_2^2$	$\mathbb{Z}_2^4 \times \mathbb{Z}_3$
$Z_2 \times Z_2^T$	$\mathbb{Z}_2^3$	$\mathbb{Z}_2^2$	$\mathbb{Z}_2^8$	$\mathbb{Z}_2^4$
Z_4		$\mathbb{Z}_4$	$\mathbb{Z}_2^2$	$\mathbb{Z}_2^3 \times \mathbb{Z}_3 \times \mathbb{Z}_4$
Z_6		$\mathbb{Z}_6$	$\mathbb{Z}_2 \times \mathbb{Z}_6$	$\mathbb{Z}_2^3 \times \mathbb{Z}_3 \times \mathbb{Z}_6$
$Z_2 \times Z_2$	$\mathbb{Z}_2^2$	$\mathbb{Z}_2^3$	$\mathbb{Z}_2^6$	$\mathbb{Z}_2^6 \times \mathbb{Z}_3$
$Z_4 \times Z_4$	$\mathbb{Z}_4^2$	$\mathbb{Z}_4^3$	$\mathbb{Z}_2^5 \times \mathbb{Z}_4$	$\mathbb{Z}_2^4 \times \mathbb{Z}_3 \times \mathbb{Z}_4^2$
$Z_6 \times Z_6$	$\mathbb{Z}_6^2$	$\mathbb{Z}_6^3$	$\mathbb{Z}_2^3 \times \mathbb{Z}_6^3$	$\mathbb{Z}_2^4 \times \mathbb{Z}_3 \times \mathbb{Z}_6^2$
$U(1)$				$\mathbb{Z} \times \mathbb{Z}_2^3 \times \mathbb{Z}_3$
$SU(2)$				$\mathbb{Z}_2^2 \times \mathbb{Z}_3$
$SO(3)$			$\mathbb{Z}_2^2$	$\mathbb{Z}_2^2 \times \mathbb{Z}_3$

Space group #198 $P2_13$

G_0	$E_{3,\infty}^3$	$E_{2,\infty}^2$	$E_{1,\infty}^1$	$E_{0,\infty}^0$
$\varnothing$				$\mathbb{Z}_3$
Z_2^T	$\mathbb{Z}_2$			
Z_2				$\mathbb{Z}_2 \times \mathbb{Z}_3$
$Z_2 \times Z_2^T$	$\mathbb{Z}_2^3$			$\mathbb{Z}_2$
Z_4				$\mathbb{Z}_3 \times \mathbb{Z}_4$
Z_6		$\mathbb{Z}_3$	$\mathbb{Z}_3$	$\mathbb{Z}_3 \times \mathbb{Z}_6$
$Z_2 \times Z_2$	$\mathbb{Z}_2^2$			$\mathbb{Z}_2^2 \times \mathbb{Z}_3$
$Z_4 \times Z_4$	$\mathbb{Z}_4^2$			$\mathbb{Z}_3 \times \mathbb{Z}_4^2$
$Z_6 \times Z_6$	$\mathbb{Z}_6^2$	$\mathbb{Z}_3^3$	$\mathbb{Z}_3^3$	$\mathbb{Z}_3 \times \mathbb{Z}_6^2$
$U(1)$				$\mathbb{Z} \times \mathbb{Z}_3$
$SU(2)$				$\mathbb{Z}_3$
$SO(3)$				$\mathbb{Z}_3$

Space group #199 $I2_13$

G_0	$E_{3,\infty}^3$	$E_{2,\infty}^2$	$E_{1,\infty}^1$	$E_{0,\infty}^0$
$\varnothing$				$\mathbb{Z}_2 \times \mathbb{Z}_3$
Z_2^T	$\mathbb{Z}_2$		$\mathbb{Z}_2^2$	$\mathbb{Z}_2$
Z_2		$\mathbb{Z}_2$	$\mathbb{Z}_2$	$\mathbb{Z}_2^2 \times \mathbb{Z}_3$
$Z_2 \times Z_2^T$	$\mathbb{Z}_2^3$	$\mathbb{Z}_2^2$	$\mathbb{Z}_2^4$	$\mathbb{Z}_2^2$
Z_4		$\mathbb{Z}_2$	$\mathbb{Z}_2$	$\mathbb{Z}_2 \times \mathbb{Z}_3 \times \mathbb{Z}_4$
Z_6		$\mathbb{Z}_6$	$\mathbb{Z}_6$	$\mathbb{Z}_2 \times \mathbb{Z}_3 \times \mathbb{Z}_6$
$Z_2 \times Z_2$	$\mathbb{Z}_2^2$	$\mathbb{Z}_2^3$	$\mathbb{Z}_2^3$	$\mathbb{Z}_2^3 \times \mathbb{Z}_3$
$Z_4 \times Z_4$	$\mathbb{Z}_4^2$	$\mathbb{Z}_2^3$	$\mathbb{Z}_2^3$	$\mathbb{Z}_2 \times \mathbb{Z}_3 \times \mathbb{Z}_4^2$
$Z_6 \times Z_6$	$\mathbb{Z}_6^2$	$\mathbb{Z}_6^3$	$\mathbb{Z}_6^3$	$\mathbb{Z}_2 \times \mathbb{Z}_3 \times \mathbb{Z}_6^2$
$U(1)$				$\mathbb{Z} \times \mathbb{Z}_2 \times \mathbb{Z}_3$
$SU(2)$				$\mathbb{Z}_2 \times \mathbb{Z}_3$
$SO(3)$			$\mathbb{Z}_2$	$\mathbb{Z}_2 \times \mathbb{Z}_3$

Space group #200 $Pm\bar{3}$

G_0	$E_{3,\infty}^3$	$E_{2,\infty}^2$	$E_{1,\infty}^1$	$E_{0,\infty}^0$
$\varnothing$		$\mathbb{Z}_2^2$	$\mathbb{Z}_2^4$	$\mathbb{Z}_2^8 \times \mathbb{Z}_3$
Z_2^T	$\mathbb{Z}_2$	$\mathbb{Z}_2^4$	$\mathbb{Z}_2^{16}$	$\mathbb{Z}_2^8$

TABLE II (Continued.)

G_0	$E_{3,\infty}^3$	$E_{2,\infty}^2$	$E_{1,\infty}^1$	$E_{0,\infty}^0$
Z_2		$\mathbb{Z}_2^6$	$\mathbb{Z}_2^{12}$	$\mathbb{Z}_2^{12} \times \mathbb{Z}_3$
$Z_2 \times Z_2^T$	$\mathbb{Z}_2^3$	$\mathbb{Z}_2^{12}$	$\mathbb{Z}_2^{28}$	$\mathbb{Z}_2^{12}$
Z_4		$\mathbb{Z}_2^6$	$\mathbb{Z}_2^{12}$	$\mathbb{Z}_2^{11} \times \mathbb{Z}_3 \times \mathbb{Z}_4$
Z_6		$\mathbb{Z}_2^6$	$\mathbb{Z}_2^{12}$	$\mathbb{Z}_2^{11} \times \mathbb{Z}_3 \times \mathbb{Z}_6$
$Z_2 \times Z_2$	$\mathbb{Z}_2^2$	$\mathbb{Z}_2^{14}$	$\mathbb{Z}_2^{24}$	$\mathbb{Z}_2^{16} \times \mathbb{Z}_3$
$Z_4 \times Z_4$	$\mathbb{Z}_2^2$	$\mathbb{Z}_2^{14}$	$\mathbb{Z}_2^{24}$	$\mathbb{Z}_2^{14} \times \mathbb{Z}_3 \times \mathbb{Z}_4^2$
$Z_6 \times Z_6$	$\mathbb{Z}_2^2$	$\mathbb{Z}_2^{14}$	$\mathbb{Z}_2^{24}$	$\mathbb{Z}_2^{14} \times \mathbb{Z}_3 \times \mathbb{Z}_6^2$
$U(1)$		$\mathbb{Z}_2^5$	$\mathbb{Z}_2^4$	$\mathbb{Z} \times \mathbb{Z}_2^{11} \times \mathbb{Z}_3$
$SU(2)$		$\mathbb{Z}_2^3$	$\mathbb{Z}_2^4$	$\mathbb{Z}_2^8 \times \mathbb{Z}_3$
$SO(3)$		$\mathbb{Z}_2^5$	$\mathbb{Z}_2^8$	$\mathbb{Z}_2^8 \times \mathbb{Z}_3$

Space group #201 $Pn\bar{3}$

G_0	$E_{3,\infty}^3$	$E_{2,\infty}^2$	$E_{1,\infty}^1$	$E_{0,\infty}^0$
$\varnothing$				$\mathbb{Z}_2^4 \times \mathbb{Z}_3$
Z_2^T	$\mathbb{Z}_2$		$\mathbb{Z}_2^6$	$\mathbb{Z}_2^4$
Z_2		$\mathbb{Z}_2^2$	$\mathbb{Z}_2^2$	$\mathbb{Z}_2^8 \times \mathbb{Z}_3$
$Z_2 \times Z_2^T$	$\mathbb{Z}_2^3$	$\mathbb{Z}_2^4$	$\mathbb{Z}_2^{12}$	$\mathbb{Z}_2^8$
Z_4		$\mathbb{Z}_2^2$	$\mathbb{Z}_2^2$	$\mathbb{Z}_2^7 \times \mathbb{Z}_3 \times \mathbb{Z}_4$
Z_6		$\mathbb{Z}_2^2$	$\mathbb{Z}_2^2$	$\mathbb{Z}_2^7 \times \mathbb{Z}_3 \times \mathbb{Z}_6$
$Z_2 \times Z_2$	$\mathbb{Z}_2^2$	$\mathbb{Z}_2^6$	$\mathbb{Z}_2^8$	$\mathbb{Z}_2^{12} \times \mathbb{Z}_3$
$Z_4 \times Z_4$	$\mathbb{Z}_2^2$	$\mathbb{Z}_2^6$	$\mathbb{Z}_2^8$	$\mathbb{Z}_2^{10} \times \mathbb{Z}_3 \times \mathbb{Z}_4^2$
$Z_6 \times Z_6$	$\mathbb{Z}_2^2$	$\mathbb{Z}_2^6$	$\mathbb{Z}_2^8$	$\mathbb{Z}_2^{10} \times \mathbb{Z}_3 \times \mathbb{Z}_6^2$
$U(1)$		$\mathbb{Z}_2$		$\mathbb{Z} \times \mathbb{Z}_2^7 \times \mathbb{Z}_3$
$SU(2)$		$\mathbb{Z}_2$		$\mathbb{Z}_2^4 \times \mathbb{Z}_3$
$SO(3)$		$\mathbb{Z}_2$	$\mathbb{Z}_2^4$	$\mathbb{Z}_2^4 \times \mathbb{Z}_3$

Space group #202 $Fm\bar{3}$

G_0	$E_{3,\infty}^3$	$E_{2,\infty}^2$	$E_{1,\infty}^1$	$E_{0,\infty}^0$
$\varnothing$		$\mathbb{Z}_2$	$\mathbb{Z}_2$	$\mathbb{Z}_2^4 \times \mathbb{Z}_3$
Z_2^T	$\mathbb{Z}_2$	$\mathbb{Z}_2^2$	$\mathbb{Z}_2^8$	$\mathbb{Z}_2^4$
Z_2		$\mathbb{Z}_2^3$	$\mathbb{Z}_2^5$	$\mathbb{Z}_2^8 \times \mathbb{Z}_3$
$Z_2 \times Z_2^T$	$\mathbb{Z}_2^3$	$\mathbb{Z}_2^6$	$\mathbb{Z}_2^{15}$	$\mathbb{Z}_2^8$
Z_4		$\mathbb{Z}_2^3$	$\mathbb{Z}_2^5$	$\mathbb{Z}_2^7 \times \mathbb{Z}_3 \times \mathbb{Z}_4$
Z_6		$\mathbb{Z}_2^3$	$\mathbb{Z}_2^5$	$\mathbb{Z}_2^7 \times \mathbb{Z}_3 \times \mathbb{Z}_6$
$Z_2 \times Z_2$	$\mathbb{Z}_2^2$	$\mathbb{Z}_2^7$	$\mathbb{Z}_2^{12}$	$\mathbb{Z}_2^{12} \times \mathbb{Z}_3$
$Z_4 \times Z_4$	$\mathbb{Z}_2^2$	$\mathbb{Z}_2^7$	$\mathbb{Z}_2^{12}$	$\mathbb{Z}_2^{10} \times \mathbb{Z}_3 \times \mathbb{Z}_4^2$
$Z_6 \times Z_6$	$\mathbb{Z}_2^2$	$\mathbb{Z}_2^7$	$\mathbb{Z}_2^{12}$	$\mathbb{Z}_2^{10} \times \mathbb{Z}_3 \times \mathbb{Z}_6^2$
$U(1)$		$\mathbb{Z}_2^3$	$\mathbb{Z}_2$	$\mathbb{Z} \times \mathbb{Z}_2^7 \times \mathbb{Z}_3$
$SU(2)$		$\mathbb{Z}_2^2$	$\mathbb{Z}_2$	$\mathbb{Z}_2^4 \times \mathbb{Z}_3$
$SO(3)$		$\mathbb{Z}_2^3$	$\mathbb{Z}_2^4$	$\mathbb{Z}_2^4 \times \mathbb{Z}_3$

Space group #203 $Fd\bar{3}$

G_0	$E_{3,\infty}^3$	$E_{2,\infty}^2$	$E_{1,\infty}^1$	$E_{0,\infty}^0$
$\varnothing$				$\mathbb{Z}_2^2 \times \mathbb{Z}_3$
Z_2^T	$\mathbb{Z}_2$		$\mathbb{Z}_2^4$	$\mathbb{Z}_2^2$
Z_2		$\mathbb{Z}_2$	$\mathbb{Z}_2$	$\mathbb{Z}_2^6 \times \mathbb{Z}_3$
$Z_2 \times Z_2^T$	$\mathbb{Z}_2^3$	$\mathbb{Z}_2^2$	$\mathbb{Z}_2^8$	$\mathbb{Z}_2^6$
Z_4		$\mathbb{Z}_2$	$\mathbb{Z}_2$	$\mathbb{Z}_2^5 \times \mathbb{Z}_3 \times \mathbb{Z}_4$
Z_6		$\mathbb{Z}_2$	$\mathbb{Z}_2$	$\mathbb{Z}_2^5 \times \mathbb{Z}_3 \times \mathbb{Z}_6$
$Z_2 \times Z_2$	$\mathbb{Z}_2^2$	$\mathbb{Z}_2^3$	$\mathbb{Z}_2^5$	$\mathbb{Z}_2^{10} \times \mathbb{Z}_3$
$Z_4 \times Z_4$	$\mathbb{Z}_2^2$	$\mathbb{Z}_2^3$	$\mathbb{Z}_2^5$	$\mathbb{Z}_2^8 \times \mathbb{Z}_3 \times \mathbb{Z}_4^2$
$Z_6 \times Z_6$	$\mathbb{Z}_2^2$	$\mathbb{Z}_2^3$	$\mathbb{Z}_2^5$	$\mathbb{Z}_2^8 \times \mathbb{Z}_3 \times \mathbb{Z}_6^2$
$U(1)$		$\mathbb{Z}_2$		$\mathbb{Z} \times \mathbb{Z}_2^5 \times \mathbb{Z}_3$
$SU(2)$		$\mathbb{Z}_2$		$\mathbb{Z}_2^2 \times \mathbb{Z}_3$
$SO(3)$		$\mathbb{Z}_2$	$\mathbb{Z}_2^3$	$\mathbb{Z}_2^2 \times \mathbb{Z}_3$

TABLE II (Continued.)

G_0	$E^3_{3,\infty}$	$E^2_{2,\infty}$	$E^1_{1,\infty}$	$E^0_{0,\infty}$
**Space group #204 $Im\bar{3}$**				
$\varnothing$		$\mathbb{Z}_2$	$\mathbb{Z}_2^2$	$\mathbb{Z}_2^5 \times \mathbb{Z}_3$
Z_2^T	$\mathbb{Z}_2$	$\mathbb{Z}_2^2$	$\mathbb{Z}_2^9$	$\mathbb{Z}_2^5$
Z_2		$\mathbb{Z}_2^4$	$\mathbb{Z}_2^6$	$\mathbb{Z}_2^8 \times \mathbb{Z}_3$
$Z_2 \times Z_2^T$	$\mathbb{Z}_2^3$	$\mathbb{Z}_2^8$	$\mathbb{Z}_2^{16}$	$\mathbb{Z}_2^8$
Z_4		$\mathbb{Z}_2^3 \times \mathbb{Z}_4$	$\mathbb{Z}_2^6$	$\mathbb{Z}_2^7 \times \mathbb{Z}_3 \times \mathbb{Z}_4$
Z_6		$\mathbb{Z}_2^4$	$\mathbb{Z}_2^6$	$\mathbb{Z}_2^7 \times \mathbb{Z}_3 \times \mathbb{Z}_6$
$Z_2 \times Z_2$	$\mathbb{Z}_2^2$	$\mathbb{Z}_2^{10}$	$\mathbb{Z}_2^{13}$	$\mathbb{Z}_2^{11} \times \mathbb{Z}_3$
$Z_4 \times Z_4$	$\mathbb{Z}_2^2$	$\mathbb{Z}_2^7 \times \mathbb{Z}_4^3$	$\mathbb{Z}_2^{12} \times \mathbb{Z}_4$	$\mathbb{Z}_2^9 \times \mathbb{Z}_3 \times \mathbb{Z}_4^2$
$Z_6 \times Z_6$	$\mathbb{Z}_2^2$	$\mathbb{Z}_2^{10}$	$\mathbb{Z}_2^{13}$	$\mathbb{Z}_2^9 \times \mathbb{Z}_3 \times \mathbb{Z}_6^2$
$U(1)$		$\mathbb{Z}_2^3$	$\mathbb{Z}_2^2$	$\mathbb{Z} \times \mathbb{Z}_2^7 \times \mathbb{Z}_3$
$SU(2)$		$\mathbb{Z}_2^2$	$\mathbb{Z}_2^2$	$\mathbb{Z}_2^5 \times \mathbb{Z}_3$
$SO(3)$		$\mathbb{Z}_2^3$	$\mathbb{Z}_2^5$	$\mathbb{Z}_2^5 \times \mathbb{Z}_3$
**Space group #205 $Pa\bar{3}$**				
$\varnothing$				$\mathbb{Z}_2^2 \times \mathbb{Z}_3$
Z_2^T	$\mathbb{Z}_2$		$\mathbb{Z}_2$	$\mathbb{Z}_2^2$
Z_2		$\mathbb{Z}_2$		$\mathbb{Z}_2^4 \times \mathbb{Z}_3$
$Z_2 \times Z_2^T$	$\mathbb{Z}_2^3$	$\mathbb{Z}_2^2$	$\mathbb{Z}_2^2$	$\mathbb{Z}_2^4$
Z_4		$\mathbb{Z}_2$		$\mathbb{Z}_2^3 \times \mathbb{Z}_3 \times \mathbb{Z}_4$
Z_6		$\mathbb{Z}_2$		$\mathbb{Z}_2^3 \times \mathbb{Z}_3 \times \mathbb{Z}_6$
$Z_2 \times Z_2$	$\mathbb{Z}_2^2$	$\mathbb{Z}_2^3$	$\mathbb{Z}_2$	$\mathbb{Z}_2^6 \times \mathbb{Z}_3$
$Z_4 \times Z_4$	$\mathbb{Z}_2^2$	$\mathbb{Z}_2^3$	$\mathbb{Z}_2$	$\mathbb{Z}_2^4 \times \mathbb{Z}_3 \times \mathbb{Z}_4^2$
$Z_6 \times Z_6$	$\mathbb{Z}_2^2$	$\mathbb{Z}_2^3$	$\mathbb{Z}_2$	$\mathbb{Z}_2^4 \times \mathbb{Z}_3 \times \mathbb{Z}_6^2$
$U(1)$		$\mathbb{Z}_2$		$\mathbb{Z} \times \mathbb{Z}_2^3 \times \mathbb{Z}_3$
$SU(2)$		$\mathbb{Z}_2$		$\mathbb{Z}_2^2 \times \mathbb{Z}_3$
$SO(3)$		$\mathbb{Z}_2$	$\mathbb{Z}_2$	$\mathbb{Z}_2^2 \times \mathbb{Z}_3$
**Space group #206 $Ia\bar{3}$**				
$\varnothing$				$\mathbb{Z}_2^3 \times \mathbb{Z}_3$
Z_2^T	$\mathbb{Z}_2$		$\mathbb{Z}_2^4$	$\mathbb{Z}_2^3$
Z_2		$\mathbb{Z}_2^2$	$\mathbb{Z}_2$	$\mathbb{Z}_2^6 \times \mathbb{Z}_3$
$Z_2 \times Z_2^T$	$\mathbb{Z}_2^3$	$\mathbb{Z}_2^4$	$\mathbb{Z}_2^8$	$\mathbb{Z}_2^6$
Z_4		$\mathbb{Z}_2^2$	$\mathbb{Z}_2$	$\mathbb{Z}_2^5 \times \mathbb{Z}_3 \times \mathbb{Z}_4$
Z_6		$\mathbb{Z}_2^2$	$\mathbb{Z}_2$	$\mathbb{Z}_2^5 \times \mathbb{Z}_3 \times \mathbb{Z}_6$
$Z_2 \times Z_2$	$\mathbb{Z}_2^2$	$\mathbb{Z}_2^6$	$\mathbb{Z}_2^5$	$\mathbb{Z}_2^9 \times \mathbb{Z}_3$
$Z_4 \times Z_4$	$\mathbb{Z}_2^2$	$\mathbb{Z}_2^6$	$\mathbb{Z}_2^5$	$\mathbb{Z}_2^7 \times \mathbb{Z}_3 \times \mathbb{Z}_4^2$
$Z_6 \times Z_6$	$\mathbb{Z}_2^2$	$\mathbb{Z}_2^6$	$\mathbb{Z}_2^5$	$\mathbb{Z}_2^7 \times \mathbb{Z}_3 \times \mathbb{Z}_6^2$
$U(1)$		$\mathbb{Z}_2$		$\mathbb{Z} \times \mathbb{Z}_2^5 \times \mathbb{Z}_3$
$SU(2)$		$\mathbb{Z}_2$		$\mathbb{Z}_2^3 \times \mathbb{Z}_3$
$SO(3)$		$\mathbb{Z}_2$	$\mathbb{Z}_2^3$	$\mathbb{Z}_2^3 \times \mathbb{Z}_3$
**Space group #207 $P432$**				
$\varnothing$				$\mathbb{Z}_2^6$
Z_2^T	$\mathbb{Z}_2$		$\mathbb{Z}_2^{10}$	$\mathbb{Z}_2^6$
Z_2		$\mathbb{Z}_2^2$	$\mathbb{Z}_2^5$	$\mathbb{Z}_2^{10}$
$Z_2 \times Z_2^T$	$\mathbb{Z}_2^3$	$\mathbb{Z}_2^4$	$\mathbb{Z}_2^{20}$	$\mathbb{Z}_2^{10}$
Z_4		$\mathbb{Z}_2^2$	$\mathbb{Z}_2^3 \times \mathbb{Z}_4^2$	$\mathbb{Z}_2^8 \times \mathbb{Z}_4^2$
Z_6		$\mathbb{Z}_2^2$	$\mathbb{Z}_2^4 \times \mathbb{Z}_6$	$\mathbb{Z}_2^9 \times \mathbb{Z}_6$
$Z_2 \times Z_2$	$\mathbb{Z}_2^2$	$\mathbb{Z}_2^6$	$\mathbb{Z}_2^{15}$	$\mathbb{Z}_2^{14}$
$Z_4 \times Z_4$	$\mathbb{Z}_4^2$	$\mathbb{Z}_2^6$	$\mathbb{Z}_2^{10} \times \mathbb{Z}_4^5$	$\mathbb{Z}_2^{10} \times \mathbb{Z}_4^4$
$Z_6 \times Z_6$	$\mathbb{Z}_6^2$	$\mathbb{Z}_2^6$	$\mathbb{Z}_2^{13} \times \mathbb{Z}_6^2$	$\mathbb{Z}_2^{12} \times \mathbb{Z}_6^2$

TABLE II (Continued.)

G_0	$E^3_{3,\infty}$	$E^2_{2,\infty}$	$E^1_{1,\infty}$	$E^0_{0,\infty}$
$U(1)$				$\mathbb{Z} \times \mathbb{Z}_2^8 \times \mathbb{Z}_4$
$SU(2)$				$\mathbb{Z}_2^6$
$SO(3)$			$\mathbb{Z}_2^5$	$\mathbb{Z}_2^6$
**Space group #208 $P4_232$**				
$\varnothing$				$\mathbb{Z}_2^6$
Z_2^T	$\mathbb{Z}_2$		$\mathbb{Z}_2^{10}$	$\mathbb{Z}_2^6$
Z_2		$\mathbb{Z}_2^2$	$\mathbb{Z}_2^5$	$\mathbb{Z}_2^{10}$
$Z_2 \times Z_2^T$	$\mathbb{Z}_2^3$	$\mathbb{Z}_2^4$	$\mathbb{Z}_2^{20}$	$\mathbb{Z}_2^{10}$
Z_4		$\mathbb{Z}_2^2$	$\mathbb{Z}_2^5$	$\mathbb{Z}_2^9 \times \mathbb{Z}_4$
Z_6		$\mathbb{Z}_2^2$	$\mathbb{Z}_2^4 \times \mathbb{Z}_6$	$\mathbb{Z}_2^9 \times \mathbb{Z}_6$
$Z_2 \times Z_2$	$\mathbb{Z}_2^2$	$\mathbb{Z}_2^6$	$\mathbb{Z}_2^{15}$	$\mathbb{Z}_2^{14}$
$Z_4 \times Z_4$	$\mathbb{Z}_4^2$	$\mathbb{Z}_2^6$	$\mathbb{Z}_2^{15}$	$\mathbb{Z}_2^{12} \times \mathbb{Z}_4^2$
$Z_6 \times Z_6$	$\mathbb{Z}_6^2$	$\mathbb{Z}_2^6$	$\mathbb{Z}_2^{13} \times \mathbb{Z}_6^2$	$\mathbb{Z}_2^{12} \times \mathbb{Z}_6^2$
$U(1)$				$\mathbb{Z} \times \mathbb{Z}_2^9$
$SU(2)$				$\mathbb{Z}_2^6$
$SO(3)$			$\mathbb{Z}_2^5$	$\mathbb{Z}_2^6$
**Space group #209 $F432$**				
$\varnothing$				$\mathbb{Z}_2^4$
Z_2^T	$\mathbb{Z}_2$		$\mathbb{Z}_2^8$	$\mathbb{Z}_2^4$
Z_2		$\mathbb{Z}_2$	$\mathbb{Z}_2^4$	$\mathbb{Z}_2^8$
$Z_2 \times Z_2^T$	$\mathbb{Z}_2^3$	$\mathbb{Z}_2^2$	$\mathbb{Z}_2^{16}$	$\mathbb{Z}_2^8$
Z_4		$\mathbb{Z}_2$	$\mathbb{Z}_2^3 \times \mathbb{Z}_4$	$\mathbb{Z}_2^7 \times \mathbb{Z}_4$
Z_6		$\mathbb{Z}_2$	$\mathbb{Z}_2^3 \times \mathbb{Z}_6$	$\mathbb{Z}_2^7 \times \mathbb{Z}_6$
$Z_2 \times Z_2$	$\mathbb{Z}_2^2$	$\mathbb{Z}_2^3$	$\mathbb{Z}_2^{12}$	$\mathbb{Z}_2^{12}$
$Z_4 \times Z_4$	$\mathbb{Z}_4^2$	$\mathbb{Z}_2^3$	$\mathbb{Z}_2^{10} \times \mathbb{Z}_4^2$	$\mathbb{Z}_2^{10} \times \mathbb{Z}_4^2$
$Z_6 \times Z_6$	$\mathbb{Z}_6^2$	$\mathbb{Z}_2^3$	$\mathbb{Z}_2^{10} \times \mathbb{Z}_6^2$	$\mathbb{Z}_2^{10} \times \mathbb{Z}_6^2$
$U(1)$				$\mathbb{Z} \times \mathbb{Z}_2^7$
$SU(2)$				$\mathbb{Z}_2^4$
$SO(3)$			$\mathbb{Z}_2^4$	$\mathbb{Z}_2^4$
**Space group #210 $F4_132$**				
$\varnothing$				$\mathbb{Z}_2$
Z_2^T	$\mathbb{Z}_2$		$\mathbb{Z}_2^5$	$\mathbb{Z}_2$
Z_2		$\mathbb{Z}_2$	$\mathbb{Z}_2^2$	$\mathbb{Z}_2^4$
$Z_2 \times Z_2^T$	$\mathbb{Z}_2^3$	$\mathbb{Z}_2^2$	$\mathbb{Z}_2^{10}$	$\mathbb{Z}_2^4$
Z_4		$\mathbb{Z}_2$	$\mathbb{Z}_2^2$	$\mathbb{Z}_2^3 \times \mathbb{Z}_4$
Z_6		$\mathbb{Z}_2$	$\mathbb{Z}_2 \times \mathbb{Z}_6$	$\mathbb{Z}_2^3 \times \mathbb{Z}_6$
$Z_2 \times Z_2$	$\mathbb{Z}_2^2$	$\mathbb{Z}_2^3$	$\mathbb{Z}_2^7$	$\mathbb{Z}_2^7$
$Z_4 \times Z_4$	$\mathbb{Z}_4^2$	$\mathbb{Z}_2^3$	$\mathbb{Z}_2^7$	$\mathbb{Z}_2^5 \times \mathbb{Z}_4^2$
$Z_6 \times Z_6$	$\mathbb{Z}_6^2$	$\mathbb{Z}_2^3$	$\mathbb{Z}_2^5 \times \mathbb{Z}_6^2$	$\mathbb{Z}_2^5 \times \mathbb{Z}_6^2$
$U(1)$				$\mathbb{Z} \times \mathbb{Z}_2^3$
$SU(2)$				$\mathbb{Z}_2$
$SO(3)$			$\mathbb{Z}_2^3$	$\mathbb{Z}_2$
**Space group #211 $I432$**				
$\varnothing$				$\mathbb{Z}_2^5$
Z_2^T	$\mathbb{Z}_2$		$\mathbb{Z}_2^8$	$\mathbb{Z}_2^5$
Z_2		$\mathbb{Z}_2^2$	$\mathbb{Z}_2^4$	$\mathbb{Z}_2^8$
$Z_2 \times Z_2^T$	$\mathbb{Z}_2^3$	$\mathbb{Z}_2^4$	$\mathbb{Z}_2^{16}$	$\mathbb{Z}_2^8$
Z_4		$\mathbb{Z}_2^2$	$\mathbb{Z}_2^3 \times \mathbb{Z}_4$	$\mathbb{Z}_2^7 \times \mathbb{Z}_4$
Z_6		$\mathbb{Z}_2^2$	$\mathbb{Z}_2^3 \times \mathbb{Z}_6$	$\mathbb{Z}_2^7 \times \mathbb{Z}_6$

TABLE II (Continued.)

G_0	$E_{3,\infty}^3$	$E_{2,\infty}^2$	$E_{1,\infty}^1$	$E_{0,\infty}^0$
$Z_2 \times Z_2$	$\mathbb{Z}_2^2$	$\mathbb{Z}_2^6$	$\mathbb{Z}_2^{12}$	$\mathbb{Z}_2^{11}$
$Z_4 \times Z_4$	$\mathbb{Z}_4^2$	$\mathbb{Z}_2^6$	$\mathbb{Z}_2^{10} \times \mathbb{Z}_4^2$	$\mathbb{Z}_2^9 \times \mathbb{Z}_4^2$
$Z_6 \times Z_6$	$\mathbb{Z}_6^2$	$\mathbb{Z}_2^6$	$\mathbb{Z}_2^{10} \times \mathbb{Z}_6^2$	$\mathbb{Z}_2^9 \times \mathbb{Z}_6^2$
$U(1)$				$\mathbb{Z} \times \mathbb{Z}_2^7$
$SU(2)$				$\mathbb{Z}_2^5$
$SO(3)$			$\mathbb{Z}_2^4$	$\mathbb{Z}_2^5$

Space group #212 $P4_332$

G_0	$E_{3,\infty}^3$	$E_{2,\infty}^2$	$E_{1,\infty}^1$	$E_{0,\infty}^0$
$\varnothing$				$\mathbb{Z}_2$
Z_2^T	$\mathbb{Z}_2$		$\mathbb{Z}_2^2$	$\mathbb{Z}_2$
Z_2		$\mathbb{Z}_2$	$\mathbb{Z}_2$	$\mathbb{Z}_2^2$
$Z_2 \times Z_2^T$	$\mathbb{Z}_2^3$	$\mathbb{Z}_2^2$	$\mathbb{Z}_2^4$	$\mathbb{Z}_2^2$
Z_4		$\mathbb{Z}_2$	$\mathbb{Z}_2$	$\mathbb{Z}_2 \times \mathbb{Z}_4$
Z_6		$\mathbb{Z}_2$	$\mathbb{Z}_6$	$\mathbb{Z}_2 \times \mathbb{Z}_6$
$Z_2 \times Z_2$	$\mathbb{Z}_2^2$	$\mathbb{Z}_2^3$	$\mathbb{Z}_2^3$	$\mathbb{Z}_2^3$
$Z_4 \times Z_4$	$\mathbb{Z}_4^2$	$\mathbb{Z}_2^3$	$\mathbb{Z}_2^3$	$\mathbb{Z}_2 \times \mathbb{Z}_4^2$
$Z_6 \times Z_6$	$\mathbb{Z}_6^2$	$\mathbb{Z}_2^3$	$\mathbb{Z}_2 \times \mathbb{Z}_6^2$	$\mathbb{Z}_2 \times \mathbb{Z}_6^2$
$U(1)$				$\mathbb{Z} \times \mathbb{Z}_2$
$SU(2)$				$\mathbb{Z}_2$
$SO(3)$			$\mathbb{Z}_2$	$\mathbb{Z}_2$

Space group #213 $P4_132$

G_0	$E_{3,\infty}^3$	$E_{2,\infty}^2$	$E_{1,\infty}^1$	$E_{0,\infty}^0$
$\varnothing$				$\mathbb{Z}_2$
Z_2^T	$\mathbb{Z}_2$		$\mathbb{Z}_2^2$	$\mathbb{Z}_2$
Z_2		$\mathbb{Z}_2$	$\mathbb{Z}_2$	$\mathbb{Z}_2^2$
$Z_2 \times Z_2^T$	$\mathbb{Z}_2^3$	$\mathbb{Z}_2^2$	$\mathbb{Z}_2^4$	$\mathbb{Z}_2^2$
Z_4		$\mathbb{Z}_2$	$\mathbb{Z}_2$	$\mathbb{Z}_2 \times \mathbb{Z}_4$
Z_6		$\mathbb{Z}_2$	$\mathbb{Z}_6$	$\mathbb{Z}_2 \times \mathbb{Z}_6$
$Z_2 \times Z_2$	$\mathbb{Z}_2^2$	$\mathbb{Z}_2^3$	$\mathbb{Z}_2^3$	$\mathbb{Z}_2^3$
$Z_4 \times Z_4$	$\mathbb{Z}_4^2$	$\mathbb{Z}_2^3$	$\mathbb{Z}_2^3$	$\mathbb{Z}_2 \times \mathbb{Z}_4^2$
$Z_6 \times Z_6$	$\mathbb{Z}_6^2$	$\mathbb{Z}_2^3$	$\mathbb{Z}_2 \times \mathbb{Z}_6^2$	$\mathbb{Z}_2 \times \mathbb{Z}_6^2$
$U(1)$				$\mathbb{Z} \times \mathbb{Z}_2$
$SU(2)$				$\mathbb{Z}_2$
$SO(3)$			$\mathbb{Z}_2$	$\mathbb{Z}_2$

Space group #214 $I4_132$

G_0	$E_{3,\infty}^3$	$E_{2,\infty}^2$	$E_{1,\infty}^1$	$E_{0,\infty}^0$
$\varnothing$				$\mathbb{Z}_2^4$
Z_2^T	$\mathbb{Z}_2$		$\mathbb{Z}_2^6$	$\mathbb{Z}_2^4$
Z_2		$\mathbb{Z}_2^2$	$\mathbb{Z}_2^3$	$\mathbb{Z}_2^6$
$Z_2 \times Z_2^T$	$\mathbb{Z}_2^3$	$\mathbb{Z}_2^4$	$\mathbb{Z}_2^{12}$	$\mathbb{Z}_2^6$
Z_4		$\mathbb{Z}_2^2$	$\mathbb{Z}_2^3$	$\mathbb{Z}_2^5 \times \mathbb{Z}_4$
Z_6		$\mathbb{Z}_2^2$	$\mathbb{Z}_2^2 \times \mathbb{Z}_6$	$\mathbb{Z}_2^5 \times \mathbb{Z}_6$
$Z_2 \times Z_2$	$\mathbb{Z}_2^2$	$\mathbb{Z}_2^6$	$\mathbb{Z}_2^9$	$\mathbb{Z}_2^8$
$Z_4 \times Z_4$	$\mathbb{Z}_4^2$	$\mathbb{Z}_2^6$	$\mathbb{Z}_2^9$	$\mathbb{Z}_2^6 \times \mathbb{Z}_4^2$
$Z_6 \times Z_6$	$\mathbb{Z}_6^2$	$\mathbb{Z}_2^6$	$\mathbb{Z}_2^7 \times \mathbb{Z}_6^2$	$\mathbb{Z}_2^6 \times \mathbb{Z}_6^2$
$U(1)$				$\mathbb{Z} \times \mathbb{Z}_2^5$
$SU(2)$				$\mathbb{Z}_2^4$
$SO(3)$			$\mathbb{Z}_2^3$	$\mathbb{Z}_2^4$

Space group #215 $P\bar{4}3m$

G_0	$E_{3,\infty}^3$	$E_{2,\infty}^2$	$E_{1,\infty}^1$	$E_{0,\infty}^0$
$\varnothing$		$\mathbb{Z}_2$	$\mathbb{Z}_2^2$	$\mathbb{Z}_2^4$
Z_2^T	$\mathbb{Z}_2$	$\mathbb{Z}_2^2$	$\mathbb{Z}_2^{10}$	$\mathbb{Z}_2^4$
Z_2		$\mathbb{Z}_2^4$	$\mathbb{Z}_2^6$	$\mathbb{Z}_2^8$
$Z_2 \times Z_2^T$	$\mathbb{Z}_2^3$	$\mathbb{Z}_2^8$	$\mathbb{Z}_2^{18}$	$\mathbb{Z}_2^8$
Z_4		$\mathbb{Z}_2^4$	$\mathbb{Z}_2^6$	$\mathbb{Z}_2^6 \times \mathbb{Z}_4^2$
Z_6		$\mathbb{Z}_2^3 \times \mathbb{Z}_6$	$\mathbb{Z}_2^6$	$\mathbb{Z}_2^7 \times \mathbb{Z}_6$
$Z_2 \times Z_2$	$\mathbb{Z}_2^2$	$\mathbb{Z}_2^{10}$	$\mathbb{Z}_2^{14}$	$\mathbb{Z}_2^{12}$
$Z_4 \times Z_4$	$\mathbb{Z}_2^2$	$\mathbb{Z}_2^{10}$	$\mathbb{Z}_2^{13} \times \mathbb{Z}_4$	$\mathbb{Z}_2^8 \times \mathbb{Z}_4^4$
$Z_6 \times Z_6$	$\mathbb{Z}_2^2$	$\mathbb{Z}_2^7 \times \mathbb{Z}_6^3$	$\mathbb{Z}_2^{13} \times \mathbb{Z}_6$	$\mathbb{Z}_2^{10} \times \mathbb{Z}_6^2$
$U(1)$		$\mathbb{Z}_2^3$	$\mathbb{Z}_2^2$	$\mathbb{Z} \times \mathbb{Z}_2^6 \times \mathbb{Z}_4$
$SU(2)$		$\mathbb{Z}_2^2$	$\mathbb{Z}_2^2$	$\mathbb{Z}_2^4$
$SO(3)$		$\mathbb{Z}_2^3$	$\mathbb{Z}_2^6$	$\mathbb{Z}_2^4$

Space group #216 $F\bar{4}3m$

G_0	$E_{3,\infty}^3$	$E_{2,\infty}^2$	$E_{1,\infty}^1$	$E_{0,\infty}^0$
$\varnothing$		$\mathbb{Z}_2$	$\mathbb{Z}_2^2$	$\mathbb{Z}_2^2$
Z_2^T	$\mathbb{Z}_2$	$\mathbb{Z}_2^2$	$\mathbb{Z}_2^8$	$\mathbb{Z}_2^2$
Z_2		$\mathbb{Z}_2^3$	$\mathbb{Z}_2^5$	$\mathbb{Z}_2^6$
$Z_2 \times Z_2^T$	$\mathbb{Z}_2^3$	$\mathbb{Z}_2^6$	$\mathbb{Z}_2^{14}$	$\mathbb{Z}_2^6$
Z_4		$\mathbb{Z}_2^3$	$\mathbb{Z}_2^5$	$\mathbb{Z}_2^4 \times \mathbb{Z}_4^2$
Z_6		$\mathbb{Z}_2^2 \times \mathbb{Z}_6$	$\mathbb{Z}_2^5$	$\mathbb{Z}_2^5 \times \mathbb{Z}_6$
$Z_2 \times Z_2$	$\mathbb{Z}_2^2$	$\mathbb{Z}_2^7$	$\mathbb{Z}_2^{11}$	$\mathbb{Z}_2^{10}$
$Z_4 \times Z_4$	$\mathbb{Z}_2^2$	$\mathbb{Z}_2^7$	$\mathbb{Z}_2^{10} \times \mathbb{Z}_4$	$\mathbb{Z}_2^6 \times \mathbb{Z}_4^4$
$Z_6 \times Z_6$	$\mathbb{Z}_2^2$	$\mathbb{Z}_2^4 \times \mathbb{Z}_6^3$	$\mathbb{Z}_2^{10} \times \mathbb{Z}_6$	$\mathbb{Z}_2^8 \times \mathbb{Z}_6^2$
$U(1)$		$\mathbb{Z}_2^3$	$\mathbb{Z}_2^2$	$\mathbb{Z} \times \mathbb{Z}_2^4 \times \mathbb{Z}_4$
$SU(2)$		$\mathbb{Z}_2^2$	$\mathbb{Z}_2^2$	$\mathbb{Z}_2^2$
$SO(3)$		$\mathbb{Z}_2^3$	$\mathbb{Z}_2^5$	$\mathbb{Z}_2^2$

Space group #217 $I\bar{4}3m$

G_0	$E_{3,\infty}^3$	$E_{2,\infty}^2$	$E_{1,\infty}^1$	$E_{0,\infty}^0$
$\varnothing$		$\mathbb{Z}_2$	$\mathbb{Z}_2$	$\mathbb{Z}_2^3$
Z_2^T	$\mathbb{Z}_2$	$\mathbb{Z}_2^2$	$\mathbb{Z}_2^7$	$\mathbb{Z}_2^2$
Z_2		$\mathbb{Z}_2^4$	$\mathbb{Z}_2^3$	$\mathbb{Z}_2^6$
$Z_2 \times Z_2^T$	$\mathbb{Z}_2^3$	$\mathbb{Z}_2^8$	$\mathbb{Z}_2^{12}$	$\mathbb{Z}_2^5$
Z_4		$\mathbb{Z}_2^4$	$\mathbb{Z}_2^3$	$\mathbb{Z}_2^5 \times \mathbb{Z}_4$
Z_6		$\mathbb{Z}_2^3 \times \mathbb{Z}_6$	$\mathbb{Z}_2^3$	$\mathbb{Z}_2^5 \times \mathbb{Z}_6$
$Z_2 \times Z_2$	$\mathbb{Z}_2^2$	$\mathbb{Z}_2^{10}$	$\mathbb{Z}_2^8$	$\mathbb{Z}_2^9$
$Z_4 \times Z_4$	$\mathbb{Z}_2^2$	$\mathbb{Z}_2^{10}$	$\mathbb{Z}_2^8$	$\mathbb{Z}_2^7 \times \mathbb{Z}_4^2$
$Z_6 \times Z_6$	$\mathbb{Z}_2^2$	$\mathbb{Z}_2^7 \times \mathbb{Z}_6^3$	$\mathbb{Z}_2^7 \times \mathbb{Z}_6$	$\mathbb{Z}_2^7 \times \mathbb{Z}_6^2$
$U(1)$		$\mathbb{Z}_2^3$	$\mathbb{Z}_2$	$\mathbb{Z} \times \mathbb{Z}_2^5$
$SU(2)$		$\mathbb{Z}_2^2$	$\mathbb{Z}_2$	$\mathbb{Z}_2^3$
$SO(3)$		$\mathbb{Z}_2^3$	$\mathbb{Z}_2^4$	$\mathbb{Z}_2^3$

Space group #218 $P\bar{4}3n$

G_0	$E_{3,\infty}^3$	$E_{2,\infty}^2$	$E_{1,\infty}^1$	$E_{0,\infty}^0$
$\varnothing$				$\mathbb{Z}_2^4$
Z_2^T	$\mathbb{Z}_2$		$\mathbb{Z}_2^6$	$\mathbb{Z}_2^2$
Z_2		$\mathbb{Z}_2$	$\mathbb{Z}_2$	$\mathbb{Z}_2^8$
$Z_2 \times Z_2^T$	$\mathbb{Z}_2^3$	$\mathbb{Z}_2^2$	$\mathbb{Z}_2^{10}$	$\mathbb{Z}_2^6$
Z_4		$\mathbb{Z}_2$	$\mathbb{Z}_2$	$\mathbb{Z}_2^7 \times \mathbb{Z}_4$
Z_6		$\mathbb{Z}_6$	$\mathbb{Z}_2$	$\mathbb{Z}_2^7 \times \mathbb{Z}_6$
$Z_2 \times Z_2$	$\mathbb{Z}_2^2$	$\mathbb{Z}_2^3$	$\mathbb{Z}_2^5$	$\mathbb{Z}_2^{12}$
$Z_4 \times Z_4$	$\mathbb{Z}_2^2$	$\mathbb{Z}_2^3$	$\mathbb{Z}_2^5$	$\mathbb{Z}_2^{10} \times \mathbb{Z}_4^2$
$Z_6 \times Z_6$	$\mathbb{Z}_2^2$	$\mathbb{Z}_6^3$	$\mathbb{Z}_2^4 \times \mathbb{Z}_6$	$\mathbb{Z}_2^{10} \times \mathbb{Z}_6^2$
$U(1)$		$\mathbb{Z}_2$		$\mathbb{Z} \times \mathbb{Z}_2^7$
$SU(2)$		$\mathbb{Z}_2$		$\mathbb{Z}_2^4$
$SO(3)$		$\mathbb{Z}_2$	$\mathbb{Z}_2^3$	$\mathbb{Z}_2^4$

Space group #219 $F\bar{4}3c$

TABLE II (Continued.)

G_0	$E^3_{3,\infty}$	$E^2_{2,\infty}$	$E^1_{1,\infty}$	$E^0_{0,\infty}$
$\varnothing$				$\mathbb{Z}_2^2$
Z_2^T	$\mathbb{Z}_2$		$\mathbb{Z}_2^5$	
Z_2		$\mathbb{Z}_2$		$\mathbb{Z}_2^6$
$Z_2 \times Z_2^T$	$\mathbb{Z}_2^3$	$\mathbb{Z}_2^2$	$\mathbb{Z}_2^8$	$\mathbb{Z}_2^4$
Z_4		$\mathbb{Z}_2$		$\mathbb{Z}_2^4 \times \mathbb{Z}_4^2$
Z_6		$\mathbb{Z}_6$		$\mathbb{Z}_2^5 \times \mathbb{Z}_6$
$Z_2 \times Z_2$	$\mathbb{Z}_2^2$	$\mathbb{Z}_2^3$	$\mathbb{Z}_2^3$	$\mathbb{Z}_2^{10}$
$Z_4 \times Z_4$	$\mathbb{Z}_2^2$	$\mathbb{Z}_2^3$	$\mathbb{Z}_2^2 \times \mathbb{Z}_4$	$\mathbb{Z}_2^6 \times \mathbb{Z}_4^4$
$Z_6 \times Z_6$	$\mathbb{Z}_2^2$	$\mathbb{Z}_6^3$	$\mathbb{Z}_2^2 \times \mathbb{Z}_6$	$\mathbb{Z}_2^8 \times \mathbb{Z}_6^2$
$U(1)$		$\mathbb{Z}_2$		$\mathbb{Z} \times \mathbb{Z}_2^4 \times \mathbb{Z}_4$
$SU(2)$		$\mathbb{Z}_2$		$\mathbb{Z}_2^2$
$SO(3)$		$\mathbb{Z}_2$	$\mathbb{Z}_2^3$	$\mathbb{Z}_2^2$
Space group #220 $I\bar{4}3d$				
$\varnothing$				$\mathbb{Z}_2 \times \mathbb{Z}_4$
Z_2^T	$\mathbb{Z}_2$		$\mathbb{Z}_2^2$	$\mathbb{Z}_2$
Z_2		$\mathbb{Z}_2$		$\mathbb{Z}_2^3 \times \mathbb{Z}_4$
$Z_2 \times Z_2^T$	$\mathbb{Z}_2^3$	$\mathbb{Z}_2^2$	$\mathbb{Z}_2^3$	$\mathbb{Z}_2^3$
Z_4		$\mathbb{Z}_2$		$\mathbb{Z}_2^2 \times \mathbb{Z}_4^2$
Z_6		$\mathbb{Z}_6$		$\mathbb{Z}_2^2 \times \mathbb{Z}_4 \times \mathbb{Z}_6$
$Z_2 \times Z_2$	$\mathbb{Z}_2^2$	$\mathbb{Z}_2^3$	$\mathbb{Z}_2$	$\mathbb{Z}_2^5 \times \mathbb{Z}_4$
$Z_4 \times Z_4$	$\mathbb{Z}_2^2$	$\mathbb{Z}_2^3$	$\mathbb{Z}_2$	$\mathbb{Z}_2^3 \times \mathbb{Z}_4^3$
$Z_6 \times Z_6$	$\mathbb{Z}_2^2$	$\mathbb{Z}_6^3$	$\mathbb{Z}_6$	$\mathbb{Z}_2^3 \times \mathbb{Z}_4 \times \mathbb{Z}_6^2$
$U(1)$		$\mathbb{Z}_2$		$\mathbb{Z} \times \mathbb{Z}_2^2 \times \mathbb{Z}_4$
$SU(2)$		$\mathbb{Z}_2$		$\mathbb{Z}_2 \times \mathbb{Z}_4$
$SO(3)$		$\mathbb{Z}_2$	$\mathbb{Z}_2$	$\mathbb{Z}_2 \times \mathbb{Z}_4$
Space group #221 $Pm\bar{3}m$				
$\varnothing$		$\mathbb{Z}_2^3$	$\mathbb{Z}_2^5$	$\mathbb{Z}_2^{10}$
Z_2^T	$\mathbb{Z}_2$	$\mathbb{Z}_2^6$	$\mathbb{Z}_2^{20}$	$\mathbb{Z}_2^{10}$
Z_2		$\mathbb{Z}_2^9$	$\mathbb{Z}_2^{15}$	$\mathbb{Z}_2^{14}$
$Z_2 \times Z_2^T$	$\mathbb{Z}_2^3$	$\mathbb{Z}_2^{18}$	$\mathbb{Z}_2^{35}$	$\mathbb{Z}_2^{14}$
Z_4		$\mathbb{Z}_2^9$	$\mathbb{Z}_2^{15}$	$\mathbb{Z}_2^{12} \times \mathbb{Z}_4^2$
Z_6		$\mathbb{Z}_2^9$	$\mathbb{Z}_2^{15}$	$\mathbb{Z}_2^{13} \times \mathbb{Z}_6$
$Z_2 \times Z_2$	$\mathbb{Z}_2^2$	$\mathbb{Z}_2^{21}$	$\mathbb{Z}_2^{30}$	$\mathbb{Z}_2^{18}$
$Z_4 \times Z_4$	$\mathbb{Z}_2^2$	$\mathbb{Z}_2^{21}$	$\mathbb{Z}_2^{29} \times \mathbb{Z}_4$	$\mathbb{Z}_2^{14} \times \mathbb{Z}_4^4$
$Z_6 \times Z_6$	$\mathbb{Z}_2^2$	$\mathbb{Z}_2^{21}$	$\mathbb{Z}_2^{30}$	$\mathbb{Z}_2^{16} \times \mathbb{Z}_6^2$
$U(1)$		$\mathbb{Z}_2^7$	$\mathbb{Z}_2^5$	$\mathbb{Z} \times \mathbb{Z}_2^{12} \times \mathbb{Z}_4$
$SU(2)$		$\mathbb{Z}_2^4$	$\mathbb{Z}_2^5$	$\mathbb{Z}_2^{10}$
$SO(3)$		$\mathbb{Z}_2^7$	$\mathbb{Z}_2^{10}$	$\mathbb{Z}_2^{10}$
Space group #222 $Pn\bar{3}n$				
$\varnothing$				$\mathbb{Z}_2^5$
Z_2^T	$\mathbb{Z}_2$		$\mathbb{Z}_2^7$	$\mathbb{Z}_2^4$
Z_2		$\mathbb{Z}_2^2$	$\mathbb{Z}_2^2$	$\mathbb{Z}_2^9$
$Z_2 \times Z_2^T$	$\mathbb{Z}_2^3$	$\mathbb{Z}_2^4$	$\mathbb{Z}_2^{13}$	$\mathbb{Z}_2^8$
Z_4		$\mathbb{Z}_2^2$	$\mathbb{Z}_2 \times \mathbb{Z}_4$	$\mathbb{Z}_2^8 \times \mathbb{Z}_4$
Z_6		$\mathbb{Z}_2^2$	$\mathbb{Z}_2^2$	$\mathbb{Z}_2^8 \times \mathbb{Z}_6$
$Z_2 \times Z_2$	$\mathbb{Z}_2^2$	$\mathbb{Z}_2^6$	$\mathbb{Z}_2^8$	$\mathbb{Z}_2^{13}$
$Z_4 \times Z_4$	$\mathbb{Z}_2^2$	$\mathbb{Z}_2^6$	$\mathbb{Z}_2^6 \times \mathbb{Z}_4^2$	$\mathbb{Z}_2^{11} \times \mathbb{Z}_4^2$
$Z_6 \times Z_6$	$\mathbb{Z}_2^2$	$\mathbb{Z}_2^6$	$\mathbb{Z}_2^8$	$\mathbb{Z}_2^{11} \times \mathbb{Z}_6^2$
$U(1)$		$\mathbb{Z}_2$		$\mathbb{Z} \times \mathbb{Z}_2^8$

TABLE II (Continued.)

G_0	$E^3_{3,\infty}$	$E^2_{2,\infty}$	$E^1_{1,\infty}$	$E^0_{0,\infty}$
$SU(2)$		$\mathbb{Z}_2$		$\mathbb{Z}_2^5$
$SO(3)$		$\mathbb{Z}_2$	$\mathbb{Z}_2^4$	$\mathbb{Z}_2^5$
Space group #223 $Pm\bar{3}n$				
$\varnothing$		$\mathbb{Z}_2$	$\mathbb{Z}_2^3$	$\mathbb{Z}_2^6$
Z_2^T	$\mathbb{Z}_2$	$\mathbb{Z}_2^2$	$\mathbb{Z}_2^{12}$	$\mathbb{Z}_2^6$
Z_2		$\mathbb{Z}_2^4$	$\mathbb{Z}_2^8$	$\mathbb{Z}_2^{10}$
$Z_2 \times Z_2^T$	$\mathbb{Z}_2^3$	$\mathbb{Z}_2^8$	$\mathbb{Z}_2^{21}$	$\mathbb{Z}_2^{10}$
Z_4		$\mathbb{Z}_2^4$	$\mathbb{Z}_2^8$	$\mathbb{Z}_2^9 \times \mathbb{Z}_4$
Z_6		$\mathbb{Z}_2^4$	$\mathbb{Z}_2^8$	$\mathbb{Z}_2^9 \times \mathbb{Z}_6$
$Z_2 \times Z_2$	$\mathbb{Z}_2^2$	$\mathbb{Z}_2^{10}$	$\mathbb{Z}_2^{17}$	$\mathbb{Z}_2^{14}$
$Z_4 \times Z_4$	$\mathbb{Z}_2^2$	$\mathbb{Z}_2^{10}$	$\mathbb{Z}_2^{17}$	$\mathbb{Z}_2^{12} \times \mathbb{Z}_4^2$
$Z_6 \times Z_6$	$\mathbb{Z}_2^2$	$\mathbb{Z}_2^{10}$	$\mathbb{Z}_2^{17}$	$\mathbb{Z}_2^{12} \times \mathbb{Z}_6^2$
$U(1)$		$\mathbb{Z}_2^3$	$\mathbb{Z}_2^3$	$\mathbb{Z} \times \mathbb{Z}_2^9$
$SU(2)$		$\mathbb{Z}_2^2$	$\mathbb{Z}_2^3$	$\mathbb{Z}_2^6$
$SO(3)$		$\mathbb{Z}_2^3$	$\mathbb{Z}_2^7$	$\mathbb{Z}_2^6$
Space group #224 $Pn\bar{3}m$				
$\varnothing$		$\mathbb{Z}_2$	$\mathbb{Z}_2$	$\mathbb{Z}_2^8$
Z_2^T	$\mathbb{Z}_2$	$\mathbb{Z}_2^2$	$\mathbb{Z}_2^{13}$	$\mathbb{Z}_2^8$
Z_2		$\mathbb{Z}_2^5$	$\mathbb{Z}_2^7$	$\mathbb{Z}_2^{13}$
$Z_2 \times Z_2^T$	$\mathbb{Z}_2^3$	$\mathbb{Z}_2^{10}$	$\mathbb{Z}_2^{25}$	$\mathbb{Z}_2^{13}$
Z_4		$\mathbb{Z}_2^5$	$\mathbb{Z}_2^7$	$\mathbb{Z}_2^{12} \times \mathbb{Z}_4$
Z_6		$\mathbb{Z}_2^5$	$\mathbb{Z}_2^7$	$\mathbb{Z}_2^{12} \times \mathbb{Z}_6$
$Z_2 \times Z_2$	$\mathbb{Z}_2^2$	$\mathbb{Z}_2^{13}$	$\mathbb{Z}_2^{19}$	$\mathbb{Z}_2^{18}$
$Z_4 \times Z_4$	$\mathbb{Z}_2^2$	$\mathbb{Z}_2^{13}$	$\mathbb{Z}_2^{19}$	$\mathbb{Z}_2^{16} \times \mathbb{Z}_4^2$
$Z_6 \times Z_6$	$\mathbb{Z}_2^2$	$\mathbb{Z}_2^{13}$	$\mathbb{Z}_2^{19}$	$\mathbb{Z}_2^{16} \times \mathbb{Z}_6^2$
$U(1)$		$\mathbb{Z}_2^3$	$\mathbb{Z}_2$	$\mathbb{Z} \times \mathbb{Z}_2^{12}$
$SU(2)$		$\mathbb{Z}_2^2$	$\mathbb{Z}_2$	$\mathbb{Z}_2^8$
$SO(3)$		$\mathbb{Z}_2^3$	$\mathbb{Z}_2^7$	$\mathbb{Z}_2^8$
Space group #225 $Fm\bar{3}m$				
$\varnothing$		$\mathbb{Z}_2^2$	$\mathbb{Z}_2^4$	$\mathbb{Z}_2^7$
Z_2^T	$\mathbb{Z}_2$	$\mathbb{Z}_2^4$	$\mathbb{Z}_2^{15}$	$\mathbb{Z}_2^7$
Z_2		$\mathbb{Z}_2^6$	$\mathbb{Z}_2^{11}$	$\mathbb{Z}_2^{11}$
$Z_2 \times Z_2^T$	$\mathbb{Z}_2^3$	$\mathbb{Z}_2^{12}$	$\mathbb{Z}_2^{26}$	$\mathbb{Z}_2^{11}$
Z_4		$\mathbb{Z}_2^6$	$\mathbb{Z}_2^{11}$	$\mathbb{Z}_2^{10} \times \mathbb{Z}_4$
Z_6		$\mathbb{Z}_2^6$	$\mathbb{Z}_2^{11}$	$\mathbb{Z}_2^{10} \times \mathbb{Z}_6$
$Z_2 \times Z_2$	$\mathbb{Z}_2^2$	$\mathbb{Z}_2^{14}$	$\mathbb{Z}_2^{22}$	$\mathbb{Z}_2^{15}$
$Z_4 \times Z_4$	$\mathbb{Z}_2^2$	$\mathbb{Z}_2^{14}$	$\mathbb{Z}_2^{22}$	$\mathbb{Z}_2^{13} \times \mathbb{Z}_4^2$
$Z_6 \times Z_6$	$\mathbb{Z}_2^2$	$\mathbb{Z}_2^{14}$	$\mathbb{Z}_2^{22}$	$\mathbb{Z}_2^{13} \times \mathbb{Z}_6^2$
$U(1)$		$\mathbb{Z}_2^5$	$\mathbb{Z}_2^4$	$\mathbb{Z} \times \mathbb{Z}_2^{10}$
$SU(2)$		$\mathbb{Z}_2^3$	$\mathbb{Z}_2^4$	$\mathbb{Z}_2^7$
$SO(3)$		$\mathbb{Z}_2^5$	$\mathbb{Z}_2^8$	$\mathbb{Z}_2^7$
Space group #226 $Fm\bar{3}c$				
$\varnothing$		$\mathbb{Z}_2$	$\mathbb{Z}_2$	$\mathbb{Z}_2^5$
Z_2^T	$\mathbb{Z}_2$	$\mathbb{Z}_2^2$	$\mathbb{Z}_2^9$	$\mathbb{Z}_2^5$
Z_2		$\mathbb{Z}_2^4$	$\mathbb{Z}_2^5$	$\mathbb{Z}_2^9$
$Z_2 \times Z_2^T$	$\mathbb{Z}_2^3$	$\mathbb{Z}_2^8$	$\mathbb{Z}_2^{17}$	$\mathbb{Z}_2^9$
Z_4		$\mathbb{Z}_2^4$	$\mathbb{Z}_2^5$	$\mathbb{Z}_2^7 \times \mathbb{Z}_4^2$
Z_6		$\mathbb{Z}_2^4$	$\mathbb{Z}_2^5$	$\mathbb{Z}_2^8 \times \mathbb{Z}_6$
$Z_2 \times Z_2$	$\mathbb{Z}_2^2$	$\mathbb{Z}_2^{10}$	$\mathbb{Z}_2^{13}$	$\mathbb{Z}_2^{13}$

TABLE II (Continued.)

G_0	$E^3_{3,\infty}$	$E^2_{2,\infty}$	$E^1_{1,\infty}$	$E^0_{0,\infty}$
$Z_4 \times Z_4$	$\mathbb{Z}_2^2$	$\mathbb{Z}_2^{10}$	$\mathbb{Z}_2^{12} \times \mathbb{Z}_4$	$\mathbb{Z}_2^9 \times \mathbb{Z}_4^4$
$Z_6 \times Z_6$	$\mathbb{Z}_2^2$	$\mathbb{Z}_2^{10}$	$\mathbb{Z}_2^{13}$	$\mathbb{Z}_2^{11} \times \mathbb{Z}_6^2$
$U(1)$		$\mathbb{Z}_2^3$	$\mathbb{Z}_2$	$\mathbb{Z} \times \mathbb{Z}_2^7 \times \mathbb{Z}_4$
$SU(2)$		$\mathbb{Z}_2^2$	$\mathbb{Z}_2$	$\mathbb{Z}_2^5$
$SO(3)$		$\mathbb{Z}_2^3$	$\mathbb{Z}_2^5$	$\mathbb{Z}_2^5$
Space group #227 $Fd\bar{3}m$				
$\varnothing$		$\mathbb{Z}_2$	$\mathbb{Z}_2$	$\mathbb{Z}_2^5$
Z_2^T	$\mathbb{Z}_2$	$\mathbb{Z}_2^2$	$\mathbb{Z}_2^9$	$\mathbb{Z}_2^5$
Z_2		$\mathbb{Z}_2^4$	$\mathbb{Z}_2^5$	$\mathbb{Z}_2^9$
$Z_2 \times Z_2^T$	$\mathbb{Z}_2^3$	$\mathbb{Z}_2^8$	$\mathbb{Z}_2^{17}$	$\mathbb{Z}_2^9$
Z_4		$\mathbb{Z}_2^4$	$\mathbb{Z}_2^5$	$\mathbb{Z}_2^8 \times \mathbb{Z}_4$
Z_6		$\mathbb{Z}_2^4$	$\mathbb{Z}_2^5$	$\mathbb{Z}_2^8 \times \mathbb{Z}_6$
$Z_2 \times Z_2$	$\mathbb{Z}_2^2$	$\mathbb{Z}_2^{10}$	$\mathbb{Z}_2^{13}$	$\mathbb{Z}_2^{13}$
$Z_4 \times Z_4$	$\mathbb{Z}_2^2$	$\mathbb{Z}_2^{10}$	$\mathbb{Z}_2^{13}$	$\mathbb{Z}_2^{11} \times \mathbb{Z}_4^2$
$Z_6 \times Z_6$	$\mathbb{Z}_2^2$	$\mathbb{Z}_2^{10}$	$\mathbb{Z}_2^{13}$	$\mathbb{Z}_2^{11} \times \mathbb{Z}_6^2$
$U(1)$		$\mathbb{Z}_2^3$	$\mathbb{Z}_2$	$\mathbb{Z} \times \mathbb{Z}_2^8$
$SU(2)$		$\mathbb{Z}_2^2$	$\mathbb{Z}_2$	$\mathbb{Z}_2^5$
$SO(3)$		$\mathbb{Z}_2^3$	$\mathbb{Z}_2^5$	$\mathbb{Z}_2^5$
Space group #228 $Fd\bar{3}c$				
$\varnothing$				$\mathbb{Z}_2^3$
Z_2^T	$\mathbb{Z}_2$		$\mathbb{Z}_2^6$	$\mathbb{Z}_2^2$
Z_2		$\mathbb{Z}_2^2$	$\mathbb{Z}_2$	$\mathbb{Z}_2^7$
$Z_2 \times Z_2^T$	$\mathbb{Z}_2^3$	$\mathbb{Z}_2^4$	$\mathbb{Z}_2^{11}$	$\mathbb{Z}_2^6$
Z_4		$\mathbb{Z}_2^2$	$\mathbb{Z}_2$	$\mathbb{Z}_2^6 \times \mathbb{Z}_4$
Z_6		$\mathbb{Z}_2^2$	$\mathbb{Z}_2$	$\mathbb{Z}_2^6 \times \mathbb{Z}_6$
$Z_2 \times Z_2$	$\mathbb{Z}_2^2$	$\mathbb{Z}_2^6$	$\mathbb{Z}_2^6$	$\mathbb{Z}_2^{11}$
$Z_4 \times Z_4$	$\mathbb{Z}_2^2$	$\mathbb{Z}_2^6$	$\mathbb{Z}_2^6$	$\mathbb{Z}_2^9 \times \mathbb{Z}_4^2$
$Z_6 \times Z_6$	$\mathbb{Z}_2^2$	$\mathbb{Z}_2^6$	$\mathbb{Z}_2^6$	$\mathbb{Z}_2^9 \times \mathbb{Z}_6^2$
$U(1)$		$\mathbb{Z}_2$		$\mathbb{Z} \times \mathbb{Z}_2^6$
$SU(2)$		$\mathbb{Z}_2$		$\mathbb{Z}_2^3$
$SO(3)$		$\mathbb{Z}_2$	$\mathbb{Z}_2^4$	$\mathbb{Z}_2^3$
Space group #229 $Im\bar{3}m$				
$\varnothing$		$\mathbb{Z}_2^2$	$\mathbb{Z}_2^3$	$\mathbb{Z}_2^8$
Z_2^T	$\mathbb{Z}_2$	$\mathbb{Z}_2^4$	$\mathbb{Z}_2^{15}$	$\mathbb{Z}_2^8$
Z_2		$\mathbb{Z}_2^7$	$\mathbb{Z}_2^{10}$	$\mathbb{Z}_2^{12}$
$Z_2 \times Z_2^T$	$\mathbb{Z}_2^3$	$\mathbb{Z}_2^{14}$	$\mathbb{Z}_2^{27}$	$\mathbb{Z}_2^{12}$
Z_4		$\mathbb{Z}_2^7$	$\mathbb{Z}_2^{10}$	$\mathbb{Z}_2^{11} \times \mathbb{Z}_4$
Z_6		$\mathbb{Z}_2^7$	$\mathbb{Z}_2^{10}$	$\mathbb{Z}_2^{11} \times \mathbb{Z}_6$
$Z_2 \times Z_2$	$\mathbb{Z}_2^2$	$\mathbb{Z}_2^{17}$	$\mathbb{Z}_2^{22}$	$\mathbb{Z}_2^{16}$
$Z_4 \times Z_4$	$\mathbb{Z}_2^2$	$\mathbb{Z}_2^{17}$	$\mathbb{Z}_2^{22}$	$\mathbb{Z}_2^{14} \times \mathbb{Z}_4^2$
$Z_6 \times Z_6$	$\mathbb{Z}_2^2$	$\mathbb{Z}_2^{17}$	$\mathbb{Z}_2^{22}$	$\mathbb{Z}_2^{14} \times \mathbb{Z}_6^2$
$U(1)$		$\mathbb{Z}_2^5$	$\mathbb{Z}_2^3$	$\mathbb{Z} \times \mathbb{Z}_2^{11}$
$SU(2)$		$\mathbb{Z}_2^3$	$\mathbb{Z}_2^3$	$\mathbb{Z}_2^8$
$SO(3)$		$\mathbb{Z}_2^5$	$\mathbb{Z}_2^8$	$\mathbb{Z}_2^8$
Space group #230 $Ia\bar{3}d$				
$\varnothing$				$\mathbb{Z}_2^4$
Z_2^T	$\mathbb{Z}_2$		$\mathbb{Z}_2^5$	$\mathbb{Z}_2^3$
Z_2		$\mathbb{Z}_2^2$	$\mathbb{Z}_2$	$\mathbb{Z}_2^7$
$Z_2 \times Z_2^T$	$\mathbb{Z}_2^3$	$\mathbb{Z}_2^4$	$\mathbb{Z}_2^9$	$\mathbb{Z}_2^6$

TABLE II (Continued.)

G_0	$E^3_{3,\infty}$	$E^2_{2,\infty}$	$E^1_{1,\infty}$	$E^0_{0,\infty}$
Z_4		$\mathbb{Z}_2^2$	$\mathbb{Z}_2$	$\mathbb{Z}_2^6 \times \mathbb{Z}_4$
Z_6		$\mathbb{Z}_2^2$	$\mathbb{Z}_2$	$\mathbb{Z}_2^6 \times \mathbb{Z}_6$
$Z_2 \times Z_2$	$\mathbb{Z}_2^2$	$\mathbb{Z}_2^6$	$\mathbb{Z}_2^5$	$\mathbb{Z}_2^{10}$
$Z_4 \times Z_4$	$\mathbb{Z}_2^2$	$\mathbb{Z}_2^6$	$\mathbb{Z}_2^5$	$\mathbb{Z}_2^8 \times \mathbb{Z}_4^2$
$Z_6 \times Z_6$	$\mathbb{Z}_2^2$	$\mathbb{Z}_2^6$	$\mathbb{Z}_2^5$	$\mathbb{Z}_2^8 \times \mathbb{Z}_6^2$
$U(1)$		$\mathbb{Z}_2$		$\mathbb{Z} \times \mathbb{Z}_2^6$
$SU(2)$		$\mathbb{Z}_2$		$\mathbb{Z}_2^4$
$SO(3)$		$\mathbb{Z}_2$	$\mathbb{Z}_2^3$	$\mathbb{Z}_2^4$

[1] R. Thorngren and D. V. Else, Gauging spatial symmetries and the classification of topological crystalline phases, Phys. Rev. X **8**, 011040 (2018).
[2] K. S. Brown, *Cohomology of Groups*, Graduate Texts in Mathematics (Springer-Verlag New York, 1982).
[3] Q.-R. Wang and Z.-C. Gu, Towards a complete classification of symmetry-protected topological phases for interacting fermions in three dimensions and a general group supercohomology theory, Phys. Rev. X **8**, 011055 (2018).
[4] Q.-R. Wang and Z.-C. Gu, Construction and classification of symmetry protected topological phases in interacting fermion systems, arXiv:1811.00536 [cond-mat.str-el].
[5] G. Brumfiel and J. Morgan, The pontrjagin dual of 3-dimensional spin bordism, arXiv:1612.02860 [math.AT].
